# Evidence-based recommendations for delivering the diagnosis of X & Y chromosome multisomies in children, adolescents, and young adults: an integrative review

**DOI:** 10.1186/s12887-024-04723-0

**Published:** 2024-04-22

**Authors:** Kirsten A. Riggan, Kelly E. Ormond, Megan A. Allyse, Sharron Close

**Affiliations:** 1 Biomedical Ethics Research Program, Mayo Clinic, Rochester, MN USA; 2 Department of Health Sciences and Technology, Health Ethics and Policy Lab, ETH-Zurich, Zurich, Switzerland; 3 Department of Genetics, Stanford University School of Medicine, Stanford, CA USA; 4 Department of Obstetrics & Gynecology, Mayo Clinic, Rochester, MN USA; 5 Nell Hodgson Woodruff School of Nursing, Emory University, 1520 Clifton Road NE, Atlanta, GA 30342 USA

**Keywords:** X and Y chromosome variations, Sex chromosome variations, Sex chromosome aneuploidies, Sex chromosome multisomies, Genetics, Diagnosis delivery

## Abstract

**Background:**

The diagnosis of supernumerary X & Y chromosome variations has increased following the implementation of genetic testing in pediatric practice. Empirical evidence suggests that the delivery of the diagnosis has a lasting impact on how affected individuals and their parents perceive and adapt to the diagnosis. The purpose of this review is to synthesize the literature to obtain useful recommendations for delivering a pediatric diagnosis of a sex chromosome multisomy (SCM) based upon a growing body of quantitative and qualitative literature on patient experiences.

**Methods:**

We conducted an integrative literature review using PubMed, Web of Science and CINAHL employing keywords “genetic diagnosis delivery,” “genetic diagnosis disclosure,” “sex chromosome aneuploidy,” “Klinefelter syndrome” or “”47, XXY,” “Jacob syndrome” or “47, XYY,” “Trisomy X,” “Triple X” or “47, XXX,” and “48 XXYY from January 1, 2000, to October 31, 2023.

**Results:**

Literature supports that patients and parents value the provision of up-to-date information and connection with supportive resources. Discussion of next steps of care, including relevant referrals, prevents perceptions of provider abandonment and commitment to ongoing support. Proactively addressing special concerns such as disclosing the diagnosis to their child, family, and community is also beneficial. Tables are provided for useful information resources, medical specialties that may be required to support patients, and common misconceptions that interfere with accurate information about the diagnosis.

**Conclusion:**

Patient experiences suggest there should be heightened attention to diagnosis delivery, in reference to the broader ethical and social impacts of a SCM diagnosis. We present recommendations for optimal disclosure of a SCM diagnosis in early and late childhood, adolescence, and young adulthood.

**Supplementary Information:**

The online version contains supplementary material available at 10.1186/s12887-024-04723-0.

## Introduction

Sex chromosome multisomies (SCM) are conditions characterized by one or more extra X or Y chromosomes. Collectively, they have a prevalence of approximately 1 in 500 [1, 2]. Trisomy conditions, including Klinefelter syndrome (47, XXY), Jacob syndrome (47, XYY), and Trisomy X (47, XXX), are the most common, but tetrasomy or pentasomy conditions also occur (e.g., 48, XXYY) [3, 4]. With the exception of tall stature, SCM does not typically present with dysmorphic physical features and phenotypes can encompass a range of physical, neurocognitive, psychological, learning, behavioral, and psychosocial symptoms, and even be undetected [5–11]. Intelligence of people with SCM is in the average to slightly lower-than-average range, although is slightly lower than in sibling controls [8, 12]. It is beyond the scope of this article to comprehensively present the phenotype of each SCM, but many excellent clinical reviews are available [6, 9, 11, 13–16]. The purpose of this paper is to synthesize current literature on diagnosis disclosure of SCM to parents and patients and to offer recommendations for pediatric providers to use when delivering this news.

## Background

Historically, pediatric patients with symptoms of uncertain etiology were referred to geneticists and genetic counselors for diagnostic testing and delivery of genetic diagnoses. Genetic testing is increasingly used as a tool in general pediatric clinical practice [17, 18]. More providers, therefore, will be returning a diagnosis of SCM whether they have received special training in medical genetics or not. It is important for clinicians to be prepared to deliver a diagnosis and follow-up counseling in a sensitive and accurate manner. The genetics literature suggests that clinicians in pediatrics and family medicine exhibit varying degrees of comfort with delivering genetic diagnoses; many feel ill-equipped to present this sensitive information to parents and patients [17, 18]. SCMs have been historically underdiagnosed; only ~ 10–30% of patients were diagnosed in their lifetime [19], typically following significant diagnostic delays. This, however, is changing as genetic testing has become more accessible and is increasingly ordered for symptoms of uncertain etiology and as parents advocate for genetic testing to provide concrete answers to their concerns [20, 21]. Moreover, while the focus of this paper is on diagnoses that occur during childhood, the inclusion of sex chromosomes in noninvasive prenatal screening panels has also increased early detection of SCM [22, 23].

Current literature suggests that the receipt of a genetic diagnosis is recognized as a ‘flashbulb memory,’ and is deeply embedded into the memories of parents and individuals [24]. Ensuring that this moment is as positive as possible is critical for post-diagnosis adaptation and resilience. Many parents have reported negative diagnostic experiences resulting in emotional distress and difficulty processing [25–27]. Poor experiences have ethical implications for the relationship between pediatricians and parents, including erosion of trust and engendering feelings of provider misunderstanding or even abandonment [26]. Parents who receive inaccurate information as part of the diagnosis delivery and/or minimal post-diagnosis support struggle with emotional and mental health concerns that impact their relationship with their child [26, 27]. When the diagnosis of SCM occurs during childhood, diagnostic news is usually shared in one of three potential scenarios: following a pediatric work-up for developmental delay and/or behavioral issues, endocrinology issues related to puberty, and endocrinology issues related to reproductive health. Age at diagnosis also varies by scenario: in one survey, children with Klinefelter who presented with developmental concerns were diagnosed at an average age of 10 years of age (diagnostic delay of 4.8 years from the time of first concern to genetic diagnosis) vs. those with endocrine symptoms had an average age of 21 (diagnostic delay of 2.0 years from the time of first concern to genetic diagnosis) [28]. SCM may also be diagnosed as a secondary or incidental diagnosis in the absence of clinically detectable symptoms, and therefore be unexpected by parents and individuals. As such, there is a wide spectrum of developmental stages where patients may be diagnosed.

While many excellent review articles exist on topics SCM, none as yet have focused upon the process of delivering the diagnosis. As well, no specific protocols or guidelines on diagnosis delivery can be found among national societies including the National Society of Genetic Counselors, the American College of Medical Genetics or the National Association of X & Y Chromosome Variations (AXYS). We endeavored to address this gap by gathering evidence that would provide guidance on how to disclose the diagnosis of SCM to individuals or caregivers.

## Methods

Prior to the introduction of pre-natal cell-free DNA testing in 2011, SCM were rarely diagnosed in childhood. Since the early 2000’s with growing availability and use of this testing and improved awareness of these aneuploidies, new diagnoses have climbed rapidly. Our review was designed to capture what is known about SCM diagnosis disclosure during this period. We chose to employ an integrative literature review method to provide a more comprehensive understanding of this particular healthcare problem [29] and to provide methodological structure using the recommendations of Whittemore & Knafl [30]. While a scoping review aims to map the literature to classify results, this review focuses on synthesis of results that can be applied in practice. Our review focused upon addressing the research question of what evidence exists to inform pediatric clinicians about disclosing and delivering the news that a child has a SCM. An initial search was done to extract key search terms. The search strategies used the following formula: genetic diagnosis disclosure OR genetic diagnosis delivery AND sex chromosome aneuploidy OR Klinefelter syndrome OR 47 XXY OR Jacob’s syndrome OR 47 XYY OR Trisomy X, OR Triple X, OR 47 XXX, OR XXYY. During October of 2023, the databases of PubMed, CINAHL and Web of Science searching the literature from January 1, 2000, to October 31, 2023. Selection of studies for this review were based on the inclusion criteria of relevance to disclosing a diagnosis in a child, adolescent, or young adult and family support post-diagnosis. Additionally, references within discovered articles were reviewed for comprehensiveness. Articles discussing a diagnosis of monosomy conditions (e.g., 45, XO) only were excluded from this review. The monosomy condition known as Turner Syndrome (45, XO) was excluded from this review due to distinct differences in syndrome characteristics compared to multisomies. While some features such as learning challenges overlap with multisomies, 45, XO is unique in its presentation and health risks. For specificity in this article, we use the term “sex chromosome multisomies” rather than “sex chromosome aneuploidy,” which includes monosomy conditions beyond the scope of this review.

This review was based on the inclusion criteria of relevance to disclosing a diagnosis in a child, adolescent, or young adult and family support post-diagnosis with regard to sex chromosome multisomies. While this review was integrative in method, we followed PRISMA-Scoping Review criteria as much as possible to assure rigor in our method [31, 32]. Two reviewers (KR, SC) independently search the literature for title, abstract and full-text screening according to pre-defined limits and using key words. They each read and scored abstracts of articles according to inclusion criteria by labeling as either relevant or irrelevant to the research question. Full text articles were reviewed by KR and SC and consensus for inclusion were achieved through review with MA and KO. Decisions about selection of articles were achieved via bi-monthly team phone meetings from April of 2023-August of 2023. Articles for inclusion were reviewed and decisions were made in-tandem with any discrepancy in agreement achieved through synchronous discussion.

Data from the selected articles were entered into a chart to demonstrate relevant variables to extract.

## Results

**Fig. 1:**
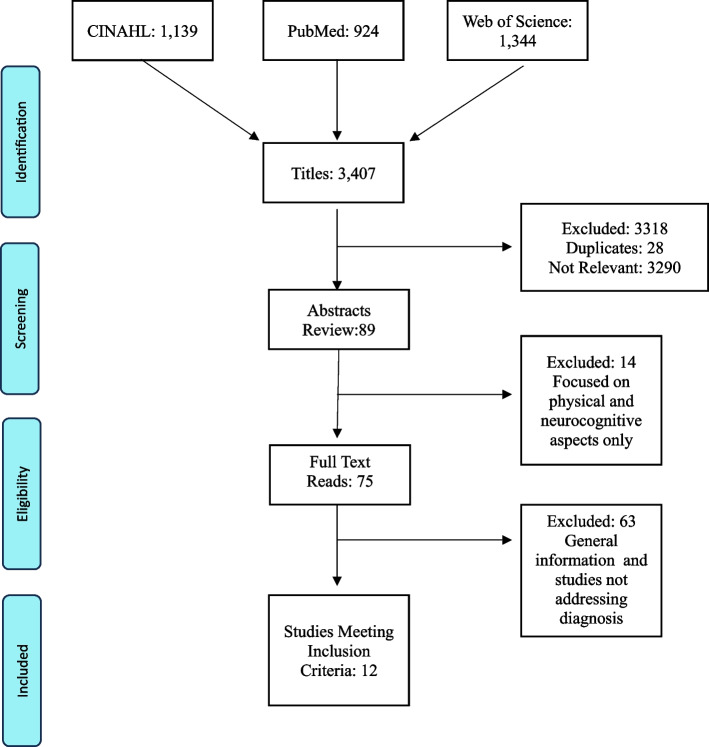
Flowchart of study selection

Our search identified a total of 3,407 articles as potentially relevant from the databases previously described. After reviewing for relevancy by title, 89 were chosen to undergo abstract review. Of these, 75 articles underwent full-text review from which 12 were chosen as relevant for the chart as shown in Fig. 1. Of the 12 that were chosen, 9 were conducted in the US, and 1 each from Australia, Italy and Canada. A descriptive summary of each study is shown in Table 1. We reviewed concerns specific to pediatric populations and included factors that were relevant to delivering the diagnosis. Results from these 12 studies were used to create useful recommendations for clinicians to employ when disclosing the diagnosis and caring for patients with SCM. The results are organized under 6 main headings: Parent Informational Needs, Communication Strategies, Post-diagnosis Support and Adaptation, Timing of Diagnosis and Disclosing the Diagnosis to the Child and Community.

**Table 1:** Descriptive summary of relevant studies

**Reference**	**Author, Year and Country**	**Title**	**Study Design**	**Study Population**	**Diagnosis Disclosure Relevancy**
[25]	Riggan, K., Close, S., Allyse, M. (2020) USA	Family experiences and attitudes about receiving the diagnosis of sex chromosome aneuploidy in a child	Mixed Methods	Parents/Caregivers of Children with a sex chromosome aneuploidy diagnosis *n* = 323	Majority of parents received the diagnosis from a non-genetic medical provider. Few parents reported receiving materials explaining their child’s condition that was up-to-date, accurate and unbiased. Parents receiving the news prior to birth, reported experiencing depression, anxiety and less optimism than parents receiving the diagnosis after birth.
[26]	Jaramillo, C., Nyquist K., Riggan, J, Egginton, S., Phelan, S., Allyse, M. (2019) USA	Delivering the diagnosis of sex chromosome aneuploidy: experiences and preferences of parents and individuals	Descriptive Qualitative	Parents and individuals who received a diagnosis of SCA. Parents* n* = 35 Individuals *n*= 35	Participants expressed almost unanimous interest in more optimistic portrayals of their condition from providers. Participants reported receiving outdated or misleading information about their condition and lacked direction in how to access coordinated care.
[27]	Riggan, K., Gross, B., Close, S., Steinberg, A., Allyse, M (2023) USA	‘Knowledge is Power”: Parental views on the benefits of early diagnosis and awareness of sex chromosome multisomy among pediatric professionals	Inductive qualitative analysis of open-ended survey responses	Parents and individuals receiving diagnosis of SCA. *n* = 20	Parents expressed a feeling of relief at learning of the confirmed diagnosis of their child. They reported frustration that non-medical symptoms related to cognitive and neuropsychiatric were not flagged as potential indicators of SCM. Participants suggested that greater awareness of clinicians, educators and other professionals may lead to earlier diagnosis and intervention.
[33]	Close, S., Sadler, L., Grey, M. (2016) USA	In the Dark: Challenges of caring for sons with Klinefelter syndrome	Mixed methods	Parents with sons who have Klinefelter Syndrome *n* = 40	Participants described feeling uninformed and without support to make decisions about managing care for their sons after learning the diagnosis. Results showed that stress, quality of life and family management struggles varied by parent age.
[34]	Richardson, J.P., Ahlawat, N., Riggan, K.A., Close, S., Allyse, M. (2022) USA	Experiences of individuals receiving a sex chromosome multisomy diagnosis	Mixed Methods	Individuals receiving the diagnosis of sex chromosome multisomy. *n *= 55	Participants expressed dissatisfaction with the delivery of the diagnosis from their health care provider and frustrated by the lack of high-quality information and resources offered. Participants described the social and psychological impact of the diagnosis and how it was delivered.
[35]	Richardson, J.P., Riggan, K.A., Allyse, M (2021) USA	The Expert in the room: Parental advocacy for children with sex chromosome aneuploidies	Descriptive Qualitative Methods	Mothers and father of children with sex chromosome aneuploidies *n*= 34 Mothers *n*= 29 Fathers *n* = 5	Parents reported that they while they suspected that something was developmentally different with their child, they struggled to get a diagnosis. Parents expressed that when they brought specific symptoms up to their provider, they felt dismissed.
[36]	Riggan, K.A., Gross., Close, S., Weinberg, A., Allyse, M. (2021) USA	Prenatal diagnosis of a sex chromosome aneuploidy: Parents experiences	Mixed Methods	Parents receiving a prenatal diagnosis *n* = 122	Most parents were not aware that they could receive a diagnosis of a SCA with prenatal testing. Participants reported that the diagnosis was delivered in a way that emphasized negative attributes and that they received limited support materials. Participants expressed the need for more supportive delivery of the prenatal diagnosis with a focus on parental education and nuanced discussion of potential phenotypes.
[37]	Bourke, E., Snow, A., Herlihy, D. Amor, D. Metcalfe, S. (2014) Australia	A Qualitative Exploration of mothers’ and fathers’ experiences of having a child with Klinefelter syndrome and the process of reaching this diagnosis	Descriptive Qualitative Method	Parents of children born with Klinefelter Syndrome *n *= 15 Mothers *n* = 10 Fathers *n *= 5	Results show that parental experiences were complex and multifaceted including the timing of when the diagnosis was received, who provided the diagnosis, and what information was provided by health care providers.
[38]	Dennis, A., Howell, S., Cordeiro,L, Tartaglia, N. (2015) USA	:How should I tell my child?” Disclosing the diagnosis of sex chromosome aneuploidies	2 Surveys	Parents of children with sex chromosome aneuploidies *n*= 139 Individuals with sex chromosome aneuploidies *n*= 67	Most frequent topics of concern for parents focused on learning disabilities and genetics. Parents reported frequently accessing websites, support groups and discussion with physician. Parental concerns included making the diagnosis conversation age-appropriate, discussing infertility when indicated and thinking ahead about the child’s self-esteem. Parents and individuals endorsed disclosing the diagnosis early, before puberty and continuing discussions over time.
[39]	Aliberti, L., Gagliardi, S., Bigoni, S., Lupo, S., Caracciole, A., Ferlini et al. (2022) Italy	Communicating the diagnosis of Klinefelter syndrome to children and adolescents	Mixed methods	Parents of children and adolescents diagnosed with KS. Parents *n* = 77 Individuals* n* = 41	Results suggest that most parents of children with KS and Individuals with postnatal diagnoses KS should have communication about the condition prior to the age of 14 years, while those with prenatal diagnosis consider that the best timing is between ages of 14-18 years.
[40]	Gratton, NC., Myring, J., Middlemiss, P., Shears, D., Wellesly, D., et al. (2016) United Kingdom	Children with sex chromosome trisomies: parental disclosure of genetic status	Study 1: Descriptive qualitative method using parental focus groups. Study 2: Secondary quantitative analysis of diagnosis disclosure from a large data set	Study 1 34 parents of *N *= 12 females with 47,XXX *N* = 22 males with 47, XYY Study 2: Parents of XXX *n*= 54 XYY *n* = 53 XXY *n* = 19	Study 1: Decisions about disclosure of diagnosis to child were affected by the child; level of cognitive, social and emotional functioning. Parents were more likely to disclose if their child was experiencing difficulties. Study 2; Older children were more likely to know their diagnosis and a substantial proportion tended to be told before 11 years of age. Age was not associated with difference across diagnostic groups.
[41]	Tremblay, I., Van Vliet, G., Gonthier, M., Janvier, A. (2016) Canada	Partnering with parents to disclose Klinefelter syndrome to their child	Case Study and narrative review	Case report of an 11-year-old boy with a prenatal diagnosis with Klinefelter syndrome whose parents and health care provider had not disclosed the diagnosis to him	Disclosure decisions are complex involving cognitive limitations of the child, respect for parental autonomy in deciding when to disclose and the child’s right to know. Health care providers may face an ethical dilemma in considering all viewpoints and may feel uncomfortable when there is misalignment between what parents believe to be in the best interest of their child.

### Parent informational needs

Results demonstrate that parents and individuals who receive news about the diagnosis of SCM desire accurate information about the conditions and how to manage healthcare and other concerns going forward. Their experiences were reflected in several studies where parents and individuals describe that information was not up to date and were misleading [25, 26, 33, 34].

### Communication strategies

Parents reported that they were informed by providers who did not have a background in genetics and were not prepared to counsel them about the condition or what to expect regarding health care needs [25]. They further expressed that when news was delivered, descriptions of the condition were negatively portrayed by them and that this experience that caused exacerbated stress and anxiety about their child [25, 33, 34].

### Post-diagnosis support and adaptation

Parents and individual reported that after learning of the diagnosis, they lacked support for how to manage health care and non-medical symptoms related to potential learning challenges and developmental concerns [33, 36]. They also expressed frustration that when non-medical issues arose during care that their concerns were frequently dismissed by providers [35].

### Timing of diagnosis

Timing of diagnosis and initial counseling needs vary by circumstances regarding whether news is delivered as a pre-natal diagnosis during childhood, adolescence or adulthood. Parents who receive a pre-natal diagnosis reported experiencing depression, anxiety and less optimism than those who learned of the diagnosis after birth [25]. In a 2023 study, parents receiving the diagnosis after birth expressed relief when their concerns and the child’s symptoms were explained by the diagnosis of SCM [27, 35]. Some variations in SCM are associated with reproductive health issues that require special attention and sensitivity about medical surveillance and available treatment options [38].

### Disclosing the diagnosis to the child and community

Genetic diagnoses for SCM may carry some degree of social stigma for individuals and families, so the manner in which genetic information is disclosed must be handle with great sensitivity [33]. Decisions about when and how to disclose the diagnosis to the affected individual and others is highly personal involving special considerations about privacy and autonomy [41]. Decisions about when and how to disclose information to a child is necessarily dependent on a child’s cognitive, emotion and social development [38, 40]. Parental surveys conducted between 2015 and 2022 suggest that children be informed during the peri-pubertal development when they are developmentally more capable of understanding how the genetic diagnosis may be affecting their health and development [38–40]. Healthcare providers need to be sensitive to consideration of parental autonomy in decisions about disclosure of diagnosis to their child and the child’s right know [41]. This review identified 12 papers focused on the topic of diagnosis disclosure in SCM. Evidence from these papers showed five main categories of consideration that clinicians may contemplate when preparing to deliver the diagnosis of the SCM to an individual or caregiver. Focusing on parental information needs, post-diagnostic support and adaptation, consideration of diagnostic timing in the individual’s development and how to assist in disclosing the diagnosis to a child and others provides a patient-centered frame of reference for discussing sensitive genetic information. Results from this review are intended to be combined with what is known about SCM to help prepare clinicians to educate patients and families about how the diagnosis may impact health and well-being.

## Discussion

Pediatric clinicians may feel ill-prepared to discuss health, development, and behavioral concerns of children presenting with SCM. Anticipatory guidance about a child’s developmental trajectory is also difficult; the physical and behavioral phenotype of SCM vary between multiploidy-type and within individuals with similar SCM types. Clinical guidance on optimal health surveillance for SCMs throughout childhood is available in the literature [6, 9, 11, 13–15]. Several studies explored parents’ information needs at the time of diagnosis [14, 25, 26, 33]. Families reported appreciation for receiving up-to-date informational weblinks and lay-person oriented educational materials as shown in Table 2. Informative content from these types of resources includes medical, learning, and behavioral symptoms and needs at future life stages (e.g., late childhood, adolescence, young adult). Parents also reported appreciating candor and veracity from their physicians about what can and cannot be known about their child [42].

**Table 2:** Resources for information about sex chromosome multisomies

Association of X & Y Chromosome Variations (AXYS)	www.genetic.org	The AXYS organization provides information to patients, families, and health care providers online and in printed materials
AXYS Clinic & Research Consortium	https://genetic.org/im-adult-looking-answers/clinics/	Information about Regional Multidisciplinary Clinics within the US
AXYS Klinefelter (47, XXY) CME Course	https://genetic.org/axys-klinefelter-syndrome-cme-course/	These modules provide 2.5 CME credits. The course is accredited by Wake Forest University
National Organization for Rare Disorders	https://rarediseases.org/about/	Patient Advocacy Organization dedicated to individuals with rare diseases and organizations that serve them

Ideally, parents expressed the need to be provided with information about their child’s specific SCM to help alleviate anxiety that may be generated from naïve Internet searches. Proactive referral to specialty care reassures parents that their child’s potential medical needs will be evaluated. Specialty care needs to be customized according to individuals’ SCM type and involve reciprocal communication among clinicians to coordinate care for best outcomes [15, 43]. Medical specialties to consider in building a multidisciplinary care team can be found in Table 3.

**Table 3:** Specialty referrals to consider post-diagnosis based on need

Specialty Referrals
Genetics/Genetic Counseling
Endocrinology
Neuropsychology
Gastroenterology
Reproductive GYN and Urology
Psychiatry
Behavioral Specialist
Social Work
Speech and Language Therapy
Occupational Therapy

With regard to explanation of physical neurocognitive and behavioral traits, studies have shown that individuals and parents appreciate learning that “classic” phenotypes and descriptions of SCMs may be misleading as historically they were informed by more seriously affected individuals, with clinically diagnosed symptoms prompting karyotyping, and that they or their child will grow, develop and mature on their own trajectory. Predictions about future development vary widely [27, 34, 35].

As in other literature about sharing a new diagnosis, parents and individuals frequently cite the mode of delivery as having significant impact on clinical experience and resilience [26, 34]. A recent study demonstrated that parents who discovered the diagnosis through the return of laboratory results or a brief phone call were often upset that the diagnosis was communicated in an impersonal or inconvenient setting (e.g., in a grocery store or business meeting) with limited opportunity for discussion and absorption of the information [26]. A preferred setting is in person, with both parents present if appropriate, in a private location, and with proactive conversations about the desirability of having the child present. Parents expressed a strong preference for these optimal settings, even if it meant a delay in the delivery of the diagnosis [26, 34]. Clinical workflow and processes (e.g., automatic release of results to the electronic health record) may prevent ideal communication of the diagnosis. In these circumstances, it is important to consider that arranging for an in-person appointment would best be scheduled to discuss the diagnosis to determine next steps of care.

Our review suggests several strategies for pediatricians delivering a diagnosis of SCM. Current literature provides helpful guidance on delivering genetic diagnoses more broadly [44–47], and we recommend a strategy for the process of delivering an SCM diagnosis with helpful details that are informed by the SCM literature as shown in Table 4 [26, 27, 33–37]. Recommendations include providing a clear and brief definition of the diagnosis and exploring what parents and/or individuals already know and what their concerns are (e.g., “Can you tell me what you have heard/know about SCMs?). Many individuals and parents, particularly those where there may be suspicion of a SCM diagnosis or those who received a preliminary telephone call with diagnostic results, have already consulted the Internet and various social media sources. Non-evidence-informed sources often contain medicalized images designed to emphasize physical trait differences and uninformed lay opinions about medical management.

**Table 4:** Recommendations for the process of delivering a SCM diagnosis

Prepare for the Visit	• Inform yourself about the specific diagnosis prior to the communication • Resources for providers are offered in Table 2 • Partner with a genetic counselor who is well-equipped to explain genetic underpinnings and specific information according to SCM
Mode and Manner of Delivery	• Allow enough uninterrupted time to spend with patients/families • When possible, deliver the diagnosis in-person or by Telehealth technology rather than over the telephone • If circumstances prohibit in-person delivery, the diagnosis should be communicated when the parent is in a quiet and confidential location • Providers should proactively inquire as to whether the parent wishes their child to be present when the diagnosis is delivered
Parent Questions & Concerns	• Begin the visit by inquiring about their specific concerns and questions • Check back frequently to be sure that information is being understood • Suggest that this conversation may be ongoing and may need to occur over more than one visit to allow parents time to process the diagnosis and determine their questions • Provide reassurance that the child’s health and well-being is the primary focus of care
Explanation of SCM Condition	• Provide a brief and clear explanation of the specific SCM condition and describe any additional special health surveillance that might be required • Direct patients and families to well-vetted and accurate information sources such as www.genetic.org or https://rarediseases.org/ • Advise patients and families to be cautious about receiving information from general search engines and social media sites • Provide supportive reference material ideally written in lay language
SCM Prevalence & Genetics	• Become familiar with prevalence by SCM: 47, XXY (1 in 600), 47, XYY (1 in 1000),47, XXX (1in 1000) and 48, XXYY (1 in 80,000) • Explain that physical, behavioral, and psychosocial features vary among the SCMs and among individuals • Explain that the SCM is a random event during egg and sperm development and that it was caused by anything the parents did or didn’t do before or during the pregnancy • Be prepared to offer a warm hand-off to a genetic counselor for more information
Follow-up Care and Next Steps	• Inform parents of medical management and health surveillance recommended for the specific SCM • Be proactive in assembling a team of specialists for referrals as required
Additional Support Considerations	• Be ready to refer parents and individuals to the national advocacy organization (AXYS) and for social support through regional and local support groups • Referral to a professional counselor may be beneficial for additional emotional processing of the diagnosis • During the course of the child’s care, be prepared to counsel parents about how they would like to disclose the diagnosis to their child, to family members and to others

A summary of common misconceptions about SCMs concerning impact on intelligence, behavior, sexual orientation, gender identity, and reproductive health [8, 16, 44, 48–53] is shown in Table 5.

**Table 5:** Common misconceptions about Sex Chromosome Multisomies (SCM)

Misconception	Explanations
Persons with SCM are more likely to exhibit criminal behaviors	Studies from the 1960’s and 1970’s conducted in prisons or other institutionalized populations erroneously concluded that people with SCM, especially Jacob syndrome, are at higher risk of sexual deviance and criminality. These conclusions are not supported by larger observational studies of children diagnosed as infants or prenatally, as well as studies characterizing neuropsychology of these conditions. Individuals with some SCM conditions do experience challenges with executive function, impacting judgment, decision-making, emotional regulation, and impulse control that may render them more likely to interact with legal and educational authorities and this should be clearly distinguished from criminality
SCM are associated with sexual orientation or gender identity	Based on the association with the sex chromosomes, there is a common misconception that SCM impacts gender identity and/or sexual orientation. A distinction should be drawn between SCM and intersex conditions. Although there is a spectrum of gender and sexuality in individuals who have SCM, there little evidence showing links between SCM and gender dysmorphia and/or sexuality
All persons with SCM are infertile	Because SCM have historically been under-diagnosed, many individuals previously received an unexpected diagnosis when seeking fertility care. This has led to a misconception that all individuals with SCM are infertile and cannot have biological children. People with SCM may have reduced fertility. For example, Klinefelter syndrome does result in testicular insufficiency and azoospermia, but advances in assisted fertility procedures allow approximately half of individuals with Klinefelter to biologically reproduce. Several clinics in the US now offer fertility procedures to achieve pregnancy with gametes from individuals with SCM

Providers with less familiarity with SCM can prepare themselves for this conversation by understanding the phenotypic spectrum of these conditions, as well as common misperceptions. It may be helpful to ask parents what kind of information they have encountered and when appropriate, provide them with counter examples from the medical and patient experience literature.

In articles by Jaramillo et al. (2019) and Richardson et al. (2021) parents were reported to speak positively about conversations that included contextual information about the condition—including the number of children living with the condition including that many individuals remain undiagnosed in their lifetime, the availability of other families of children with a diagnosis, and contacts for support organizations [26, 34]. Parent participants stressed that the diagnosis should be presented as a positive development in their child’s medical journey and that having a diagnosis will improve clinical management and assist in gathering resources rather than as a negative event [25]. Parents also emphasized the need to avoid apologetic language and suggestions that the diagnosis may in any way limit future opportunities for the child. They also valued reassurance that the diagnosis itself would not influence their relationship with their child [27].

Extensive knowledge about genetics is not required to deliver the diagnosis but part of the informational process should include a warm hand off to medical genetics and genetic counseling. If specialty services are not available locally, research has shown that telehealth approaches show comparative effectiveness in genetic counseling [54]. During the process, providers should clarify that SCM are different from autosomal aneuploidy conditions (e.g., Trisomy 21) to minimize confusion about the potential prognosis. The use of colloquial terms such as ‘female’ or ‘male’ chromosomes in explanations of genetic etiology can also be confusing. It is preferable to use terms such as ‘extra x’ or ‘extra y’ when communicating the karyotype to prevent misunderstanding or attribution of their child’s gender and sexual identity to the aneuploidy. Terms such as ‘super female’ or ‘super male’ are also inaccurate and outmoded. While emphasized in the medical and lay literature, parents report that extensive discussion of potential infertility detracts from their immediate concerns about the health and social issues their child might encounter [27].

Another highly valued resource for parents is the provision of educational materials that provide social and emotional context in addition to describing the phenotype and symptoms. Provided materials should be reflective of the latest scholarship, easy to understand, and free of technical jargon, but this is an unmet need at present. Some patient-facing materials have been developed, especially for Klinefelter syndrome [55, 56], but materials for less prevalent SCM conditions may be difficult to access, especially in languages other than English. Parents strongly desire a balanced presentation of both positive and negative aspects of the condition [26]. While the literature has historically been focused on symptom characterization, studies are beginning to highlight relative strengths of children and individuals with these conditions, including kindness, honesty, being eager to please, and love of learning [57, 58], which can be contextualized in reference to the parent–child relationship.

Parents express a strong desire for partnership with clinicians on an actionable plan for clinical and social care moving forward [27, 33]. In the pediatric context, a SCM diagnosis is delivered for a known child and, with some exceptions, is typically in response to observed symptoms or parent concerns. At this stage, parents’ primary concern is how to support their child and navigate the health and social impacts of the diagnosis. Parents find it most helpful when the clinician focuses the conversation on how to receive individualized help and support for their child. Discussion of concrete next steps, including specialty care and available resources for social and educational support are perceived to be the most helpful in addressing immediate concerns raised by the diagnosis [27].

Parents may express a wide spectrum of responses to the diagnosis, which often reflect their diagnostic journey. Some parents reported feelings of relief at learning of the diagnosis, especially if they experienced a lengthy diagnostic delay or if genetic testing was for differential diagnoses of more serious conditions [25, 33, 37]. They also expressed frustration if their child had not receive an earlier diagnosis, was misdiagnosed, and/or received ineffective treatments [25]. Some parents reported feeling overwhelmed and having difficulty processing news of the diagnosis. Parent referral for professional counseling may be beneficial to understand and work through the potential implications for their family dynamics and any complicated feelings that may arise from a genetic diagnosis in a child [27, 34].

Connection with local or national support groups offers parents an established network of support, access to non-medical resources, and a place for additional context on parenting and day-to-day management. Organizations such as the Association for X and Y Chromosome Conditions (AXYS) and the XXYY Project support families diagnosed with SCM in the U.S. and internationally as shown in Table 2. Private social media groups have also grown in popularity as a mechanism to connect parents with shared experiences, although these groups are subject to online group dynamics, including potential exclusion of parents who experience greater challenges or with more involved phenotypes, or pressure towards a ‘right’ way to parent a child with SCM. Both parents and individuals with SCM find it helpful to connect with other families farther along the same journey, see examples of adults with the same condition leading typical lives, and discuss strategies for daily life management [34, 35].

Comprehensive medical care may be difficult to access post-diagnosis; there are only a handful of specialty SCM clinics in the U.S. [43, 59]. Because of the lack of coordinated care for these conditions, parents are often placed in the position of becoming ‘experts’ about their child’s condition and symptoms, advocating for necessary referrals, managing communication between specialty providers, and educating providers on the condition and its sequelae [14, 60]. Decisions about initiation of testosterone therapy and fertility preservation may also weigh on parents of children with Klinefelter’s syndrome, especially in the peri-pubertal period. Published clinical guidance recommends referral to endocrinology at first signs of puberty for this time-sensitive discussion, although there is variance among providers regarding optimal timing for fertility preservation [15, 61].

Parents cited a particular need for help in attaining educational support. Learning disorders are common among children with SCM and may be one of the first ‘soft’ symptoms identified by parents or educators [27, 58]. Parents stressed challenges over navigating special education services, including state-specific requirements for Individualized Education Plans (IEP) or 504 plans. Access to services often require a letter from a physician confirming the diagnosis, their eligibility for special education services under the categories of disability outlined in the federal Individuals with Disabilities Education Act (IDEA), and recommended accommodations.

Anticipating this need may relieve the advocacy burdens experienced by many parents [35]. Teachers and administrators may have limited understanding of the impact of the SCM on classroom behavior and learning; the importance of strong partnerships between parents and schools is also cited by parents as key to the thriving of their child in educational environments [27, 35, 60]. It may be useful to refer parents to the helpful recommendations for educators available in Thompson et al. [60].

It is common for SCM to be diagnosed following delayed or incomplete pubertal development or following an infertility work-up, although this is more typical of early to mid-adulthood. As adolescents and young adults (AYAs) begin to develop greater autonomy over their medical care, they may raise confidential concerns with pediatric providers, including indications for SCM related to sexual development and reproductive health concerns [62, 63]. Respect for this autonomy must be balanced with their ability to understand and process medical information as well as the need for support following a genetic diagnosis. Emotional maturity, comprehension, and communication are impacted in SCM; reports in our review indicate that young adults felt blindsided by the diagnosis and struggled to comprehend its implications [26, 34]. It is helpful to proactively engage AYA patients about whether they wish to have a parent or other support person present when the diagnosis is delivered.

Special sensitivity should be given towards the impact of the diagnosis on AYAs self-image and self-esteem. Individuals diagnosed prior to the millennium reported that objectification of genitalia, unnecessary genital exams, and remarks on sexuality were more common at that time. Those diagnosed more recently reported fewer problematic experiences, but mentioned that off-hand comments about infertility, genital size, or gender expression are deeply distressing and should not be made. These comments may be remembered for many years after diagnosis and may impact perceptions of self-worth or generate misperceptions about their sexuality and relationships [34]. A survey of AYAs with Klinefelter syndrome found that one-fifth of respondents expressed that differences in physical appearance, including small testes, gynecomastia, and lack of muscle mass and these were some of the most difficult aspects of living with the condition. Instances of bullying were also reported [64].

For AYAs, a diagnosis may bring clarity to prior learning or social struggles and diffuse feelings of failure, but it may also reinforce feelings of difference from peers [34, 65–67]. Learning of a genetic diagnosis is often an identity-forming event; [65, 66] some AYAs have noted difficulty developing trusting relationships with their providers and finding information on the lived experience of AYAs with SCM [67]. Adolescents and young adults may benefit from referrals to counseling or psychology given the direct implications on self-image, sexuality and romantic relationships, and future education and employment plans [67]. The same survey of AYAs with Klinefelter syndrome found that nearly one-third stated that psychological challenges including depression, anxiety, low self-esteem, mood instability were of the greatest concern followed by reproductive health concerns [64]. Other worries of AYAs include how to communicate the diagnosis and its reproductive impact to partners or friends [61], managing side effects related to hormone replacement therapy and the transition to adult care [62, 63, 67].

Parents should be encouraged to carefully think through when to disclose the SCM diagnosis to their child [68]. There are diverse views on diagnosis disclosure; some parents prefer early disclosure as a means of normalizing the condition, whereas others believe the diagnosis should only be disclosed when they feel their child is developmentally ready, even as late as early adulthood. Parents may want to avoid early disclosure to prevent internalized stigma [33] or to minimize negative impact on self-esteem [38, 69] although at least one study suggests perceived stigmatization among adolescents and adults with Klinefelter’s is low [70]. The literature suggests that most parents of children diagnosed in early or late childhood believe the diagnosis should be communicated to a child before they reach their teen years and that perception of condition severity influences this decision [38–40]. Parents may also elect to inform their child of the SCM though a ‘seed planting’ approach, using developmentally appropriate language in early childhood, followed by increasingly in-depth disclosure as the child ages. This strategy may mitigate child anxiety or suspicion while allowing parents to individualize the framing of the conversation to the personality, abilities, and needs of their child. Additional guidance on disclosing a genetic diagnosis is available in the literature [38, 41].

On rare occasion, a parent may request to withhold the diagnosis from a child or delay communication of diagnosis until adulthood. This may raise ethical dilemmas for providers regarding their clinical duty to medically care for and act in the best interests of their pediatric patients while also respecting parental authority [71–73]. It is generally not advisable to hide a SCM diagnosis from an affected individual because it may result in confusion and harm as they navigate educational, social, and reproductive difficulties [73]. Many adults with SCM report that they wish their diagnosis was discovered or disclosed much earlier in life to give context to their struggles and for early initiation of therapies [34]. It may be helpful to discuss the potential negatives of postponed disclosure with parents, including child suspicion and anxiety of a ‘secret’ condition, the potential for inopportune disclosure in overheard conversations or through their child’s own exploration of their symptoms, and resentment that they were not told earlier [38, 41, 74, 75]. Patients with SCM have a right to understand the physical, cognitive, and reproductive impact of their condition. Pediatric patients’ increasing autonomy as they mature should also be respected; early disclosure permits pediatric patients to engage in decision-making and assent to their care [72, 73]. In cases of parental request for nondisclosure, providers should explore the reasons for this request and clearly communicate their comfort with and limits of nondisclosure (e.g., will not lie or evade answering a direct question from the pediatric patient about the cause of their symptoms; will disclose in a developmentally appropriate manner as it becomes relevant to their reproductive or medical care), while also seeking opportunities to compromise and engage parents in the disclosure process (e.g., joint communication with parents; referral to a genetic counselor for this discussion).

Finally, parents may need assistance weighing the positives and negatives of disclosure to extended family, friends, and other community members. Similar to disclosure to a child, parents’ decision to inform extended family and educational staff is impacted by their child’s functioning and support needs [40]. Selective disclosure to teachers, child care workers, and/or others involved in the ongoing care of the child may allow for greater understanding and empathy towards behaviors, needs, and struggles and avoid perceptions that a child is simply poorly behaved or disciplined [27, 39]. In other circumstances, such as sporting activities or other transient social events, disclosure may raise privacy concerns or have heightened potential that the condition could be incidentally disclosed to peers. Disclosure also carries the risk that typical age-related behaviors may be over-attributed to the SCM or raise concerns about the best way to correct or mitigate unwanted behaviors with this additional knowledge.

Of the 12 papers reviewed on diagnosis disclosure, six were conducted in the United States and the others were from Australia, United Kingdom and Italy. Since the body of evidence is very limited, it is difficult to assess any country-specific findings for comparison. However, issues and concerns about diagnosis disclosure were well-aligned among the papers.

### Strengths & limitations

Limitations of integrative reviews include potential problems with accuracy, bias, or rigor as different methodologies are used across included studies. The major strength of this review, however, is that it addresses a gap in the literature about evidence-based recommendations for genetic diagnosis disclosure in SCM and offers useful recommendations that can be used in practice by pediatric health care providers.

## Conclusion

Our review has identified important factors to be considered when informing a patient or caregiver about the diagnosis of SCM. This review is combined with what is known about SCM to provide clinicians with information and insight as they prepare to inform patients and caregivers about the diagnosis. We offer practical strategies to navigate the challenging responsibility of delivering a SCM diagnosis and avoid the more ethically concerning experiences previously reported by parents and individuals. Pediatric clinicians are in an optimal position to provide sensitive delivery of an SCM diagnosis and post-diagnostic support to children, AYAs, and their families. More research is needed to understand how patients and caregivers receive the diagnosis of SCM and how their needs and preferences can be incorporated into practice for clinicians who deliver these diagnoses.

### Supplementary Information

**Supplementary Material 1. **

## Data Availability

Data for the literature review has been uploaded in supplementary files. These files may also be accessed here: https://pubmed.ncbi.nlm.nih.gov/collections/63798536/?sort=pubdate.

## Abbreviations

SCM: Sex chromosome multisomies
U.S.: United States
IEP: Individualized Education Plan
IDEA: Individuals with Disabilities Act
AYA: Adolescents and Young Adults
