# Supplementary material for: Evidence-based recommendations for delivering the diagnosis of X & Y chromosome multisomies in children, adolescents, and young adults: an integrative review
Source: BMC Pediatr. 2024 Apr 22;24:263. doi: 10.1186/s12887-024-04723-0 (PMC11034074; doi:10.1186/s12887-024-04723-0)
Supplement: Supplementary file 1 — Supplementary Material 1. [file 12887_2024_4723_MOESM1_ESM.zip › Data file for PubMed Search Diagnosis Disclosure in SCM.pdf]

1. Fentiman IS. Risk factors for male breast cancer. Am J Transl Res. 2023 Dec
15;15(12):6918-6925. eCollection 2023. Review. PubMed [citation] PMID: 38186995,
PMCID: PMC10767513

2. Rao ZZ, Tang ZW, Wen J. Advances in drug resistance of triple negative breast
cancer caused by pregnane X receptor. World J Clin Oncol. 2023 Sep
24;14(9):335-342. doi: 10.5306/wjco.v14.i9.335. Review. PubMed [citation] PMID:
37771631, PMCID: PMC10523191

3. Bashiri Z, Gholipourmalekabadi M, Khadivi F, Salem M, Afzali A, Cham TC, Koruji
M. In vitro spermatogenesis in artificial testis: current knowledge and clinical
implications for male infertility. Cell Tissue Res. 2023 Dec;394(3):393-421. doi:
10.1007/s00441-023-03824-z. Epub 2023 Sep 18. Review. PubMed [citation] PMID:
37721632

4. Gold S, Huang C, Radi R, Gupta P, Felner EI, Haw JS, Childress K, Sokkary N,
Tangpricha V, Goodman M, Yeung H. Dermatologic care of patients with differences
of sex development. Int J Womens Dermatol. 2023 Sep 5;9(3):e106. doi:
10.1097/JW9.0000000000000106. eCollection 2023 Oct. Review. PubMed [citation]
PMID: 37671254, PMCID: PMC10473340

5. Jia H, Wang W, Zhou Z, Chen Z, Lan Z, Bo H, Fan L. Single-cell RNA sequencing
technology in human spermatogenesis: Progresses and perspectives. Mol Cell
Biochem. 2023 Sep 2. doi: 10.1007/s11010-023-04840-x. [Epub ahead of print]
Review. PubMed [citation] PMID: 37659974

6. Kyaw TT, Abdou A, Arunjaroensuk S, Nakata H, Kanazawa M, Pimkhaokham A. Effect of
chemical and electrochemical decontamination protocols on single and
multiple-used healing abutments: A comparative analysis of contact surface area,
micro-gap, micro-leakage, and surface topography. Clin Implant Dent Relat Res.
2023 Dec;25(6):1207-1215. doi: 10.1111/cid.13269. Epub 2023 Sep 1. PubMed
[citation] PMID: 37654160

7. Ayyavoo A. Gynecomastia. Indian J Pediatr. 2023
Oct;90(10):1013-1017. doi:
10.1007/s12098-023-04810-7. Epub 2023 Aug 18. Review. PubMed
[citation] PMID:
37592101

8. Jones TH, Dobs AS, Randeva H, Moore W, Parkin JM. Leflutrozole in
male
obesity-associated hypogonadotropic hypogonadism: Ph 2b double-blind
randomised
controlled trial. Eur J Endocrinol. 2023 Sep 1;189(3):297-308. doi:
10.1093/ejendo/lvad099. PubMed [citation] PMID: 37579053

9. Ramgopal MN, Castagna A, Cazanave C, Diaz-Brito V, Dretler R, Oka
S, Osiyemi O,
Walmsley S, Sims J, Di Perri G, Sutton K, Sutherland-Phillips D, Berni
A, Latham
CL, Zhang F, D'Amico R, Pascual Bernáldez M, Van Solingen-Ristea R,
Van Eygen V,
Patel P, Chounta V, Spreen WR, et al. Efficacy, safety, and
tolerability of
switching to long-acting cabotegravir plus rilpivirine versus
continuing
fixed-dose bictegravir, emtricitabine, and tenofovir alafenamide in
virologically
suppressed adults with HIV, 12-month results (SOLAR): a randomised,
open-label,
phase 3b, non-inferiority t... Lancet HIV. 2023 Sep;10(9):e566-e577.
doi:
10.1016/S2352-3018(23)00136-4. Epub 2023 Aug 8. PubMed [citation]
PMID: 37567205

10. Chen C, Luo Y, Hou X, Li T. Clinical characterization of epilepsy
in children
with chromosomal aberration 47, XXY. Brain Behav. 2023
Aug;13(8):e3178. doi:
10.1002/brb3.3178. Epub 2023 Jul 21. PubMed [citation] PMID: 37479950,
PMCID:
PMC10454333

11. Zhu FC, Zhong GH, Huang WJ, Chu K, Zhang L, Bi ZF, Zhu KX, Chen Q,
Zheng TQ,
Zhang ML, Liu S, Xu JB, Pan HX, Sun G, Zheng FZ, Zhang QF, Yi XM,
Zhuang SJ,
Huang SJ, Pan HR, Su YY, Wu T, et al. Head-to-head immunogenicity
comparison of
an Escherichia coli-produced 9-valent human papillomavirus vaccine and
Gardasil 9
in women aged 18-26 years in China: a randomised blinded clinical

trial. Lancet
Infect Dis. 2023 Nov;23(11):1313-1322. doi: 10.1016/
S1473-3099(23)00275-X. Epub
2023 Jul 17. PubMed [citation] PMID: 37475116

12. Tallaksen HBL, Johannsen EB, Just J, Viuff MH, Gravholt CH, Skakkebæk A. The
multi-omic landscape of sex chromosome abnormalities: current status and future
directions. Endocr Connect. 2023 Aug 1;12(9). pii: e230011. doi:
10.1530/EC-23-0011. Review. PubMed [citation] PMID: 37399516, PMCID:
PMC10448593

13. Rogol AD. Sex chromosome aneuploidies and fertility: 47,XXY, 47,XYY, 47,XXX and
45,X/47,XXX. Endocr Connect. 2023 Aug 1;12(9). pii: e220440. doi:
10.1530/EC-22-0440. Review. PubMed [citation] PMID: 37399523, PMCID:
PMC10448573

14. Genchi VA, Palma G, Sorice GP, D'Oria R, Caccioppoli C, Marrano N, Biondi G,
Caruso I, Cignarelli A, Natalicchio A, Laviola L, Giorgino F, Perrini S.
Pharmacological modulation of adaptive thermogenesis: new clues for obesity
management? J Endocrinol Invest. 2023 Nov;46(11):2213-2236. doi:
10.1007/s40618-023-02125-0. Epub 2023 Jun 28. Review. PubMed [citation] PMID:
37378828, PMCID: PMC10558388

15. Bhatt SP, Rabe KF, Hanania NA, Vogelmeier CF, Cole J, Bafadhel M, Christenson SA,
Papi A, Singh D, Laws E, Mannent LP, Patel N, Staudinger HW, Yancopoulos GD,
Mortensen ER, Akinlade B, Maloney J, Lu X, Bauer D, Bansal A, Robinson LB,
Abdulai RM; et al. Dupilumab for COPD with Type 2 Inflammation Indicated by
Eosinophil Counts. N Engl J Med. 2023 Jul 20;389(3):205-214. doi:
10.1056/NEJMoa2303951. Epub 2023 May 21. PubMed [citation] PMID:
37272521

16. Guven DC, Yildirim HC, Kus F, Erul E, Kertmen N, Dizdar O, Aksoy S. Optimal
adjuvant treatment strategies for TNBC patients with residual disease after
neoadjuvant treatment. Expert Rev Anticancer Ther. 2023 Jul-Dec;23(10):1049-1059.
doi: 10.1080/14737140.2023.2218090. Epub 2023 May 31. Review. PubMed [citation]

PMID: 37224429

17. Juul A, Gravholt CH, De Vos M, Koledova E, Cools M. Individuals with numerical
and structural variations of sex chromosomes: interdisciplinary management with
focus on fertility potential. Front Endocrinol (Lausanne). 2023 May 5;14:1160884.
doi: 10.3389/fendo.2023.1160884. eCollection 2023. Review. PubMed [citation]
PMID: 37214245, PMCID: PMC10197804

18. Grande G, Graziani A, Di Mambro A, Selice R, Ferlin A. Osteoporosis and bone
metabolism in patients with Klinefelter syndrome. Endocr Connect. 2023 Jul
5;12(8). pii: e230058. doi: 10.1530/EC-23-0058. Review. PubMed [citation] PMID:
37166398, PMCID: PMC10388662

19. Dong F, Ping P, Ma Y, Chen XF. Application of single-cell RNA sequencing on human
testicular samples: a comprehensive review. Int J Biol Sci. 2023 Apr
9;19(7):2167-2197. doi: 10.7150/ijbs.82191. eCollection 2023. Review. PubMed
[citation] PMID: 37151874, PMCID: PMC10158017

20. Ridder LO, Berglund A, Stochholm K, Chang S, Gravholt CH. Morbidity, mortality,
and socioeconomics in Klinefelter syndrome and 47,XYY syndrome: a comparative
review. Endocr Connect. 2023 Apr 26;12(5). pii: e230024. doi: 10.1530/EC-23-0024.
Print 2023 May 1. Review. PubMed [citation] PMID: 37098811, PMCID: PMC10160544

21. De Sanctis V, Soliman AT, Tzoulis P, Daar S, Di Maio S, Kattamis C. Unilateral
breast enlargement in males during adolescence (10-19 years): Review of current
literature and personal experience. Acta Biomed. 2023 Apr 24;94(2):e2023004. doi:
10.23750/abm.v94i2.14324. Review. PubMed [citation] PMID: 37092615, PMCID:
PMC10210567

22. Sá R, Ferraz L, Barros A, Sousa M. The Klinefelter Syndrome and Testicular Sperm
Retrieval Outcomes. Genes (Basel). 2023 Mar 4;14(3). pii: 647. doi:
10.3390/genes14030647. Review. PubMed [citation] PMID: 36980920,

PMCID:
PMC10048758

23. Liu Y, Zhu XZ, Xiao Y, Wu SY, Zuo WJ, Yu Q, Cao AY, Li JJ, Yu KD, Liu GY, Wu J,
Sun T, Cui JW, Lv Z, Li HP, Zhu XY, Jiang YZ, Wang ZH, Shao ZM. Subtyping-based
platform guides precision medicine for heavily pretreated metastatic
triple-negative breast cancer: The FUTURE phase II umbrella clinical trial. Cell
Res. 2023 May;33(5):389-402. doi: 10.1038/s41422-023-00795-2. Epub 2023 Mar 27.
PubMed [citation] PMID: 36973538, PMCID: PMC10156707

24. Wu FT, Chen CP, Chen SW, Chern SR, Chen PT, Chiu CL, Lee CC, Chen WL, Wang W.
Concomitance of 47,XXY, a balanced reciprocal translocation of t(4;17)(q12;q11.2)
encompassing SPINK2 at 4q12 and NOS at 17q11.2 and an AZFa sY86 deletion in an
infertile male. Taiwan J Obstet Gynecol. 2023 Mar;62(2):336-342. doi:
10.1016/j.tjog.2022.11.014. Review. PubMed [citation] PMID: 36965905

25. Izu Y, Birk DE. Collagen XII mediated cellular and extracellular mechanisms in
development, regeneration, and disease. Front Cell Dev Biol. 2023 Mar
2;11:1129000. doi: 10.3389/fcell.2023.1129000. eCollection 2023. Review. PubMed
[citation] PMID: 36936682, PMCID: PMC10017729

26. Vetter J. The Mushroom Glucans: Molecules of High Biological and Medicinal
Importance. Foods. 2023 Feb 27;12(5). pii: 1009. doi: 10.3390/foods12051009.
Review. PubMed [citation] PMID: 36900525, PMCID: PMC10000499

27. van Rijn S, Kuiper K, Bouw N, Urbanus E, Swaab H. Neurocognitive and behavioral
development in young children (1-7 years) with sex chromosome trisomy. Endocr
Connect. 2023 Apr 19;12(5). pii: e220494. doi: 10.1530/EC-22-0494. Print 2023 May
1. Review. PubMed [citation] PMID: 36880404, PMCID: PMC10160554

28. Debien V, De Caluwé A, Wang X, Piccart-Gebhart M, Tuohy VK, Romano E, Buisseret
L. Immunotherapy in breast cancer: an overview of current strategies and
perspectives. NPJ Breast Cancer. 2023 Feb 13;9(1):7. doi:
10.1038/s41523-023-00508-3. Review. PubMed [citation] PMID: 36781869,

PMCID:
PMC9925769

29. Wang X, Collet L, Rediti M, Debien V, De Caluwé A, Venet D, Romano E, Rothé F,
Sotiriou C, Buisseret L. Predictive Biomarkers for Response to Immunotherapy in
Triple Negative Breast Cancer: Promises and Challenges. J Clin Med. 2023 Jan
26;12(3). pii: 953. doi: 10.3390/jcm12030953. Review. PubMed [citation] PMID:
36769602, PMCID: PMC9917763

30. Ljubicic ML, Johannsen TH, Fischer MB, Upners EN, Busch AS, Main KM, Andersson
AM, Hagen CP, Juul A. Serum LH/FSH ratios in 87 infants with differences of sex
development. Endocr Connect. 2023 Feb 23;12(3). pii: e220275. doi:
10.1530/EC-22-0275. Print 2023 Mar 1. Review. PubMed [citation] PMID: 36696141,
PMCID: PMC9986384

31. Molyneux K, Beck-Esmay J, Koyfman A, Long B. High risk and low prevalence
diseases: Mesenteric ischemia. Am J Emerg Med. 2023 Mar;65:154-161. doi:
10.1016/j.ajem.2023.01.001. Epub 2023 Jan 4. Review. PubMed [citation] PMID:
36638612

32. Liu S, Chang Q, Yang F, Xu Y, Jia B, Wu R, Li L, Yin A, Chen W, Huang F, Yang X,
Li F. Non-invasive prenatal test findings in 41,819 pregnant women: results from
a clinical laboratory in southern China. Arch Gynecol Obstet. 2023
Sep;308(3):787-795. doi: 10.1007/s00404-022-06908-3. Epub 2023 Jan 5. Review.
PubMed [citation] PMID: 36602559

33. Gravholt CH, Ferlin A, Gromoll J, Juul A, Raznahan A, van Rijn S, Rogol AD,
Skakkebæk A, Tartaglia N, Swaab H. New developments and future trajectories in
supernumerary sex chromosome abnormalities: a summary of the 2022 3rd
International Workshop on Klinefelter Syndrome, Trisomy X, and XYY. Endocr
Connect. 2023 Feb 8;12(3). pii: e220500. doi: 10.1530/EC-22-0500. Print 2023 Mar
1. Review. PubMed [citation] PMID: 36598290, PMCID: PMC9986408

34. Shear MA, Swanson K, Garg R, Jelin AC, Boscardin J, Norton ME, Sparks TN. A systematic review and meta-analysis of cell-free DNA testing for detection of fetal sex chromosome aneuploidy. Prenat Diagn. 2023 Feb;43(2):133-143. doi: 10.1002/pd.6298. Epub 2023 Jan 8. Review. PubMed [citation] PMID: 36588186, PMCID: PMC10268789

35. Ferraguti G, Terracina S, Micangeli G, Lucarelli M, Tarani L, Ceccanti M, Spaziani M, D'Orazi V, Petrella C, Fiore M. NGF and BDNF in pediatrics syndromes. Neurosci Biobehav Rev. 2023 Feb;145:105015. doi: 10.1016/ j.neubiorev.2022.105015. Epub 2022 Dec 21. Review. PubMed [citation] PMID: 36563920

36. Røssevold AH, Andresen NK, Bjerre CA, Gilje B, Jakobsen EH, Raj SX, Falk RS, Russnes HG, Jahr T, Mathiesen RR, Lømo J, Garred Ø, Chauhan SK, Lereim RR, Dunn C, Naume B, Kyte JA. Atezolizumab plus anthracycline-based chemotherapy in metastatic triple-negative breast cancer: the randomized, double-blind phase 2b ALICE trial. Nat Med. 2022 Dec;28(12):2573-2583. doi: 10.1038/ s41591-022-02126-1. Epub 2022 Dec 8. PubMed [citation] PMID: 36482103, PMCID: PMC9800277

37. Nowotny HF, Reisch N. Challenges Waiting for an Adult with DSD. Horm Res Paediatr. 2023;96(2):207-221. doi: 10.1159/000527433. Epub 2022 Dec 6. Review. PubMed [citation] PMID: 36473446

38. Di Persio S, Neuhaus N. Human spermatogonial stem cells and their niche in male (in)fertility: novel concepts from single-cell RNA-sequencing. Hum Reprod. 2023 Jan 5;38(1):1-13. doi: 10.1093/humrep/deac245. Review. PubMed [citation] PMID: 36409992, PMCID: PMC9825264

39. Ershadi R, Vahedi M, Zadeh HK, Fathi F, Ebrahimpour H, Zadeh JS, Rafieian S. Subcutaneous octreotide therapy for malignant pleural effusion after pleurodesis with talc powder: a placebo-controlled, triple-blind, randomized trial. Support

Care Cancer. 2022 Dec;30(12):9833-9840. doi: 10.1007/
s00520-022-07440-5. Epub
2022 Nov 11. PubMed [citation] PMID: 36357795

40. Ricciardi G, Cammisa L, Bove R, Picchiotti G, Spaziani M, Isidori AM, Aceti F,
Giacchetti N, Romani M, Sogos C. Clinical, Cognitive and Neurodevelopmental
Profile in Tetrasomies and Pentasomies: A Systematic Review. Children (Basel).
2022 Nov 9;9(11). pii: 1719. doi: 10.3390/children9111719. Review. PubMed
[citation] PMID: 36360447, PMCID: PMC9688827

41. Bradshaw AW, Deebel NA, Xu MC, Kogan S, Atala A, Sadri-Ardekani H. Examining
potential mechanisms of testicular fibrosis in Klinefelter Syndrome: A review of
current understanding. Andrology. 2023 Mar;11(3):435-443. doi:
10.1111/andr.13327. Epub 2022 Oct 27. Review. PubMed [citation] PMID: 36252136

42. White M, Zacharin MR, Fawcett S, McGillivray G. Klinefelter Syndrome: What should
we tell prospective parents? Prenat Diagn. 2023 Feb;43(2):240-249. doi:
10.1002/pd.6250. Epub 2022 Oct 21. Review. PubMed [citation] PMID: 36225116

43. Sui J, Zheng L, Zheng XL. ADAMTS13 Biomarkers in Management of Immune Thrombotic
Thrombocytopenic Purpura. Arch Pathol Lab Med. 2023 Aug 1;147(8):974-979. doi:
10.5858/arpa.2022-0050-RA. Review. PubMed [citation] PMID: 36223210

44. Rajabzadeh M, Taheri N, Jazayeri O. 49,XXXXY syndrome: A case study and a
systematic review of clinical features among the Iranian population. Clin Case
Rep. 2022 Sep 24;10(9):e6342. doi: 10.1002/ccr3.6342. eCollection 2022 Sep.
PubMed [citation] PMID: 36188049, PMCID: PMC9508802

45. He M, Bao N, Qi RQ, Wu Y, Liu M. A randomized, prospective pilot study for
comparison of a triple combination of 2940 nm Er:YAG Laser and triamcinolone
acetonide solution with either 308 nm excimer laser or 0.1% tacrolimus in
treatment of stable segmental vitiligo. Dermatol Ther. 2022

Nov;35(11):e15875.
doi: 10.1111/dth.15875. Epub 2022 Oct 14. PubMed [citation] PMID:
36181292

46. Nicola AG, Carsote M, Gheorghe AM, Petrova E, Popescu AD, Staicu
AN, Ţuculină MJ,
Petcu C, Dascălu IT, Tircă T. Approach of Heterogeneous Spectrum
Involving
3beta-Hydroxysteroid Dehydrogenase 2 Deficiency. Diagnostics (Basel).
2022 Sep
7;12(9). pii: 2168. doi: 10.3390/diagnostics12092168. Review. PubMed
[citation]
PMID: 36140569, PMCID: PMC9497988

47. Mateos MV, Prosper F, Martin Sánchez J, Ocio EM, Oriol A, Motlló
C, Michot JM,
Jarque I, Iglesias R, Solé M, Martínez S, Kahatt C, Fudio S, Corral G,
Zeaiter A,
Montilla L, Ribrag V. Phase I study of plitidepsin in combination with
bortezomib
and dexamethasone in patients with relapsed/refractory multiple
myeloma. Cancer
Med. 2023 Feb;12(4):3999-4009. doi: 10.1002/cam4.5250. Epub 2022 Sep
20. PubMed
[citation] PMID: 36127823, PMCID: PMC9972151

48. Rashid MH. Full-length recombinant antibodies from Escherichia
coli: production,
characterization, effector function (Fc) engineering, and clinical
evaluation.
MAbs. 2022 Jan-Dec;14(1):2111748. doi: 10.1080/19420862.2022.2111748.
Review.
PubMed [citation] PMID: 36018829, PMCID: PMC9423848

49. Pach J, Regulski PA, Tomczyk J, Strużycka I. Clinical implications
of a diagnosis
of taurodontism: A literature review. Adv Clin Exp Med. 2022
Dec;31(12):1385-1389. doi: 10.17219/acem/152120. Review. PubMed
[citation] PMID:
36000881

50. Tian X, Zhao K, Teng A, Li Y, Wang W. A rethinking of collagen as
tough
biomaterials in meat packaging: assembly from native to synthetic.
Crit Rev Food
Sci Nutr. 2024;64(4):957-977. doi: 10.1080/10408398.2022.2111401. Epub
2022 Aug
23. Review. PubMed [citation] PMID: 35997287

51. Navon D. How do genetic tests answer questions about

neurodevelopmental
differences? A sociological take. Dev Med Child Neurol. 2022
Dec;64(12):1462-1469. doi: 10.1111/dmcn.15376. Epub 2022 Aug 13.
Review. PubMed
[citation] PMID: 35962997

52. Maxwell F, Savignac A, Bekdache O, Calvez S, Lebacle C, Arama E,
Garrouche N,
Rocher L. Leydig Cell Tumors of the Testis: An Update of the Imaging
Characteristics of a Not So Rare Lesion. Cancers (Basel). 2022 Jul
27;14(15).
pii: 3652. doi: 10.3390/cancers14153652. Review. PubMed [citation]
PMID:
35954321, PMCID: PMC9367522

53. Butler G, Srirangalingam U, Faithfull J, Sangster P, Senniappan S,
Mitchell R.
Klinefelter syndrome: going beyond the diagnosis. Arch Dis Child. 2023
Mar;108(3):166-171. doi: 10.1136/archdischild-2020-320831. Epub 2022
Aug 10.
Review. PubMed [citation] PMID: 35948402, PMCID: PMC7614197

54. Ekhtiari H, Soleimani G, Kuplicki R, Yeh HW, Cha YH, Paulus M.
Transcranial
direct current stimulation to modulate fMRI drug cue reactivity in
methamphetamine users: A randomized clinical trial. Hum Brain Mapp.
2022 Dec
1;43(17):5340-5357. doi: 10.1002/hbm.26007. Epub 2022 Aug 1. PubMed
[citation]
PMID: 35915567, PMCID: PMC9812244

55. Dirvanskyte P, Gurram B, Bolton C, Warner N, Jones KDJ, Griffin
HR; Genomics
England Research Consortium., Park JY, Keller KM, Gilmour KC,
Hambleton S, Muise
AM, Wysocki C, Uhlig HH. Chromosomal Numerical Aberrations and Rare
Copy Number
Variation in Patients with Inflammatory Bowel Disease. J Crohns
Colitis. 2023 Jan
27;17(1):49-60. doi: 10.1093/ecco-jcc/jjac103. PubMed [citation] PMID:
35907265,
PMCID: PMC9880952

56. Ionescu S, Nicolescu AC, Marincas M, Madge OL, Simion L. An Update
on the General
Features of Breast Cancer in Male Patients—A Literature Review.
Diagnostics
(Basel). 2022 Jun 26;12(7). pii: 1554. doi: 10.3390/
diagnostics12071554. Review.
PubMed [citation] PMID: 35885460, PMCID: PMC9323942

57. Cortes J, Rugo HS, Cescon DW, Im SA, Yusof MM, Gallardo C, Lipatov O, Barrios CH,
Perez-Garcia J, Iwata H, Masuda N, Torregroza Otero M, Gokmen E, Loi S, Guo Z,
Zhou X, Karantza V, Pan W, Schmid P; KEYNOTE-355 Investigators.. Pembrolizumab
plus Chemotherapy in Advanced Triple-Negative Breast Cancer. N Engl J Med. 2022
Jul 21;387(3):217-226. doi: 10.1056/NEJMoa2202809. PubMed [citation] PMID:
35857659

58. Luo F, Ye Q, Shen J. Systemic lupus erythematosus with trisomy X: a case report
and review of the literature. J Med Case Rep. 2022 Jul 19;16(1):281. doi:
10.1186/s13256-022-03478-5. Review. PubMed [citation] PMID: 35850774, PMCID:
PMC9295272

59. Dwyer AA, Héritier V, Llahana S, Edelman L, Papadakis GE, Vaucher L, Pitteloud N,
Hauschild M. Navigating Disrupted Puberty: Development and Evaluation of a
Mobile-Health Transition Passport for Klinefelter Syndrome. Front Endocrinol
(Lausanne). 2022 Jun 24;13:909830. doi: 10.3389/fendo.2022.909830. eCollection
2022. Erratum in: Front Endocrinol (Lausanne). 2023 Mar 15;14:1182233. PubMed
[citation] PMID: 35813640, PMCID: PMC9264386

60. Krsnik D, Marić T, Bulić-Jakuš F, Sinčić N, Bojanac AK. LIN28 Family in Testis:
Control of Cell Renewal, Maturation, Fertility and Aging. Int J Mol Sci. 2022 Jun
29;23(13). pii: 7245. doi: 10.3390/ijms23137245. Review. PubMed [citation] PMID:
35806250, PMCID: PMC9266904

61. Xue Y, Shamp T, Nagana Gowda GA, Crabtree M, Bagchi D, Raftery D. A Combination
of Nicotinamide and D-Ribose (RiaGev) Is Safe and Effective to Increase NAD(+)
Metabolome in Healthy Middle-Aged Adults: A Randomized, Triple-Blind,
Placebo-Controlled, Cross-Over Pilot Clinical Trial. Nutrients. 2022 May
26;14(11). pii: 2219. doi: 10.3390/nu14112219. PubMed [citation] PMID: 35684021,

PMCID: PMC9183138

62. Ackermans LLGC, Volmer L, Timmermans QMMA, Brecheisen R, Damink SMWO, Dekker A,
Loeffen D, Poeze M, Blokhuis TJ, Wee L, Ten Bosch JA. Clinical evaluation of
automated segmentation for body composition analysis on abdominal L3 CT slices in
polytrauma patients. Injury. 2022 Nov;53 Suppl 3:S30-S41. doi:
10.1016/j.injury.2022.05.004. Epub 2022 Jun 2. PubMed [citation] PMID: 35680433

63. Moreau P, Garfall AL, van de Donk NWCJ, Nahi H, San-Miguel JF, Oriol A, Nooka AK,
Martin T, Rosinol L, Chari A, Karlin L, Benboubker L, Mateos MV, Bahlis N, Popat
R, Besemer B, Martínez-López J, Sidana S, Delforge M, Pei L, Trancucci D, Verona
R, et al. Teclistamab in Relapsed or Refractory Multiple Myeloma. N Engl J Med.
2022 Aug 11;387(6):495-505. doi: 10.1056/NEJMoa2203478. Epub 2022 Jun 5. PubMed
[citation] PMID: 35661166, PMCID: PMC10587778

64. Mehmet B, Gillard S, Jayasena CN, Llahana S. Association between domains of
quality of life and patients with Klinefelter syndrome: a systematic review. Eur
J Endocrinol. 2022 Jun 24;187(2):S21-S34. doi: 10.1530/EJE-21-1239. Print 2022
Aug 1. PubMed [citation] PMID: 35639859, PMCID: PMC9254297

65. Hovnik T, Zitnik E, Avbelj Stefanija M, Bertok S, Sedej K, Bancic Silva V,
Battelino T, Groselj U. An Adolescent Boy with Klinefelter Syndrome and
47,XXY/46,XX Mosaicism: Case Report and Review of Literature. Genes (Basel). 2022
Apr 23;13(5). pii: 744. doi: 10.3390/genes13050744. Review. PubMed [citation]
PMID: 35627128, PMCID: PMC9141365

66. Mameli C, Fiore G, Sangiorgio A, Agostinelli M, Zichichi G, Zuccotti G, Verduci
E. Metabolic and Nutritional Aspects in Paediatric Patients with Klinefelter
Syndrome: A Narrative Review. Nutrients. 2022 May 18;14(10). pii: 2107. doi:
10.3390/nu14102107. Review. PubMed [citation] PMID: 35631248, PMCID: PMC9147015

67. Willemars MMA, Nabben M, Verdonschot JAJ, Hoes MF. Evaluation of the Interaction
of Sex Hormones and Cardiovascular Function and Health. Curr Heart Fail Rep. 2022
Aug;19(4):200-212. doi: 10.1007/s11897-022-00555-0. Epub 2022 May 28. Review.
PubMed [citation] PMID: 35624387, PMCID: PMC9329157

68. Tharakan T, Corona G, Foran D, Salonia A, Sofikitis N, Giwercman A, Krausz C, Yap
T, Jayasena CN, Minhas S. Does hormonal therapy improve sperm retrieval rates in
men with non-obstructive azoospermia: a systematic review and meta-analysis. Hum
Reprod Update. 2022 Aug 25;28(5):609-628. doi: 10.1093/humupd/dmac016. PubMed
[citation] PMID: 35526153, PMCID: PMC9434299

69. Nguyen J, Bashan KA, Jiang C, Lin M, Tootla Y, Udobi K, Williams KN, Gelbard R,
Nguyen CT, Sola R Jr, Smith RN, Sciarretta JD, Butler C, Morse BC, Grant AA, Rhee
P. Rectal Contrast CT Scans of Limited Utility in Diagnosing Colonic Injuries in
Penetrating Trauma: A Meta-Analysis. Am Surg. 2023
Jun;89(6):2284-2290. doi:
10.1177/00031348221086792. Epub 2022 Apr 18. PubMed [citation] PMID: 35437027

70. Wang Q, Li J. Intelligent Algorithm-Based Gastrointestinal X-Ray Examination in
Evaluating the Therapeutic Effect of Probiotics Combined with Triple Therapy on
Children with Helicobacter Infection. Contrast Media Mol Imaging. 2022 Mar
27;2022:8464361. doi: 10.1155/2022/8464361. eCollection 2022. PubMed [citation]
PMID: 35414799, PMCID: PMC8977303

71. Liang B, Cheung AS, Nolan BJ. Clinical features and prevalence of Klinefelter
syndrome in transgender individuals: A systematic review. Clin Endocrinol (Oxf).
2022 Jul;97(1):3-12. doi: 10.1111/cen.14734. Epub 2022 Apr 15. PubMed [citation]
PMID: 35394664, PMCID: PMC9540025

72. El Fekih S, Gueganic N, Tous C, Douet-Guilbert N, Blesson S, Morel F, Perrin A.

Meiotic Segregation of an Isodicentric Derived from Chromosome 15 in Sperm of a
Patient with Mosaic Karyotype: Case Report and Review of the Literature.
Cytogenet Genome Res. 2022;162(1-2):34-39. doi: 10.1159/000523916. Epub 2022 Apr
7. Review. PubMed [citation] PMID: 35390789

73. Picker J, Lan Z, Arora S, Green M, Hahn M, Cosgriff-Hernandez E, Hook M.
Prokaryotic Collagen-Like Proteins as Novel Biomaterials. Front Bioeng
Biotechnol. 2022 Mar 17;10:840939. doi: 10.3389/fbioe.2022.840939. eCollection
2022. Review. PubMed [citation] PMID: 35372322, PMCID: PMC8968730

74. Gaudino R, De Filippo G, Bozzola E, Gasparri M, Bozzola M, Villani A, Radetti G.
Current clinical management of constitutional delay of growth and puberty. Ital J
Pediatr. 2022 Mar 24;48(1):45. doi: 10.1186/s13052-022-01242-5. Review. PubMed
[citation] PMID: 35331309, PMCID: PMC8944060

75. Ye F, Bian L, Wen J, Yu P, Li N, Xie X, Wang X. Additional capecitabine use in
early-stage triple negative breast cancer patients receiving standard
chemotherapy: a new era? A meta-analysis of randomized controlled trials. BMC
Cancer. 2022 Mar 12;22(1):261. doi: 10.1186/s12885-022-09326-5. PubMed [citation]
PMID: 35279130, PMCID: PMC8917675

76. Matuschek C, Jazmati D, Bölke E, Tamaskovics B, Corradini S, Budach W, Krug D,
Mohrmann S, Ruckhäberle E, Fehm T, Nestle Krämling C, Dommach M, Haussmann J.
Post-Neoadjuvant Treatment Strategies in Breast Cancer. Cancers (Basel). 2022 Feb
28;14(5). pii: 1246. doi: 10.3390/cancers14051246. Review. PubMed [citation]
PMID: 35267554, PMCID: PMC8909560

77. Cacovich S, Dally P, Vidon G, Legrand M, Gbegnon S, Rousset J, Puel JB,
Guillemoles JF, Schulz P, Bouttemy M, Etcheberry A. In-Depth Chemical and
Optoelectronic Analysis of Triple-Cation Perovskite Thin Films by Combining XPS
Profiling and PL Imaging. ACS Appl Mater Interfaces. 2022 Aug
3;14(30):34228-34237. doi: 10.1021/acsami.1c22286. Epub 2022 Mar 4.

Review.
PubMed [citation] PMID: 35245028

78. Bateman JF, Shoulders MD, Lamandé SR. Collagen misfolding mutations: the
contribution of the unfolded protein response to the molecular pathology. Connect
Tissue Res. 2022 May;63(3):210-227. doi: 10.1080/03008207.2022.2036735. Epub 2022
Feb 26. Review. PubMed [citation] PMID: 35225118, PMCID: PMC8977234

79. Erro R, Reich SG. Rare tremors and tremors occurring in other neurological
disorders. J Neurol Sci. 2022 Apr 15;435:120200. doi: 10.1016/j.jns.2022.120200.
Epub 2022 Feb 19. Review. PubMed [citation] PMID: 35220114

80. Thirumalai A, Anawalt BD. Epidemiology of Male Hypogonadism. Endocrinol Metab
Clin North Am. 2022 Mar;51(1):1-27. doi: 10.1016/j.ecl.2021.11.016. Epub 2022 Feb
8. Review. PubMed [citation] PMID: 35216709, PMCID: PMC9136962

81. Wren G, Davies W. Sex-linked genetic mechanisms and atrial fibrillation risk. Eur
J Med Genet. 2022 Apr;65(4):104459. doi: 10.1016/j.ejmg.2022.104459. Epub 2022
Feb 19. Review. PubMed [citation] PMID: 35189376

82. Köhn FM, Schuppe HC, Kliesch S. [Desire for children among patients with
Klinefelter Syndrome]. MMW Fortschr Med. 2022 Feb;164(Suppl 4):36-37. doi:
10.1007/s15006-022-0674-9. Review. German. No abstract available. PubMed
[citation] PMID: 35146716

83. Zitzmann M. [Klinefelter Syndrome and associated disorders]. MMW Fortschr Med.
2022 Feb;164(Suppl 4):32-34. doi: 10.1007/s15006-022-0673-x. Review. German. No
abstract available. PubMed [citation] PMID: 35146715

84. Mallefet P, Armogida M, Doll WJ, Page RC, Sandefer EP. A single-dose, open-label,
randomized, scintigraphic study to investigate the gastrointestinal behavior of 2
triple-combination cold products (acetaminophen, phenylephrine, and
dextromethorphan) in healthy male volunteers. Trials. 2022 Feb 8;23(1):128. doi:

10.1186/s13063-022-06037-x. PubMed [citation] PMID: 35135601, PMCID: PMC8822665

85. Robles-González L, Ramírez Maldonado M, Alcalá-Escamilla JC, Jurado-Fasoli L, Miras-Moreno S, Soriano MA, García-Ramos A, Ruiz JR, Amaro-Gahete FJ. Caffeine ingestion attenuates diurnal variation of lower-body ballistic performance in resistance-trained women. Eur J Sport Sci. 2023 Mar;23(3):381-392. doi: 10.1080/17461391.2022.2038274. Epub 2022 Mar 3. PubMed [citation] PMID: 35109780

86. Zou C, Yu D, Geng H, Lan X, Sun W. A patient with 47, XYY mosaic karyotype and congenital absence of bilateral vas deferens: a case report and literature review. BMC Urol. 2022 Feb 2;22(1):16. doi: 10.1186/ s12894-022-00965-1. Review. PubMed [citation] PMID: 35109852, PMCID: PMC8809031

87. Siniscalchi C, Di Palo A, Russo A, Potenza N. The lncRNAs at X Chromosome Inactivation Center: Not Just a Matter of Sex Dosage Compensation. Int J Mol Sci. 2022 Jan 6;23(2). pii: 611. doi: 10.3390/ijms23020611. Review. PubMed [citation] PMID: 35054794, PMCID: PMC8775829

88. Rey RA. Recent advancement in the treatment of boys and adolescents with hypogonadism. Ther Adv Endocrinol Metab. 2022 Jan 5;13:20420188211065660. doi: 10.1177/20420188211065660. eCollection 2022. Review. PubMed [citation] PMID: 35035874, PMCID: PMC8753232

89. Joensuu H, Kellokumpu-Lehtinen PL, Huovinen R, Jukkola A, Tanner M, Ahlgren J, Auvinen P, Lahdenperä O, Villman K, Nyandoto P, Nilsson G, Poikonen-Saksela P, Kataja V, Bono P, Junnila J, Lindman H. Adjuvant Capecitabine for Early Breast Cancer: 15-Year Overall Survival Results From a Randomized Trial. J Clin Oncol. 2022 Apr 1;40(10):1051-1058. doi: 10.1200/JCO.21.02054. Epub 2022 Jan 12. PubMed [citation] PMID: 35020465, PMCID: PMC8966968

90. Yang XW, Wang J, Nai Z, Yang YJ. [Klinefelter syndrome: Advances in research].
Zhonghua Nan Ke Xue. 2021 Mar;27(3):269-273. Review. Chinese. PubMed [citation]
PMID: 34914311

91. Li L, Shi Y, Zhao N, Liu Z, Zhao Z, Song Z, Zheng S, Yan M, Leng Z, Chen S, Shang
G, Kou H, Liu H. A patient with Turner syndrome received the percutaneous vertebroplasty seven times: a case report and literature review. Eur
J Med Res. 2021 Dec 7;26(1):139. doi: 10.1186/s40001-021-00617-4. Review. PubMed
[citation] PMID: 34876225, PMCID: PMC8650291

92. Swanson K, Bishop JC, Al-Kouatly HB, Makhamreh M, Felton T, Vora NL, Sparks TN,
Jelin AC. Prenatal phenotype of 47, XXY (Klinefelter syndrome). Prenat Diagn.
2023 Feb;43(2):207-212. doi: 10.1002/pd.6071. Epub 2021 Dec 7. Review. PubMed
[citation] PMID: 34874073, PMCID: PMC9170827

93. Liu S, Yuan T, Song S, Chen S, Wang L, Fu Y, Dong Y, Tang Y, Zhao W. Glucose
metabolic disorder in Klinefelter syndrome: a retrospective analysis in a single
Chinese hospital and literature review. BMC Endocr Disord. 2021 Dec 1;21(1):239.
doi: 10.1186/s12902-021-00893-5. Review. PubMed [citation] PMID: 34852815, PMCID:
PMC8638221

94. Stadler JA 3rd. Neurosurgical Evaluation and Management of Patients with
Chromosomal Abnormalities. Neurosurg Clin N Am. 2022 Jan;33(1):61-65. doi:
10.1016/j.nec.2021.09.012. Epub 2021 Oct 27. Review. PubMed [citation] PMID:
34801142

95. Laan M, Kasak L, Punab M. Translational aspects of novel findings in genetics of
male infertility-status quo 2021. Br Med Bull. 2021 Dec 16;140(1):5-22. doi:
10.1093/bmb/ldab025. Review. PubMed [citation] PMID: 34755838, PMCID: PMC8677437

96. Li S, Myerson MS. Management of Complex Tarsal Coalition in Children. Foot Ankle

Clin. 2021 Dec;26(4):941-954. doi: 10.1016/j.fcl.2021.07.012. Epub 2021 Aug 30.
Review. PubMed [citation] PMID: 34752245

97. Majzoub A, Arafa M, Clemens H, Imperial J, Leisegang K, Khalafalla K, Agarwal A,
Henkel R, Elbardisi H. A systemic review and meta-analysis exploring the
predictors of sperm retrieval in patients with non-obstructive azoospermia and
chromosomal abnormalities. Andrologia. 2022 Apr;54(3):e14303. doi:
10.1111/and.14303. Epub 2021 Nov 2. Review. PubMed [citation] PMID: 34729809

98. Vila FD, Kas JJ, Rehr JJ, Kowalski K, Peng B. Equation-of-Motion Coupled-Cluster
Cumulant Green's Function for Excited States and X-Ray Spectra. Front Chem. 2021
Sep 22;9:734945. doi: 10.3389/fchem.2021.734945. eCollection 2021. Review. PubMed
[citation] PMID: 34631660, PMCID: PMC8493088

99. Gonzalez-Latapi P, Sousa M, Lang AE. Movement Disorders Associated with
Hypogonadism. Mov Disord Clin Pract. 2021 Jul 29;8(7):997-1011. doi:
10.1002/mdc3.13308. eCollection 2021 Oct. Review. PubMed [citation] PMID:
34631935, PMCID: PMC8485629

100. Mitrovic IZ, Almalki S, Tekin SB, Sedghi N, Chalker PR, Hall S. Oxides for
Rectenna Technology. Materials (Basel). 2021 Sep 10;14(18). pii: 5218. doi:
10.3390/ma14185218. Review. PubMed [citation] PMID: 34576441, PMCID: PMC8471330

101. Cueto-Robledo G, Jurado-Hernandez MY, Camacho-Delgado FR, Roldan-Valadez E,
Heredia-Arroyo AL, Cueto-Romero HD, Palafox LEG, Anaya RO, Dircio AR, Vazquez HM,
Mateo PA. Pulmonary Thromboendarterectomy in Klinefelter Syndrome. Literature
Review. Curr Probl Cardiol. 2022 Dec;47(12):101003. doi:
10.1016/j.cpcardiol.2021.101003. Epub 2021 Sep 24. Review. PubMed [citation]
PMID: 34571108

102. Punjani N, Kang C, Lamb DJ, Schlegel PN. Current updates and future perspectives
in the evaluation of azoospermia: A systematic review. Arab J Urol.

2021 Jul
22;19(3):206-214. doi: 10.1080/2090598X.2021.1954415. eCollection
2021. PubMed
[citation] PMID: 34552771, PMCID: PMC8451618

103. Hou N, Wu J, Xiao J, Wang Z, Song Z, Ke Z, Wang R, Wei M, Xu M,
Wei J, Qian X, Xu
X, Yi J, Wang T, Zhang J, Li N, Fan J, Hou G, Wang Y, Wang Z, Ling R.
Development, verification, and comparison of a risk stratification
model
integrating residual cancer burden to predict individual prognosis in
early-stage
breast cancer treated with neoadjuvant therapy. ESMO Open. 2021
Oct;6(5):100269.
doi: 10.1016/j.esmoop.2021.100269. Epub 2021 Sep 16. PubMed [citation]
PMID:
34537675, PMCID: PMC8455687

104. Houston BJ, Riera-Escamilla A, Wyrwoll MJ, Salas-Huetos A, Xavier
MJ, Nagirnaja
L, Friedrich C, Conrad DF, Aston KI, Krausz C, Tüttelmann F, O'Bryan
MK, Veltman
JA, Oud MS. A systematic review of the validated monogenic causes of
human male
infertility: 2020 update and a discussion of emerging gene-disease
relationships.
Hum Reprod Update. 2021 Dec 21;28(1):15-29. doi: 10.1093/humupd/
dmab030. PubMed
[citation] PMID: 34498060, PMCID: PMC8730311

105. Farshbaf-Khalili A, Farajnia S, Pourzeinali S, Shakouri SK,
Salehi-Pourmehr H.
The effect of nanomicelle curcumin supplementation and Nigella sativa
oil on the
expression level of miRNA-21, miRNA-422a, and miRNA-503 gene in
postmenopausal
women with low bone mass density: A randomized, triple-blind, placebo-
controlled
clinical trial with factorial design. Phytother Res. 2021
Nov;35(11):6216-6227.
doi: 10.1002/ptr.7259. Epub 2021 Sep 8. PubMed [citation] PMID:
34496087

106. Crowley C, Connor OJO, Ciet P, Tiddens HAWM, Maher MM. The
evolving role of
radiological imaging in cystic fibrosis. Curr Opin Pulm Med. 2021 Nov
1;27(6):575-585. doi: 10.1097/MCP.0000000000000828. Review. PubMed
[citation]
PMID: 34482339

107. Barry PJ, Mall MA, Álvarez A, Colombo C, de Winter-de Groot KM, Fajac I,
McBennett KA, McKone EF, Ramsey BW, Sutharsan S, Taylor-Cousar JL, Tullis E,
Ahluwalia N, Jun LS, Moskowitz SM, Prieto-Centurion V, Tian S, Waltz D, Xuan F,
Zhang Y, Rowe SM, Polineni D; et al. Triple Therapy for Cystic Fibrosis
Phe508del-Gating and -Residual Function Genotypes. N Engl J Med. 2021 Aug
26;385(9):815-825. doi: 10.1056/NEJMoa2100665. PubMed [citation] PMID: 34437784,
PMCID: PMC8982185

108. Weng HY, Lin TY, Lin YM, Cheng YS. The fertility preservation decision-making and
testicular sperm retrieval outcome in older adolescents with nonmosaic
Klinefelter syndrome and azoospermia. J Chin Med Assoc. 2021 Nov
1;84(11):1023-1027. doi: 10.1097/JCMA.0000000000000603. Review. PubMed [citation]
PMID: 34393187

109. Billa E, Kanakis GA, Goulis DG. Endocrine Follow-Up of Men with Non-Obstructive
Azoospermia Following Testicular Sperm Extraction. J Clin Med. 2021 Jul
28;10(15). pii: 3323. doi: 10.3390/jcm10153323. Review. PubMed [citation] PMID:
34362107, PMCID: PMC8347935

110. Pook CJ, Cocca A, Grandone A, Al-Hussini M, Lam W. The Evidence for Fertility
Preservation in Pediatric Klinefelter Syndrome. Front Reprod Health. 2021 Aug
3;3:629179. doi: 10.3389/frph.2021.629179. eCollection 2021. Review. PubMed
[citation] PMID: 36304035, PMCID: PMC9580826

111. Vatzia K, Fanariotis M, Makridis KG, Vlychou M, Fezoulidis IV, Vassiou K.
Frequency of sternal variations and anomalies in living individuals evaluated by
MDCT. Eur J Radiol. 2021 Sep;142:109828. doi: 10.1016/j.ejrad.2021.109828. Epub
2021 Jun 24. Review. PubMed [citation] PMID: 34280594

112. Inuzuka LM, Guerra-Peixe M, Macedo-Souza LI, Pedreira CC, Gurgel-Giannetti J,
Monteiro FP, Ramos L, Costa LA, Crippa ACS, Lourenco CM, Pachito DV,
Sukys-Claudino L, Gaspar LS, Antoniuk SA, Dutra LPS, Diniz SSL, Pires

RB, Garzon
E, Kok F. MECP2-related conditions in males: A systematic literature review and 8
additional cases. Eur J Paediatr Neurol. 2021 Sep;34:7-13. doi:
10.1016/j.ejpn.2021.05.013. Epub 2021 Jun 21. PubMed [citation] PMID:
34271245

113. Skakkebæk A, Wallentin M, Gravholt CH. Klinefelter syndrome or testicular
dysgenesis: Genetics, endocrinology, and neuropsychology. Handb Clin Neurol.
2021;181:445-462. doi: 10.1016/B978-0-12-820683-6.00032-4. Review. PubMed
[citation] PMID: 34238477

114. Dieamant F, Petersen CG, Vagnini LD, Renzi A, Petersen B, Massaro F, Zamara C,
Nicoletti A, Ricci J, Oliani AH, Oliveira JBA, Franco JG Jr. Impact of
Intracytoplasmic Morphologically Selected Sperm Injection (IMSI) on Birth
Defects: A Systematic Review and Meta-Analysis. JBRA Assist Reprod. 2021 Jul
21;25(3):466-472. doi: 10.5935/1518-0557.20210030. PubMed [citation] PMID:
34224237, PMCID: PMC8312289

115. Foldi J, Rozenblit M, Park TS, Knowlton CA, Golshan M, Moran M, Pusztai L.
Optimal Management for Residual Disease Following Neoadjuvant Systemic Therapy.
Curr Treat Options Oncol. 2021 Jul 2;22(9):79. doi: 10.1007/s11864-021-00879-4.
Review. PubMed [citation] PMID: 34213636

116. van den Berge M, De Backer J, Van Holsbeke C, De Backer W, Trivedi R, Jenkins M,
Dorinsky P, Aurivillius M. Functional respiratory imaging assessment of
budesonide/glycopyrrolate/formoterol fumarate and glycopyrrolate/formoterol
fumarate metered dose inhalers in patients with COPD: the value of inhaled
corticosteroids. Respir Res. 2021 Jul 1;22(1):191. doi:
10.1186/s12931-021-01772-2. PubMed [citation] PMID: 34210340, PMCID:
PMC8247252

117. Alexander E, Katharina T, Verena F, Jürgen G, Maximilian N, Calvin K, Andreas S,
Wolfgang S, Harald H, Dominik R. Comparison of different mechanical chest

compression devices in the alpine rescue setting: a randomized triple crossover
experiment. Scand J Trauma Resusc Emerg Med. 2021 Jun 29;29(1):84. doi:
10.1186/s13049-021-00899-x. PubMed [citation] PMID: 34187532, PMCID: PMC8244219

118. Coudray A, Choquet O, Swisser F, Hochman M, Bringuier S, Capdevila X. Combination
of real-time needle-tip pressure sensing and minimal intensity stimulation limits
unintentional intraneural injection during an ultrasound-guided peripheral nerve
block procedure: A randomized, parallel group, controlled trial. J Clin Anesth.
2021 Nov;74:110420. doi: 10.1016/j.jclinane.2021.110420. Epub 2021 Jun 23. PubMed
[citation] PMID: 34171709

119. Ly A, Sermondade N, Brioude F, Berthaut I, Bachelot A, Hamid RH, Khattabi LE,
Prades M, Lévy R, Dupont C. Fertility preservation in young men with Klinefelter
syndrome: A systematic review. J Gynecol Obstet Hum Reprod. 2021
Nov;50(9):102177. doi: 10.1016/j.jogoh.2021.102177. Epub 2021 Jun 1. PubMed
[citation] PMID: 34087451

120. Zheng XL. The standard of care for immune thrombotic thrombocytopenic purpura
today. J Thromb Haemost. 2021 Aug;19(8):1864-1871. doi: 10.1111/jth.15406. Epub
2021 Jun 30. Review. PubMed [citation] PMID: 34060225, PMCID: PMC8324529

121. El-Zaatari ZM, Ro JY. Mediastinal Germ Cell Tumors: A Review and Update on
Pathologic, Clinical, and Molecular Features. Adv Anat Pathol. 2021 Sep
1;28(5):335-350. doi: 10.1097/PAP.0000000000000304. Review. PubMed [citation]
PMID: 34029275

122. Barbonetti A, D'Andrea S, Vena W, Pizzocaro A, Rastrelli G, Pallotti F,
Condorelli R, Calogero AE, Pasquali D, Ferlin A, Foresta C, Jannini EA, Maggi M,
Lenzi A, Pivonello R, Isidori A, Garolla A, Francavilla S, Corona G; KING,
Klinefelter ItaliaN Group.. Erectile Dysfunction and Decreased Libido

in
Klinefelter Syndrome: A Prevalence Meta-Analysis and Meta-Regression Study. J Sex
Med. 2021 Jun;18(6):1053-1064. doi: 10.1016/j.jsxm.2021.03.078. Epub 2021 May 20.
Review. PubMed [citation] PMID: 34023236

123. Amer M, Vaccalluzzo L, Vena W, Mazziotti G, Morenghi E, Pizzocaro A. Oncological
diseases in Klinefelter Syndrome: an overview. Minerva Endocrinol (Torino). 2023
Mar;48(1):106-114. doi: 10.23736/S2724-6507.21.03440-0. Epub 2021 May 20. Review.
PubMed [citation] PMID: 34014064

124. Shiraishi K. Genome medicine in male infertility: From karyotyping to single-cell
analysis. J Obstet Gynaecol Res. 2021 Aug;47(8):2586-2596. doi:
10.1111/jog.14828. Epub 2021 May 16. Review. PubMed [citation] PMID: 33998107

125. Gastaldelli A, Stefan N, Häring HU. Liver-targeting drugs and their effect on
blood glucose and hepatic lipids. Diabetologia. 2021 Jul;64(7):1461-1479. doi:
10.1007/s00125-021-05442-2. Epub 2021 Apr 20. Review. PubMed [citation] PMID:
33877366, PMCID: PMC8187191

126. Shepherd S, Oates R. At what age should we attempt to retrieve sperm from males
with Klinefelter syndrome. Transl Androl Urol. 2021 Mar;10(3):1432-1441. doi:
10.21037/tau-19-858. Review. PubMed [citation] PMID: 33850778, PMCID: PMC8039581

127. Witherspoon L, Dergham A, Flannigan R. Y-microdeletions: a review of the genetic
basis for this common cause of male infertility. Transl Androl Urol. 2021
Mar;10(3):1383-1390. doi: 10.21037/tau-19-599. Review. PubMed [citation] PMID:
33850774, PMCID: PMC8039600

128. Pelzman DL, Hwang K. Genetic testing for men with infertility: techniques and
indications. Transl Androl Urol. 2021 Mar;10(3):1354-1364. doi:
10.21037/tau-19-725. Review. PubMed [citation] PMID: 33850771, PMCID: PMC8039607

129. Stormont GD, Deibert CM. Genetic causes and management of male infertility.
Transl Androl Urol. 2021 Mar;10(3):1365-1372. doi: 10.21037/tau.2020.03.34.
Review. PubMed [citation] PMID: 33850772, PMCID: PMC8039619

130. Cioppi F, Rosta V, Krausz C. Genetics of Azoospermia. Int J Mol Sci. 2021 Mar
23;22(6). pii: 3264. doi: 10.3390/ijms22063264. Review. PubMed [citation] PMID:
33806855, PMCID: PMC8004677

131. Soukkhaphone B, Lindsay C, Langlois S, Little J, Rousseau F, Reinharz D.
Non-invasive prenatal testing for the prenatal screening of sex chromosome
aneuploidies: A systematic review and meta-analysis of diagnostic test accuracy
studies. Mol Genet Genomic Med. 2021 May;9(5):e1654. doi: 10.1002/mgg3.1654. Epub
2021 Mar 23. PubMed [citation] PMID: 33755350, PMCID: PMC8172189

132. Nassau DE, Best JC, Cohen J, Gonzalez DC, Alam A, Ramasamy R. Androgenization in
Klinefelter syndrome: Clinical spectrum from infancy through young adulthood. J
Pediatr Urol. 2021 Jun;17(3):346-352. doi: 10.1016/j.jpurol.2021.02.021. Epub
2021 Feb 24. Review. PubMed [citation] PMID: 33726973

133. Khan NAJ, Tirona M. An updated review of epidemiology, risk factors, and
management of male breast cancer. Med Oncol. 2021 Mar 15;38(4):39. doi:
10.1007/s12032-021-01486-x. Review. PubMed [citation] PMID: 33721121

134. Majidpoor J, Mortezaee K. The efficacy of PD-1/PD-L1 blockade in cold cancers and
future perspectives. Clin Immunol. 2021 May;226:108707. doi:
10.1016/j.clim.2021.108707. Epub 2021 Mar 1. Review. PubMed [citation] PMID:
33662590

135. Chopra S, Fornito A, Francey SM, O'Donoghue B, Cropley V, Nelson B, Graham J,
Baldwin L, Tahtalian S, Yuen HP, Allott K, Alvarez-Jimenez M, Harrigan S,
Sabaroedin K, Pantelis C, Wood SJ, McGorry P. Differentiating the effect of
antipsychotic medication and illness on brain volume reductions in

first-episode
psychosis: A Longitudinal, Randomised, Triple-blind, Placebo-controlled MRI
Study. Neuropsychopharmacology. 2021 Jul;46(8):1494-1501. doi:
10.1038/s41386-021-00980-0. Epub 2021 Feb 26. PubMed [citation] PMID: 33637835,
PMCID: PMC8209146

136. Huang R, Lin J, Zhou J, Fan E, Zhang X, Chen R, Wu F, Li L. Hierarchical
Triple-Shelled MnCo(2) O(4) Hollow Microspheres as High-Performance Anode
Materials for Potassium-Ion Batteries. Small. 2021 Mar;17(11):e2007597. doi:
10.1002/smll.202007597. Epub 2021 Feb 23. Review. PubMed [citation] PMID:
33619897

137. Davies W. The contribution of Xp22.31 gene dosage to Turner and Klinefelter
syndromes and sex-biased phenotypes. Eur J Med Genet. 2021 Apr;64(4):104169. doi:
10.1016/j.ejmg.2021.104169. Epub 2021 Feb 19. Review. PubMed [citation] PMID:
33610733

138. Karani R, Sung JM, Xie L, Arada RB, Jefferson FA, Parkhomenko E, Lama DJ, Lee S,
Houshyar R, Lall C, Okhunov Z, Jiang P, Tapiero S, Patel RM, Clayman RV, Landman
J. Comparison of Conventional and Triple Bolus Computerized Tomographic Urography
Protocols for Radiation Dose Reduction in Hematuria Evaluation: A Randomized
Controlled Trial. J Urol. 2021 Jun;205(6):1740-1747. doi:
10.1097/JU.0000000000001603. Epub 2021 Feb 19. PubMed [citation] PMID:
33605796

139. Kailash Y, Raheem AA, Homa ST. How Successful Is Surgical Sperm Retrieval in
Klinefelter Syndrome? Front Reprod Health. 2021 Feb 18;3:636629. doi:
10.3389/frph.2021.636629. eCollection 2021. Review. PubMed [citation] PMID:
36303963, PMCID: PMC9580639

140. Raheem AA, Zaghloul AS, Sadek AMG, Rayes B, Abdel-Raheem TM. The Impact and
Management of Gynaecomastia in Klinefelter Syndrome. Front Reprod Health. 2021
Feb 12;3:629673. doi: 10.3389/frph.2021.629673. eCollection 2021.

Review. PubMed
[citation] PMID: 36303983, PMCID: PMC9580767

141. Manabe N, Yamaguchi Y. 3D Structural Insights into β-Glucans and Their Binding
Proteins. Int J Mol Sci. 2021 Feb 4;22(4). pii: 1578. doi: 10.3390/ijms22041578.
Review. PubMed [citation] PMID: 33557270, PMCID: PMC7915573

142. Casto C, Pepe G, Li Pomi A, Corica D, Aversa T, Wasniewska M. Hashimoto's
Thyroiditis and Graves' Disease in Genetic Syndromes in Pediatric Age. Genes
(Basel). 2021 Feb 4;12(2). pii: 222. doi: 10.3390/genes12020222. Review. PubMed
[citation] PMID: 33557156, PMCID: PMC7913917

143. Xiao Y, Gu Y, Qin L, Chen L, Chen X, Cui W, Li F, Xiang N, He X. Injectable
thermosensitive hydrogel-based drug delivery system for local cancer therapy.
Colloids Surf B Biointerfaces. 2021 Apr;200:111581. doi:
10.1016/j.colsurfb.2021.111581. Epub 2021 Jan 16. Review. PubMed [citation] PMID:
33524696

144. Vorontsova MV, Kalinchenko NY. [Klinefelter syndrome: literature review on using
modern methods of assisted reproductive technologies]. Probl Endokrinol (Mosk).
2020 Dec 30;66(6):31-38. doi: 10.14341/probl12666. Review. Russian. PubMed
[citation] PMID: 33481365

145. Ghanami Gashti N, Sadighi Gilani MA, Abbasi M. Sertoli cell-only syndrome:
etiology and clinical management. J Assist Reprod Genet. 2021 Mar;38(3):559-572.
doi: 10.1007/s10815-021-02063-x. Epub 2021 Jan 11. Review. PubMed [citation]
PMID: 33428073, PMCID: PMC7910341

146. Thakker S, Persily J, Najari BB. Kallman syndrome and central non-obstructive
azoospermia. Best Pract Res Clin Endocrinol Metab. 2020 Dec;34(6):101475. doi:
10.1016/j.beem.2020.101475. Epub 2020 Dec 1. Review. PubMed [citation] PMID:
33419659

147. Goulart LC, Ferreira-Filho LA, da Silva MM, Carneiro ISB, Carneiro SS,
Vilela-Filho O. Tectocerebellar dysraphia and occipital encephalocele associated
with trisomy X: case report and review of the literature. Childs Nerv Syst. 2021
Oct;37(10):3257-3260. doi: 10.1007/s00381-020-04989-6. Epub 2021 Jan 6. Review.
PubMed [citation] PMID: 33404715

148. Peña VN, Kohn TP, Herati AS. Genetic mutations contributing to non-obstructive
azoospermia. Best Pract Res Clin Endocrinol Metab. 2020 Dec;34(6):101479. doi:
10.1016/j.beem.2020.101479. Epub 2020 Dec 15. Review. PubMed [citation] PMID:
33390350

149. Deebel NA, Bradshaw AW, Sadri-Ardekani H. Infertility considerations in
klinefelter syndrome: From origin to management. Best Pract Res Clin Endocrinol
Metab. 2020 Dec;34(6):101480. doi: 10.1016/j.beem.2020.101480. Epub 2020 Dec 15.
Review. PubMed [citation] PMID: 33358481

150. Nozu K, Takaoka Y, Kai H, Takasato M, Yabuuchi K, Yamamura T, Horinouchi T,
Sakakibara N, Ninchoji T, Nagano C, Iijima K. Genetic background, recent advances
in molecular biology, and development of novel therapy in Alport syndrome. Kidney
Res Clin Pract. 2020 Dec 31;39(4):402-413. doi: 10.23876/j.krcp.20.111. Review.
PubMed [citation] PMID: 33214343, PMCID: PMC7771000

151. Singer A, Grinshpun-Cohen J, Sagi-Dain L. Colchicine treatment increases the risk
for fetal chromosomal aberrations-an observational study and systematic
literature review. Rheumatology (Oxford). 2021 May 14;60(5):2342-2347. doi:
10.1093/rheumatology/keaa602. PubMed [citation] PMID: 33179053

152. Acién P, Acién M. Disorders of Sex Development: Classification, Review, and
Impact on Fertility. J Clin Med. 2020 Nov 4;9(11). pii: E3555. doi:
10.3390/jcm9113555. Review. PubMed [citation] PMID: 33158283, PMCID: PMC7694247

153. Sesti F, Pofi R, Pozza C, Minnetti M, Gianfrilli D, Kanakis GA. Cardiovascular
Complications in Patients with Klinefelter's Syndrome. Curr Pharm Des.
2020;26(43):5556-5563. doi: 10.2174/1381612826666201102105408. Review. PubMed
[citation] PMID: 33138758

154. Aitken RJ, Baker MA. The Role of Genetics and Oxidative Stress in the Etiology of
Male Infertility-A Unifying Hypothesis? Front Endocrinol (Lausanne). 2020 Sep
30;11:581838. doi: 10.3389/fendo.2020.581838. eCollection 2020. Review. PubMed
[citation] PMID: 33101214, PMCID: PMC7554587

155. Ozkan E, Lacerda MP. Genetics, Cytogenetic Testing And Conventional Karyotype.
2023 Aug 8. StatPearls [Internet]. Treasure Island (FL): StatPearls Publishing;
2024 Jan-. PubMed [citation] PMID: 33085440

156. Kang J, Mashaal H, Anjum F. Mediastinal Germ Cell Tumors. 2023 Apr 27. StatPearls
[Internet]. Treasure Island (FL): StatPearls Publishing; 2024 Jan-. PubMed
[citation] PMID: 33085379

157. Kuroda S, Usui K, Sanjo H, Takeshima T, Kawahara T, Uemura H, Yumura Y. Genetic
disorders and male infertility. Reprod Med Biol. 2020 Jun 27;19(4):314-322. doi:
10.1002/rmb2.12336. eCollection 2020 Oct. Review. PubMed [citation] PMID:
33071633, PMCID: PMC7542010

158. An J, Derington CG, Luong T, Olson KL, King JB, Bress AP, Jackevicius CA.
Fixed-Dose Combination Medications for Treating Hypertension: A Review of
Effectiveness, Safety, and Challenges. Curr Hypertens Rep. 2020 Oct 14;22(11):95.
doi: 10.1007/s11906-020-01109-2. Review. PubMed [citation] PMID: 33052522

159. Liu Z, Jiao Y, He L, Wang H, Wang D. A rare case report of immunoglobulin
G4-related sclerosing mesenteritis and review of the literature. Medicine
(Baltimore). 2020 Oct 9;99(41):e22579. doi: 10.1097/
MD.0000000000022579. Review.

PubMed [citation] PMID: 33031306, PMCID: PMC7544369

160. Josso N, Rey RA. What Does AMH Tell Us in Pediatric Disorders of Sex Development?
Front Endocrinol (Lausanne). 2020 Sep 8;11:619. doi: 10.3389/fendo.2020.00619.
eCollection 2020. Review. PubMed [citation] PMID: 33013698, PMCID: PMC7506080

161. Bonouvrie K, van der Werff Ten Bosch J, van den Akker M. Klinefelter syndrome and
germ cell tumors: review of the literature. Int J Pediatr Endocrinol.
2020;2020:18. doi: 10.1186/s13633-020-00088-0. Epub 2020 Sep 30. Review. PubMed
[citation] PMID: 33005196, PMCID: PMC7526209

162. Rojas AP, Vo DV, Mwangi L, Rehman S, Peiris AN. Oncologic manifestations of
Klinefelter syndrome. Hormones (Athens). 2020 Dec;19(4):497-504. doi:
10.1007/s42000-020-00241-7. Epub 2020 Oct 1. Review. PubMed [citation] PMID:
33000452

163. Kyritsi EM, Kanaka-Gantenbein C. Autoimmune Thyroid Disease in Specific Genetic
Syndromes in Childhood and Adolescence. Front Endocrinol (Lausanne). 2020 Aug
19;11:543. doi: 10.3389/fendo.2020.00543. eCollection 2020. Review. PubMed
[citation] PMID: 32973676, PMCID: PMC7466763

164. Zitzmann M, Aksglaede L, Corona G, Isidori AM, Juul A, T'Sjoen G, Kliesch S,
D'Hauwers K, Toppari J, Słowikowska-Hilczer J, Tüttelmann F, Ferlin A. European
academy of andrology guidelines on Klinefelter Syndrome Endorsing Organization:
European Society of Endocrinology. Andrology. 2021 Jan;9(1):145-167. doi:
10.1111/andr.12909. Epub 2020 Oct 6. Review. PubMed [citation] PMID: 32959490

165. Chennamadhavuni A, Lyengar V, Mukkamalla SKR, Shimanovsky A. Leukemia. 2023 Jan
17. StatPearls [Internet]. Treasure Island (FL): StatPearls Publishing; 2024
Jan-. PubMed [citation] PMID: 32809325

166. Mbamognoua NGA, Aziouaz F, Matali S, Ouahabi HE, Ajdi F. [Association between

karyotype 47XYY and 5-alpha reductase deficiency revealed by micropenis: about a
case and literature review]. Pan Afr Med J. 2020 Jun 1;36:48. doi:
10.11604/pamj.2020.36.48.8209. eCollection 2020. Review. French. PubMed
[citation] PMID: 32774624, PMCID: PMC7388608

167. Urakami T. Tall stature in children and adolescents. Minerva Pediatr. 2020
Dec;72(6):472-483. doi: 10.23736/S0026-4946.20.05971-X. Epub 2020 Aug 4. Review.
PubMed [citation] PMID: 32748612

168. Hammami MB, Elkhapery A. Sexual and developmental aspects of 49, XXXXY Syndrome:
A case report. Andrologia. 2020 Nov;52(10):e13771. doi: 10.1111/and.13771. Epub
2020 Jul 29. Review. PubMed [citation] PMID: 32725928

169. Kirschner S, Yuan K, Ingleson MJ. Haloboration: scope, mechanism and utility. New
J Chem. 2020 Jul 8;45(33):14855-14868. doi: 10.1039/d0nj02908d. eCollection 2021
Aug 23. Review. PubMed [citation] PMID: 34483652, PMCID: PMC8381870

170. Garmpis N, Damaskos C, Garmpi A, Nikolettos K, Dimitroulis D, Diamantis E,
Farmaki P, Patsouras A, Voutyritsa E, Syllaios A, Zografos CG, Antoniou EA,
Nikolettos N, Kostakis A, Kontzoglou K, Schizas D, Nonni A. Molecular
Classification and Future Therapeutic Challenges of Triple-negative Breast
Cancer. In Vivo. 2020 Jul-Aug;34(4):1715-1727. doi: 10.21873/invivo.11965.
Review. PubMed [citation] PMID: 32606140, PMCID: PMC7439891

171. Coelingh Bennink HJT, Egberts JFM, Mol JA, Roes KCB, van Diest PJ. Breast Cancer
and Major Deviations of Genetic and Gender-related Structures and Function. J
Clin Endocrinol Metab. 2020 Sep 1;105(9). pii: dgaa404. doi:
10.1210/clinem/dgaa404. Review. PubMed [citation] PMID: 32594127

172. Pizzocaro A, Vena W, Condorelli R, Radicioni A, Rastrelli G, Pasquali D, Selice
R, Ferlin A, Foresta C, Jannini EA, Maggi M, Lenzi A, Pivonello R, Isidori AM,
Garolla A, Francavilla S, Corona G; King, Klinefelter ItaliaN Group..
Testosterone treatment in male patients with Klinefelter syndrome: a systematic

review and meta-analysis. J Endocrinol Invest. 2020
Dec;43(12):1675-1687. doi:
10.1007/s40618-020-01299-1. Epub 2020 Jun 21. PubMed [citation] PMID:
32567016

173. Díaz-Flores L, Gutiérrez R, García MP, Gayoso S, Gutiérrez E,
Díaz-Flores L Jr,
Carrasco JL. Telocytes in the Normal and Pathological Peripheral
Nervous System.
Int J Mol Sci. 2020 Jun 17;21(12). pii: E4320. doi: 10.3390/
ijms21124320. Review.
PubMed [citation] PMID: 32560571, PMCID: PMC7352954

174. Chen W, Bai MZ, Yang Y, Sun D, Wu S, Sun J, Wu Y, Feng Y, Wei Y,
Chen Z, Zhang Z.
ART strategies in Klinefelter syndrome. J Assist Reprod Genet. 2020
Sep;37(9):2053-2079. doi: 10.1007/s10815-020-01818-2. Epub 2020 Jun
20. Review.
PubMed [citation] PMID: 32562095, PMCID: PMC7492331

175. Cangiano B, Indirli R, Profka E, Castellano E, Goggi G, Vezzoli
V, Mantovani G,
Arosio M, Persani L, Borretta G, Ferrante E, Bonomi M. Central
hypogonadism in
Klinefelter syndrome: report of two cases and review of the
literature. J
Endocrinol Invest. 2021 Mar;44(3):459-470. doi: 10.1007/
s40618-020-01324-3. Epub
2020 Jun 14. Review. PubMed [citation] PMID: 32537678

176. Ye Q, Wang H, Xia X, Zhou C, Liu Z, Xia ZE, Zhang Z, Zhao Y,
Yehenala J, Wang S,
Zhou G, Hu K, Wu B, Wu CT, Wang S, He Y. Safety and efficacy
assessment of
allogeneic human dental pulp stem cells to treat patients with severe
COVID-19:
structured summary of a study protocol for a randomized controlled
trial (Phase I
/ II). Trials. 2020 Jun 12;21(1):520. doi: 10.1186/s13063-020-04380-5.
PubMed
[citation] PMID: 32532356, PMCID: PMC7290137

177. Arshad MA, Majzoub A, Esteves SC. Predictors of surgical sperm
retrieval in
non-obstructive azoospermia: summary of current literature. Int Urol
Nephrol.
2020 Nov;52(11):2015-2038. doi: 10.1007/s11255-020-02529-4. Epub 2020
Jun 9.
Review. PubMed [citation] PMID: 32519242

178. Tartaglia N, Howell S, Davis S, Kowal K, Tanda T, Brown M, Boada C, Alston A,
Crawford L, Thompson T, van Rijn S, Wilson R, Janusz J, Ross J. Early neurodevelopmental and medical profile in children with sex chromosome trisomies:
Background for the prospective eXtraordinarY babies study to identify early risk
factors and targets for intervention. Am J Med Genet C Semin Med Genet. 2020
Jun;184(2):428-443. doi: 10.1002/ajmg.c.31807. Epub 2020 Jun 7. Review. PubMed
[citation] PMID: 32506668, PMCID: PMC7413625

179. Berglund A, Stochholm K, Gravholt CH. The epidemiology of sex chromosome
abnormalities. Am J Med Genet C Semin Med Genet. 2020
Jun;184(2):202-215. doi:
10.1002/ajmg.c.31805. Epub 2020 Jun 7. Review. PubMed [citation] PMID: 32506765

180. Day NC, Kumar S, Criner G, Dransfield M, Halpin DMG, Han MK, Jones CE, Kaisermann
MC, Kilbride S, Lange P, Lomas DA, Martin N, Martinez FJ, Singh D, Wise R, Lipson
DA. Single-inhaler triple therapy fluticasone furoate/umeclidinium/vilanterol
versus fluticasone furoate/vilanterol and umeclidinium/vilanterol in patients
with COPD: results on cardiovascular safety from the IMPACT trial. Respir Res.
2020 Jun 5;21(1):139. doi: 10.1186/s12931-020-01398-w. PubMed [citation] PMID:
32503599, PMCID: PMC7275457

181. Chang S, Skakkebaek A, Davis SM, Gravholt CH. Morbidity in Klinefelter syndrome
and the effect of testosterone treatment. Am J Med Genet C Semin Med Genet. 2020
Jun;184(2):344-355. doi: 10.1002/ajmg.c.31798. Epub 2020 Jun 4. Review. PubMed
[citation] PMID: 32496001, PMCID: PMC7413637

182. Omeh DJ, Shlofmitz E. Angiography. 2023 Aug 7. StatPearls [Internet]. Treasure
Island (FL): StatPearls Publishing; 2024 Jan-. PubMed [citation] PMID: 32491409

183. Sood B, Clemente Fuentes RW. Jacobs Syndrome. 2022 Sep 26. StatPearls [Internet].
Treasure Island (FL): StatPearls Publishing; 2024 Jan-. PubMed

[citation] PMID:
32491631

184. Mehmood KT, Rentea RM. Ambiguous Genitalia and Disorders of Sexual
Differentiation. 2023 Aug 28. StatPearls [Internet]. Treasure Island (FL):
StatPearls Publishing; 2024 Jan–. PubMed [citation] PMID: 32491367

185. Skakkebaek A, Viuff M, Nielsen MM, Gravholt CH. Epigenetics and genomics in
Klinefelter syndrome. Am J Med Genet C Semin Med Genet. 2020 Jun;184(2):216-225.
doi: 10.1002/ajmg.c.31802. Epub 2020 Jun 2. Review. PubMed [citation] PMID:
32484281

186. Aksglaede L, Davis SM, Ross JL, Juul A. Minipuberty in Klinefelter syndrome:
Current status and future directions. Am J Med Genet C Semin Med Genet. 2020
Jun;184(2):320-326. doi: 10.1002/ajmg.c.31794. Epub 2020 Jun 1. Review. PubMed
[citation] PMID: 32476267, PMCID: PMC7413638

187. Skakkebaek A, Gravholt CH, Chang S, Moore PJ, Wallentin M. Psychological
functioning, brain morphology, and functional neuroimaging in Klinefelter
syndrome. Am J Med Genet C Semin Med Genet. 2020 Jun;184(2):506-517. doi:
10.1002/ajmg.c.31806. Epub 2020 May 28. Review. PubMed [citation]
PMID: 32468713

188. Karipidis II, Hong DS. Specific learning disorders in sex chromosome
aneuploidies: Neural circuits of literacy and mathematics. Am J Med Genet C Semin
Med Genet. 2020 Jun;184(2):518-530. doi: 10.1002/ajmg.c.31801. Epub 2020 May 28.
Review. PubMed [citation] PMID: 32463563

189. Spaziani M, Radicioni AF. Metabolic and cardiovascular risk factors in
Klinefelter syndrome. Am J Med Genet C Semin Med Genet. 2020 Jun;184(2):334-343.
doi: 10.1002/ajmg.c.31792. Epub 2020 May 26. Review. PubMed [citation] PMID:
32452627

190. Winge SB, Soraggi S, Schierup MH, Rajpert-De Meyts E, Almstrup K. Integration and
reanalysis of transcriptomics and methylomics data derived from blood and testis
tissue of men with 47,XXY Klinefelter syndrome indicates the primary involvement
of Sertoli cells in the testicular pathogenesis. Am J Med Genet C Semin Med
Genet. 2020 Jun;184(2):239-255. doi: 10.1002/ajmg.c.31793. Epub 2020 May 25.
Review. PubMed [citation] PMID: 32449318

191. Rey RA, Grinspon RP. Androgen Treatment in Adolescent Males With Hypogonadism. Am
J Mens Health. 2020 May-Jun;14(3):1557988320922443. doi:
10.1177/1557988320922443. Review. PubMed [citation] PMID: 32448030, PMCID:
PMC7249582

192. Navarro-Cobos MJ, Balaton BP, Brown CJ. Genes that escape from X-chromosome
inactivation: Potential contributors to Klinefelter syndrome. Am J Med Genet C
Semin Med Genet. 2020 Jun;184(2):226-238. doi: 10.1002/ajmg.c.31800. Epub 2020
May 22. Review. PubMed [citation] PMID: 32441398, PMCID: PMC7384012

193. Wistuba J, Beumer C, Brehm R, Gromoll J. 41,XX(Y) * male mice: An animal model
for Klinefelter syndrome. Am J Med Genet C Semin Med Genet. 2020
Jun;184(2):267-278. doi: 10.1002/ajmg.c.31796. Epub 2020 May 20. Review. PubMed
[citation] PMID: 32432406

194. Iqbal F. Meiotic Behavior of Extra Sex Chromosomes in Patients with the 47,XXY
and 47,XYY Karyotype and Its Ultimate Consequences for Spermatogenesis. Crit Rev
Eukaryot Gene Expr. 2020;30(1):19-37. doi:
10.1615/CritRevEukaryotGeneExpr.2020026203. Review. PubMed [citation] PMID:
32421982

195. Zitzmann M, Rohayem J. Gonadal dysfunction and beyond: Clinical challenges in
children, adolescents, and adults with 47,XXY Klinefelter syndrome. Am J Med
Genet C Semin Med Genet. 2020 Jun;184(2):302-312. doi: 10.1002/ajmg.c.31786. Epub
2020 May 16. Review. PubMed [citation] PMID: 32415901

196. Willems M, Gies I, Van Saen D. Germ cell loss in Klinefelter syndrome: When and
why? Am J Med Genet C Semin Med Genet. 2020 Jun;184(2):356-370. doi:
10.1002/ajmg.c.31787. Epub 2020 May 15. Review. PubMed [citation]
PMID: 32412180

197. Di Palo A, Siniscalchi C, Salerno M, Russo A, Gravholt CH, Potenza N. What
microRNAs could tell us about the human X chromosome. Cell Mol Life Sci. 2020
Oct;77(20):4069-4080. doi: 10.1007/s00018-020-03526-7. Epub 2020 Apr 30. Review.
Erratum in: Cell Mol Life Sci. 2021 Apr;78(8):4067. PubMed [citation] PMID:
32356180, PMCID: PMC7854456

198. Kim MJ, Jeong HR. Spontaneous Sexual Development and Heavy Menstrual Bleeding in
45,X Monosomy and 45,X/47,XXX Mosaic Turner Syndrome and a Review of the
Literature. J Pediatr Adolesc Gynecol. 2020 Oct;33(5):602-606. doi:
10.1016/j.jpag.2020.04.002. Epub 2020 Apr 18. Review. PubMed [citation] PMID:
32315714

199. Herati AS, Kohn TP, Kassiri B. New frontiers in fertility preservation: a
hypothesis on fertility optimization in men with hypergonadotrophic hypogonadism.
Transl Androl Urol. 2020 Mar;9(Suppl 2):S171-S177. doi: 10.21037/tau.2019.12.39.
Review. PubMed [citation] PMID: 32257857, PMCID: PMC7108987

200. Montemurro F, Nuzzolese I, Ponzone R. Neoadjuvant or adjuvant chemotherapy in
early breast cancer? Expert Opin Pharmacother. 2020 Jun;21(9):1071-1082. doi:
10.1080/14656566.2020.1746273. Epub 2020 Apr 1. Review. PubMed [citation] PMID:
32237920

201. Masterson TA 3rd, Nassau DE, Ramasamy R. A clinical algorithm for management of
fertility in adolescents with the Klinefelter syndrome. Curr Opin Urol. 2020
May;30(3):324-327. doi: 10.1097/MOU.0000000000000757. Review. PubMed [citation]
PMID: 32235276

202. Nassau DE, Chu KY, Blachman-Braun R, Castellan M, Ramasamy R. The pediatric
patient and future fertility: optimizing long-term male reproductive health
outcomes. Fertil Steril. 2020 Mar;113(3):489-499. doi:
10.1016/j.fertnstert.2020.01.003. Review. PubMed [citation] PMID: 32192588

203. Rogol AD. Human sex chromosome aneuploidies: The hypothalamic-pituitary-gonadal
axis. Am J Med Genet C Semin Med Genet. 2020 Jun;184(2):313-319. doi:
10.1002/ajmg.c.31782. Epub 2020 Mar 14. Review. PubMed [citation]
PMID: 32170911

204. Murugaiah V, Tsolaki AG, Kishore U. Collectins: Innate Immune Pattern Recognition
Molecules. Adv Exp Med Biol. 2020;1204:75-127. doi: 10.1007/978-981-15-1580-4_4.
Review. PubMed [citation] PMID: 32152944, PMCID: PMC7120701

205. Mason KA, Schoelwer MJ, Rogol AD. Androgens During Infancy, Childhood, and
Adolescence: Physiology and Use in Clinical Practice. Endocr Rev. 2020 Jun
1;41(3). pii: bnaa003. doi: 10.1210/endrev/bnaa003. Review. PubMed [citation]
PMID: 32115641

206. Zhang X, Liu X, Xi Q, Zhu H, Li L, Liu R, Yu Y. Reproductive outcomes of 3
infertile males with XYY syndrome: Retrospective case series and literature
review. Medicine (Baltimore). 2020 Feb;99(9):e19375. doi:
10.1097/MD.0000000000019375. Review. PubMed [citation] PMID: 32118782, PMCID:
PMC7478696

207. Vitacca M, Lastoria C, Delmastro M, Fiorenza D, De Cata P, Fusar Poli B, Gilè S,
Prometti P, Paneroni M, Bianchi C, Mandora E, Porri R, Fracchia C. Use of inhaled
devices during a hospital exacerbation of COPD: a summary of an interdisciplinary
audit held at ICS Maugeri Pavia, Italy (March-June 2019). Monaldi Arch Chest Dis.
2020 Feb 12;90(1). doi: 10.4081/monaldi.2020.1176. PubMed [citation] PMID:
32072800

208. Efstathopoulou A, Ghielmini M, Zucca E. MYC/BCL2/BCL6 triple hit

lymphoma of the
pericardium: a case report and review of the literature. J Cancer Res Clin Oncol.
2020 Sep;146(9):2435-2438. doi: 10.1007/s00432-020-03137-6. Epub 2020 Feb 6.
Review. No abstract available. PubMed [citation] PMID: 32025812

209. Faure-Conter C, Orbach D, Fresneau B, Verité C, Bonneau J, Thebaud E, Poirée M,
Thouvenin S, Pluchart C, Mure PY, Dijoud F, Morel Y. Disorder of sex development
with germ cell tumors: Which is uncovered first? Pediatr Blood Cancer. 2020
Apr;67(4):e28169. doi: 10.1002/pbc.28169. Epub 2020 Feb 4. PubMed [citation]
PMID: 32020769

210. Monica MP, Merkely B, Szilveszter B, Drobni ZD, Maurovich-Horvat P. Computed
Tomographic Angiography for Risk Stratification in Patients with Acute Chest Pain
- The Triple Rule-out Concept in the Emergency Department. Curr Med Imaging Rev.
2020;16(2):98-110. doi: 10.2174/1573405614666180604095120. Review. PubMed
[citation] PMID: 32003310

211. Samango-Sprouse CA, Yu C, Porter GF, Tipton ES, Lasutschinkow PC, Gropman AL. A
review of the intriguing interaction between testosterone and neurocognitive
development in males with 47,XXY. Curr Opin Obstet Gynecol. 2020
Apr;32(2):140-146. doi: 10.1097/GCO.0000000000000612. Review. PubMed [citation]
PMID: 32004174

212. Pinti E, Lengyel A, Fekete G, Haltrich I. What should we consider in the case of
combined Down- and 47,XY,+i(X)(q10) Klinefelter syndromes? The unique case of a
male newborn and review of the literature. BMC Pediatr. 2020 Jan 13;20(1):17.
doi: 10.1186/s12887-019-1905-9. Review. PubMed [citation] PMID: 31931754, PMCID:
PMC6958764

213. Ahmad A, Mishra RK, Vyawahare A, Kumar A, Rehman MU, Qamar W, Khan AQ, Khan R.
Thymoquinone (2-Isoprpyl-5-methyl-1, 4-benzoquinone) as a
chemopreventive/anticancer agent: Chemistry and biological effects.

Saudi Pharm
J. 2019 Dec;27(8):1113-1126. doi: 10.1016/j.jsps.2019.09.008. Epub
2019 Sep 25.
Review. PubMed [citation] PMID: 31885471, PMCID: PMC6921197

214. Vockel M, Riera-Escamilla A, Tüttelmann F, Krausz C. The X
chromosome and male
infertility. Hum Genet. 2021 Jan;140(1):203-215. doi: 10.1007/
s00439-019-02101-w.
Epub 2019 Dec 24. Review. PubMed [citation] PMID: 31875237, PMCID:
PMC7864851

215. Caparica R, Brandão M, Piccart M. Systemic treatment of patients
with early
breast cancer: recent updates and state of the art. Breast. 2019
Nov;48 Suppl
1:S7-S20. doi: 10.1016/S0960-9776(19)31115-4. Review. PubMed
[citation] PMID:
31839166

216. Deebel NA, Galdon G, Zarandi NP, Stogner-Underwood K, Howards S,
Lovato J, Kogan
S, Atala A, Lue Y, Sadri-Ardekani H. Age-related presence of
spermatogonia in
patients with Klinefelter syndrome: a systematic review and meta-
analysis. Hum
Reprod Update. 2020 Jan 1;26(1):58-72. doi: 10.1093/humupd/dmz038.
PubMed
[citation] PMID: 31822886

217. Friedrich RE, Kohlrusch FK, Luebke AM. Symptomatic Mandibular
Fibrous Dysplasia
With Concurrent Triple X- and Premutation Stage Fragile-X-Syndrome:
Case Report
With Short Literature Survey. Anticancer Res. 2019
Dec;39(12):6769-6780. doi:
10.21873/anticanres.13892. Review. PubMed [citation] PMID: 31810942

218. Perrett KP, Halperin SA, Nolan T, Carmona Martínez A, Martinón-
Torres F,
García-Sicilia J, Virta M, Vanderkooi OG, Zuccotti GV, Manzoni P,
Kostanyan L,
Meyer N, Ceregido MA, Cheuvart B, Kuriyakose SO, Stranak Z, Merino
Arribas JM,
Cilleruelo Ortega MJ, Miranda-Valdivieso M, Arias Novas B, Ramos
Amador JT,
Omeñaca F, et al. Impact of tetanus-diphtheria-acellular pertussis
immunization
during pregnancy on subsequent infant immunization seroresponses:
follow-up from

a large randomized placebo-controlled trial. Vaccine. 2020 Feb
18;38(8):2105-2114. doi: 10.1016/j.vaccine.2019.10.104. Epub 2019 Nov
24. PubMed
[citation] PMID: 31776027

219. Perrett KP, Halperin SA, Nolan T, Martínez Pancorbo C, Tapiero B,
Martinón-Torres
F, Stranak Z, Virta M, Vanderkooi OG, Kosina P, Encinas Pardilla MB,
Cristobal
García I, Zuccotti GV, Kostanyan L, Meyer N, Ceregido MA, Cheuvart B,
Kuriyakose
SO, Marcos Fernández M, Rodríguez Zambrano MÁ, Martín García A, Asenjo
de la
Fuente JE, et al. Immunogenicity, transplacental transfer of pertussis
antibodies
and safety following pertussis immunization during pregnancy: Evidence
from a
randomized, placebo-controlled trial. Vaccine. 2020 Feb
18;38(8):2095-2104. doi:
10.1016/j.vaccine.2019.10.105. Epub 2019 Nov 24. PubMed [citation]
PMID: 31776029

220. McGraw KL, Cheng CH, Chen YA, Hou HA, Nilsson B, Genovese G,
Cluzeau T,
Pellagatti A, Przychodzen BP, Mallo M, Arenillas L, Mohamedali A, Adès
L, Sallman
DA, Padron E, Sokol L, Moreilhon C, Raynaud S, Tien HF, Boultwood J,
Ebert BL,
Sole F, et al. Non-del(5q) myelodysplastic syndromes-associated loci
detected by
SNP-array genome-wide association meta-analysis. Blood Adv. 2019 Nov
26;3(22):3579-3589. doi: 10.1182/bloodadvances.2019000922. PubMed
[citation]
PMID: 31738830, PMCID: PMC6880887

221. Baker JR, Sakoff JA, McCluskey A. The aryl hydrocarbon receptor
(AhR) as a breast
cancer drug target. Med Res Rev. 2020 May;40(3):972-1001. doi:
10.1002/med.21645.
Epub 2019 Nov 12. Review. PubMed [citation] PMID: 31721255

222. Samango-Sprouse CA, Counts DR, Tran SL, Lasutschinkow PC, Porter
GF, Gropman AL.
Update On The Clinical Perspectives And Care Of The Child With 47,XXY
(Klinefelter Syndrome). Appl Clin Genet. 2019 Oct 23;12:191-202. doi:
10.2147/TACG.S180450. eCollection 2019. Review. PubMed [citation]
PMID: 31695472,
PMCID: PMC6815760

223. Corona G, Minhas S, Giwercman A, Bettocchi C, Dinkelman-Smit M,

Dohle G, Fusco F,
Kadioglou A, Kliesch S, Kopa Z, Krausz C, Pelliccione F, Pizzocaro A, Rassweiler
J, Verze P, Vignozzi L, Weidner W, Maggi M, Sofikitis N. Sperm recovery and ICSI
outcomes in men with non-obstructive azoospermia: a systematic review and
meta-analysis. Hum Reprod Update. 2019 Nov 5;25(6):733-757. doi:
10.1093/humupd/dmz028. PubMed [citation] PMID: 31665451

224. Haltrich I. Chromosomal Aberrations with Endocrine Relevance (Turner Syndrome,
Klinefelter Syndrome, Prader-Willi Syndrome). Exp Suppl. 2019;111:443-473. doi:
10.1007/978-3-030-25905-1_20. Review. PubMed [citation] PMID: 31588543

225. Fainberg J, Hayden RP, Schlegel PN. Fertility management of Klinefelter syndrome.
Expert Rev Endocrinol Metab. 2019 Nov;14(6):369-380. doi:
10.1080/17446651.2019.1671821. Epub 2019 Oct 7. Review. PubMed [citation] PMID:
31587581

226. Palka C, Guanciali-Franchi P, Morizio E, Alfonsi M, Papponetti M, Sabbatinelli G,
Palka G, Calabrese G, Benn P. Non-invasive prenatal screening: A 20-year
experience in Italy. Eur J Obstet Gynecol Reprod Biol X. 2019 May 18;3:100050.
doi: 10.1016/j.eurox.2019.100050. eCollection 2019 Jul. Review. PubMed [citation]
PMID: 31403132, PMCID: PMC6687402

227. Singh D, Beeh KM, Colgan B, Kornmann O, Leaker B, Watz H, Lucci G, Geraci S,
Emirova A, Govoni M, Nandeuil MA. Effect of the inhaled PDE4 inhibitor CHF6001 on
biomarkers of inflammation in COPD. Respir Res. 2019 Aug 9;20(1):180. doi:
10.1186/s12931-019-1142-7. PubMed [citation] PMID: 31399091, PMCID:
PMC6688371

228. Mathis G. [Use of lung and pleural ultrasonography in emergency and intensive
care medicine]. Med Klin Intensivmed Notfmed. 2019 Sep;114(6):504-508. doi:
10.1007/s00063-019-0596-1. Epub 2019 Aug 7. Review. German. PubMed [citation]
PMID: 31392352, PMCID: PMC7096083

229. Di Cosimo S, La Verde N, Moretti A, Cazzaniga ME, Generali D, Bianchi GV, Mariani
L, Torri V, Crippa F, Paolini B, Scaperrotta G, De Santis MC, Di Nicola M,
Apolone G, Gulino A, Tripodo C, Colombo MP, Folli S, de Braud F. Neoadjuvant
eribulin mesylate following anthracycline and taxane in triple negative breast
cancer: Results from the HOPE study. PLoS One. 2019 Aug 7;14(8):e0220644. doi:
10.1371/journal.pone.0220644. eCollection 2019. PubMed [citation] PMID: 31390375,
PMCID: PMC6685628

230. O'Connor MJ, Snyder EA, Hayes FJ. Klinefelter Syndrome and Diabetes. Curr Diab
Rep. 2019 Jul 31;19(9):71. doi: 10.1007/s11892-019-1197-3. Review. PubMed
[citation] PMID: 31367971

231. Ladjouze A, Donaldson M. Primary gonadal failure. Best Pract Res Clin Endocrinol
Metab. 2019 Jun;33(3):101295. doi: 10.1016/j.beem.2019.101295. Epub 2019 Jul 12.
Review. PubMed [citation] PMID: 31327696

232. Lauffer P, Kamp GA, Menke LA, Wit JM, Oostdijk W;
on behalf of the Dutch Working Group on Triage and Diagnosis of Growth Disorders
in Children.. Towards a Rational and Efficient Diagnostic Approach in Children
Referred for Tall Stature and/or Accelerated Growth to the General Paediatrician.
Horm Res Paediatr. 2019;91(5):293-310. doi: 10.1159/000500810. Epub 2019 Jul 12.
Review. PubMed [citation] PMID: 31302655

233. Merg AD, Touponse G, van Genderen E, Zuo X, Bazrafshan A, Blum T, Hughes S,
Salaita K, Abrahams JP, Conticello VP. 2D Crystal Engineering of Nanosheets
Assembled from Helical Peptide Building Blocks. Angew Chem Int Ed Engl. 2019 Sep
16;58(38):13507-13512. doi: 10.1002/anie.201906214. Epub 2019 Aug 12. Review.
PubMed [citation] PMID: 31291499

234. Pusztai L, Foldi J, Dhawan A, DiGiovanna MP, Mamounas EP. Changing frameworks in
treatment sequencing of triple-negative and HER2-positive, early-stage

breast
cancers. Lancet Oncol. 2019 Jul;20(7):e390-e396. doi:
10.1016/S1470-2045(19)30158-5. Review. PubMed [citation] PMID:
31267973

235. Lu Y, Zhang S, Wang Y, Ren X, Han J. Molecular mechanisms and clinical
manifestations of rare genetic disorders associated with type I collagen.
Intractable Rare Dis Res. 2019 May;8(2):98-107. doi: 10.5582/irdr.2019.01064.
Review. PubMed [citation] PMID: 31218159, PMCID: PMC6557237

236. Ferràs-Tarragó J, Valencia JMM, Belmar PR, Vergara SP, Gómez PJ, Hermida JLB,
Hermida PB, Hermida TB. Cobb angle measurement with a conventional convex
echography probe and a smartphone. Eur Spine J. 2019 Sep;28(9):1955-1961. doi:
10.1007/s00586-019-06030-0. Epub 2019 Jun 14. PubMed [citation] PMID:
31201564

237. Hua L, Zhang J, Zhang S, Zhang C, Wang Z, Zhang Y. [Application of iodine
contrast agent optimization protocol with fixed injection time in triple-rule-out
CT examination of chest pain]. Zhonghua Wei Zhong Bing Ji Jiu Yi Xue. 2019
May;31(5):582-587. doi: 10.3760/cma.j.issn.2095-4352.2019.05.012. Chinese. PubMed
[citation] PMID: 31198144

238. Junker D, Stöggl T. The Training Effects of Foam Rolling on Core Strength
Endurance, Balance, Muscle Performance and Range of Motion: A Randomized
Controlled Trial. J Sports Sci Med. 2019 Jun 1;18(2):229-238. eCollection 2019
Jun. PubMed [citation] PMID: 31191092, PMCID: PMC6543984

239. Chan YM, Feld A, Jonsdottir-Lewis E. Effects of the Timing of Sex-Steroid
Exposure in Adolescence on Adult Health Outcomes. J Clin Endocrinol Metab. 2019
Oct 1;104(10):4578-4586. doi: 10.1210/jc.2019-00569. Review. PubMed [citation]
PMID: 31194243, PMCID: PMC6736212

240. Wijaya M, Huamei M, Jun Z, Du M, Li Y, Chen Q, Chen H, Song G. Etiology of

primary adrenal insufficiency in children: a 29-year single-center experience. J
Pediatr Endocrinol Metab. 2019 Jun 26;32(6):615-622. doi: 10.1515/jpem-2018-0445.
PubMed [citation] PMID: 31141483

241. Westhoff B, Lederer C, Krauspe R. [Perthes disease-news in diagnostics and
treatment]. Orthopade. 2019 Jun;48(6):515-522. doi: 10.1007/s00132-019-03737-2.
Review. German. PubMed [citation] PMID: 31139870

242. Zain R, Smith CIE. Targeted Oligonucleotides for Treating Neurodegenerative
Tandem Repeat Diseases. Neurotherapeutics. 2019 Apr;16(2):248-262. doi:
10.1007/s13311-019-00712-9. Review. PubMed [citation] PMID: 31098852, PMCID:
PMC6554256

243. Tang R, Lin L, Guo Z, Hou H, Yu Q. Ovarian reserve evaluation in a woman with
45,X/47,XXX mosaicism: A case report and a review of literature. Mol Genet
Genomic Med. 2019 Jul;7(7):e00732. doi: 10.1002/mgg3.732. Epub 2019 May 8.
Review. PubMed [citation] PMID: 31070017, PMCID: PMC6625135

244. Truong L, Ferré-D'Amaré AR. From fluorescent proteins to fluorogenic RNAs: Tools
for imaging cellular macromolecules. Protein Sci. 2019 Aug;28(8):1374-1386. doi:
10.1002/pro.3632. Epub 2019 May 11. Review. PubMed [citation] PMID: 31017335,
PMCID: PMC6635776

245. Shiraishi K, Matsuyama H. Klinefelter syndrome: From pediatrics to geriatrics.
Reprod Med Biol. 2018 Dec 8;18(2):140-150. doi: 10.1002/rmb2.12261. eCollection
2019 Apr. Review. PubMed [citation] PMID: 30996677, PMCID: PMC6452011

246. Kaser S, Winhofer-Stöckl Y, Kazemi-Shirazi L, Hofer SE, Brath H, Sourij H, Vila
G, Abrahamian H, Riedl M, Weitgasser R, Resl M, Clodi M, Luger A. [Other specific
types of diabetes and exocrine pancreatic insufficiency (Update 2019)]. Wien Klin
Wochenschr. 2019 May;131(Suppl 1):16-26. doi: 10.1007/s00508-019-1454-0. Review.

German. PubMed [citation] PMID: 30980164

247. Mohammed SK, Jan A. Young Syndrome. 2023 Aug 22. StatPearls [Internet]. Treasure
Island (FL): StatPearls Publishing; 2024 Jan–. PubMed [citation] PMID: 30969689

248. Li L, Gong C. Central precocious puberty as a prelude of gonad dysplasia. Pediatr
Investig. 2019 Mar 22;3(1):50-54. doi: 10.1002/ped4.12118. eCollection 2019 Mar.
Review. PubMed [citation] PMID: 32851289, PMCID: PMC7331341

249. Farid A, Faber FWM. Bilateral Triple Talocalcaneal, Calcaneonavicular, and
Talonavicular Tarsal Coalition: A Case Report. J Foot Ankle Surg. 2019
Mar;58(2):374-376. doi: 10.1053/j.jfas.2018.08.047. Review. PubMed [citation]
PMID: 30850104

250. Viuff M, Skakkebaek A, Nielsen MM, Chang S, Gravholt CH. Epigenetics and genomics
in Turner syndrome. Am J Med Genet C Semin Med Genet. 2019 Mar;181(1):68-75. doi:
10.1002/ajmg.c.31683. Epub 2019 Feb 27. Review. PubMed [citation] PMID: 30811826

251. San Roman AK, Page DC. A strategic research alliance: Turner syndrome and sex
differences. Am J Med Genet C Semin Med Genet. 2019 Mar;181(1):59-67. doi:
10.1002/ajmg.c.31677. Epub 2019 Feb 20. Review. PubMed [citation] PMID: 30790449,
PMCID: PMC6414291

252. Accardo G, Amoresano Paglionico V, Di Fraia R, Cittadini A, Salzano A, Esposito
D, De Bellis A, Pasquali D. Management of cardiovascular complications in
Klinefelter syndrome patients. Expert Rev Endocrinol Metab. 2019
Mar;14(2):145-152. doi: 10.1080/17446651.2019.1584036. Epub 2019 Feb 22. PubMed
[citation] PMID: 30793993

253. Jung KH, LoRusso P, Burris H, Gordon M, Bang YJ, Hellmann MD, Cervantes A, Ochoa
de Olza M, Marabelle A, Hodi FS, Ahn MJ, Emens LA, Barlesi F, Hamid O, Calvo E,
McDermott D, Soliman H, Rhee I, Lin R, Pourmohamad T, Suchomel J, Tsuhako A, et

al. Phase I Study of the Indoleamine 2,3-Dioxygenase 1 (IDO1) Inhibitor Navoximod
(GDC-0919) Administered with PD-L1 Inhibitor (Atezolizumab) in Advanced Solid
Tumors. Clin Cancer Res. 2019 Jun 1;25(11):3220-3228. doi:
10.1158/1078-0432.CCR-18-2740. Epub 2019 Feb 15. PubMed [citation] PMID:
30770348, PMCID: PMC7980952

254. Bearelly P, Oates R. Recent advances in managing and understanding Klinefelter
syndrome. F1000Res. 2019 Jan 28;8. pii: F1000 Faculty Rev-112. doi:
10.12688/f1000research.16747.1. eCollection 2019. Review. PubMed [citation] PMID:
30755791, PMCID: PMC6352920

255. Vasani A, Kumar MS. Advances in the proteomics of amniotic fluid to detect
biomarkers for chromosomal abnormalities and fetomaternal complications during
pregnancy. Expert Rev Proteomics. 2019 Apr;16(4):277-286. doi:
10.1080/14789450.2019.1578213. Epub 2019 Feb 13. Review. PubMed [citation] PMID:
30722712

256. Giudice MG, Del Vento F, Wyns C. Male fertility preservation in DSD, XXY,
pre-gonadotoxic treatments - Update, methods, ethical issues, current outcomes,
future directions. Best Pract Res Clin Endocrinol Metab. 2019 Jun;33(3):101261.
doi: 10.1016/j.beem.2019.01.002. Epub 2019 Jan 23. Review. PubMed [citation]
PMID: 30718080

257. Gray RE, Harris GT. Renal Cell Carcinoma: Diagnosis and Management. Am Fam
Physician. 2019 Feb 1;99(3):179-184. Review. Erratum in: Am Fam Physician. 2019
Jun 15;99(12):732. PubMed [citation] PMID: 30702258

258. Santi D, De Vincentis S, Scaltriti S, Rochira V. Relative hyperestrogenism in
Klinefelter Syndrome: results from a meta-analysis. Endocrine. 2019
May;64(2):209-219. doi: 10.1007/s12020-019-01850-y. Epub 2019 Jan 30. PubMed
[citation] PMID: 30701446

259. van Rijn S. A review of neurocognitive functioning and risk for psychopathology

in sex chromosome trisomy (47,XXY, 47,XXX, 47, XYY). Curr Opin Psychiatry. 2019
Mar;32(2):79-84. doi: 10.1097/YCO.0000000000000471. Review. PubMed [citation]
PMID: 30689602, PMCID: PMC6687415

260. Maillefer A, Sabe M, Coste C, Bartolomei J, Jaafar J, Sentissi O. Sexual Identity
Disorder and Psychosis in Klinefelter Syndrome: A Synthesis of Literature and a
Case Report. J Nerv Ment Dis. 2019 Feb;207(2):121-125. doi:
10.1097/NMD.0000000000000930. Review. PubMed [citation] PMID: 30672880

261. Yu W, Lv Y, Yin S, Liu H, Li X, Liang B, Kong L, Liu C. Screening of fetal
chromosomal aneuploidy diseases using noninvasive prenatal testing in twin
pregnancies. Expert Rev Mol Diagn. 2019 Feb;19(2):189-196. doi:
10.1080/14737159.2019.1562906. Epub 2019 Jan 2. PubMed [citation]
PMID: 30582381

262. Basulto-Martínez M, Klein I, Gutiérrez-Aceves J. The role of extracorporeal shock
wave lithotripsy in the future of stone management. Curr Opin Urol. 2019
Mar;29(2):96-102. doi: 10.1097/MOU.0000000000000584. Review. PubMed [citation]
PMID: 30562186

263. Wong BKY, Sutton VR. Aicardi syndrome, an unsolved mystery: Review of diagnostic
features, previous attempts, and future opportunities for genetic examination. Am
J Med Genet C Semin Med Genet. 2018 Dec;178(4):423-431. doi:
10.1002/ajmg.c.31658. Epub 2018 Dec 10. Review. PubMed [citation]
PMID: 30536540

264. Snowsill T, Yang H, Griffin E, Long L, Varley-Campbell J, Coelho H, Robinson S,
Hyde C. Low-dose computed tomography for lung cancer screening in high-risk
populations: a systematic review and economic evaluation. Health Technol Assess.
2018 Nov;22(69):1-276. doi: 10.3310/hta22690. PubMed [citation] PMID: 30518460,
PMCID: PMC6304730

265. Lizarazo AH, McLoughlin M, Vogiatzi MG. Endocrine aspects of Klinefelter
syndrome. Curr Opin Endocrinol Diabetes Obes. 2019 Feb;26(1):60-65.

doi:
10.1097/MED.0000000000000454. Review. PubMed [citation] PMID: 30507702

266. Tsuruta S, Kimura N, Ishido K, Kudo D, Sato K, Endo T, Yoshizawa T, Sukeda A,
Hiraoka N, Kijima H, Hakamada K. Calcifying nested stromal epithelial tumor of
the liver in a patient with Klinefelter syndrome: a case report and review of the
literature. World J Surg Oncol. 2018 Nov 19;16(1):227. doi:
10.1186/s12957-018-1528-x. Review. PubMed [citation] PMID: 30453967, PMCID:
PMC6245822

267. Foland-Ross LC, Ross JL, Reiss AL. Androgen treatment effects on hippocampus
structure in boys with Klinefelter syndrome. Psychoneuroendocrinology. 2019
Feb;100:223-228. doi: 10.1016/j.psyneuen.2018.09.039. Epub 2018 Oct 2. PubMed
[citation] PMID: 30388596, PMCID: PMC6644684

268. Xu HY, Zhang HX, Xiao Z, Qiao J, Li R. Regulation of anti-Müllerian hormone (AMH)
in males and the associations of serum AMH with the disorders of male fertility.
Asian J Androl. 2019 Mar-Apr;21(2):109-114. doi: 10.4103/aja.aja_83_18. Review.
PubMed [citation] PMID: 30381580, PMCID: PMC6413543

269. Joseph L, Farmer C, Chlebowski C, Henry L, Fish A, Mankiw C, Xenophontos A,
Clasen L, Sauls B, Seidlitz J, Blumenthal J, Torres E, Thurm A, Raznahan A.
Characterization of autism spectrum disorder and neurodevelopmental profiles in
youth with XYY syndrome. J Neurodev Disord. 2018 Oct 22;10(1):30. doi:
10.1186/s11689-018-9248-7. PubMed [citation] PMID: 30348076, PMCID:
PMC6198503

270. Davies JC, Moskowitz SM, Brown C, Horsley A, Mall MA, McKone EF, Plant BJ, Prais
D, Ramsey BW, Taylor-Cousar JL, Tullis E, Uluer A, McKee CM, Robertson S,
Shilling RA, Simard C, Van Goor F, Waltz D, Xuan F, Young T, Rowe SM;
VX16-659-101 Study Group.. VX-659-Tezacaftor-Ivacaftor in Patients with Cystic
Fibrosis and One or Two Phe508del Alleles. N Engl J Med. 2018 Oct
25;379(17):1599-1611. doi: 10.1056/NEJMoa1807119. Epub 2018 Oct 18. PubMed

[citation] PMID: 30334693, PMCID: PMC6277022

271. Souyris M, Mejía JE, Chaumeil J, Guéry JC. Female predisposition to TLR7-driven
autoimmunity: gene dosage and the escape from X chromosome inactivation. Semin
Immunopathol. 2019 Mar;41(2):153-164. doi: 10.1007/s00281-018-0712-y. Epub 2018
Oct 1. Review. PubMed [citation] PMID: 30276444

272. Ballegeer R, Demyttenaere K, Simons W. [The Klinefelter syndrome: a systematic
review of secondary psychiatric comorbidities]. Tijdschr Psychiatr.
2018;60(9):606-618. Dutch. PubMed [citation] PMID: 30215449

273. Stangl MK, Böcker W, Chubanov V, Ferrari U, Fischereder M, Gudermann T, Hesse E,
Meinke P, Reincke M, Reisch N, Saller MM, Seissler J, Schmidmaier R, Schoser B,
Then C, Thorand B, Drey M. Sarcopenia - Endocrinological and Neurological
Aspects. Exp Clin Endocrinol Diabetes. 2019 Jan;127(1):8-22. doi:
10.1055/a-0672-1007. Epub 2018 Sep 10. Review. PubMed [citation] PMID: 30199918

274. Jiang T, Zhao Q, Huang M, Sun J, Tian G. Contrast-Enhanced Ultrasound in Residual
Tumor of Hepatocellular Carcinoma following Transarterial Chemoembolization: Is
It Helpful for Tumor Response? Biomed Res Int. 2018 Aug 2;2018:8632069. doi:
10.1155/2018/8632069. eCollection 2018. PubMed [citation] PMID: 30175147, PMCID:
PMC6098931

275. Green T, Flash S, Reiss AL. Sex differences in psychiatric disorders: what we can
learn from sex chromosome aneuploidies. Neuropsychopharmacology. 2019
Jan;44(1):9-21. doi: 10.1038/s41386-018-0153-2. Epub 2018 Jul 16. Review. PubMed
[citation] PMID: 30127341, PMCID: PMC6235860

276. Richard-Eaglin A. Male and Female Hypogonadism. Nurs Clin North Am. 2018
Sep;53(3):395-405. doi: 10.1016/j.cnur.2018.04.006. Review. PubMed [citation]
PMID: 30100005

277. O'Brien MER, Sarker D, Bhosle J, Thillai K, Yap TA, Uttenreuther-Fischer M,

Pemberton K, Jin X, Wiebe S, de Bono J, Spicer J. A phase I study to assess
afatinib in combination with carboplatin or with carboplatin plus paclitaxel in
patients with advanced solid tumors. Cancer Chemother Pharmacol. 2018
Nov;82(5):757-766. doi: 10.1007/s00280-018-3661-1. Epub 2018 Aug 7. PubMed
[citation] PMID: 30088048, PMCID: PMC6182763

278. Yu Y, Xi Q, Jing J, Li L, Zhang H, Liu R, Pan Y. Intracytoplasmic sperm injection
outcome of ejaculated spermatozoa from a man with mosaic Klinefelter's Syndrome:
case report and literature review. J Int Med Res. 2018
Oct;46(10):4323-4331. doi:
10.1177/0300060518788757. Epub 2018 Jul 30. Review. PubMed [citation] PMID:
30060725, PMCID: PMC6166348

279. Mavridi A, Ntali G, Theodora M, Stamatelopoulos K, Michala L. A Spontaneous
Pregnancy in a Patient with Turner Syndrome with 45,X/47,XXX Mosaicism: A Case
Report and Review of the Literature. J Pediatr Adolesc Gynecol. 2018
Dec;31(6):651-654. doi: 10.1016/j.jpag.2018.07.005. Epub 2018 Aug 20. Review.
PubMed [citation] PMID: 30012427

280. Bacitracin. Drugs and Lactation Database (LactMed®) [Internet]. Bethesda (MD):
National Institute of Child Health and Human Development; 2006-. PubMed
[citation] PMID: 30000490

281. Hwu EE, Boisen A. Hacking CD/DVD/Blu-ray for Biosensing. ACS Sens. 2018 Jul
27;3(7):1222-1232. doi: 10.1021/acssensors.8b00340. Epub 2018 Jul 18. Review.
Erratum in: ACS Sens. 2022 Aug 26;7(8):2492. PubMed [citation] PMID: 29978699,
PMCID: PMC6066758

282. Giagulli VA, Campone B, Castellana M, Salzano C, Fisher AD, de Angelis C,
Pivonello R, Colao A, Pasquali D, Maggi M, Triggiani V, On Behalf Of The
Klinefelter ItaliaN Group King. Neuropsychiatric Aspects in Men with Klinefelter
Syndrome. Endocr Metab Immune Disord Drug Targets. 2019;19(2):109-115. doi:

10.2174/1871530318666180703160250. Review. PubMed [citation] PMID: 29972105,
PMCID: PMC7360906

283. Martins Filho ED, Vasconcelos CFM, Oliveira FSC, Pereira ADF, Ferraz ÁAB.
Evaluation of nicotine patch in pain control of patients undergoing laparoscopic
cholecystectomy. Rev Col Bras Cir. 2018 Jun 21;45(3):e1756. doi:
10.1590/0100-6991e-20181756. English, Portuguese. PubMed [citation] PMID:
29947648

284. Davis SM, Lahlou N, Cox-Martin M, Kowal K, Zeitler PS, Ross JL. Oxandrolone
Treatment Results in an Increased Risk of Gonadarche in Prepubertal Boys With
Klinefelter Syndrome. J Clin Endocrinol Metab. 2018 Sep 1;103(9):3449-3455. doi:
10.1210/jc.2018-00682. PubMed [citation] PMID: 29931143, PMCID: PMC6126887

285. Manoharan D, Sharma S, Das CJ, Kumar R, Singh G, Kumar P. Single-Acquisition
Triple-Bolus Dual-Energy CT Protocol for Comprehensive Evaluation of Renal
Masses: A Single-Center Randomized Noninferiority Trial. AJR Am J Roentgenol.
2018 Jul;211(1):W22-W32. doi: 10.2214/AJR.17.18786. Epub 2018 May 24. PubMed
[citation] PMID: 29792728

286. Chen Y, Wang Q, Li J, Jia Y, Yang Q, He T. Triple-rule-out CT angiography using
two axial scans with 16 cm wide-detector for radiation dose reduction. Eur
Radiol. 2018 Nov;28(11):4654-4661. doi: 10.1007/s00330-018-5426-y. Epub 2018 May
22. PubMed [citation] PMID: 29789908

287. Morel A, Peyroux E, Leleu A, Favre E, Franck N, Demily C. Overview of Social
Cognitive Dysfunctions in Rare Developmental Syndromes With Psychiatric
Phenotype. Front Pediatr. 2018 May 3;6:102. doi: 10.3389/fped.2018.00102.
eCollection 2018. Review. PubMed [citation] PMID: 29774207, PMCID: PMC5943552

288. Fentiman IS. The endocrinology of male breast cancer. Endocr

Relat Cancer. 2018
Jun;25(6):R365-R373. doi: 10.1530/ERC-18-0117. Review. PubMed
[citation] PMID:
29752333

289. Watabe T, Koga H. Survival in double aneuploidy involving trisomy 18 and sex
chromosome trisomy: A case report of a 27-month-old child and a review of the
literature. Congenit Anom (Kyoto). 2019 Mar;59(2):43-46. doi: 10.1111/cga.12287.
Epub 2018 May 22. Review. No abstract available. PubMed [citation]
PMID: 29732662

290. Eliveld J, van Wely M, Meißner A, Repping S, van der Veen F, van Pelt AMM. The
risk of TESE-induced hypogonadism: a systematic review and meta-analysis. Hum
Reprod Update. 2018 Jul 1;24(4):442-454. doi: 10.1093/humupd/dmy015. PubMed
[citation] PMID: 29726895, PMCID: PMC6016714

291. Yu Z, Yang J, Liu JH. [Microdissection testicular sperm extraction for patients
with non-mosaic Klinefelter's syndrome: An update]. Zhonghua Nan Ke Xue. 2017
Sep;23(9):842-847. Review. Chinese. PubMed [citation] PMID: 29726668

292. Forbes CM, Flannigan R, Schlegel PN. Spermatogonial stem cell transplantation and
male infertility: Current status and future directions. Arab J Urol. 2017 Dec
27;16(1):171-180. doi: 10.1016/j.aju.2017.11.015. eCollection 2018 Mar. Review.
PubMed [citation] PMID: 29713548, PMCID: PMC5922182

293. Zore T, Palafox M, Reue K. Sex differences in obesity, lipid metabolism, and
inflammation-A role for the sex chromosomes? Mol Metab. 2018 Sep;15:35-44. doi:
10.1016/j.molmet.2018.04.003. Epub 2018 Apr 12. Review. PubMed [citation] PMID:
29706320, PMCID: PMC6066740

294. da Costa Poubel LA, de Gouvea CVD, Calazans FS, Dip EC, Alves WV, Marins SS,
Barcelos R, Barceleiro MO. Pre-operative use of dexamethasone does not reduce
incidence or intensity of bleaching-induced tooth sensitivity. A triple-blind,

parallel-design, randomized clinical trial. Clin Oral Investig. 2019
Jan;23(1):435-444. doi: 10.1007/s00784-018-2452-8. Epub 2018 Apr 25.
PubMed
[citation] PMID: 29696420

295. Flannigan R, Patel P, Paduch DA. Klinefelter Syndrome. The
Effects of Early
Androgen Therapy on Competence and Behavioral Phenotype. Sex Med Rev.
2018
Oct;6(4):595-606. doi: 10.1016/j.sxmr.2018.02.008. Epub 2018 Apr 19.
PubMed
[citation] PMID: 29680294

296. Le Fèvre C, Vigneron C, Schuster H, Walter A, Marcellin L,
Massard G, Lutz P,
Noël G. Metastatic mediastinal mature teratoma with malignant
transformation in a
young man with an adenocarcinoma in a Klinefelter's syndrome: Case
report and
review of the literature. Cancer Radiother. 2018 May;22(3):255-263.
doi:
10.1016/j.canrad.2017.10.006. Epub 2018 Apr 16. Review. PubMed
[citation] PMID:
29673950

297. Toney-Butler TJ, Nicolas S, Wilcox L. Dose Calculation Desired
Over Have Formula
Method. 2023 Jun 20. StatPearls [Internet]. Treasure Island (FL):
StatPearls
Publishing; 2024 Jan-. PubMed [citation] PMID: 29630214

298. Salzano A, D'Assante R, Heaney LM, Monaco F, Rengo G, Valente P,
Pasquali D,
Bossone E, Gianfrilli D, Lenzi A, Cittadini A, Marra AM, Napoli R.
Klinefelter
syndrome, insulin resistance, metabolic syndrome, and diabetes: review
of
literature and clinical perspectives. Endocrine. 2018
Aug;61(2):194-203. doi:
10.1007/s12020-018-1584-6. Epub 2018 Mar 23. Review. PubMed [citation]
PMID:
29572708

299. Krausz C, Cioppi F, Riera-Escamilla A. Testing for genetic
contributions to
infertility: potential clinical impact. Expert Rev Mol Diagn. 2018
Apr;18(4):331-346. doi: 10.1080/14737159.2018.1453358. Epub 2018 Mar
22. Review.
PubMed [citation] PMID: 29540081

300. Schmidt M, Weyer-Elberich V, Hengstler JG, Heimes AS, Almstedt K, Gerhold-Ay A,
Lebrecht A, Battista MJ, Hasenburg A, Sahin U, Kalogeras KT, Kellokumpu-Lehtinen
PL, Fountzilas G, Wirtz RM, Joensuu H. Prognostic impact of CD4-positive T cell
subsets in early breast cancer: a study based on the FinHer trial patient
population. Breast Cancer Res. 2018 Feb 26;20(1):15. doi:
10.1186/s13058-018-0942-x. PubMed [citation] PMID: 29482642, PMCID: PMC5827982

301. Gravholt CH, Chang S, Wallentin M, Fedder J, Moore P, Skakkebæk A. Klinefelter
Syndrome: Integrating Genetics, Neuropsychology, and Endocrinology. Endocr Rev.
2018 Aug 1;39(4):389-423. doi: 10.1210/er.2017-00212. Review. PubMed [citation]
PMID: 29438472

302. Tang YD, Wang W, Yang M, Zhang K, Chen J, Qiao S, Yan H, Wu Y, Huang X, Xu B, Gao
R, Yang Y; CREATIVE Investigators.. Randomized Comparisons of Double-Dose
Clopidogrel or Adjunctive Cilostazol Versus Standard Dual Antiplatelet in
Patients With High Posttreatment Platelet Reactivity: Results of the CREATIVE
Trial. Circulation. 2018 May 22;137(21):2231-2245. doi:
10.1161/CIRCULATIONAHA.117.030190. Epub 2018 Feb 2. PubMed [citation] PMID:
29420189

303. Martin RJ, Smith G, Hughes J, Morrison PJ. Incidence, puberty, and fertility in
45,X/47,XXX mosaicism: Report of a patient and a literature review. Am J Med
Genet A. 2018 Apr;176(4):1029. doi: 10.1002/ajmg.a.38624. Epub 2018 Feb 1.
Review. No abstract available. PubMed [citation] PMID: 29388329

304. Meyer EJ, Wittert G. Endogenous testosterone and mortality risk. Asian J Androl.
2018 Mar-Apr;20(2):115-119. doi: 10.4103/aja.aja_70_17. Review. PubMed [citation]
PMID: 29384142, PMCID: PMC5858093

305. Kanakis GA, Nieschlag E. Klinefelter syndrome: more than hypogonadism.
Metabolism. 2018 Sep;86:135-144. doi: 10.1016/j.metabol.2017.09.017.

Epub 2018
Jan 31. Review. PubMed [citation] PMID: 29382506

306. Skuse D, Printzlau F, Wolstencroft J. Sex chromosome aneuploidies. Handb Clin
Neurol. 2018;147:355-376. doi: 10.1016/B978-0-444-63233-3.00024-5. Review. PubMed
[citation] PMID: 29325624

307. Tabberer M, Lomas DA, Birk R, Brealey N, Zhu CQ, Pascoe S, Locantore N, Lipson
DA. Once-Daily Triple Therapy in Patients with COPD: Patient-Reported Symptoms
and Quality of Life. Adv Ther. 2018 Jan;35(1):56-71. doi:
10.1007/s12325-017-0650-4. Epub 2018 Jan 8. PubMed [citation] PMID: 29313286,
PMCID: PMC5778187

308. Scheuerle AE, Ursini MV. Incontinentia Pigmenti. 1999 Jun 8 [updated 2017 Dec
21]. In: Adam MP, Feldman J, Mirzaa GM, Pagon RA, Wallace SE, Bean LJH, Gripp KW,
Amemiya A, editors. GeneReviews(®) [Internet]. Seattle (WA): University of
Washington, Seattle; 1993-2024. PubMed [citation] PMID: 20301645

309. Liu X, Huang C, Xue L, Xu Q, Xia W, Li X, Miao L. Simultaneous determination of
bentysrepinine (Y101) and its metabolites M8 and M9 in human plasma by UPLC-MS/MS
and its application to a pharmacokinetic study. J Pharm Biomed Anal. 2018 Feb
20;150:287-293. doi: 10.1016/j.jpba.2017.12.010. Epub 2017 Dec 13. PubMed
[citation] PMID: 29258048

310. Akinola OB, Gabriel MO. Neuroanatomical and molecular correlates of cognitive and
behavioural outcomes in hypogonadal males. Metab Brain Dis. 2018
Apr;33(2):491-505. doi: 10.1007/s11011-017-0163-5. Epub 2017 Dec 11. Review.
PubMed [citation] PMID: 29230619

311. Ito S. Study of the Air-Tolerant 1,3-Diphosphacyclobutane-2,4-diyl through the
Direct Arylation. Chem Rec. 2018 Apr;18(4):445-458. doi: 10.1002/tcr.201700052.
Epub 2017 Nov 14. Review. PubMed [citation] PMID: 29134769

312. Badeau M, Lindsay C, Blais J, Nshimyumukiza L, Takwoingi Y,

Langlois S, Légaré F,
Giguère Y, Turgeon AF, Witteman W, Rousseau F. Genomics-based non-invasive
prenatal testing for detection of fetal chromosomal aneuploidy in pregnant women.
Cochrane Database Syst Rev. 2017 Nov 10;11:CD011767. doi:
10.1002/14651858.CD011767.pub2. Review. PubMed [citation] PMID: 29125628, PMCID:
PMC6486016

313. Stamou MI, Georgopoulos NA. Kallmann syndrome: phenotype and genotype of
hypogonadotropic hypogonadism. Metabolism. 2018 Sep;86:124-134. doi:
10.1016/j.metabol.2017.10.012. Epub 2017 Nov 3. Review. PubMed [citation] PMID:
29108899, PMCID: PMC5934335

314. Stage TB, Graff M, Wong S, Rasmussen LL, Nielsen F, Pottegård A, Brøsen K, Kroetz
DL, Khojasteh SC, Damkier P. Dicloxacillin induces CYP2C19, CYP2C9 and CYP3A4 in
vivo and in vitro. Br J Clin Pharmacol. 2018 Mar;84(3):510-519. doi:
10.1111/bcp.13467. Epub 2018 Jan 10. PubMed [citation] PMID: 29105855, PMCID:
PMC5809358

315. Hamel J. [Corrective procedures and indications for cavovarus foot deformities in
children and adolescents]. Oper Orthop Traumatol. 2017 Dec;29(6):473-482. doi:
10.1007/s00064-017-0520-x. Epub 2017 Oct 25. Review. German. PubMed [citation]
PMID: 29071376

316. Barg A, Ruiz R, Hintermann B. [Triple arthrodesis for correction of cavovarus
deformity]. Oper Orthop Traumatol. 2017 Dec;29(6):461-472. doi:
10.1007/s00064-017-0519-3. Epub 2017 Oct 20. Review. German. PubMed [citation]
PMID: 29052742

317. Riffaud J, Cassette P, Lacour D, Lourenço V, Tartès I, Kellett MA, Corbel M, Lépy
MC, Domergue C, Destouches C, Carcreff H, Vigneau O. Measurement of absolute K
X-ray emission intensities in the decay of (103m)Rh. Appl Radiat Isot. 2018
Apr;134:399-405. doi: 10.1016/j.apradiso.2017.10.003. Epub 2017 Oct 4. Review.
PubMed [citation] PMID: 29031491

318. Shinagare AB, Somarouthu B, Guo H, Tolaney SM, Ramaiya NH. Occurrence and
significance of morphologic changes in patients with metastatic triple negative
breast cancer treated with Cabozantinib. Clin Imaging. 2018 Mar - Apr;48:44-47.
doi: 10.1016/j.clinimag.2017.09.014. Epub 2017 Sep 28. PubMed [citation] PMID:
29028513

319. Oud MS, Ramos L, O'Bryan MK, McLachlan RI, Okutman Ö, Viville S, de Vries PF,
Smeets DFCM, Lugtenberg D, Hehir-Kwa JY, Gilissen C, van de Vorst M, Vissers
LELM, Hoischen A, Meijerink AM, Fleischer K, Veltman JA, Noordam MJ. Validation
and application of a novel integrated genetic screening method to a cohort of
1,112 men with idiopathic azoospermia or severe oligozoospermia. Hum Mutat. 2017
Nov;38(11):1592-1605. doi: 10.1002/humu.23312. Epub 2017 Sep 6. PubMed [citation]
PMID: 28801929

320. O'Donovan R, Völlm B. Klinefelter's syndrome and sexual offending - A literature
review. Crim Behav Ment Health. 2018 Apr;28(2):132-140. doi: 10.1002/cbm.2052.
Epub 2017 Aug 7. Review. PubMed [citation] PMID: 28782868

321. Krishnan V, Chawla A, Sharbidre KG, Peh WCG. Current Techniques and Clinical
Applications of Computed Tomography Urography. Curr Probl Diagn Radiol. 2018 Jul
- Aug;47(4):245-256. doi: 10.1067/j.cpradiol.2017.07.002. Epub 2017 Jul 8.
Review. PubMed [citation] PMID: 28774661

322. Treibel TA, Rossi A, Pugliese F, Davies LC. Functional assessment of coronary
artery disease by cardiac computed tomography. Expert Rev Cardiovasc Ther. 2017
Sep;15(9):657-665. doi: 10.1080/14779072.2017.1359087. Epub 2017 Jul 25. Review.
PubMed [citation] PMID: 28728443

323. Crawford D, Dearmun A. Klinefelter syndrome. Nurs Child Young People. 2017 Jul
10;29(6):19. doi: 10.7748/ncyp.29.6.19.s21. Review. PubMed [citation]

PMID:
28691621

324. Morel A, Demily C. [Social cognition in children with neurogenetic syndromes: A
literature review]. Arch Pediatr. 2017 Aug;24(8):757-765. doi:
10.1016/j.arcped.2017.05.006. Epub 2017 Jun 28. Review. French. PubMed [citation]
PMID: 28668215

325. Close S, Talboy A, Fennoy I. Complexities of Care in Klinefelter Syndrome: An
APRN Perspective. Pediatr Endocrinol Rev. 2017 Jun;14(Suppl 2):462-471. doi:
10.17458/per.vol14.2017.ctf.complexitiescareklinefelter. Review. PubMed
[citation] PMID: 28647951

326. de Sanctis V, Soliman AT, Elsedfy H, Soliman N, Elalaily R, Di Maio S. Is the
Second to Fourth Digit Ratio (2D:4D) a Biomarker of Sex-Steroids Activity?
Pediatr Endocrinol Rev. 2017 Jun;14(4):378-386. doi:
10.17458/per.vol14.2017.SSE.SexSteroids. Review. PubMed [citation]
PMID: 28613048

327. Weintraub A, Eldar-Geva T. Anti-Mullerian Hormone (AMH) Determinations in the
Pediatric and Adolescent Endocrine Practice. Pediatr Endocrinol Rev. 2017
Jun;14(4):364-370. doi: 10.17458/per.vol14.2017.WG.Mullerian. Review. PubMed
[citation] PMID: 28613046

328. Röpke A, Tüttelmann F. MECHANISMS IN ENDOCRINOLOGY: Aberrations of the X
chromosome as cause of male infertility. Eur J Endocrinol. 2017
Nov;177(5):R249-R259. doi: 10.1530/EJE-17-0246. Epub 2017 Jun 13. Review. PubMed
[citation] PMID: 28611019

329. Sundararajan S, Carter YM. Mediastinal Nonseminoma. 2023 Jun 12. StatPearls
[Internet]. Treasure Island (FL): StatPearls Publishing; 2024 Jan-. PubMed
[citation] PMID: 28613751

330. Flannigan R, Schlegel PN. Genetic diagnostics of male infertility in clinical
practice. Best Pract Res Clin Obstet Gynaecol. 2017 Oct;44:26-37. doi:

10.1016/j.bpobgyn.2017.05.002. Epub 2017 May 10. Review. PubMed [citation] PMID:
28601348

331. Masuda N, Lee SJ, Ohtani S, Im YH, Lee ES, Yokota I, Kuroi K, Im SA, Park BW, Kim
SB, Yanagita Y, Ohno S, Takao S, Aogi K, Iwata H, Jeong J, Kim A, Park KH, Sasano
H, Ohashi Y, Toi M. Adjuvant Capecitabine for Breast Cancer after Preoperative
Chemotherapy. N Engl J Med. 2017 Jun 1;376(22):2147-2159. doi:
10.1056/NEJMoa1612645. PubMed [citation] PMID: 28564564

332. Gutierrez-Solana LG. [Novel therapies in neurometabolic diseases: the importance
of early intervention]. Rev Neurol. 2017 May 17;64(s03):S25-S28. Review. Spanish.
PubMed [citation] PMID: 28524215

333. Plontke SK. [Diagnostics and Therapy of Idiopathic Sudden Sensorineural Hearing
Loss]. Laryngorhinootologie. 2017 Apr;96(S 01):S103-S122. doi:
10.1055/s-0042-122385. Epub 2017 May 12. Review. German. PubMed [citation] PMID:
28499297

334. Coutant R, Donzeau A, Decrequy A, Louvigné M, Bouhours-Nouet N. How to
investigate a child with excessive growth? Ann Endocrinol (Paris). 2017
Jun;78(2):98-103. doi: 10.1016/j.ando.2017.04.006. Epub 2017 May 5. Review.
PubMed [citation] PMID: 28483364

335. Cabrejas Gómez MDC, Fuentes Gómez C, Pérez García L, González Cabrera N, Diez
Lopez I. Mosaicism XXY/X0. Endocrinol Diabetes Nutr. 2017 Feb;64(2):118-119. doi:
10.1016/j.endinu.2016.11.005. Epub 2017 Feb 1. Review. English, Spanish. No
abstract available. PubMed [citation] PMID: 28440776

336. Corona G, Pizzocaro A, Lanfranco F, Garolla A, Pelliccione F, Vignozzi L, Ferlin
A, Foresta C, Jannini EA, Maggi M, Lenzi A, Pasquali D, Francavilla S;
Klinefelter ItaliaN Group (KING).. Sperm recovery and ICSI outcomes in
Klinefelter syndrome: a systematic review and meta-analysis. Hum Reprod Update.
2017 May 1;23(3):265-275. doi: 10.1093/humupd/dmx008. Review. PubMed [citation]

PMID: 28379559

337. Samango-Sprouse C, Keen C, Sadeghin T, Gropman A. The benefits and limitations of
cell-free DNA screening for 47, XXY (Klinefelter syndrome). Prenat Diagn. 2017
May;37(5):497-501. doi: 10.1002/pd.5044. Epub 2017 Apr 17. Review. PubMed
[citation] PMID: 28346690

338. Drake AL, Unger JA, Ronen K, Matemo D, Perrier T, DeRenzi B, Richardson BA,
Kinuthia J, John-Stewart G. Evaluation of mHealth strategies to optimize
adherence and efficacy of Option B+ prevention of mother-to-child HIV
transmission: Rationale, design and methods of a 3-armed randomized controlled
trial. Contemp Clin Trials. 2017 Jun;57:44-50. doi: 10.1016/j.cct.2017.03.007.
Epub 2017 Mar 14. PubMed [citation] PMID: 28315480, PMCID: PMC5522580

339. Ross JL, Kushner H, Kowal K, Bardsley M, Davis S, Reiss AL, Tartaglia N, Roeltgen
D. Androgen Treatment Effects on Motor Function, Cognition, and Behavior in Boys
with Klinefelter Syndrome. J Pediatr. 2017 Jun;185:193-199.e4. doi:
10.1016/j.jpeds.2017.02.036. Epub 2017 Mar 10. PubMed [citation] PMID: 28285751,
PMCID: PMC6754744

340. Reue K. Sex differences in obesity: X chromosome dosage as a risk factor for
increased food intake, adiposity and co-morbidities. Physiol Behav. 2017 Jul
1;176:174-182. doi: 10.1016/j.physbeh.2017.02.040. Epub 2017 Mar 8. Review.
PubMed [citation] PMID: 28284880, PMCID: PMC5444325

341. Albuquerque EVA, Scalco RC, Jorge AAL. MANAGEMENT OF ENDOCRINE DISEASE:
Diagnostic and therapeutic approach of tall stature. Eur J Endocrinol. 2017
Jun;176(6):R339-R353. doi: 10.1530/EJE-16-1054. Epub 2017 Mar 8. Review. PubMed
[citation] PMID: 28274950

342. Calogero AE, Giagulli VA, Mongioì LM, Triggiani V, Radicioni AF, Jannini EA,
Pasquali D; Klinefelter ItaliaN Group (KING).. Klinefelter syndrome:
cardiovascular abnormalities and metabolic disorders. J Endocrinol

Invest. 2017
Jul;40(7):705-712. doi: 10.1007/s40618-017-0619-9. Epub 2017 Mar 3.
Review.
PubMed [citation] PMID: 28258556

343. Kovacs CS. The Skeleton Is a Storehouse of Mineral That Is
Plundered During
Lactation and (Fully?) Replenished Afterwards. J Bone Miner Res. 2017
Apr;32(4):676-680. doi: 10.1002/jbmr.3090. Epub 2017 Feb 28. Review.
PubMed
[citation] PMID: 28177150

344. Demily C, Poisson A, Peyroux E, Gatellier V, Nicolas A, Rigard C,
Schluth-Bolard
C, Sanlaville D, Rossi M. Autism spectrum disorder associated with
49,XYYYY: case
report and review of the literature. BMC Med Genet. 2017 Jan
31;18(1):9. doi:
10.1186/s12881-017-0371-1. Review. PubMed [citation] PMID: 28137251,
PMCID:
PMC5282903

345. Margery-Muir AA, Bundell C, Nelson D, Groth DM, Wetherall JD.
Gender balance in
patients with systemic lupus erythematosus. Autoimmun Rev. 2017
Mar;16(3):258-268. doi: 10.1016/j.autrev.2017.01.007. Epub 2017 Jan
27. Review.
PubMed [citation] PMID: 28137478

346. Bella J, Hulmes DJ. Fibrillar Collagens. Subcell Biochem.
2017;82:457-490. doi:
10.1007/978-3-319-49674-0_14. Review. PubMed [citation] PMID: 28101870

347. Squire JM, Parry DA. Fibrous Protein Structures: Hierarchy,
History and Heroes.
Subcell Biochem. 2017;82:1-33. doi: 10.1007/978-3-319-49674-0_1.
Review. PubMed
[citation] PMID: 28101857

348. Maese L, Li KD, Xu X, Afify Z, Paxton CN, Putnam A. Acute
Leukemia and Concurrent
Mediastinal Germ Cell Tumor: Case Report and Literature Review. Fetal
Pediatr
Pathol. 2017 Apr;36(2):168-176. doi: 10.1080/15513815.2016.1273983.
Epub 2017 Jan
19. Review. PubMed [citation] PMID: 28103116

349. Stöger L, Schaefer-Prokop C, Geurts BH. Imaging of nontraumatic
thoracic
emergencies. Curr Opin Pulm Med. 2017 Mar;23(2):184-192. doi:

10.1097/MCP.0000000000000355. Review. PubMed [citation] PMID: 28009644

350. Giagulli VA, Guastamacchia E, Licchelli B, Triggiani V. Serum Testosterone and
Cognitive Function in Ageing Male: Updating the Evidence. Recent Pat Endocr Metab
Immune Drug Discov. 2016;10(1):22-30. Review. PubMed [citation] PMID: 27981914

351. de la Mora Cervantes R, Dennie C. Triple rule-out cardiac computed tomography: is
it finally a reality? Minerva Cardioangiol. 2017 Jun;65(3):225-234. doi:
10.23736/S0026-4725.16.04272-9. Epub 2016 Dec 13. Review. PubMed [citation] PMID:
27958691

352. Hoshino M, Ohtawa J, Akitsu K. Effects of the addition of tiotropium on airway
dimensions in symptomatic asthma. Allergy Asthma Proc. 2016 Nov;37(6):147-153.
PubMed [citation] PMID: 27931291

353. Van Batavia JP, Kolon TF. Fertility in disorders of sex development: A review. J
Pediatr Urol. 2016 Dec;12(6):418-425. doi: 10.1016/j.jpurol.2016.09.015. Epub
2016 Nov 3. Review. PubMed [citation] PMID: 27856173

354. Davis SM, Cox-Martin MG, Bardsley MZ, Kowal K, Zeitler PS, Ross JL. Effects of
Oxandrolone on Cardiometabolic Health in Boys With Klinefelter Syndrome: A
Randomized Controlled Trial. J Clin Endocrinol Metab. 2017 Jan 1;102(1):176-184.
doi: 10.1210/jc.2016-2904. PubMed [citation] PMID: 27802097, PMCID: PMC5413098

355. Fish AM, Cachia A, Fischer C, Mankiw C, Reardon PK, Clasen LS, Blumenthal JD,
Greenstein D, Giedd JN, Mangin JF, Raznahan A. Influences of Brain Size, Sex, and
Sex Chromosome Complement on the Architecture of Human Cortical Folding. Cereb
Cortex. 2017 Dec 1;27(12):5557-5567. doi: 10.1093/cercor/bhw323. PubMed
[citation] PMID: 27799275, PMCID: PMC6075547

356. Schliemann C, Gerss J, Wiebe S, Mikesch JH, Knoblauch N, Sauer T, Angenendt L,

Kewitz T, Urban M, Butterfass-Bahloul T, Edemir S, Vehring K, Müller-Tidow C,
Berdel WE, Krug U. A Phase I Dose Escalation Study of the Triple Angiokinase
Inhibitor Nintedanib Combined with Low-Dose Cytarabine in Elderly Patients with
Acute Myeloid Leukemia. PLoS One. 2016 Oct 7;11(10):e0164499. doi:
10.1371/journal.pone.0164499. eCollection 2016. PubMed [citation] PMID: 27716819,
PMCID: PMC5055288

357. Bonomi M, Rochira V, Pasquali D, Balercia G, Jannini EA, Ferlin A; Klinefelter
ItaliaN Group (KING).. Klinefelter syndrome (KS): genetics, clinical phenotype
and hypogonadism. J Endocrinol Invest. 2017 Feb;40(2):123-134. doi:
10.1007/s40618-016-0541-6. Epub 2016 Sep 19. Review. PubMed [citation] PMID:
27644703, PMCID: PMC5269463

358. Breman AM, Chow JC, U'Ren L, Normand EA, Qdaisat S, Zhao L, Henke DM, Chen R,
Shaw CA, Jackson L, Yang Y, Vossaert L, Needham RH, Chang EJ, Campton D, Werbin
JL, Seubert RC, Van den Veyver IB, Stilwell JL, Kaldjian EP, Beaudet AL. Evidence
for feasibility of fetal trophoblastic cell-based noninvasive prenatal testing.
Prenat Diagn. 2016 Nov;36(11):1009-1019. doi: 10.1002/pd.4924. Epub 2016 Oct 2.
PubMed [citation] PMID: 27616633, PMCID: PMC5129580

359. Caffarelli C, Santamaria F, Di Mauro D, Mastrorilli C, Mirra V, Bernasconi S.
Progress in pediatrics in 2015: choices in allergy, endocrinology,
gastroenterology, genetics, haematology, infectious diseases, neonatology,
nephrology, neurology, nutrition, oncology and pulmonology. Ital J Pediatr. 2016
Aug 27;42(1):75. doi: 10.1186/s13052-016-0288-x. Review. PubMed [citation] PMID:
27566421, PMCID: PMC5002164

360. Chen Q, Wen J, Li H, Xu Y, Liu F, Sun S. Recent advances in different modal
imaging-guided photothermal therapy. Biomaterials. 2016 Nov;106:144-66. doi:
10.1016/j.biomaterials.2016.08.022. Epub 2016 Aug 16. Review. PubMed [citation]
PMID: 27561885

361. Slama R, Long B, Koyfman A. The emergency medicine approach to abdominal vascular
graft complications. Am J Emerg Med. 2016 Oct;34(10):2014-2017. doi:
10.1016/j.ajem.2016.07.001. Epub 2016 Jul 4. Review. PubMed [citation] PMID:
27519454

362. Suson KD. Transitional Urology for Male Adolescents: What Adult Urologists Should
Know. Curr Urol Rep. 2016 Oct;17(10):71. doi: 10.1007/
s11934-016-0630-2. Review.
PubMed [citation] PMID: 27517862

363. Oates R. Adolescent Klinefelter syndrome: is there an advantage to testis tissue
harvesting or not? F1000Res. 2016 Jul 6;5. pii: F1000 Faculty Rev-1595. doi:
10.12688/f1000research.8395.1. eCollection 2016. Review. PubMed [citation] PMID:
27441089, PMCID: PMC4937818

364. Rau RE, Carroll AJ, Heerema NA, Arland L, Carroll WL, Winick NJ, Raetz EA, Loh
ML, Yang W, Relling MV, Dai Y, Devidas M, Hunger SP. Klinefelter syndrome and
47,XYY syndrome in children with B cell acute lymphoblastic leukaemia. Br J
Haematol. 2017 Dec;179(5):843-846. doi: 10.1111/bjh.14258. Epub 2016 Jul 19.
Review. No abstract available. PubMed [citation] PMID: 27434379, PMCID:
PMC5247399

365. Davis S, Howell S, Wilson R, Tanda T, Ross J, Zeitler P, Tartaglia N. Advances in
the Interdisciplinary Care of Children with Klinefelter Syndrome. Adv Pediatr.
2016 Aug;63(1):15-46. doi: 10.1016/j.yapd.2016.04.020. Review. No abstract
available. PubMed [citation] PMID: 27426894, PMCID: PMC5340500

366. Franik S, Hoeijmakers Y, D'Hauwers K, Braat DD, Nelen WL, Smeets D, Claahsen-van
der Grinten HL, Ramos L, Fleischer K. Klinefelter syndrome and fertility: sperm
preservation should not be offered to children with Klinefelter syndrome. Hum
Reprod. 2016 Sep;31(9):1952-9. doi: 10.1093/humrep/dew179. Epub 2016 Jul 13.

Review. PubMed [citation] PMID: 27412247

367. Tournaye H, Krausz C, Oates RD. Concepts in diagnosis and therapy for male
reproductive impairment. Lancet Diabetes Endocrinol. 2017 Jul;5(7):554-564. doi:
10.1016/S2213-8587(16)30043-2. Epub 2016 Jul 7. Review. PubMed [citation] PMID:
27395770

368. Zahedi AR, Lüring C, Janßen D. [Tönnis and Kalchschmidt triple pelvic osteotomy].
Orthopade. 2016 Aug;45(8):673-7. doi: 10.1007/s00132-016-3291-4. Review. German.
PubMed [citation] PMID: 27385385

369. Sudah M, Masarwah A, Kainulainen S, Pitkänen M, Matikka H, Dabravolskaite V,
Aaltomaa S, Vanninen R. Comprehensive MR Urography Protocol: Equally Good
Diagnostic Performance and Enhanced Visibility of the Upper Urinary Tract
Compared to Triple-Phase CT Urography. PLoS One. 2016 Jul 6;11(7):e0158673. doi:
10.1371/journal.pone.0158673. eCollection 2016. PubMed [citation] PMID: 27384417,
PMCID: PMC4934766

370. Belli S, Santi D, Leoni E, Dall'Olio E, Fanelli F, Mezzullo M, Pelusi C, Roli L,
Tagliavini S, Trenti T, Granata AR, Pagotto U, Pasquali R, Rochira V, Carani C,
Simoni M. Human chorionic gonadotropin stimulation gives evidence of differences
in testicular steroidogenesis in Klinefelter syndrome, as assessed by liquid
chromatography-tandem mass spectrometry. Eur J Endocrinol. 2016
Jun;174(6):801-11. doi: 10.1530/EJE-15-1224. PubMed [citation] PMID:
27188454

371. Franco G, Scarselli F, Casciani V, De Nunzio C, Dente D, Leonardo C, Greco PF,
Greco A, Minasi MG, Greco E. A novel stepwise micro-TESE approach in non
obstructive azoospermia. BMC Urol. 2016 May 12;16(1):20. doi:
10.1186/s12894-016-0138-6. PubMed [citation] PMID: 27176005, PMCID:
PMC4866333

372. Crespo M, Navarro J, Martinez-Rebollar M, Podzamczer D, Domingo P, Mallolas J,

Saumoy M, Mateo GM, Curran A, Gatell J, Ribera E. Improvement of BMD after
Switching from Lopinavir/R Plus Two Nucleos(T)ide Reverse Transcriptase
Inhibitors to Lopinavir/R Plus Lamivudine: OLE-LIP Substudy. HIV Clin Trials.
2016 May;17(3):89-95. doi: 10.1080/15284336.2016.1149929. Epub 2016 Mar 16.
PubMed [citation] PMID: 27125363

373. Yki-Järvinen H. Diagnosis of non-alcoholic fatty liver disease (NAFLD).
Diabetologia. 2016 Jun;59(6):1104-11. doi: 10.1007/s00125-016-3944-1. Epub 2016
Apr 18. Review. PubMed [citation] PMID: 27091184

374. Bella J. Collagen structure: new tricks from a very old dog. Biochem J. 2016 Apr
15;473(8):1001-25. doi: 10.1042/BJ20151169. Review. PubMed [citation] PMID:
27060106

375. Song SH, Chiba K, Ramasamy R, Lamb DJ. Recent advances in the genetics of
testicular failure. Asian J Androl. 2016 May-Jun;18(3):350-5. doi:
10.4103/1008-682X.178857. Review. PubMed [citation] PMID: 27048782, PMCID:
PMC4854078

376. Rajapaksa G, Thomas C, Gustafsson JÅ. Estrogen signaling and unfolded protein
response in breast cancer. J Steroid Biochem Mol Biol. 2016 Oct;163:45-50. doi:
10.1016/j.jsbmb.2016.03.036. Epub 2016 Apr 1. Review. PubMed [citation] PMID:
27045680

377. Kingery SE, Wintergerst KA. Turner Syndrome and Klinefelter Syndrome. Adolesc Med
State Art Rev. 2015 Aug;26(2):411-27. Review. No abstract available. PubMed
[citation] PMID: 26999880

378. Lehner A, Schecklmann M, Greenlee MW, Rupprecht R, Langguth B. Triple-site rTMS
for the treatment of chronic tinnitus: a randomized controlled trial. Sci Rep.
2016 Mar 1;6:22302. doi: 10.1038/srep22302. PubMed [citation] PMID: 26927363,
PMCID: PMC4772792

379. Pradhan D, Kaman L, Dhillon J, Mohanty SK. Mediastinal mixed germ cell tumor in
an infertile male with Klinefelter syndrome:A case report and literature review.
J Cancer Res Ther. 2015 Oct-Dec;11(4):1034. doi: 10.4103/0973-1482.150697.
Review. PubMed [citation] PMID: 26881632

380. Salzano A, Arcopinto M, Marra AM, Bobbio E, Esposito D, Accardo G, Giallauria F,
Bossone E, Vigorito C, Lenzi A, Pasquali D, Isidori AM, Cittadini A. Klinefelter
syndrome, cardiovascular system, and thromboembolic disease: review of literature
and clinical perspectives. Eur J Endocrinol. 2016 Jul;175(1):R27-40. doi:
10.1530/EJE-15-1025. Epub 2016 Feb 5. Review. PubMed [citation] PMID: 26850445

381. Bird RJ, Hurren BJ. Anatomical and clinical aspects of Klinefelter's syndrome.
Clin Anat. 2016 Jul;29(5):606-19. doi: 10.1002/ca.22695. Epub 2016 Feb 21.
Review. PubMed [citation] PMID: 26823086

382. Sroubek J, Probst V, Mazzanti A, Delise P, Hevia JC, Ohkubo K, Zorzi A, Champagne
J, Kostopoulou A, Yin X, Napolitano C, Milan DJ, Wilde A, Sacher F, Borggrefe M,
Ellinor PT, Theodorakis G, Nault I, Corrado D, Watanabe I, Antzelevitch C,
Allocca G, et al. Programmed Ventricular Stimulation for Risk Stratification in
the Brugada Syndrome: A Pooled Analysis. Circulation. 2016 Feb 16;133(7):622-30.
doi: 10.1161/CIRCULATIONAHA.115.017885. Epub 2016 Jan 21. Review. PubMed
[citation] PMID: 26797467, PMCID: PMC4758872

383. Nahata L, Yu RN, Paltiel HJ, Chow JS, Logvinenko T, Rosoklija I, Cohen LE. Sperm
Retrieval in Adolescents and Young Adults with Klinefelter Syndrome: A
Prospective, Pilot Study. J Pediatr. 2016 Mar;170:260-5.e1-2. doi:
10.1016/j.jpeds.2015.12.028. Epub 2015 Dec 31. PubMed [citation] PMID: 26746120

384. Gies I, Oates R, De Schepper J, Tournaye H. Testicular biopsy and
cryopreservation for fertility preservation of prepubertal boys with Klinefelter

syndrome: a pro/con debate. Fertil Steril. 2016 Feb;105(2):249-55.
doi:
10.1016/j.fertnstert.2015.12.011. Epub 2015 Dec 31. Review. PubMed
[citation]
PMID: 26748226

385. Schütte P, Möricke A, Zimmermann M, Bleckmann K, Reismüller B,
Attarbaschi A,
Mann G, Bodmer N, Niggli F, Schrappe M, Stanulla M, Kratz CP.
Preexisting
conditions in pediatric ALL patients: Spectrum, frequency and clinical
impact.
Eur J Med Genet. 2016 Mar;59(3):143-51. doi: 10.1016/
j.ejmg.2015.12.008. Epub
2015 Dec 28. Review. PubMed [citation] PMID: 26732628

386. Chang S, Skakkebæk A, Gravholt CH. Klinefelter Syndrome and
medical treatment:
hypogonadism and beyond. Hormones (Athens). 2015 Oct-Dec;14(4):531-48.
doi:
10.14310/horm.2002.1622. Review. PubMed [citation] PMID: 26732150

387. Rabant M, Amrouche L, Morin L, Bonifay R, Lebreton X, Aouni L,
Benon A, Sauvaget
V, Le Vaillant L, Aulagnon F, Sberro R, Snanoudj R, Mejean A, Legendre
C, Terzi
F, Anglicheau D. Early Low Urinary CXCL9 and CXCL10 Might Predict
Immunological
Quiescence in Clinically and Histologically Stable Kidney Recipients.
Am J
Transplant. 2016 Jun;16(6):1868-81. doi: 10.1111/ajt.13677. Epub 2016
Mar 10.
PubMed [citation] PMID: 26694099

388. Ferzoco RM, Ruddy KJ. The Epidemiology of Male Breast Cancer.
Curr Oncol Rep.
2016 Jan;18(1):1. doi: 10.1007/s11912-015-0487-4. Review. PubMed
[citation] PMID:
26694922

389. Hoffmann U, Akers SR, Brown RK, Cummings KW, Cury RC, Greenberg
SB, Ho VB, Hsu
JY, Min JK, Panchal KK, Stillman AE, Woodard PK, Jacobs JE. ACR
Appropriateness
Criteria Acute Nonspecific Chest Pain-Low Probability of Coronary
Artery Disease.
J Am Coll Radiol. 2015 Dec;12(12 Pt A):1266-71. doi: 10.1016/
j.jacr.2015.09.004.
Review. Erratum in: J Am Coll Radiol. 2016 Feb;13(2):231. PubMed
[citation] PMID:

26653833

390. Muhammad S, Güresir Á, Greschus S, Scorzin J, Vatter H, Güresir E. Posterior
Reversible Encephalopathy Syndrome as an Overlooked Complication of Induced
Hypertension for Cerebral Vasospasm: Systematic Review and Illustrative Case.
Stroke. 2016 Feb;47(2):519-22. doi: 10.1161/STROKEAHA.115.011697. Epub 2015 Dec
1. Review. PubMed [citation] PMID: 26628389

391. Olsson KM, Palazzini M. Challenges in pulmonary hypertension: managing the
unexpected. Eur Respir Rev. 2015 Dec;24(138):674-81. doi:
10.1183/16000617.0060-2015. Review. PubMed [citation] PMID: 26621981, PMCID:
PMC9487621

392. Kiełbowicz Z, Piątek A, Bieżyński J, Skrzypczak P, Kuropka P, Kuryszko J, Nikodem
A, Kafarski P, Pezowicz C. The experimental osteoporosis in sheep--clinical
approach. Pol J Vet Sci. 2015;18(3):645-54. doi: 10.1515/pjvs-2015-0083. PubMed
[citation] PMID: 26618599

393. Christe N, Meier CA. Hypotestosteronaemia in the aging male: should we treat it?
Swiss Med Wkly. 2015 Nov 24;145:w14216. doi: 10.4414/smw.2015.14216. eCollection
2015. Review. PubMed [citation] PMID: 26599486

394. Davis SM, Rogol AD, Ross JL. Testis Development and Fertility Potential in Boys
with Klinefelter Syndrome. Endocrinol Metab Clin North Am. 2015 Dec;44(4):843-65.
doi: 10.1016/j.ecl.2015.07.008. Epub 2015 Sep 28. Review. PubMed [citation] PMID:
26568497, PMCID: PMC4648691

395. Kinsella S, Murphy K, Breen M, O'Neill S, McLaughlin P, Coyle J, Bogue C, O'Neill
F, Moore N, McGarrigle A, Molloy MG, Maher MM, Eustace JA. Comparison of single
CT scan assessment of bone mineral density, vascular calcification and fat mass
with standard clinical measurements in renal transplant subjects: the ABC HeART
study. BMC Nephrol. 2015 Nov 11;16:188. doi: 10.1186/

s12882-015-0182-6. PubMed
[citation] PMID: 26558994, PMCID: PMC4642694

396. Chen F, Dai Z, Kang Y, Lv G, Keller ET, Jiang Y. Effects of zoledronic acid on
bone fusion in osteoporotic patients after lumbar fusion. Osteoporos Int. 2016
Apr;27(4):1469-1476. doi: 10.1007/s00198-015-3398-1. Epub 2015 Nov 10. PubMed
[citation] PMID: 26556733, PMCID: PMC4792656

397. Wang L, Meng Q, Tang X, Yin T, Zhang J, Yang S, Wang X, Wu H, Shi Q, Jenkins EC,
Zhong N, Gu Y. Maternal mosaicism of sex chromosome causes discordant sex
chromosomal aneuploidies associated with noninvasive prenatal testing. Taiwan J
Obstet Gynecol. 2015 Oct;54(5):527-31. doi: 10.1016/j.tjog.2014.10.009. PubMed
[citation] PMID: 26522104

398. Volc S, Schanz S. [Andrologically relevant findings from the perspective of the
dermatologist]. Hautarzt. 2015 Dec;66(12):892-7. doi: 10.1007/s00105-015-3712-z.
Review. German. PubMed [citation] PMID: 26487495

399. Barat-Houari M, Sarrabay G, Gatinois V, Fabre A, Dumont B, Genevieve D, Touitou
I. Mutation Update for COL2A1 Gene Variants Associated with Type II
Collagenopathies. Hum Mutat. 2016 Jan;37(1):7-15. doi: 10.1002/humu.22915. Epub
2015 Oct 21. Review. PubMed [citation] PMID: 26443184

400. Everett JK, Tejero R, Murthy SB, Acton TB, Aramini JM, Baran MC, Benach J, Cort
JR, Eletsky A, Forouhar F, Guan R, Kuzin AP, Lee HW, Liu G, Mani R, Mao B, Mills
JL, Montelione AF, Pederson K, Powers R, Ramelot T, Rossi P, et al. A community
resource of experimental data for NMR / X-ray crystal structure pairs. Protein
Sci. 2016 Jan;25(1):30-45. doi: 10.1002/pro.2774. Epub 2015 Sep 22. Review.
PubMed [citation] PMID: 26293815, PMCID: PMC4815321

401. Fedder J, Gravholt CH, Kristensen SG, Marcussen N, Engvad B, Milton AM, Andersen
CY. Testicular Sperm Sampling by Subcapsular Orchiectomy in Klinefelter Patients:

A New Simplified Treatment Approach. Urology. 2015 Oct;86(4):744-50.
doi:
10.1016/j.urology.2015.06.044. Epub 2015 Aug 4. PubMed [citation]
PMID: 26254174

402. Yuan SM. Postperfusion lung syndrome: Respiratory mechanics,
respiratory indices
and biomarkers. Ann Thorac Med. 2015 Jul-Sep;10(3):151-7. doi:
10.4103/1817-1737.150736. Review. PubMed [citation] PMID: 26229556,
PMCID:
PMC4518344

403. Maqdasy S, Bogenmann L, Batisse-Lignier M, Roche B, Franck F,
Desbiez F, Tauveron
I. Leydig cell tumor in a patient with 49,XXXXY karyotype: a review of
literature. Reprod Biol Endocrinol. 2015 Jul 10;13:72. doi:
10.1186/s12958-015-0071-7. Review. PubMed [citation] PMID: 26160035,
PMCID:
PMC4496935

404. Barnett CP, van Bon BW. Monogenic and chromosomal causes of
isolated speech and
language impairment. J Med Genet. 2015 Nov;52(11):719-29. doi:
10.1136/jmedgenet-2015-103161. Epub 2015 Jul 2. Review. PubMed
[citation] PMID:
26139234

405. Godler DE, Inaba Y, Schwartz CE, Bui QM, Shi EZ, Li X, Herlihy
AS, Skinner C,
Hagerman RJ, Francis D, Amor DJ, Metcalfe SA, Hopper JL, Slater HR.
Detection of
skewed X-chromosome inactivation in Fragile X syndrome and X
chromosome
aneuploidy using quantitative melt analysis. Expert Rev Mol Med. 2015
Jul
1;17:e13. doi: 10.1017/erm.2015.11. Review. PubMed [citation] PMID:
26132880,
PMCID: PMC4836209

406. Barstow C, Rerucha C. Evaluation of Short and Tall Stature in
Children. Am Fam
Physician. 2015 Jul 1;92(1):43-50. Review. PubMed [citation] PMID:
26132126

407. TARDIS Trial Investigators., Krishnan K, Beridze M, Christensen
H, Dineen R,
Duley L, Heptinstall S, James M, Markus HS, Pocock S, Ranta A,
Robinson T, Nikola
N, Venables G, Bath P. Safety and efficacy of intensive vs. guideline
antiplatelet therapy in high-risk patients with recent ischemic stroke

or
transient ischemic attack: rationale and design of the Triple Antiplatelets for
Reducing Dependency after Ischaemic Stroke (TARDIS) trial (ISRCTN47823388). Int J
Stroke. 2015 Oct;10(7):1159-65. doi: 10.1111/ijs.12538. Epub 2015 Jun 16. PubMed
[citation] PMID: 26079743, PMCID: PMC4855643

408. Zacharin M. Pubertal induction in hypogonadism: Current approaches including use
of gonadotrophins. Best Pract Res Clin Endocrinol Metab. 2015 Jun;29(3):367-83.
doi: 10.1016/j.beem.2015.01.002. Epub 2015 Feb 26. Review. PubMed [citation]
PMID: 26051297

409. Flatøy B, Röhrl SM, Rydinge J, Dahl J, Diep LM, Nordsletten L. Triple taper stem
design shows promising fixation and bone remodelling characteristics:
radiostereometric analysis in a randomised controlled trial. Bone Joint J. 2015
Jun;97-B(6):755-61. doi: 10.1302/0301-620X.97B6.34736. PubMed [citation] PMID:
26033054

410. Barazani Y, Sabanegh E Jr. Rare case of monozygotic twins diagnosed with
klinefelter syndrome during evaluation for infertility. Rev Urol.
2015;17(1):42-5. Review. PubMed [citation] PMID: 26029003, PMCID:
PMC4444776

411. Nesgaard JM, Stimec BV, Bakka AO, Edwin B, Ignjatovic D; RCC study group..
Navigating the mesentery: a comparative pre- and per-operative visualization of
the vascular anatomy. Colorectal Dis. 2015 Sep;17(9):810-8. doi:
10.1111/codi.13003. PubMed [citation] PMID: 25988347

412. Basarslan SK, Gocmez C, Kamasak K, Ceviz A. The Gigant primary cerebral hydatid
cyst with no marked manifestation: a case report and review of literature. Eur
Rev Med Pharmacol Sci. 2015 Apr;19(8):1327-9. Review. PubMed [citation] PMID:
25967703

413. Starodub AN, Ocean AJ, Shah MA, Guarino MJ, Picozzi VJ Jr, Vahdat LT, Thomas SS,
Govindan SV, Maliakal PP, Wegener WA, Hamburger SA, Sharkey RM,

Goldenberg DM.
First-in-Human Trial of a Novel Anti-Trop-2 Antibody-SN-38 Conjugate, Sacituzumab
Govitecan, for the Treatment of Diverse Metastatic Solid Tumors. Clin Cancer Res.
2015 Sep 1;21(17):3870-8. doi: 10.1158/1078-0432.CCR-14-3321. Epub 2015 May 5.
PubMed [citation] PMID: 25944802, PMCID: PMC4558321

414. Granovsky Y, Liem KS, Weissman-Fogel I, Yarnitsky D, Chistyakov A, Sinai A.
'Virtual lesion' in pain research; a study on magnetic stimulation of the primary
motor cortex. Eur J Pain. 2016 Feb;20(2):241-9. doi: 10.1002/ejp.715. Epub 2015
Apr 27. PubMed [citation] PMID: 25919687

415. Kyritsi EM, Sertedaki A, Charmandari E, Chrousos GP. Familial or Sporadic Adrenal
Hypoplasia Syndromes. 2018 Oct 22. In: Feingold KR, Anawalt B, Blackman MR, Boyce
A, Chrousos G, Corpas E, de Herder WW, Dhatariya K, Dungan K, Hofland J, Kalra S,
Kaltsas G, Kapoor N, Koch C, Kopp P, Korbonits M, Kovacs CS, Kuohung W, et al,
editors.Endotext [Internet]. South Dartmouth (MA): MDText.com, Inc.; 2000-.
PubMed [citation] PMID: 25905355

416. Feingold KR. Atypical Forms of Diabetes. 2022 Feb 24. In: Feingold KR, Anawalt B,
Blackman MR, Boyce A, Chrousos G, Corpas E, de Herder WW, Dhatariya K, Dungan K,
Hofland J, Kalra S, Kaltsas G, Kapoor N, Koch C, Kopp P, Korbonits M, Kovacs CS,
Kuohung W, et al, editors.Endotext [Internet]. South Dartmouth (MA): MDText.com,
Inc.; 2000-. PubMed [citation] PMID: 25905351

417. Behre HM, Bergmann M, Simoni M, Tüttelmann F. Primary Testicular Failure. 2015
Aug 30. In: Feingold KR, Anawalt B, Blackman MR, Boyce A, Chrousos G, Corpas E,
de Herder WW, Dhatariya K, Dungan K, Hofland J, Kalra S, Kaltsas G, Kapoor N,
Koch C, Kopp P, Korbonits M, Kovacs CS, Kuohung W, et al, editors.Endotext
[Internet]. South Dartmouth (MA): MDText.com, Inc.; 2000-. PubMed [citation]
PMID: 25905302

418. Skakkebæk A, Wallentin M, Gravholt CH. Neuropsychology and socioeconomic aspects
of Klinefelter syndrome: new developments. Curr Opin Endocrinol Diabetes Obes.
2015 Jun;22(3):209-16. doi: 10.1097/MED.0000000000000157. Review. PubMed
[citation] PMID: 25899809

419. Stagi S, Iurato C, Lapi E, Cavalli L, Brandi ML, de Martino M. Bone status in
genetic syndromes: a review. Hormones (Athens). 2015 Jan-Mar;14(1):19-31. Review.
PubMed [citation] PMID: 25885101

420. Herlihy AS, McLachlan RI. Screening for Klinefelter syndrome. Curr Opin
Endocrinol Diabetes Obes. 2015 Jun;22(3):224-9. doi:
10.1097/MED.0000000000000154. Review. PubMed [citation] PMID: 25871960

421. Adams DJ, Clark DA. Common genetic and epigenetic syndromes. Pediatr Clin North
Am. 2015 Apr;62(2):411-26. doi: 10.1016/j.pcl.2014.11.005. Epub 2015 Jan 22.
Review. PubMed [citation] PMID: 25836705

422. Yassen GH, Sabrah AH, Eckert GJ, Platt JA. Effect of different endodontic
regeneration protocols on wettability, roughness, and chemical composition of
surface dentin. J Endod. 2015 Jun;41(6):956-60. doi: 10.1016/ j.joen.2015.02.023.
Epub 2015 Mar 29. PubMed [citation] PMID: 25823403

423. Hwang MJ, Brown H, Murrin R, Momtahan N, Sterne GD. Breast implant-associated
anaplastic large cell lymphoma: a case report and literature review. Aesthetic
Plast Surg. 2015 Jun;39(3):391-5. doi: 10.1007/s00266-015-0463-2. Epub 2015 Mar
5. Review. PubMed [citation] PMID: 25740078

424. Ropp A, Lin CT, White CS. Coronary computed tomography angiography for the
assessment of acute chest pain in the emergency department: evidence, guidelines,
and tips for implementation. J Thorac Imaging. 2015 May;30(3):169-75. doi:
10.1097/RTI.0000000000000128. Review. PubMed [citation] PMID: 25730553

425. Zeis R. Materials and characterization techniques for high-temperature polymer
electrolyte membrane fuel cells. Beilstein J Nanotechnol. 2015 Jan 7;6:68-83.
doi: 10.3762/bjnano.6.8. eCollection 2015. Review. PubMed [citation] PMID:
25671153, PMCID: PMC4311728

426. Tang JN, Shen DL, Liu CL, Wang XF, Zhang L, Xuan XX, Cui LL, Zhang JY. Plasma
levels of C1q/TNF-related protein 1 and interleukin 6 in patients with acute
coronary syndrome or stable angina pectoris. Am J Med Sci. 2015 Feb;349(2):130-6.
doi: 10.1097/MAJ.0000000000000378. PubMed [citation] PMID: 25635749

427. Liu B, Huang JW, Li Y, Hu BS, He X, Zhao W, Zheng YB, Lu LG. Single-Agent versus
Combination Doxorubicin-Based Transarterial Chemoembolization in the Treatment of
Hepatocellular Carcinoma: A Single-Blind, Randomized, Phase II Trial. Oncology.
2015;89(1):23-30. doi: 10.1159/000371522. Epub 2015 Jan 21. PubMed [citation]
PMID: 25613214

428. Jørgensen IN, Skakkebaek A, Andersen NH, Pedersen LN, Hougaard DM, Bojesen A,
Trolle C, Gravholt CH. Short QTc interval in males with klinefelter
syndrome-influence of CAG repeat length, body composition, and testosterone
replacement therapy. Pacing Clin Electrophysiol. 2015 Apr;38(4):472-82. doi:
10.1111/pace.12580. Epub 2015 Jan 23. PubMed [citation] PMID: 25615644

429. Lüscher TF, Steffel J. Individualized antithrombotic therapy. Hamostaseologie.
2016;36(1):26-32. doi: 10.5482/HAMO-14-12-0080. Epub 2015 Jan 19. Review. PubMed
[citation] PMID: 25597592

430. Huang J, Zhang L, Deng H, Chang L, Liu Q, Liu P. Global transcriptome analysis of
peripheral blood identifies the most significantly down-regulated genes
associated with metabolism regulation in Klinefelter syndrome. Mol Reprod Dev.
2015 Jan;82(1):17-25. doi: 10.1002/mrd.22438. Epub 2015 Jan 7. PubMed [citation]
PMID: 25581374

431. Fields GB. Biophysical studies of matrix metalloproteinase/ triple-helix
complexes. Adv Protein Chem Struct Biol. 2014;97:37-48. doi: 10.1016/bs.apcsb.2014.09.001. Epub 2014 Nov 7. Review. PubMed [citation] PMID:
25458354, PMCID: PMC4337812

432. Opitz JM. Serendipity or prepared mind? Recollections of the KOP translocation
(1967) and of one form of Perrault syndrome. Am J Med Genet C Semin Med Genet.
2014 Dec;166C(4):387-96. doi: 10.1002/ajmg.c.31420. Epub 2014 Nov 25. Review.
PubMed [citation] PMID: 25424868

433. Plotton I, Giscard d'Estaing S, Cuzin B, Brosse A, Benchaib M, Lornage J,
Ecochard R, Dijoud F, Lejeune H; FERTIPRESERVE group.. Preliminary results of a
prospective study of testicular sperm extraction in young versus adult patients
with nonmosaic 47,XXY Klinefelter syndrome. J Clin Endocrinol Metab. 2015
Mar;100(3):961-7. doi: 10.1210/jc.2014-3083. Epub 2014 Nov 25. PubMed [citation]
PMID: 25423570

434. Ahlberg S, Antonopulos A, Diendorf J, Dringen R, Epple M, Flöck R, Goedecke W,
Graf C, Haberl N, Helmlinger J, Herzog F, Heuer F, Hirn S, Johannes C, Kittler S,
Köller M, Korn K, Kreyling WG, Krombach F, Lademann J, Loza K, Luther EM, et al.
PVP-coated, negatively charged silver nanoparticles: A multi-center study of
their physicochemical characteristics, cell culture and in vivo experiments.
Beilstein J Nanotechnol. 2014 Nov 3;5:1944-65. doi: 10.3762/ bjnano.5.205.
eCollection 2014. Review. PubMed [citation] PMID: 25383306, PMCID: PMC4222445

435. Köhn FM, Schuppe HC. [Male infertility--pathogenic factors]. MMW Fortschr Med.
2014 Jun 26;156(12):40-3. Review. German. No abstract available. PubMed
[citation] PMID: 25369684

436. Clumeck N, Hill A, Moecklinghoff C. Effects of switching to

protease inhibitor
monotherapy on nucleoside analogue-related adverse events. AIDS Rev. 2014
Oct-Dec;16(4):236-45. Review. PubMed [citation] PMID: 25350532

437. Chikarmane SA, Tirumani SH, Howard SA, Jagannathan JP, DiPiro PJ. Metastatic
patterns of breast cancer subtypes: what radiologists should know in the era of
personalized cancer medicine. Clin Radiol. 2015 Jan;70(1):1-10. doi:
10.1016/j.crad.2014.08.015. Epub 2014 Oct 7. Review. PubMed [citation] PMID:
25300558

438. Blagden S, Omlin A, Josephs D, Stavraka C, Zivi A, Pinato DJ, Anthoney A,
Decordova S, Swales K, Riisnaes R, Pope L, Noguchi K, Shiokawa R, Inatani M,
Prince J, Jones K, Twelves C, Spicer J, Banerji U. First-in-human study of
CH5132799, an oral class I PI3K inhibitor, studying toxicity, pharmacokinetics,
and pharmacodynamics, in patients with metastatic cancer. Clin Cancer Res. 2014
Dec 1;20(23):5908-17. doi: 10.1158/1078-0432.CCR-14-1315. Epub 2014 Sep 17.
Erratum in: Clin Cancer Res. 2015 Feb 1;21(3):660. Olmin, Aurelius [corrected to
Omlin, Aurelius]. PubMed [citation] PMID: 25231405, PMCID: PMC4254850

439. Franco G, Misuraca L, Ciletti M, Leonardo C, De Nunzio C, Palminteri E, De
Dominicis C. [Surgery of male infertility: an update]. Urologia. 2014
Jul-Sep;81(3):154-64. doi: 10.5301/uro.5000088. Epub 2014 Sep 12. Review.
Italian. PubMed [citation] PMID: 25214369

440. Jacobs PA. An opportune life: 50 years in human cytogenetics. Annu Rev Genomics
Hum Genet. 2014;15:29-46. doi: 10.1146/annurev-genom-090413-025457. Review.
PubMed [citation] PMID: 25184528

441. Nistal M, Paniagua R, González-Peramato P, Reyes-Múgica M. Perspectives in
Pediatric Pathology, Chapter 16. Klinefelter Syndrome and Other Anomalies in X
and Y Chromosomes. Clinical and Pathological Entities. Pediatr Dev Pathol. 2016
Jul-Aug;19(4):259-77. doi: 10.2350/14-06-1512-PB.1. Epub 2014 Aug 8.

Review. No
abstract available. PubMed [citation] PMID: 25105890

442. Bar G, Lunenfeld E, Levitas E. [Klinefelter syndrome: genetic aspects,
characteristics and reproduction--present and future]. Harefuah. 2014
Jun;153(6):342-5, 366. Review. Hebrew. PubMed [citation] PMID:
25095608

443. Abramowitz LK, Olivier-Van Stichelen S, Hanover JA. Chromosome imbalance as a
driver of sex disparity in disease. J Genomics. 2014 Apr 1;2:77-88. doi:
10.7150/jgen.8123. eCollection 2014. Review. PubMed [citation] PMID: 25031659,
PMCID: PMC4091450

444. Hamaia S, Farndale RW. Integrin recognition motifs in the human collagens. Adv
Exp Med Biol. 2014;819:127-42. doi: 10.1007/978-94-017-9153-3_9. Review. PubMed
[citation] PMID: 25023172

445. Chinnaiyan KM, Bilolikar AN, Walsh E, Wood D, DePetris A, Gentry R, Boura J,
Abbara S, Al-Mallah M, Bis K, Boswell G, Gallagher M, Arunakul IO, Halliburton S,
Jacobs J, Lesser J, Schoepf UJ, Valeti US, Raff GL. CT dose reduction using
prospectively triggered or fast-pitch spiral technique employed in cardiothoracic
imaging (the CT dose study). J Cardiovasc Comput Tomogr. 2014
May-Jun;8(3):205-14. doi: 10.1016/j.jcct.2014.04.001. Epub 2014 Apr 13. PubMed
[citation] PMID: 24939069

446. Juul A, Almstrup K, Andersson AM, Jensen TK, Jørgensen N, Main KM, Rajpert-De
Meyts E, Toppari J, Skakkebæk NE. Possible fetal determinants of male
infertility. Nat Rev Endocrinol. 2014 Sep;10(9):553-62. doi:
10.1038/nrendo.2014.97. Epub 2014 Jun 17. Review. PubMed [citation] PMID:
24935122

447. Glueck CJ, Wang P. Testosterone therapy, thrombosis, thrombophilia,
cardiovascular events. Metabolism. 2014 Aug;63(8):989-94. doi:
10.1016/j.metabol.2014.05.005. Epub 2014 May 15. Review. PubMed [citation] PMID:
24930993

448. Mueller SC, Grissom EM, Dohanich GP. Assessing gonadal hormone contributions to
affective psychopathologies across humans and animal models.
Psychoneuroendocrinology. 2014 Aug;46:114-28. doi:
10.1016/j.psyneuen.2014.04.015. Epub 2014 Apr 28. Review. PubMed [citation] PMID:
24882164

449. Skakkebaek A, Bojesen A, Kristensen MK, Cohen A, Hougaard DM, Hertz JM, Fedder J,
Laurberg P, Wallentin M, Østergaard JR, Pedersen AD, Gravholt CH. Neuropsychology
and brain morphology in Klinefelter syndrome - the impact of genetics. Andrology.
2014 Jul;2(4):632-40. doi: 10.1111/j.2047-2927.2014.00229.x. Epub 2014 May 28.
PubMed [citation] PMID: 24865607

450. Landry A, Koyfman A. Should triple rule-out CT angiography be used in patients
with suspected acute coronary artery disease, aortic dissection, or pulmonary
embolus? Ann Emerg Med. 2015 Feb;65(2):216-7. doi:
10.1016/j.annemergmed.2014.04.015. Epub 2014 May 16. Review. No abstract
available. PubMed [citation] PMID: 24836704

451. Shanbhogue VV, Hansen S, Jørgensen NR, Brixen K, Gravholt CH. Bone geometry,
volumetric density, microarchitecture, and estimated bone strength assessed by
HR-pQCT in Klinefelter syndrome. J Bone Miner Res. 2014 Nov;29(11):2474-82. doi:
10.1002/jbmr.2272. PubMed [citation] PMID: 24806509

452. Chiquet M, Birk DE, Bönnemann CG, Koch M. Collagen XII: Protecting bone and
muscle integrity by organizing collagen fibrils. Int J Biochem Cell Biol. 2014
Aug;53:51-4. doi: 10.1016/j.biocel.2014.04.020. Epub 2014 May 4. Review. PubMed
[citation] PMID: 24801612, PMCID: PMC4119597

453. Gies I, Unuane D, Velkeniers B, De Schepper J. Management of Klinefelter syndrome
during transition. Eur J Endocrinol. 2014 Aug;171(2):R67-77. doi:
10.1530/EJE-14-0213. Epub 2014 May 6. Review. PubMed [citation] PMID:
24801585

454. Goossens E, Tournaye H. Male fertility preservation, where are we in 2014? Ann
Endocrinol (Paris). 2014 May;75(2):115-7. doi: 10.1016/j.ando.2014.03.011. Epub
2014 Apr 29. Review. PubMed [citation] PMID: 24793992

455. Nieschlag E, Werler S, Wistuba J, Zitzmann M. New approaches to the Klinefelter
syndrome. Ann Endocrinol (Paris). 2014 May;75(2):88-97. doi:
10.1016/j.ando.2014.03.007. Epub 2014 Apr 30. Review. PubMed [citation] PMID:
24793990

456. Plotton I, Brosse A, Cuzin B, Lejeune H. Klinefelter syndrome and TESE-ICSI. Ann
Endocrinol (Paris). 2014 May;75(2):118-25. doi: 10.1016/j.ando.2014.04.004. Epub
2014 Apr 29. Review. PubMed [citation] PMID: 24786702

457. Stouffs K, Seneca S, Lissens W. Genetic causes of male infertility. Ann
Endocrinol (Paris). 2014 May;75(2):109-11. doi: 10.1016/j.ando.2014.03.004. Epub
2014 Apr 24. Review. PubMed [citation] PMID: 24768008

458. Krausz C, Chianese C. Genetic testing and counselling for male infertility. Curr
Opin Endocrinol Diabetes Obes. 2014 Jun;21(3):244-50. doi:
10.1097/MED.0000000000000058. Review. PubMed [citation] PMID: 24739313

459. Zhu RH, Li HD, Cai HL, Jiang ZP, Xu P, Dai LB, Peng WX. Validated HILIC-MS/MS
assay for determination of vindesine in human plasma: Application to a population
pharmacokinetic study. J Pharm Biomed Anal. 2014 Aug 5;96:31-6. doi:
10.1016/j.jpba.2014.03.017. Epub 2014 Mar 23. PubMed [citation] PMID:
24721203

460. Hotaling J, Carrell DT. Clinical genetic testing for male factor infertility:
current applications and future directions. Andrology. 2014 May;2(3):339-50. doi:
10.1111/j.2047-2927.2014.00200.x. Epub 2014 Apr 7. Review. PubMed [citation]
PMID: 24711280

461. McCabe MJ, Bancalari RE, Dattani MT. Diagnosis and evaluation of hypogonadism.
Pediatr Endocrinol Rev. 2014 Feb;11 Suppl 2:214-29. Review. PubMed [citation]

PMID: 24683946

462. Park MR, Jeong RD, Kim KH. Understanding the intracellular trafficking and
intercellular transport of potexviruses in their host plants. Front Plant Sci.
2014 Mar 18;5:60. doi: 10.3389/fpls.2014.00060. eCollection 2014. Review. PubMed
[citation] PMID: 24672528, PMCID: PMC3957223

463. Darnis A, Launay O, Perrin G, Barrey C. Surgical management of multilevel lumbar
spondylolysis: a case report and review of the literature. Orthop Traumatol Surg
Res. 2014 May;100(3):347-51. doi: 10.1016/j.otsr.2013.12.021. Epub 2014 Mar 19.
Review. PubMed [citation] PMID: 24657151

464. Overvad S, Bay K, Bojesen A, Gravholt CH. Low INSL3 in Klinefelter syndrome is
related to osteocalcin, testosterone treatment and body composition, as well as
measures of the hypothalamic-pituitary-gonadal axis. Andrology. 2014
May;2(3):421-7. doi: 10.1111/j.2047-2927.2014.00204.x. Epub 2014 Mar 21. PubMed
[citation] PMID: 24659579

465. Prasad R, Kowalczyk JC, Meimaridou E, Storr HL, Metherell LA. Oxidative stress
and adrenocortical insufficiency. J Endocrinol. 2014 Jun;221(3):R63-73. doi:
10.1530/JOE-13-0346. Epub 2014 Mar 12. Review. PubMed [citation] PMID: 24623797,
PMCID: PMC4045218

466. Aston KI. Genetic susceptibility to male infertility: news from genome-wide
association studies. Andrology. 2014 May;2(3):315-21. doi:
10.1111/j.2047-2927.2014.00188.x. Epub 2014 Feb 19. Review. PubMed [citation]
PMID: 24574159

467. Hong DS, Reiss AL. Cognitive and neurological aspects of sex chromosome
aneuploidies. Lancet Neurol. 2014 Mar;13(3):306-18. doi:
10.1016/S1474-4422(13)70302-8. Epub 2014 Feb 17. Review. PubMed [citation] PMID:
24556008

468. Brinton LA, Cook MB, McCormack V, Johnson KC, Olsson H,

Casagrande JT, Cooke R,
Falk RT, Gapstur SM, Gaudet MM, Gaziano JM, Gkiokas G, Guénel P, Henderson BE,
Hollenbeck A, Hsing AW, Kolonel LN, Isaacs C, Lubin JH, Michels KB, Negri E,
Parisi D, et al. Anthropometric and hormonal risk factors for male breast cancer:
male breast cancer pooling project results. J Natl Cancer Inst. 2014
Mar;106(3):djt465. doi: 10.1093/jnci/djt465. Epub 2014 Feb 19. Erratum in: J Natl
Cancer Inst. 2014 May;106(5):dju117. PubMed [citation] PMID: 24552677, PMCID:
PMC3975166

469. Butnariu L, Rusu C, Caba L, Pânzaru M, Braha E, Grămescu M, Popescu R, Bujoranu
C, Gorduza EV. Genotype- phenotype correlation in trisomy X: a retrospective
study of a selected group of 36 patients and review of literature. Rev Med Chir
Soc Med Nat Iasi. 2013 Jul-Sep;117(3):714-21. Review. PubMed [citation] PMID:
24502039

470. Irwig MS. Male hypogonadism and skeletal health. Curr Opin Endocrinol Diabetes
Obes. 2013 Dec;20(6):517-22. doi: 10.1097/01.med.0000436185.36717.76. Review.
PubMed [citation] PMID: 24468754

471. Truong QA, Gewirtz H. Cardiac PET-CT for monitoring medical and interventional
therapy in patients with CAD: PET alone versus hybrid PET-CT? Curr Cardiol Rep.
2014 Mar;16(3):460. doi: 10.1007/s11886-013-0460-5. Review. PubMed [citation]
PMID: 24464305, PMCID: PMC4484581

472. Margari L, Lamanna AL, Craig F, Simone M, Gentile M. Autism spectrum disorders in
XYY syndrome: two new cases and systematic review of the literature. Eur J
Pediatr. 2014 Mar;173(3):277-83. doi: 10.1007/s00431-014-2267-9. Epub 2014 Jan
25. Review. PubMed [citation] PMID: 24464091

473. Allan CA. Sex steroids and glucose metabolism. Asian J Androl. 2014
Mar-Apr;16(2):232-8. doi: 10.4103/1008-682X.122589. Review. PubMed [citation]

PMID: 24457840, PMCID: PMC3955332

474. Lejeune H, Brosse A; Groupe Fertipreserve., Plotton I. [Fertility in Klinefelter
syndrome]. Presse Med. 2014 Feb;43(2):162-70. doi: 10.1016/j.lpm.2013.12.002.
Epub 2014 Jan 16. Review. French. PubMed [citation] PMID: 24439539

475. Høst C, Skakkebæk A, Groth KA, Bojesen A. The role of hypogonadism in Klinefelter
syndrome. Asian J Androl. 2014 Mar-Apr;16(2):185-91. doi:
10.4103/1008-682X.122201. Review. PubMed [citation] PMID: 24407186, PMCID:
PMC3955327

476. Cox KH, Bonthuis PJ, Rissman EF. Mouse model systems to study sex chromosome
genes and behavior: relevance to humans. Front Neuroendocrinol. 2014
Oct;35(4):405-19. doi: 10.1016/j.yfrne.2013.12.004. Epub 2014 Jan 2. Review.
PubMed [citation] PMID: 24388960, PMCID: PMC4079771

477. Josso N, Rey RA, Picard JY. Anti-müllerian hormone: a valuable addition to the
toolbox of the pediatric endocrinologist. Int J Endocrinol. 2013;2013:674105.
doi: 10.1155/2013/674105. Epub 2013 Dec 8. Review. PubMed [citation] PMID:
24382961, PMCID: PMC3870610

478. Metcalfe A, Hippman C, Pastuck M, Johnson JA. Beyond Trisomy 21: Additional
Chromosomal Anomalies Detected through Routine Aneuploidy Screening. J Clin Med.
2014 Apr 8;3(2):388-415. doi: 10.3390/jcm3020388. Review. PubMed [citation] PMID:
26237381, PMCID: PMC4449689

479. Bertorini TE, Perez A. Neurologic complications of disorders of the adrenal
glands. Handb Clin Neurol. 2014;120:749-71. doi:
10.1016/B978-0-7020-4087-0.00050-4. Review. PubMed [citation] PMID:
24365350

480. Keenan NG, Pugliese F, Davies LC. The role of computed tomography in
cardiovascular imaging: from X-ray department to emergency room. Expert Rev
Cardiovasc Ther. 2014 Jan;12(1):57-69. doi:
10.1586/14779072.2014.870034. Epub

2013 Dec 18. Review. PubMed [citation] PMID: 24345094

481. Koegl-Wallner M, Katschnig-Winter P, Pendl T, Melisch B, Trummer M, Holl E,
Werner U, Schmidt R, Schwingenschuh P. Tremor associated with Klinefelter
syndrome--a case series and review of the literature. Parkinsonism Relat Disord.
2014 Mar;20(3):323-7. doi: 10.1016/j.parkreldis.2013.11.010. Epub 2013 Dec 4.
Review. PubMed [citation] PMID: 24345964

482. Lau TK, Cheung SW, Lo PS, Pursley AN, Chan MK, Jiang F, Zhang H, Wang W, Jong LF,
Yuen OK, Chan HY, Chan WS, Choy KW. Non-invasive prenatal testing for fetal
chromosomal abnormalities by low-coverage whole-genome sequencing of maternal
plasma DNA: review of 1982 consecutive cases in a single center. Ultrasound
Obstet Gynecol. 2014 Mar;43(3):254-64. doi: 10.1002/uog.13277. Epub 2014 Feb 10.
Review. PubMed [citation] PMID: 24339153

483. Blomberg BA, Thomassen A, Takx RA, Vilstrup MH, Hess S, Nielsen AL, Diederichsen
AC, Mickley H, Alavi A, Høilund-Carlsen PF. Delayed sodium 18F-fluoride PET/CT
imaging does not improve quantification of vascular calcification metabolism:
results from the CAMONA study. J Nucl Cardiol. 2014 Apr;21(2):293-304. doi:
10.1007/s12350-013-9829-5. Epub 2013 Dec 5. PubMed [citation] PMID: 24307262

484. Kidoh M, Nakaura T, Nakamura S, Namimoto T, Nozaki T, Sakaino N, Harada K,
Yamashita Y. Contrast material and radiation dose reduction strategy for
triple-rule-out cardiac CT angiography: feasibility study of non-ECG-gated low
kVp scan of the whole chest following coronary CT angiography. Acta Radiol. 2014
Dec;55(10):1186-96. doi: 10.1177/0284185113514886. Epub 2013 Dec 5. PubMed
[citation] PMID: 24311703

485. Hotaling JM. Genetics of male infertility. Urol Clin North Am. 2014
Feb;41(1):1-17. doi: 10.1016/j.ucl.2013.08.009. Epub 2013 Oct 23.

Review. PubMed
[citation] PMID: 24286764

486. Veeramachaneni DN, Klinefelter GR. Phthalate-induced pathology in the foetal
testis involves more than decreased testosterone production. Reproduction. 2014
Mar 2;147(4):435-42. doi: 10.1530/REP-13-0441. Print 2014. Review. PubMed
[citation] PMID: 24282314

487. Ludwig W, Phillips M. Organic causes of erectile dysfunction in men under 40.
Urol Int. 2014;92(1):1-6. doi: 10.1159/000354931. Epub 2013 Nov 21. Review.
PubMed [citation] PMID: 24281298

488. Zeitlin SI, Rajfer J, Shapiro E. Best of the 2013 AUA Annual Meeting Part II:
More Highlights From the 2013 American Urological Association Annual Meeting, May
4-8, 2013, San Diego, CA. Rev Urol. 2013;15(3):118-23. Review. No abstract
available. PubMed [citation] PMID: 24223024, PMCID: PMC3821991

489. Guaraldi G, Zona S, Cossarizza A, Vernacotola L, Carli F, Lattanzi A, Nardini G,
Orlando G, Garlassi E, Termini R, Garau M. Switching to darunavir/ritonavir
monotherapy vs. triple-therapy on body fat redistribution and bone mass in
HIV-infected adults: the Monarch randomized controlled trial. Int J STD AIDS.
2014 Mar;25(3):207-12. doi: 10.1177/0956462413497701. Epub 2013 Aug 28. PubMed
[citation] PMID: 24216034

490. Khurana KK, Sabanegh ES Jr. Office-based sperm retrieval for treatment of
infertility. Urol Clin North Am. 2013 Nov;40(4):569-79. doi:
10.1016/j.ucl.2013.07.005. Epub 2013 Aug 8. Review. PubMed [citation] PMID:
24182976

491. Jo DG, Lee HS, Joo YM, Seo JT. Effect of testosterone replacement therapy on bone
mineral density in patients with Klinefelter syndrome. Yonsei Med J. 2013
Nov;54(6):1331-5. doi: 10.3349/ymj.2013.54.6.1331. PubMed [citation] PMID:

24142635, PMCID: PMC3809853

492. Lalatta F, Tint GS. Counseling parents before prenatal diagnosis: do we need to
say more about the sex chromosome aneuploidies? Am J Med Genet A. 2013
Nov;161A(11):2873-9. doi: 10.1002/ajmg.a.36226. Epub 2013 Sep 24. Review. PubMed
[citation] PMID: 24115600

493. Merenstein DJ, D'Amico F, Palese C, Hahn A, Sparenborg J, Tan T, Scott H, Polzin
K, Kolberg L, Roberts R. Short-term, daily intake of yogurt containing
Bifidobacterium animalis ssp. lactis Bf-6 (LMG 24384) does not affect colonic
transit time in women. Br J Nutr. 2014 Jan 28;111(2):279-86. doi:
10.1017/S0007114513002237. Epub 2013 Oct 8. PubMed [citation] PMID:
24103188

494. De Sanctis V, Fiscina B, Soliman A, Giovannini M, Yassin M. Klinefelter syndrome
and cancer: from childhood to adulthood. Pediatr Endocrinol Rev. 2013
Sep;11(1):44-50. Review. PubMed [citation] PMID: 24079078

495. den Hollander P, Savage MI, Brown PH. Targeted therapy for breast cancer
prevention. Front Oncol. 2013 Sep 23;3:250. doi: 10.3389/
fonc.2013.00250. Review.
PubMed [citation] PMID: 24069582, PMCID: PMC3780469

496. Selten JP, van der Ven E, Rutten BP, Cantor-Graae E. The social defeat hypothesis
of schizophrenia: an update. Schizophr Bull. 2013 Nov;39(6):1180-6. doi:
10.1093/schbul/sbt134. Epub 2013 Sep 23. Review. PubMed [citation] PMID:
24062592, PMCID: PMC3796093

497. Irwin RW, Brinton RD. Allopregnanolone as regenerative therapeutic for
Alzheimer's disease: translational development and clinical promise. Prog
Neurobiol. 2014 Feb;113:40-55. doi: 10.1016/j.pneurobio.2013.08.004. Epub 2013
Sep 14. Review. PubMed [citation] PMID: 24044981, PMCID: PMC10124616

498. Ayaram D, Bellolio MF, Murad MH, Laack TA, Sadosty AT, Erwin PJ, Hollander JE,
Montori VM, Stiell IG, Hess EP. Triple rule-out computed tomographic angiography
for chest pain: a diagnostic systematic review and meta-analysis. Acad

Emerg Med.
2013 Sep;20(9):861-71. doi: 10.1111/acem.12210. Review. PubMed [citation] PMID:
24050793

499. Mark Courtney D. Triple rule out: why it is not ready to roll out. Acad Emerg
Med. 2013 Sep;20(9):934-6. doi: 10.1111/acem.12196. No abstract available. PubMed
[citation] PMID: 24050800

500. Nectoux E, Hocquet B, Fron D, Mezel A, Paris A, Herbaux B. Unpredictability of
hip behavior in Dyggve-Melchior-Clausen syndrome: a mid-term assessment of
siblings. Orthop Traumatol Surg Res. 2013 Oct;99(6):745-8. doi:
10.1016/j.otsr.2013.04.006. Epub 2013 Sep 12. Review. PubMed [citation] PMID:
24035654

501. Klimeczek P, Zaleska-Dorobisz U, Jagas J, Harań T. [The clinical value of
computer tomography (CT) of diagnostics of acute thorax pain--a literature
review]. Przegl Lek. 2013;70(3):123-7. Review. Polish. PubMed [citation] PMID:
24003665

502. Piomboni P, Stendardi A, Gambera L. Chromosomal aberrations and aneuploidies of
spermatozoa. Adv Exp Med Biol. 2014;791:27-52. doi: 10.1007/978-1-4614-7783-9_3.
Review. PubMed [citation] PMID: 23955671

503. Arver S, Luong B, Fraschke A, Ghatnekar O, Stanisic S, Gultyev D, Müller E. Is
testosterone replacement therapy in males with hypogonadism cost-effective? An
analysis in Sweden. J Sex Med. 2014 Jan;11(1):262-72. doi: 10.1111/jsm.12277.
Epub 2013 Aug 12. Review. PubMed [citation] PMID: 23937088

504. Psathas ED, Katsargyris A, Lioudaki S, Moris DN, Doulaptsis M, Klonaris C.
Treatment paradigms for ductus arteriosus aneurysms in adults. Vascular. 2014
Aug;22(4):297-301. doi: 10.1177/1708538113495680. Epub 2013 Jul 2. Review. PubMed
[citation] PMID: 23929428

505. Schwemmle C, Jungheim M, Ptok M. [Gonosomal trisomy syndrome. Five case reports
and review of literature]. Laryngorhinootologie. 2013 Nov;92(11):725-31. doi:
10.1055/s-0033-1348249. Epub 2013 Aug 8. Review. German. PubMed [citation] PMID:
23929211

506. Pachajoa H. [Double aneuploidy (trisomy X, trisomy 18) in a newborn with trisomy
18 phenotype]. Arch Argent Pediatr. 2013 Jul-Aug;111(4):e101-4. doi:
10.1590/S0325-00752013000400019. Review. Spanish. PubMed [citation] PMID:
23912296

507. Liu JM, Tian WH, Tian JG, Li HT, Qi FJ, Fan Y, Chen S. [Observation on
therapeutic effect of round-sharp needle of new nine-needle and elongated needle
for piriformis syndrome with triple puncture method]. Zhongguo Zhen Jiu. 2013
May;33(5):422-5. Chinese. PubMed [citation] PMID: 23885616

508. Nieschlag E. Klinefelter syndrome: the commonest form of hypogonadism, but often
overlooked or untreated. Dtsch Arztebl Int. 2013 May;110(20):347-53. doi:
10.3238/arztebl.2013.0347. Epub 2013 May 17. Review. PubMed [citation] PMID:
23825486, PMCID: PMC3674537

509. Buckens CF, de Jong PA, Mali WP, Verhaar HJ, van der Graaf Y, Verkooijen HM.
Prevalent vertebral fractures on chest CT: higher risk for future hip fracture. J
Bone Miner Res. 2014 Feb;29(2):392-8. doi: 10.1002/jbmr.2028. PubMed [citation]
PMID: 23821454

510. Byler MC, Lebel RR. Risks of reproducing with a genetic disorder. Semin Reprod
Med. 2013 Jul;31(4):258-66. doi: 10.1055/s-0033-1345273. Epub 2013 Jun 17.
Review. PubMed [citation] PMID: 23775381

511. Pocha C, Dieperink E, McMaken KA, Knott A, Thuras P, Ho SB. Surveillance for
hepatocellular cancer with ultrasonography vs. computed tomography -- a
randomised study. Aliment Pharmacol Ther. 2013 Aug;38(3):303-12. doi:

10.1111/apt.12370. Epub 2013 Jun 10. PubMed [citation] PMID: 23750991

512. Hoffman MD. Atypical ulcers. Dermatol Ther. 2013 May-Jun;26(3):222-35. doi:
10.1111/dth.12048. Review. PubMed [citation] PMID: 23742283

513. Patel DN, Li L, Kee CL, Ge X, Low MY, Koh HL. Screening of synthetic PDE-5
inhibitors and their analogues as adulterants: analytical techniques and
challenges. J Pharm Biomed Anal. 2014 Jan;87:176-90. doi:
10.1016/j.jpba.2013.04.037. Epub 2013 May 6. Review. PubMed [citation] PMID:
23721687

514. Ulgiati F, Nicita F, Papetti L, Ursitti F, Di Maggio A, Tarani L, Spalice A.
Posterior fossa malformations and sex chromosomes anomalies. Report of a case
with XYY syndrome and overview of known associations. Eur J Pediatr. 2013
Sep;172(9):1267-70. doi: 10.1007/s00431-013-2039-y. Epub 2013 May 22. Review.
PubMed [citation] PMID: 23695861

515. Mueller SC. Magnetic resonance imaging in paediatric psychoneuroendocrinology: a
new frontier for understanding the impact of hormones on emotion and cognition. J
Neuroendocrinol. 2013 Aug;25(8):762-70. doi: 10.1111/jne.12048. Review. PubMed
[citation] PMID: 23656557

516. Hutaff-Lee C, Cordeiro L, Tartaglia N. Cognitive and medical features of
chromosomal aneuploidy. Handb Clin Neurol. 2013;111:273-9. doi:
10.1016/B978-0-444-52891-9.00030-0. Review. PubMed [citation] PMID:
23622175

517. Ithimakin S, Ratanawichitrasin A, Veerasarn V, Akewanlop C, Soparattanapaisarn N,
Rojananin S, O-Charoenrat P, Prasarttong-Osoth P, Srimuninnimit V. A phase II
study of the combination of gemcitabine plus carboplatin as the neoadjuvant
treatment in locally advanced breast cancer. J Med Assoc Thai. 2013 Feb;96 Suppl
2:S67-74. PubMed [citation] PMID: 23590024

518. Dávila Garza SA, Patrizio P. Reproductive outcomes in patients

with male
infertility because of Klinefelter's syndrome, Kartagener's syndrome, round-head
sperm, dysplasia fibrous sheath, and 'stump' tail sperm: an updated literature
review. Curr Opin Obstet Gynecol. 2013 Jun;25(3):229-46. doi:
10.1097/GCO.0b013e32835faae5. Review. PubMed [citation] PMID: 23587797

519. Valeri C, Schteingart HF, Rey RA. The prepubertal testis: biomarkers and
functions. Curr Opin Endocrinol Diabetes Obes. 2013 Jun;20(3):224-33. doi:
10.1097/MED.0b013e328360be2c. Review. PubMed [citation] PMID: 23549308

520. Rezaei H, Saevarsdottir S, Geborek P, Petersson IF, van Vollenhoven RF, Forslind
K. Evaluation of hand bone loss by digital X-ray radiogrammetry as a complement
to clinical and radiographic assessment in early rheumatoid arthritis: results
from the SWEFOT trial. BMC Musculoskelet Disord. 2013 Mar 5;14:79. doi:
10.1186/1471-2474-14-79. PubMed [citation] PMID: 23497111, PMCID: PMC3599105

521. Aksglaede L, Juul A. Testicular function and fertility in men with Klinefelter
syndrome: a review. Eur J Endocrinol. 2013 Mar 15;168(4):R67-76. doi:
10.1530/EJE-12-0934. Print 2013 Apr. Review. PubMed [citation] PMID: 23504510

522. Colfry AJ 3rd. Miscellaneous syndromes and their management: occult breast
cancer, breast cancer in pregnancy, male breast cancer, surgery in stage IV
disease. Surg Clin North Am. 2013 Apr;93(2):519-31. doi:
10.1016/j.suc.2012.12.003. Epub 2013 Feb 7. Review. PubMed [citation] PMID:
23464700

523. Goossens E, Van Saen D, Tournaye H. Spermatogonial stem cell preservation and
transplantation: from research to clinic. Hum Reprod. 2013 Apr;28(4):897-907.
doi: 10.1093/humrep/det039. Epub 2013 Feb 20. Review. PubMed [citation] PMID:
23427228

524. Umashankara M, Nanda M, Sonar M, Ganesh KN. 4(R/S)-Amino/ guanidino-substituted

proline peptides: design, synthesis and DNA transfection properties. Chimia
(Aarau). 2012;66(12):936-40. doi: 10.2533/chimia.2012.936. Review. PubMed
[citation] PMID: 23394278

525. Simpson JL, Samango-Sprouse C. Prenatal diagnosis and 47,XXY. Am J Med Genet C
Semin Med Genet. 2013 Feb 15;163C(1):64-70. doi: 10.1002/ajmg.c.31356. Review.
PubMed [citation] PMID: 23359597

526. McCarthy MM. Sexual differentiation of the brain in man and animals: of relevance
to Klinefelter syndrome? Am J Med Genet C Semin Med Genet. 2013 Feb
15;163C(1):3-15. doi: 10.1002/ajmg.c.31351. Epub 2013 Jan 18. Review. PubMed
[citation] PMID: 23335108, PMCID: PMC5320421

527. Wosnitzer MS, Paduch DA. Endocrinological issues and hormonal manipulation in
children and men with Klinefelter syndrome. Am J Med Genet C Semin Med Genet.
2013 Feb 15;163C(1):16-26. doi: 10.1002/ajmg.c.31350. Epub 2013 Jan 18. Review.
PubMed [citation] PMID: 23335092

528. Gropman A, Samango-Sprouse CA. Neurocognitive variance and neurological
underpinnings of the X and Y chromosomal variations. Am J Med Genet C Semin Med
Genet. 2013 Feb 15;163C(1):35-43. doi: 10.1002/ajmg.c.31352. Epub 2013 Jan 18.
Review. PubMed [citation] PMID: 23335129

529. Liehr T, Klein E, Mrasek K, Kosyakova N, Guilherme RS, Aust N, Venner C, Weise A,
Hamid AB. Clinical impact of somatic mosaicism in cases with small supernumerary
marker chromosomes. Cytogenet Genome Res. 2013;139(3):158-63. doi:
10.1159/000346026. Epub 2012 Dec 29. Review. PubMed [citation] PMID:
23295254

530. Jayasuriya RL, Buckley SC, Hamer AJ, Kerry RM, Stockley I, Tomouk MW, Wilkinson
JM. Effect of sliding-taper compared with composite-beam cemented femoral
prosthesis loading regime on proximal femoral bone remodeling: a randomized
clinical trial. J Bone Joint Surg Am. 2013 Jan 2;95(1):19-27. doi:

10.2106/JBJS.K.00657. PubMed [citation] PMID: 23283370

531. Uhl JF, Gillot C. Anatomy and embryology of the small saphenous vein: nerve
relationships and implications for treatment. Phlebology. 2013 Feb;28(1):4-15.
doi: 10.1258/phleb.2012.012J08. Epub 2012 Dec 19. Review. PubMed [citation] PMID:
23256200

532. Dabaja AA, Schlegel PN. Microdissection testicular sperm extraction: an update.
Asian J Androl. 2013 Jan;15(1):35-9. doi: 10.1038/aja.2012.141. Epub 2012 Dec 17.
Review. PubMed [citation] PMID: 23241638, PMCID: PMC3739122

533. Lam JT, Jacob S. Boceprevir: a recently approved protease inhibitor for hepatitis
C virus infection. Am J Health Syst Pharm. 2012 Dec 15;69(24):2135-9. doi:
10.2146/ajhp110500. Review. PubMed [citation] PMID: 23230035

534. Roivainen A, Hautaniemi S, Möttönen T, Nuutila P, Oikonen V, Parkkola R, Pricop
L, Ress R, Seneca N, Seppänen M, Yli-Kerttula T. Correlation of 18F-FDG PET/CT
assessments with disease activity and markers of inflammation in patients with
early rheumatoid arthritis following the initiation of combination therapy with
triple oral antirheumatic drugs. Eur J Nucl Med Mol Imaging. 2013
Feb;40(3):403-10. doi: 10.1007/s00259-012-2282-x. Epub 2012 Nov 15. PubMed
[citation] PMID: 23229747

535. Carafoli F, Hohenester E. Collagen recognition and transmembrane signalling by
discoidin domain receptors. Biochim Biophys Acta. 2013 Oct;1834(10):2187-94. doi:
10.1016/j.bbapap.2012.10.014. Epub 2012 Nov 2. Review. PubMed [citation] PMID:
23128141, PMCID: PMC4332414

536. Groth KA, Skakkebæk A, Høst C, Gravholt CH, Bojesen A. Clinical review:
Klinefelter syndrome--a clinical update. J Clin Endocrinol Metab. 2013
Jan;98(1):20-30. doi: 10.1210/jc.2012-2382. Epub 2012 Nov 1. Review. PubMed
[citation] PMID: 23118429

537. Zimmermann B, Hill M, Gemelos G, Demko Z, Banjevic M, Baner J, Ryan A,
Sigurjonsson S, Chopra N, Dodd M, Levy B, Rabinowitz M. Noninvasive prenatal
aneuploidy testing of chromosomes 13, 18, 21, X, and Y, using targeted sequencing
of polymorphic loci. Prenat Diagn. 2012 Dec;32(13):1233-41. doi: 10.1002/pd.3993.
Epub 2012 Oct 30. PubMed [citation] PMID: 23108718, PMCID: PMC3548605

538. Thaler M, Lechner R, Gstöttner M, Kobel C, Bach C. The use of beta-tricalcium
phosphate and bone marrow aspirate as a bone graft substitute in posterior lumbar
interbody fusion. Eur Spine J. 2013 May;22(5):1173-82. doi:
10.1007/s00586-012-2541-3. Epub 2012 Oct 17. PubMed [citation] PMID: 23073745,
PMCID: PMC3657048

539. García Vicente AM, Soriano Castrejón A, Cruz Mora MA, González Ageitos A, Muñoz
Sánchez Mdel M, León Martín A, Espinosa Aunión R, Relea Calatayud F, Muñoz Madero
V, Chacón López-Muñiz I, Cordero García JM, Jiménez Londoño GA. Semi-quantitative
lymph node assessment of (18)F-FDG PET/CT in locally advanced breast cancer:
correlation with biological prognostic factors. Eur J Nucl Med Mol Imaging. 2013
Jan;40(1):72-9. doi: 10.1007/s00259-012-2244-3. Epub 2012 Sep 28. PubMed
[citation] PMID: 23053321

540. Jahnukainen K, Stukenborg JB. Clinical review: Present and future prospects of
male fertility preservation for children and adolescents. J Clin Endocrinol
Metab. 2012 Dec;97(12):4341-51. doi: 10.1210/jc.2012-3065. Epub 2012 Oct 4.
Review. PubMed [citation] PMID: 23038680

541. Kauppi L, Jasin M, Keeney S. The tricky path to recombining X and Y chromosomes
in meiosis. Ann N Y Acad Sci. 2012 Sep;1267:18-23. doi:
10.1111/j.1749-6632.2012.06593.x. Review. PubMed [citation] PMID: 22954211,
PMCID: PMC3631422

542. Sarma A, Heilbrun ME, Conner KE, Stevens SM, Woller SC, Elliott CG. Radiation and

chest CT scan examinations: what do we know? Chest. 2012
Sep;142(3):750-760. doi:
10.1378/chest.11-2863. Review. PubMed [citation] PMID: 22948579

543. Aksglaede L, Garn ID, Hollegaard MV, Hougaard DM, Rajpert-De Meyts E, Juul A.
Detection of increased gene copy number in DNA from dried blood spot samples
allows efficient screening for Klinefelter syndrome. Acta Paediatr. 2012
Dec;101(12):e561-3. doi: 10.1111/apa.12008. Epub 2012 Sep 15. No abstract
available. PubMed [citation] PMID: 22928958

544. Li C, Yu G. [Progress in diagnosis and treatment of tarsal coalition]. Zhongguo
Xiu Fu Chong Jian Wai Ke Za Zhi. 2012 Jul;26(7):874-9. Review. Chinese. PubMed
[citation] PMID: 22905629

545. Oates RD. The natural history of endocrine function and spermatogenesis in
Klinefelter syndrome: what the data show. Fertil Steril. 2012 Aug;98(2):266-73.
doi: 10.1016/j.fertnstert.2012.06.024. Review. PubMed [citation] PMID: 22846647

546. Yang J, Liu JH. [Microdissection testicular sperm extraction for non-obstructive
azoospermia]. Zhonghua Nan Ke Xue. 2012 Jun;18(6):551-5. Review. Chinese. PubMed
[citation] PMID: 22774614

547. Gruettner J, Fink C, Walter T, Meyer M, Apfaltrer P, Schoepf UJ, Saur J,
Sueselbeck T, Traunwieser D, Takx R, Kralev S, Borggrefe M, Schoenberg SO,
Henzler T. Coronary computed tomography and triple rule out CT in patients with
acute chest pain and an intermediate cardiac risk profile. Part 1: impact on
patient management. Eur J Radiol. 2013 Jan;82(1):100-5. doi:
10.1016/j.ejrad.2012.06.001. Epub 2012 Jun 29. PubMed [citation] PMID: 22749769

548. Maiburg M, Repping S, Giltay J. The genetic origin of Klinefelter syndrome and
its effect on spermatogenesis. Fertil Steril. 2012 Aug;98(2):253-60. doi:
10.1016/j.fertnstert.2012.06.019. Epub 2012 Jun 29. Review. PubMed

[citation]
PMID: 22749222

549. Mehta A, Paduch DA. Klinefelter syndrome: an argument for early aggressive
hormonal and fertility management. Fertil Steril. 2012 Aug;98(2):274-83. doi:
10.1016/j.fertnstert.2012.06.001. Epub 2012 Jun 23. Review. PubMed [citation]
PMID: 22732737

550. Sigman M. Klinefelter syndrome: how, what, and why? Fertil Steril. 2012
Aug;98(2):251-2. doi: 10.1016/j.fertnstert.2012.05.011. Epub 2012 Jun 20. Review.
PubMed [citation] PMID: 22726951

551. Sokol RZ. It's not all about the testes: medical issues in Klinefelter patients.
Fertil Steril. 2012 Aug;98(2):261-5. doi: 10.1016/ j.fertnstert.2012.05.026. Epub
2012 Jun 15. Review. PubMed [citation] PMID: 22704628

552. Shen Z, Zou CC, Shang SQ, Jiang KW. Down-Klinefelter syndrome (48,XXY,+21) in a
child with congenital heart disease: case report and literature review. Intern
Med. 2012;51(11):1371-4. Epub 2012 Jun 1. Review. PubMed [citation] PMID:
22687844

553. Zavorsky GS, Smoliga JM, Longo LD, Uhranowsky KA, Cadman CR, Duffin J, Fisher JA.
Increased carbon monoxide clearance during exercise in humans. Med Sci Sports
Exerc. 2012 Nov;44(11):2118-24. doi: 10.1249/MSS.0b013e3182602a00. PubMed
[citation] PMID: 22648340

554. Gies I, De Schepper J, Goossens E, Van Saen D, Pennings G, Tournaye H.
Spermatogonial stem cell preservation in boys with Klinefelter syndrome: to bank
or not to bank, that's the question. Fertil Steril. 2012 Aug;98(2):284-9. doi:
10.1016/j.fertnstert.2012.04.023. Epub 2012 May 17. Review. PubMed [citation]
PMID: 22608314

555. Savic I. Advances in research on the neurological and

neuropsychiatric phenotype
of Klinefelter syndrome. Curr Opin Neurol. 2012 Apr;25(2):138-43. doi:
10.1097/WCO.0b013e32835181a0. Review. PubMed [citation] PMID: 22395004

556. Mahjoubi F, Razazian F. Constitutional complex chromosomal rearrangements in a
klinefelter patient: case report and review of literature. J Assist Reprod Genet.
2012 May;29(5):437-41. doi: 10.1007/s10815-012-9725-y. Epub 2012 Mar 1. Review.
PubMed [citation] PMID: 22382640, PMCID: PMC3348273

557. Bianchi DW, Platt LD, Goldberg JD, Abuhamad AZ, Sehnert AJ, Rava RP; MatErnal
BLood IS Source to Accurately diagnose fetal aneuploidy (MELISSA) Study Group..
Genome-wide fetal aneuploidy detection by maternal plasma DNA sequencing. Obstet
Gynecol. 2012 May;119(5):890-901. doi: 10.1097/AOG.0b013e31824fb482. Erratum in:
Obstet Gynecol. 2012 Oct;120(4):957. PubMed [citation] PMID: 22362253

558. Groheux D, Hindié E, Giacchetti S, Delord M, Hamy AS, de Roquancourt A,
Vercellino L, Berenger N, Marty M, Espié M. Triple-negative breast cancer: early
assessment with 18F-FDG PET/CT during neoadjuvant chemotherapy identifies
patients who are unlikely to achieve a pathologic complete response and are at a
high risk of early relapse. J Nucl Med. 2012 Feb;53(2):249-54. doi:
10.2967/jnumed.111.094045. Epub 2012 Jan 12. PubMed [citation] PMID: 22241914

559. Hobza P. Calculations on noncovalent interactions and databases of benchmark
interaction energies. Acc Chem Res. 2012 Apr 17;45(4):663-72. doi:
10.1021/ar200255p. Epub 2012 Jan 6. Review. PubMed [citation] PMID: 22225511

560. Shen K, Tang H, Jing R, Liu F, Zhou X. Application of triple-branched stent graft
for Stanford type A aortic dissection: potential risks. Eur J Cardiothorac Surg.
2012 Mar;41(3):e12-7. doi: 10.1093/ejcts/ezr259. Epub 2012 Jan 4. PubMed
[citation] PMID: 22223699

561. Dobson RJ, Hosking BC, Jacobson CL, Cotter JL, Besier RB, Stein PA, Reid SA.

Preserving new anthelmintics: a simple method for estimating faecal egg count
reduction test (FECRT) confidence limits when efficacy and/or nematode
aggregation is high. Vet Parasitol. 2012 May 4;186(1-2):79-92. doi:
10.1016/j.vetpar.2011.11.049. Epub 2011 Nov 20. Review. PubMed [citation] PMID:
22154971

562. Ishikawa T. Surgical recovery of sperm in non-obstructive azoospermia. Asian J
Androl. 2012 Jan;14(1):109-15. doi: 10.1038/aja.2011.61. Epub 2011 Nov 28.
Review. PubMed [citation] PMID: 22120931, PMCID: PMC3735145

563. Leung PC, Cheng KF, Chan YH. An innovative herbal product for the prevention of
osteoporosis. Chin J Integr Med. 2011 Oct;17(10):744-9. doi:
10.1007/s11655-011-0876-y. Epub 2011 Nov 19. PubMed [citation] PMID: 22101698

564. Concu R, Podda G, Gonzalez-Diaz H, Shen B. Review of computer-aided models for
predicting collagen stability. Curr Comput Aided Drug Des. 2011 Dec;7(4):287-303.
Review. PubMed [citation] PMID: 22050685

565. Stoevesandt D, Buerke M. [Triple rule-out computed tomography in emergency
departments]. Med Klin Intensivmed Notfmed. 2011 Oct;106(2):89-95. doi:
10.1007/s00063-011-0009-6. Review. German. PubMed [citation] PMID: 22038632

566. Keam B, Im SA, Koh Y, Han SW, Oh DY, Cho N, Kim JH, Han W, Kang KW, Moon WK, Kim
TY, Park IA, Noh DY, Chung JK, Bang YJ. Early metabolic response using FDG PET/CT
and molecular phenotypes of breast cancer treated with neoadjuvant chemotherapy.
BMC Cancer. 2011 Oct 20;11:452. doi: 10.1186/1471-2407-11-452. PubMed [citation]
PMID: 22011459, PMCID: PMC3224348

567. Boudko SP, Engel J, Bächinger HP. The crucial role of trimerization domains in
collagen folding. Int J Biochem Cell Biol. 2012 Jan;44(1):21-32. doi:
10.1016/j.biocel.2011.09.009. Epub 2011 Oct 5. Review. PubMed [citation] PMID:
22001560

568. Ekbote AV, Danda S. A case report of fibular aplasia, tibial campomelia, and
oligosyndactyly (FATCO) syndrome associated with Klinefelter syndrome and review
of the literature. Foot Ankle Spec. 2012 Feb;5(1):37-40. doi:
10.1177/1938640011422594. Epub 2011 Sep 30. Review. PubMed [citation] PMID:
21965580

569. Hofherr SE, Wiktor AE, Kipp BR, Dawson DB, Van Dyke DL. Clinical diagnostic
testing for the cytogenetic and molecular causes of male infertility: the Mayo
Clinic experience. J Assist Reprod Genet. 2011 Nov;28(11):1091-8. doi:
10.1007/s10815-011-9633-6. Epub 2011 Sep 13. PubMed [citation] PMID: 21912980,
PMCID: PMC3224174

570. Lisella JM, Bellapianta JM, Manoli A 2nd. Tarsal coalition resection with pes
planovalgus hindfoot reconstruction. J Surg Orthop Adv. 2011 Summer;20(2):102-5.
PubMed [citation] PMID: 21838070

571. Plotton I, Brosse A; Groupe Fertipreserve., Lejeune H. [Infertility treatment in
Klinefelter syndrome]. Gynecol Obstet Fertil. 2011 Sep;39(9):529-32. doi:
10.1016/j.gyobfe.2011.07.002. Epub 2011 Aug 10. Review. French. PubMed [citation]
PMID: 21835671

572. Becker HC, Johnson T. Cardiac CT for the assessment of chest pain: imaging
techniques and clinical results. Eur J Radiol. 2012 Dec;81(12):3675-9. doi:
10.1016/j.ejrad.2011.05.038. Epub 2011 Jul 28. Review. PubMed [citation] PMID:
21798681

573. Heard E, Turner J. Function of the sex chromosomes in mammalian fertility. Cold
Spring Harb Perspect Biol. 2011 Oct 1;3(10):a002675. doi:
10.1101/cshperspect.a002675. Review. PubMed [citation] PMID: 21730045, PMCID:
PMC3179336

574. Stembalska A, Łaczmańska I, Lech D. [Non-invasive prenatal test in the diagnosis
of aneuploidy 13, 18 and 21--theoretical and practical aspects].

Ginekol Pol.
2011 Feb;82(2):126-32. Review. Polish. PubMed [citation] PMID:
21574485

575. Georgiţă C, Albu F, David V, Medvedovici A, Monciu CM.
Linearization of the MS
response function: case study for metformin assay in plasma samples
for
bioequivalence purposes. Biomed Chromatogr. 2012 Feb;26(2):208-13.
doi:
10.1002/bmc.1647. Epub 2011 May 13. PubMed [citation] PMID: 21567434

576. Spigel DR, Hainsworth JD, Burris HA 3rd, Molthrop DC, Peacock N,
Kommor M,
Vazquez ER, Greco FA, Yardley DA. A pilot study of adjuvant
doxorubicin and
cyclophosphamide followed by paclitaxel and sorafenib in women with
node-positive
or high-risk early-stage breast cancer. Clin Adv Hematol Oncol. 2011
Apr;9(4):280-6. PubMed [citation] PMID: 21558987

577. Frühmesser A, Kotzot D. Chromosomal variants in klinefelter
syndrome. Sex Dev.
2011;5(3):109-23. doi: 10.1159/000327324. Epub 2011 Apr 29. Review.
PubMed
[citation] PMID: 21540567

578. Smith TO, Drew BT, Toms AP, Chojnowski AJ. The diagnostic
accuracy of X-ray
arthrography for triangular fibrocartilaginous complex injury: a
systematic
review and meta-analysis. J Hand Surg Eur Vol. 2012 Nov;37(9):879-87.
doi:
10.1177/1753193411402762. Epub 2011 Apr 5. Review. PubMed [citation]
PMID:
21467087

579. Setnik B, Roland CL, Cleveland JM, Webster L. The abuse potential
of Remoxy(®),
an extended-release formulation of oxycodone, compared with immediate-
and
extended-release oxycodone. Pain Med. 2011 Apr;12(4):618-31. doi:
10.1111/j.1526-4637.2011.01093.x. Epub 2011 Apr 4. PubMed [citation]
PMID:
21463474

580. Yoon YE, Wann S. Evaluation of acute chest pain in the emergency
department:
"triple rule-out" computed tomography angiography. Cardiol Rev. 2011
May-Jun;19(3):115-21. doi: 10.1097/CRD.0b013e31820f1501. Review.

PubMed
[citation] PMID: 21464639

581. Lahlou N, Fennoy I, Ross JL, Bouvattier C, Roger M. Clinical and hormonal status
of infants with nonmosaic XXY karyotype. Acta Paediatr. 2011 Jun;100(6):824-9.
doi: 10.1111/j.1651-2227.2011.02280.x. Epub 2011 Apr 20. Review. PubMed
[citation] PMID: 21429009, PMCID: PMC4977158

582. Bojesen A, Gravholt CH. Morbidity and mortality in Klinefelter syndrome (47,XXY).
Acta Paediatr. 2011 Jun;100(6):807-13. doi: 10.1111/j.1651-2227.2011.02274.x.
Epub 2011 Apr 6. Review. PubMed [citation] PMID: 21414026

583. Sabin MA, Werther GA, Kiess W. Genetics of obesity and overgrowth syndromes. Best
Pract Res Clin Endocrinol Metab. 2011 Feb;25(1):207-20. doi:
10.1016/j.beem.2010.09.010. Review. PubMed [citation] PMID: 21396586

584. Wikström AM, Dunkel L. Klinefelter syndrome. Best Pract Res Clin Endocrinol
Metab. 2011 Apr;25(2):239-50. doi: 10.1016/j.beem.2010.09.006. Review. PubMed
[citation] PMID: 21397196

585. Esteves SC, Agarwal A. Novel concepts in male infertility. Int Braz J Urol. 2011
Jan-Feb;37(1):5-15. Review. PubMed [citation] PMID: 21385475

586. Cury RC, Feuchtner G, Mascioli C, Fialkow J, Andrulonis P, Villanueva T, Pena CS,
Janowitz WR, Katzen BT, Ziffer JA. Cardiac CT in the emergency department:
convincing evidence, but cautious implementation. J Nucl Cardiol. 2011
Apr;18(2):331-41. doi: 10.1007/s12350-011-9356-1. Review. PubMed
[citation] PMID:
21359497

587. Hafner J, Böni T, Calcagni M, Jacomella V, Läuchli S, Rüttimann B, Siegrist B,
Stössel B, Mayer D. [Differential diagnosis of clinical relevance]. Ther Umsch.
2011 Mar;68(3):139-47. doi: 10.1024/0040-5930/a000141. Review. German. PubMed
[citation] PMID: 21360459

588. Gravholt CH, Jensen AS, Høst C, Bojesen A. Body composition,

metabolic syndrome
and type 2 diabetes in Klinefelter syndrome. Acta Paediatr. 2011
Jun;100(6):871-7. doi: 10.1111/j.1651-2227.2011.02233.x. Epub 2011 Mar
16.
Review. PubMed [citation] PMID: 21342256

589. Tartaglia N, Ayari N, Howell S, D'Epagnier C, Zeitler P. 48,XXYY,
48,XXXY and
49,XXXXY syndromes: not just variants of Klinefelter syndrome. Acta
Paediatr.
2011 Jun;100(6):851-60. doi: 10.1111/j.1651-2227.2011.02235.x. Epub
2011 Apr 8.
Review. PubMed [citation] PMID: 21342258, PMCID: PMC3314712

590. Houk CP, Rogol A, Lee PA. Fertility in men with Klinefleter
syndrome. Pediatr
Endocrinol Rev. 2010 Dec;8 Suppl 1:182-6. Review. PubMed [citation]
PMID:
21217611

591. Samango-Sprouse C. Expansion of the phenotypic profile of the
young child with
XXY. Pediatr Endocrinol Rev. 2010 Dec;8 Suppl 1:160-8. Review. PubMed
[citation]
PMID: 21217608

592. De Sanctis V, Ciccone S. Fertility preservation in adolescents
with Klinefelter's
syndrome. Pediatr Endocrinol Rev. 2010 Dec;8 Suppl 1:178-81. Review.
PubMed
[citation] PMID: 21217610

593. Lue YH, Wang C, Liu PY, Erkilla K, Swerdloff RS. Insights into
the pathogenesis
of XXY phenotype from comparison of the clinical syndrome with an
experimental
XXY mouse model. Pediatr Endocrinol Rev. 2010 Dec;8 Suppl 1:140-4.
Review. PubMed
[citation] PMID: 21217605

594. Ryan S. The adolescent and young adult with Klinefelter
syndrome:ensuring
successful transitions to adulthood. Pediatr Endocrinol Rev. 2010
Dec;8 Suppl
1:169-77. Review. PubMed [citation] PMID: 21217609

595. Rogol AD, Tartaglia N. Considerations for androgen therapy in
children and
adolescents with Klinefelter syndrome (47, XXY). Pediatr Endocrinol
Rev. 2010

Dec;8 Suppl 1:145-50. Review. PubMed [citation] PMID: 21217606

596. Tartaglia N, Cordeiro L, Howell S, Wilson R, Janusz J. The spectrum of the
behavioral phenotype in boys and adolescents 47,XXY (Klinefelter syndrome).
Pediatr Endocrinol Rev. 2010 Dec;8 Suppl 1:151-9. Review. PubMed [citation] PMID:
21217607, PMCID: PMC3740580

597. Radicioni AF, Ferlin A, Balercia G, Pasquali D, Vignozzi L, Maggi M, Foresta C,
Lenzi A. Consensus statement on diagnosis and clinical management of Klinefelter
syndrome. J Endocrinol Invest. 2010 Dec;33(11):839-50. Review. PubMed [citation]
PMID: 21293172

598. Fennoy I. Testosterone and the child (0-12 years) with Klinefelter syndrome
(47XXY): a review. Acta Paediatr. 2011 Jun;100(6):846-50. doi:
10.1111/j.1651-2227.2011.02184.x. Epub 2011 Mar 7. Review. PubMed [citation]
PMID: 21284716

599. Kamboj MK, Tareen RS. Management of nonpsychiatric medical conditions presenting
with psychiatric manifestations. Pediatr Clin North Am. 2011 Feb;58(1):219-41,
xii. doi: 10.1016/j.pcl.2010.10.008. Review. PubMed [citation] PMID: 21281858

600. Templado C, Vidal F, Estop A. Aneuploidy in human spermatozoa. Cytogenet Genome
Res. 2011;133(2-4):91-9. doi: 10.1159/000323795. Epub 2011 Jan 29. Review. PubMed
[citation] PMID: 21282942

601. Briot K, Kolta S, Flandre P, Boué F, Ngo Van P, Cohen-Codar I, Norton M,
Delfraissy JF, Roux C. Prospective one-year bone loss in treatment-naïve HIV+ men
and women on single or multiple drug HIV therapies. Bone. 2011 May
1;48(5):1133-9. doi: 10.1016/j.bone.2011.01.015. Epub 2011 Jan 26. PubMed
[citation] PMID: 21276883

602. Franson J, Baravarian B. Lateral ankle triad: the triple injury of ankle
synovitis, lateral ankle instability, and peroneal tendon tear. Clin

Podiatr Med
Surg. 2011 Jan;28(1):105-15. doi: 10.1016/j.cpm.2010.09.007. Review. PubMed
[citation] PMID: 21276521

603. Rogers IS, Banerji D, Siegel EL, Truong QA, Ghoshhajra BB, Irlbeck T, Abbara S,
Gupta R, Benenstein RJ, Choy G, Avery LL, Novelline RA, Bamberg F, Brady TJ,
Nagurney JT, Hoffmann U. Usefulness of comprehensive cardiothoracic computed
tomography in the evaluation of acute undifferentiated chest discomfort in the
emergency department (CAPTURE). Am J Cardiol. 2011 Mar 1;107(5):643-50. doi:
10.1016/j.amjcard.2010.10.039. Epub 2011 Jan 17. PubMed [citation]
PMID: 21247533

604. Brinton LA. Breast cancer risk among patients with Klinefelter syndrome. Acta
Paediatr. 2011 Jun;100(6):814-8. doi: 10.1111/j.1651-2227.2010.02131.x. Epub 2011
Jan 18. Review. PubMed [citation] PMID: 21241366, PMCID: PMC4024394

605. Girardin CM, Van Vliet G. Counselling of a couple faced with a prenatal diagnosis
of Klinefelter syndrome. Acta Paediatr. 2011 Jun;100(6):917-22. doi:
10.1111/j.1651-2227.2011.02156.x. Epub 2011 Feb 25. Review. PubMed [citation]
PMID: 21231960

606. Swerdloff RS, Lue Y, Liu PY, Erkkilä K, Wang C. Mouse model for men with
klinefelter syndrome: a multifaceted fit for a complex disorder. Acta Paediatr.
2011 Jun;100(6):892-9. doi: 10.1111/j.1651-2227.2011.02149.x. Epub 2011 Feb 14.
Review. PubMed [citation] PMID: 21226760

607. Herlihy AS, Gillam L, Halliday JL, McLachlan RI. Postnatal screening for
Klinefelter syndrome: is there a rationale? Acta Paediatr. 2011
Jun;100(6):923-33. doi: 10.1111/j.1651-2227.2011.02151.x. Epub 2011 Feb 3.
Review. PubMed [citation] PMID: 21226761

608. Ferlin A, Schipilliti M, Foresta C. Bone density and risk of osteoporosis in
Klinefelter syndrome. Acta Paediatr. 2011 Jun;100(6):878-84. doi:
10.1111/j.1651-2227.2010.02138.x. Epub 2011 Feb 10. Review. PubMed

[citation]
PMID: 21214887

609. Rey RA, Gottlieb S, Pasqualini T, Bastida MG, Grinspon RP, Campo SM, Bergadá I.
Are Klinefelter boys hypogonadal? Acta Paediatr. 2011 Jun;100(6):830-8. doi:
10.1111/j.1651-2227.2010.02137.x. Epub 2011 Feb 3. Review. PubMed [citation]
PMID: 21214886

610. Weakley SM, Wang H, Yao Q, Chen C. Expression and function of a large non-coding
RNA gene XIST in human cancer. World J Surg. 2011 Aug;35(8):1751-6. doi:
10.1007/s00268-010-0951-0. Review. PubMed [citation] PMID: 21212949, PMCID:
PMC3275083

611. Reis LO, Dias FG, Castro MA, Ferreira U. Male breast cancer. Aging Male. 2011
Jun;14(2):99-109. doi: 10.3109/13685538.2010.535048. Epub 2011 Jan 4. Review.
PubMed [citation] PMID: 21204612

612. Kliesch S, Zitzmann M, Behre HM. [Fertility in patients with Klinefelter syndrome
(47,XXY)]. Urologe A. 2011 Jan;50(1):26-32. doi: 10.1007/s00120-010-2443-0.
Review. German. PubMed [citation] PMID: 21207006

613. Kolta S, Flandre P, Van PN, Cohen-Codar I, Valantin MA, Pintado C, Morlat P, Boué
F, Rode R, Norton M, Knysz B, Briot K, Roux C, Delfraissy JF. Fat tissue
distribution changes in HIV-infected patients treated with lopinavir/ritonavir.
Results of the MONARK trial. Curr HIV Res. 2011 Jan;9(1):31-9. PubMed [citation]
PMID: 21198431

614. Cordts EB, Christofolini DM, Dos Santos AA, Bianco B, Barbosa CP. Genetic aspects
of premature ovarian failure: a literature review. Arch Gynecol Obstet. 2011
Mar;283(3):635-43. doi: 10.1007/s00404-010-1815-4. Epub 2010 Dec 29. Review.
PubMed [citation] PMID: 21188402

615. Krissak R, Henzler T, Prechel A, Reichert M, Gruettner J,

Sueseelbeck T,
Schoenberg SO, Fink C. Triple-rule-out dual-source CT angiography of patients
with acute chest pain: dose reduction potential of 100 kV scanning. Eur J Radiol.
2012 Dec;81(12):3691-6. doi: 10.1016/j.ejrad.2010.11.021. Epub 2010 Dec 15.
PubMed [citation] PMID: 21163600

616. Mariolis-Sapsakos T, Theodoropoulos G, Flessas II, Orfanos F, Orfanos N,
Konstantinou EA, Zagouri F, Vlachodimitropoulos D, Zografos GC. Lobular breast
cancer in men: case report and review of the literature. Onkologie.
2010;33(12):698-700. doi: 10.1159/000322224. Epub 2010 Nov 26. Review. Erratum
in: Onkologie. 2011;34(1-2):45. Konstadinou, Evangelos [corrected to
Konstantinou, Evangelos A]. PubMed [citation] PMID: 21124042

617. Martínez-Gimeno C, Rodríguez-Delgado LE, Perera-Molinero A, Trujillo Mdel C,
Chivite A, Maeso MC, Aguirre-Jaime A. A new method for the prediction of cervical
node metastases in squamous cell carcinoma of the oral cavity: a combination of
Martínez-Gimeno Scoring System and clinical palpation. J Craniomaxillofac Surg.
2011 Oct;39(7):534-7. doi: 10.1016/j.jcms.2010.10.027. Epub 2010 Nov 26. PubMed
[citation] PMID: 21112791

618. Stahl PJ, Stember DS, Hsiao W, Schlegel PN. Indications and strategies for
fertility preservation in men. Clin Obstet Gynecol. 2010 Dec;53(4):815-27. doi:
10.1097/GRF.0b013e3181f980b3. Review. PubMed [citation] PMID: 21048448

619. Close S, Smaldone A, Reame N, Fennoy I. Klinefelter syndrome: awareness and index
of suspicion. J Pediatr Nurs. 2010 Dec;25(6):592-4. doi:
10.1016/j.pedn.2010.08.002. Review. No abstract available. PubMed [citation]
PMID: 21035026

620. Gleicher N, Barad DH. The FMR1 gene as regulator of ovarian recruitment and
ovarian reserve. Obstet Gynecol Surv. 2010 Aug;65(8):523-30. doi:
10.1097/OGX.0b013e3181f8bdda. Review. PubMed [citation] PMID: 20955631

621. Giltay JC, Maiburg MC. Klinefelter syndrome: clinical and

molecular aspects.
Expert Rev Mol Diagn. 2010 Sep;10(6):765-76. doi: 10.1586/erm.10.63.
Review.
PubMed [citation] PMID: 20843200

622. Velasco G, Savarese V, Sandorfi N, Jimenez SA, Jabbour S. 46, XX
SRY-positive
male syndrome presenting with primary hypogonadism in the setting of
scleroderma.
Endocr Pract. 2011 Jan-Feb;17(1):95-8. doi: 10.4158/EP10184.CR.
Review. PubMed
[citation] PMID: 20841307

623. Sala-Pérez S, Vázquez-Delgado E, Rodríguez-Baeza A, Gay-Escoda C.
Bifid
mandibular condyle: a disorder in its own right? J Am Dent Assoc. 2010
Sep;141(9):1076-85. Review. PubMed [citation] PMID: 20807906

624. Buinauskaite E, Buinauskiene J, Kucinskiene V, Strazdiene D,
Valiukeviciene S.
Incontinentia pigmenti in a male infant with Klinefelter syndrome: a
case report
and review of the literature. Pediatr Dermatol. 2010 Sep-
Oct;27(5):492-5. doi:
10.1111/j.1525-1470.2010.01261.x. Epub 2010 Aug 27. Review. PubMed
[citation]
PMID: 20807362

625. Tramper-Stranders GA, Wolfs TF, van Haren Noman S, van Aalderen
WM, Nagelkerke
AF, Nuijsink M, Kimpen JL, van der Ent CK. Controlled trial of cycled
antibiotic
prophylaxis to prevent initial Pseudomonas aeruginosa infection in
children with
cystic fibrosis. Thorax. 2010 Oct;65(10):915-20. doi: 10.1136/
thx.2009.126128.
Epub 2010 Aug 20. PubMed [citation] PMID: 20729233

626. Plotton I, Brosse A, Lejeune H. [Is it useful to modify the care
of Klinefelter's
syndrome to improve the chances of paternity?]. Ann Endocrinol
(Paris). 2010
Dec;71(6):494-504. doi: 10.1016/j.ando.2010.06.001. Epub 2010 Aug 19.
Review.
French. PubMed [citation] PMID: 20727517

627. Hofer C, Schmalfeldt B, Gschwend JE, Herkommer K. [Male breast
cancer: a
challenge for urologists]. Urologe A. 2010 Sep;49(9):1142, 1144-8.
doi:

10.1007/s00120-010-2356-y. Review. German. PubMed [citation] PMID: 20706705

628. Thilo C, Hanley M, Bastarrika G, Ruzsics B, Schoepf UJ. Integrative computed tomographic imaging of cardiac structure, function, perfusion, and viability. Cardiol Rev. 2010 Sep-Oct;18(5):219-29. doi: 10.1097/CRD.0b013e3181d6b87a. Review. PubMed [citation] PMID: 20699669

629. Koide T. [Application of collagen-like triple-helical peptides to biochemical studies elucidating the collagen structure and functions]. Seikagaku. 2010 Jun;82(6):474-83. Review. Japanese. No abstract available. PubMed [citation] PMID: 20662255

630. Forti G, Corona G, Vignozzi L, Krausz C, Maggi M. Klinefelter's syndrome: a clinical and therapeutical update. Sex Dev. 2010 Sep;4(4-5):249-58. doi: 10.1159/000316604. Epub 2010 Jul 21. Review. PubMed [citation] PMID: 20664188

631. Ali N, Rizwi F, Iqbal A, Rashid A. Induced remote ischemic pre-conditioning on ischemia-reperfusion injury in patients undergoing coronary artery bypass. J Coll Physicians Surg Pak. 2010 Jul;20(7):427-31. doi: 07.2010/JCPSP.427431. PubMed [citation] PMID: 20642939

632. Lo-Castro A, D'Agati E, Curatolo P. ADHD and genetic syndromes. Brain Dev. 2011 Jun;33(6):456-61. doi: 10.1016/j.braindev.2010.05.011. Epub 2010 Jun 22. Review. PubMed [citation] PMID: 20573461

633. Lambert SM, Vilain EJ, Kolon TF. A practical approach to ambiguous genitalia in the newborn period. Urol Clin North Am. 2010 May;37(2):195-205. doi: 10.1016/j.ucl.2010.03.014. Review. PubMed [citation] PMID: 20569798

634. Sharma RK, Voelker DJ, Sharma RK, Singh VN, Bhatt G, Moazazi M, Nash T, Reddy HK. Coronary computed tomographic angiography (CCTA) in community hospitals: "current and emerging role". Vasc Health Risk Manag. 2010 May 25;6:307-16.

Review. PubMed
[citation] PMID: 20531948, PMCID: PMC2879291

635. Kapoor S. Cutaneous manifestations of systemic conditions associated with
gynecomastia. Skinmed. 2010 Mar-Apr;8(2):87-92; quiz 92. Review. PubMed
[citation] PMID: 20527139

636. Yin A, Swerdloff R. Treating hypogonadism in younger males. Expert Opin
Pharmacother. 2010 Jun;11(9):1529-40. doi: 10.1517/14656561003742947. Review.
PubMed [citation] PMID: 20482304

637. Sjödin A, Gasteyger C, Nielsen AL, Raben A, Mikkelsen JD, Jensen JK, Meier D,
Astrup A. The effect of the triple monoamine reuptake inhibitor tesofensine on
energy metabolism and appetite in overweight and moderately obese men. Int J Obes
(Lond). 2010 Nov;34(11):1634-43. doi: 10.1038/ijo.2010.87. Epub 2010 May 18.
PubMed [citation] PMID: 20479765

638. Ganou M, Grouios G, Koidou I, Alevriadou A. The concept of anomalous cerebral
lateralization in Klinefelter syndrome. Appl Neuropsychol. 2010 Apr;17(2):144-52.
doi: 10.1080/09084281003715683. Review. PubMed [citation] PMID: 20467956

639. Tartaglia NR, Howell S, Sutherland A, Wilson R, Wilson L. A review of trisomy X
(47,XXX). Orphanet J Rare Dis. 2010 May 11;5:8. doi: 10.1186/1750-1172-5-8.
Review. PubMed [citation] PMID: 20459843, PMCID: PMC2883963

640. Schertler T, Feuchtner G, Frauenfelder T, Alkadhi H, Leschka S. [Use of
multislice CT in the evaluation of patients with acute chest pain]. Praxis (Bern
1994). 2010 Apr 28;99(9):545-52. doi: 10.1024/1661-8157/a000104. Review. German.
PubMed [citation] PMID: 20449822

641. Hsiao EM, Rybicki FJ, Steigner M. CT coronary angiography: 256-slice and
320-detector row scanners. Curr Cardiol Rep. 2010 Jan;12(1):68-75. doi:

10.1007/s11886-009-0075-z. Review. PubMed [citation] PMID: 20425186, PMCID:
PMC2893879

642. Rovenský J, Imrich R, Lazúrová I, Payer J. Rheumatic diseases and Klinefelter's
syndrome. Ann N Y Acad Sci. 2010 Apr;1193:1-9. doi:
10.1111/j.1749-6632.2009.05292.x. Review. PubMed [citation] PMID: 20398000

643. Radicioni AF, De Marco E, Gianfrilli D, Granato S, Gandini L, Isidori AM, Lenzi
A. Strategies and advantages of early diagnosis in Klinefelter's syndrome. Mol
Hum Reprod. 2010 Jun;16(6):434-40. doi: 10.1093/molehr/gaq027. Epub 2010 Apr 14.
Review. PubMed [citation] PMID: 20392711

644. Lazaridis C, Naval N. Risk factors and medical management of vasospasm after
subarachnoid hemorrhage. Neurosurg Clin N Am. 2010 Apr;21(2):353-64. doi:
10.1016/j.nec.2009.10.006. Review. PubMed [citation] PMID: 20380975

645. Al-Arfaj HF. Klinefelter's syndrome and rheumatoid arthritis: report of a case
and review of the literature. Int J Rheum Dis. 2010 Feb 1;13(1):86-8. doi:
10.1111/j.1756-185X.2009.01452.x. Review. PubMed [citation] PMID: 20374390

646. Zhang HJ, Jin BF. [Azoospermia factor and male infertility]. Zhonghua Nan Ke Xue.
2010 Feb;16(2):166-9. Review. Chinese. PubMed [citation] PMID: 20369704

647. Furtado AD, Adraktas DD, Brasic N, Cheng SC, Ordovas K, Smith WS, Lewin MR, Chun
K, Chien JD, Schaeffer S, Wintermark M. The triple rule-out for acute ischemic
stroke: imaging the brain, carotid arteries, aorta, and heart. AJNR Am J
Neuroradiol. 2010 Aug;31(7):1290-6. doi: 10.3174/ajnr.A2075. Epub 2010 Apr 1.
PubMed [citation] PMID: 20360341, PMCID: PMC7965468

648. Vignozzi L, Corona G, Forti G, Jannini EA, Maggi M. Clinical and therapeutic
aspects of Klinefelter's syndrome: sexual function. Mol Hum Reprod. 2010

Jun;16(6):418-24. doi: 10.1093/molehr/gaq022. Epub 2010 Mar 26.
Review. PubMed
[citation] PMID: 20348547

649. Ferlin A, Schipilliti M, Di Mambro A, Vinanzi C, Foresta C.
Osteoporosis in
Klinefelter's syndrome. Mol Hum Reprod. 2010 Jun;16(6):402-10. doi:
10.1093/molehr/gaq026. Epub 2010 Mar 27. Review. PubMed [citation]
PMID: 20348548

650. Wistuba J. Animal models for Klinefelter's syndrome and their
relevance for the
clinic. Mol Hum Reprod. 2010 Jun;16(6):375-85. doi: 10.1093/molehr/
gaq024. Epub
2010 Mar 21. Review. PubMed [citation] PMID: 20308053

651. Di Mambro A, Ferlin A, De Toni L, Selice R, Caretta N, Foresta C.
Endothelial
progenitor cells as a new cardiovascular risk factor in Klinefelter's
syndrome.
Mol Hum Reprod. 2010 Jun;16(6):411-7. doi: 10.1093/molehr/gaq015. Epub
2010 Mar
11. Review. PubMed [citation] PMID: 20228052

652. Bojesen A, Høst C, Gravholt CH. Klinefelter's syndrome, type 2
diabetes and the
metabolic syndrome: the impact of body composition. Mol Hum Reprod.
2010
Jun;16(6):396-401. doi: 10.1093/molehr/gaq016. Epub 2010 Mar 15.
Review. PubMed
[citation] PMID: 20231162

653. Tüttelmann F, Gromoll J. Novel genetic aspects of Klinefelter's
syndrome. Mol Hum
Reprod. 2010 Jun;16(6):386-95. doi: 10.1093/molehr/gaq019. Epub 2010
Mar 12.
Review. PubMed [citation] PMID: 20228051

654. Chen XS, Nie XQ, Chen CM, Wu JY, Wu J, Lu JS, Shao ZM, Shen ZZ,
Shen KW. Weekly
paclitaxel plus carboplatin is an effective nonanthracycline-
containing regimen
as neoadjuvant chemotherapy for breast cancer. Ann Oncol. 2010
May;21(5):961-7.
doi: 10.1093/annonc/mdq041. Epub 2010 Mar 8. PubMed [citation] PMID:
20211870

655. Verri A, Cremante A, Clerici F, Destefani V, Radicioni A.
Klinefelter's syndrome
and psychoneurologic function. Mol Hum Reprod. 2010 Jun;16(6):425-33.

doi:
10.1093/molehr/gaq018. Epub 2010 Mar 2. Review. PubMed [citation]
PMID: 20197378

656. Gómez-Raposo C, Zambrana Tévar F, Sereno Moyano M, López Gómez M, Casado E. Male
breast cancer. Cancer Treat Rev. 2010 Oct;36(6):451-7. doi:
10.1016/j.ctrv.2010.02.002. Epub 2010 Mar 2. Review. PubMed [citation]
PMID:
20193984

657. Yadav M, Singhal P, Goswami S, Sharma P, Shrivastav PS. Microdetermination of
emtricitabine in human plasma by liquid chromatography/tandem mass spectrometry.
J AOAC Int. 2009 Nov-Dec;92(6):1681-9. PubMed [citation] PMID:
20166586

658. Bağci S, Müller A, Franz A, Heydweiller A, Berg C, Nöthen MM, Bartmann P, Reutter
H. Intestinal atresia, encephalocele, and cardiac malformations in infants with
47,XXX: Expansion of the phenotypic spectrum and a review of the literature.
Fetal Diagn Ther. 2010;27(2):113-7. doi: 10.1159/000284929. Epub 2010 Feb 16.
Review. PubMed [citation] PMID: 20160426

659. Straver ME, Aukema TS, Olmos RA, Rutgers EJ, Gilhuijs KG, Schot ME, Vogel WV,
Peeters MJ. Feasibility of FDG PET/CT to monitor the response of axillary lymph
node metastases to neoadjuvant chemotherapy in breast cancer patients. Eur J Nucl
Med Mol Imaging. 2010 Jun;37(6):1069-76. doi: 10.1007/
s00259-009-1343-2. Epub
2010 Feb 4. PubMed [citation] PMID: 20130860, PMCID: PMC2869017

660. Fullerton G, Hamilton M, Maheshwari A. Should non-mosaic Klinefelter syndrome men
be labelled as infertile in 2009? Hum Reprod. 2010 Mar;25(3):588-97. doi:
10.1093/humrep/dep431. Epub 2010 Jan 19. Review. PubMed [citation]
PMID: 20085911

661. Kamps WA, van der Pal-de Bruin KM, Veerman AJ, Fiocco M, Bierings M, Pieters R.
Long-term results of Dutch Childhood Oncology Group studies for children with
acute lymphoblastic leukemia from 1984 to 2004. Leukemia. 2010

Feb;24(2):309-19.
doi: 10.1038/leu.2009.258. Epub 2009 Dec 17. PubMed [citation] PMID:
20016528

662. Lenroot RK, Lee NR, Giedd JN. Effects of sex chromosome
aneuploidies on brain
development: evidence from neuroimaging studies. Dev Disabil Res Rev.
2009;15(4):318-27. doi: 10.1002/ddrr.86. Review. PubMed [citation]
PMID:
20014372, PMCID: PMC2996824

663. Steinman K, Ross J, Lai S, Reiss A, Hoeft F. Structural and
functional
neuroimaging in Klinefelter (47,XXY) syndrome: a review of the
literature and
preliminary results from a functional magnetic resonance imaging study
of
language. Dev Disabil Res Rev. 2009;15(4):295-308. doi: 10.1002/
ddrr.84. Review.
PubMed [citation] PMID: 20014370, PMCID: PMC2876340

664. Boada R, Janusz J, Hutaff-Lee C, Tartaglia N. The cognitive
phenotype in
Klinefelter syndrome: a review of the literature including genetic and
hormonal
factors. Dev Disabil Res Rev. 2009;15(4):284-94. doi: 10.1002/ddrr.83.
Review.
PubMed [citation] PMID: 20014369, PMCID: PMC3056507

665. Edouard T, Tauber M. [Delayed puberty]. Arch Pediatr. 2010
Feb;17(2):195-200.
doi: 10.1016/j.arcped.2009.09.017. Epub 2009 Nov 4. Review. French.
PubMed
[citation] PMID: 19892534

666. Demirhan O, Pazarbaşi A, Tanriverdi N, Aridoğan A, Karahan D. The
clinical
effects of isochromosome Xq in Klinefelter syndrome: report of a case
and review
of literature. Genet Couns. 2009;20(3):235-42. Review. PubMed
[citation] PMID:
19852429

667. Satoh M, Wakabayashi O, Araya Y, Jinushi E, Yoshida F. [Autopsy
case of von
Recklinghausen's disease associated with lung cancer, gastrointestinal
stromal
tumor of the stomach, and duodenal carcinoid tumor]. Nihon Kokyuki
Gakkai Zasshi.
2009 Sep;47(9):798-804. Review. Japanese. PubMed [citation] PMID:

19827584

668. Halpern EJ. Triple-rule-out CT angiography for evaluation of acute chest pain and
possible acute coronary syndrome. Radiology. 2009 Aug;252(2):332-45. doi:
10.1148/radiol.2522082335. Review. PubMed [citation] PMID: 19703877

669. Stark B, Avrahami G, Nirel R, Abramov A, Attias D, Ballin A, Bielorai B, Burstein
Y, Gavriel H, Elhasid R, Kapelushnik J, Sthoeger D, Toren A, Wientraub M, Yaniv
I, Izraeli S. Extended triple intrathecal therapy in children with T-cell acute
lymphoblastic leukaemia: a report from the Israeli National ALL-Studies. Br J
Haematol. 2009 Oct;147(1):113-24. doi: 10.1111/j.1365-2141.2009.07853.x. Epub
2009 Aug 19. Review. PubMed [citation] PMID: 19694717

670. Gordon MK, Hahn RA. Collagens. Cell Tissue Res. 2010 Jan;339(1):247-57. doi:
10.1007/s00441-009-0844-4. Epub 2009 Aug 20. Review. PubMed [citation] PMID:
19693541, PMCID: PMC2997103

671. McCavert M, O'Donnell ME, Aroori S, Badger SA, Sharif MA, Crothers JG, Spence RA.
Ultrasound is a useful adjunct to mammography in the assessment of breast tumours
in all patients. Int J Clin Pract. 2009 Nov;63(11):1589-94. doi:
10.1111/j.1742-1241.2009.02102.x. Epub 2009 Aug 14. Review. PubMed [citation]
PMID: 19686337

672. Bastarrika G, Thilo C, Headden GF, Zwerner PL, Costello P, Schoepf UJ. Cardiac CT
in the assessment of acute chest pain in the emergency department. AJR Am J
Roentgenol. 2009 Aug;193(2):397-409. doi: 10.2214/AJR.08.2265. Review. PubMed
[citation] PMID: 19620436

673. Rabijewski M, Zgliczyński W. [Pathogenesis, evaluation and treatment of
hypogonadism in men]. Endokrynol Pol. 2009 May-Jun;60(3):222-33. Review. Polish.
PubMed [citation] PMID: 19569024

674. Otter M, Schrander-Stumpel CT, Curfs LM. Triple X syndrome: a

review of the
literature. Eur J Hum Genet. 2010 Mar;18(3):265-71. doi: 10.1038/
ejhg.2009.109.
Epub 2009 Jul 1. Review. PubMed [citation] PMID: 19568271, PMCID:
PMC2987225

675. Yang JC, Yang YF, Uang YS, Lin CJ, Wang TH. Pharmacokinetic-
pharmacodynamic
analysis of the role of CYP2C19 genotypes in short-term rabeprazole-
based triple
therapy against Helicobacter pylori. Br J Clin Pharmacol. 2009
May;67(5):503-10.
doi: 10.1111/j.1365-2125.2009.03393.x. Epub 2009 Feb 23. PubMed
[citation] PMID:
19552744, PMCID: PMC2686066

676. Bonuccelli S, Muscelli E, Gastaldelli A, Barsotti E, Astiarraga
BD, Holst JJ,
Mari A, Ferrannini E. Improved tolerance to sequential glucose loading
(Staub-Traugott effect): size and mechanisms. Am J Physiol Endocrinol
Metab. 2009
Aug;297(2):E532-7. doi: 10.1152/ajpendo.00127.2009. Epub 2009 Jun 16.
PubMed
[citation] PMID: 19531643

677. Veldhuis JD, Hudson SB, Erickson D, Bailey JN, Reynolds GA,
Bowers CY. Relative
effects of estrogen, age, and visceral fat on pulsatile growth hormone
secretion
in healthy women. Am J Physiol Endocrinol Metab. 2009
Aug;297(2):E367-74. doi:
10.1152/ajpendo.00230.2009. Epub 2009 May 26. PubMed [citation] PMID:
19470834,
PMCID: PMC2724113

678. Sawalha AH, Harley JB, Scofield RH. Autoimmunity and
Klinefelter's syndrome: when
men have two X chromosomes. J Autoimmun. 2009 Aug;33(1):31-4. doi:
10.1016/j.jaut.2009.03.006. Epub 2009 May 22. Review. PubMed
[citation] PMID:
19464849, PMCID: PMC2885450

679. Jeanty C, Turner C. Prenatal diagnosis of double aneuploidy,
48,XXY,+21, and
review of the literature. J Ultrasound Med. 2009 May;28(5):673-81.
Review. No
abstract available. PubMed [citation] PMID: 19389908

680. Wilson RW 2nd, Snyder AC, Dorman JC. Analysis of seated and
standing triple

Wingate tests. J Strength Cond Res. 2009 May;23(3):868-73. doi:
10.1519/JSC.0b013e31819d0932. PubMed [citation] PMID: 19387391

681. Sato K, Takeuchi H, Kubota T. Pathology of intracranial germ cell tumors. Prog
Neurol Surg. 2009;23:59-75. doi: 10.1159/000210053. Epub 2009 Mar 23. Review.
PubMed [citation] PMID: 19329861

682. Poetz O, Hoeppe S, Templin MF, Stoll D, Joos TO. Proteome wide screening using
peptide affinity capture. Proteomics. 2009 Mar;9(6):1518-23. doi:
10.1002/pmic.200800842. Review. PubMed [citation] PMID: 19294621

683. Yadav M, Rao R, Kurani H, Singhal P, Goswami S, Shrivastav PS. Application of a
rapid and selective method for the simultaneous determination of protease
inhibitors, lopinavir and ritonavir in human plasma by UPLC-ESI-MS/MS for
bioequivalence study in Indian subjects. J Pharm Biomed Anal. 2009 May
1;49(4):1115-22. doi: 10.1016/j.jpba.2009.02.010. Epub 2009 Feb 20. PubMed
[citation] PMID: 19282124

684. Haram K, Svendsen E, Abildgaard U. The HELLP syndrome: clinical issues and
management. A Review. BMC Pregnancy Childbirth. 2009 Feb 26;9:8. doi:
10.1186/1471-2393-9-8. Review. PubMed [citation] PMID: 19245695, PMCID:
PMC2654858

685. Walsh TJ, Pera RR, Turek PJ. The genetics of male infertility. Semin Reprod Med.
2009 Mar;27(2):124-36. doi: 10.1055/s-0029-1202301. Epub 2009 Feb 26. Review.
PubMed [citation] PMID: 19247914

686. Schlegel PN. Nonobstructive azoospermia: a revolutionary surgical approach and
results. Semin Reprod Med. 2009 Mar;27(2):165-70. doi: 10.1055/s-0029-1202305.
Epub 2009 Feb 26. Review. PubMed [citation] PMID: 19247918

687. Paduch DA, Bolyakov A, Cohen P, Travis A. Reproduction in men with Klinefelter
syndrome: the past, the present, and the future. Semin Reprod Med. 2009
Mar;27(2):137-48. doi: 10.1055/s-0029-1202302. Epub 2009 Feb 26. Review. PubMed

[citation] PMID: 19247915

688. Urbania TH, Hope MD, Huffaker SD, Reddy GP. Role of computed tomography in the
evaluation of acute chest pain. J Cardiovasc Comput Tomogr. 2009 Jan-Feb;3(1
Suppl):S13-22. doi: 10.1016/j.jcct.2008.11.004. Epub 2008 Dec 6. Review. PubMed
[citation] PMID: 19203744

689. Shapiro MD. Is the "triple rule-out" study an appropriate indication for
cardiovascular CT? J Cardiovasc Comput Tomogr. 2009 Mar-Apr;3(2):100-3. doi:
10.1016/j.jcct.2008.12.011. Epub 2009 Jan 13. Review. PubMed [citation] PMID:
19201674

690. Bellaaj H, Sandi HS, Kammoun H, Kallel C, Kassar O, Elloumi M. [Klinefelter
syndrome and acute myeloblastic leukaemia]. Presse Med. 2009 Jun;38(6):1019-22.
doi: 10.1016/j.lpm.2008.05.025. Epub 2009 Jan 22. Review. French. No abstract
available. PubMed [citation] PMID: 19167185

691. Faraoni-Romano JJ, Turssi CP, Serra MC. Effect of a 10% carbamide peroxide on
wear resistance of enamel and dentine: in situ study. J Dent. 2009
Apr;37(4):273-8. doi: 10.1016/j.jdent.2008.12.001. Epub 2009 Jan 20. PubMed
[citation] PMID: 19157672

692. Maksimova MIu, Briukhov VV, Timerbaeva SL, Kistenev BA, Rebrova OIu, Suslina ZA.
[Effectiveness of cerebrolysin in hypertensive supratentorial intracranial
hemorrhages: results of a randomized triple blind placebo-controled study]. Zh
Nevrol Psikhiatr Im S S Korsakova. 2009;109(1):20-6. Russian. PubMed [citation]
PMID: 19156082

693. Alaraj A, Charbel FT, Amin-Hanjani S. Peri-operative measures for treatment and
prevention of cerebral vasospasm following subarachnoid hemorrhage. Neurol Res.
2009 Jul;31(6):651-9. doi: 10.1179/174313209X382395. Epub 2009 Jan 7. Review.
PubMed [citation] PMID: 19133166

694. Boer DR, Canals A, Coll M. DNA-binding drugs caught in action: the latest 3D
pictures of drug-DNA complexes. Dalton Trans. 2009 Jan 21;(3):399-414. doi:
10.1039/b809873p. Epub 2008 Nov 7. Review. PubMed [citation] PMID: 19122895

695. Jacobeit JW, Kliesch S. [Gynecomastia: diagnosis and therapy]. Dtsch Med
Wochenschr. 2008 Dec;133(49):2567-71. doi: 10.1055/s-0028-1105855. Epub 2008 Nov
27. Review. German. No abstract available. PubMed [citation] PMID: 19039712

696. Frauenfelder T, Appenzeller P, Karlo C, Scheffel H, Desbiolles L, Stolzmann P,
Marincek B, Alkadhi H, Schertler T. Triple rule-out CT in the emergency
department: protocols and spectrum of imaging findings. Eur Radiol. 2009
Apr;19(4):789-99. doi: 10.1007/s00330-008-1231-3. Epub 2008 Nov 18. Review.
PubMed [citation] PMID: 19015860

697. Xia XY, Yang B, Cui YX, Huang YF. [Genetic causes of male infertility]. Zhonghua
Nan Ke Xue. 2008 Sep;14(9):837-41. Review. Chinese. PubMed [citation] PMID:
19004117

698. Okuyama K. Revisiting the molecular structure of collagen. Connect Tissue Res.
2008;49(5):299-310. doi: 10.1080/03008200802325110. Review. PubMed [citation]
PMID: 18991083

699. Huang WJ, Yen PH. Genetics of spermatogenic failure. Sex Dev. 2008;2(4-5):251-9.
doi: 10.1159/000152041. Epub 2008 Nov 5. Review. PubMed [citation]
PMID: 18987499

700. Marco EJ, Skuse DH. Autism-lessons from the X chromosome. Soc Cogn Affect
Neurosci. 2006 Dec;1(3):183-93. doi: 10.1093/scan/nsl028. Review. PubMed
[citation] PMID: 18985105, PMCID: PMC2555419

701. Abe I. Engineering of plant polyketide biosynthesis. Chem Pharm Bull (Tokyo).

2008 Nov;56(11):1505-14. Review. PubMed [citation] PMID: 18981598

702. Tüttelmann F, Gromoll J, Kliesch S. [Genetics of male infertility]. Urologe A.
2008 Dec;47(12):1561-2, 1564-7. doi: 10.1007/s00120-008-1804-4. Review. German.
PubMed [citation] PMID: 18953522

703. Elsharkawi-Welt K, Hepp J, Scharffetter-Kochanek K. [Genetic causes of impaired
wound healing. Rare differential diagnosis of the non-healing wound]. Hautarzt.
2008 Nov;59(11):893-903. doi: 10.1007/s00105-008-1591-2. Review. German. PubMed
[citation] PMID: 18936901

704. Lee HY, Yoo SM, White CS. Coronary CT angiography in emergency department
patients with acute chest pain: triple rule-out protocol versus dedicated
coronary CT angiography. Int J Cardiovasc Imaging. 2009 Mar;25(3):319-26. doi:
10.1007/s10554-008-9375-4. Epub 2008 Oct 14. Review. PubMed [citation] PMID:
18853277

705. Paduch DA, Fine RG, Bolyakov A, Kiper J. New concepts in Klinefelter syndrome.
Curr Opin Urol. 2008 Nov;18(6):621-7. doi: 10.1097/MOU.0b013e32831367c7. Review.
PubMed [citation] PMID: 18832949

706. Crespi B. Genomic imprinting in the development and evolution of psychotic
spectrum conditions. Biol Rev Camb Philos Soc. 2008 Nov;83(4):441-93. doi:
10.1111/j.1469-185X.2008.00050.x. Epub 2008 Sep 9. Review. PubMed [citation]
PMID: 18783362

707. Ebbing M, Bleie Ø, Ueland PM, Nordrehaug JE, Nilsen DW, Vollset SE, Refsum H,
Pedersen EK, Nygård O. Mortality and cardiovascular events in patients treated
with homocysteine-lowering B vitamins after coronary angiography: a randomized
controlled trial. JAMA. 2008 Aug 20;300(7):795-804. doi: 10.1001/jama.300.7.795.
PubMed [citation] PMID: 18714059

708. Höckner M, Pinggera GM, Günther B, Sergi C, Fauth C, Erdel M, Kotzot D.
Unravelling the parental origin and mechanism of formation of the 47,XY,i(X)(q10)
Klinefelter karyotype variant. Fertil Steril. 2008 Nov;90(5):2009.e13-7. doi:
10.1016/j.fertnstert.2008.05.054. Epub 2008 Aug 6. Review. PubMed [citation]
PMID: 18687426

709. Cury RC, Feutchner G, Pena CS, Janowitz WR, Katzen BT, Ziffer JA. Acute chest
pain imaging in the emergency department with cardiac computed tomography
angiography. J Nucl Cardiol. 2008 Jul-Aug;15(4):564-75. doi:
10.1016/j.nuclcard.2008.05.006. Review. PubMed [citation] PMID: 18674724

710. Nabedryk E, Breton J. Coupling of electron transfer to proton uptake at the Q(B)
site of the bacterial reaction center: a perspective from FTIR difference
spectroscopy. Biochim Biophys Acta. 2008 Oct;1777(10):1229-48. doi:
10.1016/j.bbabio.2008.06.012. Epub 2008 Jul 11. Review. PubMed [citation] PMID:
18671937

711. Yang ZJ, Zhang YR, Chen B, Zhang SL, Jia EZ, Wang LS, Zhu TB, Li CJ, Wang H,
Huang J, Cao KJ, Ma WZ, Wu B, Wang LS, Wu CT. Phase I clinical trial on
intracoronary administration of Ad-hHGF treating severe coronary artery disease.
Mol Biol Rep. 2009 Jul;36(6):1323-9. doi: 10.1007/s11033-008-9315-3. Epub 2008
Jul 22. PubMed [citation] PMID: 18649012

712. Matsutani H, Sano T, Kondo T, Morita H, Arai T, Sekine T, Takase S, Oida A,
Fukazawa H, Suguta M, Kondo M, Kodama T, Orihara T, Yamada N, Tsuyuki M, Narula
J. ECG-edit function in multidetector-row computed tomography coronary
arteriography for patients with arrhythmias. Circ J. 2008 Jul;72(7):1071-8.
PubMed [citation] PMID: 18577814

713. Ahmad I, Sansom OJ, Leung HY. Advances in mouse models of prostate cancer. Expert
Rev Mol Med. 2008 Jun 9;10:e16. doi: 10.1017/S1462399408000689. Review. PubMed

[citation] PMID: 18538039

714. Vialard F, Pellestor F. [Benefit of human gamete cytogenetics: results and
perspectives]. Pathol Biol (Paris). 2008 Sep;56(6):388-99. doi:
10.1016/j.patbio.2008.04.012. Epub 2008 Jun 4. Review. French. PubMed [citation]
PMID: 18534785

715. Hagenäs L. [Normal and deviating puberty in boys]. Tidsskr Nor Laegeforen. 2008
May 29;128(11):1284-8. Review. Norwegian. PubMed [citation] PMID: 18511972

716. Ekerhovd E. [Infertility treatment in men with Klinefelter syndrome]. Tidsskr Nor
Laegeforen. 2008 May 29;128(11):1281-3. Review. Norwegian. PubMed [citation]
PMID: 18511971

717. Wikström AM, Dunkel L. Testicular function in Klinefelter syndrome. Horm Res.
2008;69(6):317-26. doi: 10.1159/000117387. Epub 2008 Mar 17. Review. PubMed
[citation] PMID: 18504390

718. Joseph M. Endodontic treatment in three taurodontic teeth associated with 48,XXXY
Klinefelter syndrome: a review and case report. Oral Surg Oral Med Oral Pathol
Oral Radiol Endod. 2008 May;105(5):670-7. doi: 10.1016/j.tripleo.2007.11.015.
Review. PubMed [citation] PMID: 18442747

719. Foresta C, Zuccarello D, Garolla A, Ferlin A. Role of hormones, genes, and
environment in human cryptorchidism. Endocr Rev. 2008 Aug;29(5):560-80. doi:
10.1210/er.2007-0042. Epub 2008 Apr 24. Review. PubMed [citation]
PMID: 18436703

720. Bouxsein ML. Technology insight: noninvasive assessment of bone strength in
osteoporosis. Nat Clin Pract Rheumatol. 2008 Jun;4(6):310-8. doi:
10.1038/ncprheum0798. Epub 2008 Apr 22. Review. PubMed [citation]
PMID: 18431371

721. Chantot-Bastaraud S, Ravel C, Siffroi JP. Underlying karyotype abnormalities in
IVF/ICSI patients. Reprod Biomed Online. 2008 Apr;16(4):514-22.

Review. PubMed
[citation] PMID: 18413060

722. Nordenström A, Nordenskjöld A, Frisén L, Wedell A, Ritzén M. [Boy or girl--don't
ever guess! Diagnosis and treatment of sex differentiation disorders].
Lakartidningen. 2008 Feb 27-Mar 4;105(9):629-33. Review. Swedish. No abstract
available. PubMed [citation] PMID: 18376707

723. Ruel M, Beanlands RS, Lortie M, Chan V, Camack N, deKemp RA, Suuronen EJ, Rubens
FD, DaSilva JN, Sellke FW, Stewart DJ, Mesana TG. Concomitant treatment with oral
L-arginine improves the efficacy of surgical angiogenesis in patients with severe
diffuse coronary artery disease: the Endothelial Modulation in Angiogenic Therapy
randomized controlled trial. J Thorac Cardiovasc Surg. 2008 Apr;135(4):762-70,
770.e1. doi: 10.1016/j.jtcvs.2007.09.073. Epub 2008 Mar 18. PubMed [citation]
PMID: 18374753

724. Rist C, Johnson TR, Becker CR, Reiser MF, Nikolaou K. New applications for
noninvasive cardiac imaging: dual-source computed tomography. Eur Radiol. 2007
Dec;17 Suppl 6:F16-25. Review. PubMed [citation] PMID: 18376453

725. Shingfield KJ, Arölä A, Ahvenjärvi S, Vanhatalo A, Toivonen V, Griinari JM,
Huhtanen P. Ruminal infusions of cobalt-EDTA reduce mammary delta9-desaturase
index and alter milk fatty acid composition in lactating cows. J Nutr. 2008
Apr;138(4):710-7. PubMed [citation] PMID: 18356325

726. Sipiczki M. Interspecies hybridization and recombination in Saccharomyces wine
yeasts. FEMS Yeast Res. 2008 Nov;8(7):996-1007. doi:
10.1111/j.1567-1364.2008.00369.x. Epub 2008 Mar 18. Review. PubMed [citation]
PMID: 18355270

727. Aleman A, Swart M, van Rijn S. Brain imaging, genetics and emotion. Biol Psychol.
2008 Sep;79(1):58-69. doi: 10.1016/j.biopsycho.2008.01.009. Epub 2008 Feb 2.
Review. PubMed [citation] PMID: 18329779

728. Mistri HN, Jangid AG, Pudage A, Shrivastav P. HPLC-ESI-MS/MS validated method for
simultaneous quantification of zopiclone and its metabolites, N-desmethyl
zopiclone and zopiclone-N-oxide in human plasma. J Chromatogr B Analyt Technol
Biomed Life Sci. 2008 Mar 15;864(1-2):137-48. doi: 10.1016/j.jchromb.2008.02.004.
Epub 2008 Feb 17. PubMed [citation] PMID: 18313371

729. Brodsky B, Thiagarajan G, Madhan B, Kar K. Triple-helical peptides: an approach
to collagen conformation, stability, and self-association. Biopolymers. 2008
May;89(5):345-53. doi: 10.1002/bip.20958. Review. PubMed [citation] PMID:
18275087

730. Yuan SM, Shinfeld A, Raanani E. Configurations and classifications of composite
arterial grafts in coronary bypass surgery. J Cardiovasc Med (Hagerstown). 2008
Jan;9(1):3-14. doi: 10.2459/JCM.0b013e3280110628. Review. PubMed [citation] PMID:
18268413

731. Ørstavik KH. [Genetic causes of male infertility]. Tidsskr Nor Laegeforen. 2008
Jan 31;128(3):324-6. Review. Norwegian. PubMed [citation] PMID: 18264159

732. Karaman A, Kabalar E. Double aneuploidy in a Turkish child: Down-Klinefelter
syndrome. Congenit Anom (Kyoto). 2008 Mar;48(1):45-7. doi:
10.1111/j.1741-4520.2007.00174.x. Review. Erratum in: Congenit Anom (Kyoto). 2008
Jun;48(2):101. PubMed [citation] PMID: 18230121

733. Rostami N, Keshtkar-Jahromi M, Rahnavardi M, Keshtkar-Jahromi M, Esfahani FS.
Effect of eradication of Helicobacter pylori on platelet recovery in patients
with chronic idiopathic thrombocytopenic purpura: a controlled trial. Am J
Hematol. 2008 May;83(5):376-81. doi: 10.1002/ajh.21125. PubMed [citation] PMID:
18183613

734. Perrin A, Morel F, Moy L, Colleu D, Amice V, De Braekeleer M.

Study of aneuploidy
in large-headed, multiple-tailed spermatozoa: case report and review of the
literature. Fertil Steril. 2008 Oct;90(4):1201.e13-7. doi:
10.1016/j.fertnstert.2007.09.013. Epub 2007 Dec 31. Review. PubMed [citation]
PMID: 18166187

735. Gallagher MJ, Raff GL. Use of multislice CT for the evaluation of emergency room
patients with chest pain: the so-called "triple rule-out". Catheter Cardiovasc
Interv. 2008 Jan 1;71(1):92-9. Review. PubMed [citation] PMID: 18098208

736. Spark RF. Testosterone, diabetes mellitus, and the metabolic syndrome. Curr Urol
Rep. 2007 Nov;8(6):467-71. Review. PubMed [citation] PMID: 18042326

737. Morris JK, Alberman E, Scott C, Jacobs P. Is the prevalence of Klinefelter
syndrome increasing? Eur J Hum Genet. 2008 Feb;16(2):163-70. Epub 2007 Nov 14.
PubMed [citation] PMID: 18000523

738. Lin L, Ferraz-de-Souza B, Achermann JC. Genetic disorders involving adrenal
development. Endocr Dev. 2007;11:36-46. doi: 10.1159/000111056. Review. PubMed
[citation] PMID: 17986825

739. Kim SH, Lim HK, Lee WJ, Choi D, Park CK. Scirrhous hepatocellular carcinoma:
comparison with usual hepatocellular carcinoma based on CT-pathologic features
and long-term results after curative resection. Eur J Radiol. 2009
Jan;69(1):123-30. Epub 2007 Oct 31. PubMed [citation] PMID: 17976942

740. Wang JM, Liu L, Irwin RW, Chen S, Brinton RD. Regenerative potential of
allopregnanolone. Brain Res Rev. 2008 Mar;57(2):398-409. Epub 2007 Sep 14.
Review. PubMed [citation] PMID: 17931704

741. Ogino W, Takeshima Y, Nishiyama A, Yagi M, Oka N, Matsuo M. Mosaic tetrasomy 9p
case with the phenotype mimicking Klinefelter syndrome and hyporesponse of
gonadotropin-stimulated testosterone production. Kobe J Med Sci.
2007;53(4):143-50. Review. PubMed [citation] PMID: 17932453

742. Zhan WH, Jiang ZM, Tang Y, Wu YP, Liu JW, Zhang YJ, Chen W, Liu T, Yao C. [Impact
of hypocaloric and hypo-nitrogen parenteral nutrition on clinical outcome in
postoperative patients: a multi-center randomized controlled trial of 120 cases].
Zhonghua Yi Xue Za Zhi. 2007 Jul 3;87(25):1729-33. Chinese. PubMed [citation]
PMID: 17919374

743. Maskill MP, Loveland JD, Mendicino RW, Saltrick K, Catanzariti AR. Triple
arthrodesis for the adult-acquired flatfoot deformity. Clin Podiatr Med Surg.
2007 Oct;24(4):765-78, x. Review. PubMed [citation] PMID: 17908643

744. Choy EH, Smith CM, Farewell V, Walker D, Hassell A, Chau L, Scott DL; CARDERA
(Combination Anti-Rheumatic Drugs in Early Rheumatoid Arhritis) Trial Group..
Factorial randomised controlled trial of glucocorticoids and combination disease
modifying drugs in early rheumatoid arthritis. Ann Rheum Dis. 2008
May;67(5):656-63. Epub 2007 Sep 3. PubMed [citation] PMID: 17768173

745. Loewinger L, Budoff MJ. New advances in cardiac computed tomography. Curr Opin
Cardiol. 2007 Sep;22(5):408-12. Review. PubMed [citation] PMID: 17762541

746. Fernández Rivero JM, Rocha Ramírez JL, Villanueva Sáenz E, Sierra Montenegro E,
Rojas Illanes M. [Anorectal tuberculosis. Case report]. Rev Gastroenterol Mex.
2007 Jan-Mar;72(1):40-2. Review. Spanish. PubMed [citation] PMID: 17685199

747. Lee YS, Cheng AW, Ahmed SF, Shaw NJ, Hughes IA. Genital anomalies in
Klinefelter's syndrome. Horm Res. 2007;68(3):150-5. Epub 2007 Jul 19. Review.
PubMed [citation] PMID: 17641549

748. Ferlin A, Raicu F, Gatta V, Zuccarello D, Palka G, Foresta C. Male infertility:
role of genetic background. Reprod Biomed Online. 2007 Jun;14(6):734-45. Review.
PubMed [citation] PMID: 17579990

749. Hamoir X, Salovic D, Bouziane T, Kirsch J. Dual source CT: cardio-pulmonary
applications. JBR-BTR. 2007 Mar-Apr;90(2):77-9. Review. PubMed [citation] PMID:
17555062

750. Bhasin S. Approach to the infertile man. J Clin Endocrinol Metab. 2007
Jun;92(6):1995-2004. Review. PubMed [citation] PMID: 17554051

751. Seemann MD. Detection of metastases from gastrointestinal neuroendocrine tumors:
prospective comparison of 18F-TOCA PET, triple-phase CT, and PET/CT. Technol
Cancer Res Treat. 2007 Jun;6(3):213-20. PubMed [citation] PMID: 17535030

752. Witters I, Fryns JR. Fetal nuchal translucency thickness. Genet Couns.
2007;18(1):1-7. Review. PubMed [citation] PMID: 17515296

753. Limkakeng AT, Halpern E, Takakuwa KM. Sixty-four-slice multidetector computed
tomography: the future of ED cardiac care. Am J Emerg Med. 2007 May;25(4):450-8.
Review. PubMed [citation] PMID: 17499666

754. Verchot-Lubicz J, Ye CM, Bamunusinghe D. Molecular biology of potexviruses:
recent advances. J Gen Virol. 2007 Jun;88(Pt 6):1643-1655. doi:
10.1099/vir.0.82667-0. Review. PubMed [citation] PMID: 17485523

755. Yoshino A, Katayama Y, Watanabe T, Ogino A, Ohta T, Komine C, Yokoyama T,
Fukushima T, Hirota H. Apoplexy accompanying pituitary adenoma as a complication
of preoperative anterior pituitary function tests. Acta Neurochir (Wien). 2007
Jun;149(6):557-65; discussion 565. Epub 2007 Apr 30. Review. PubMed [citation]
PMID: 17468811

756. Jeudy J, White CS. Evaluation of acute chest pain in the emergency department:
utility of multidetector computed tomography. Semin Ultrasound CT MR. 2007
Apr;28(2):109-14. Review. PubMed [citation] PMID: 17432765

757. Choolani M, Ho SS, Razvi K, Ponnusamy S, Baig S, Fisk NM, Biswas A; Rapid

Molecular Testing in Prenatal Diagnosis Group.. FastFISH: technique for
ultrarapid fluorescence in situ hybridization on uncultured amniocytes yielding
results within 2 h of amniocentesis. Mol Hum Reprod. 2007 Jun;13(6):355-9. Epub
2007 Apr 12. PubMed [citation] PMID: 17430982

758. Bojesen A, Gravholt CH. Klinefelter syndrome in clinical practice. Nat Clin Pract
Urol. 2007 Apr;4(4):192-204. Review. PubMed [citation] PMID: 17415352

759. Julow J, Major T, Mangel L, Bajzik G, Viola A. Image fusion analysis of
volumetric changes after interstitial low-dose-rate iodine-125 irradiation of
supratentorial low-grade gliomas. Radiat Res. 2007 Apr;167(4):438-44. PubMed
[citation] PMID: 17388696

760. Chen CP. Chromosomal abnormalities associated with omphalocele. Taiwan J Obstet
Gynecol. 2007 Mar;46(1):1-8. Review. PubMed [citation] PMID: 17389182

761. Richmond EJ, Rogol AD. Male pubertal development and the role of androgen
therapy. Nat Clin Pract Endocrinol Metab. 2007 Apr;3(4):338-44. Review. PubMed
[citation] PMID: 17377616

762. Hansen L, Tausche E, Hietschold V, Hotan T, Lagravère M, Harzer W.
Skeletally-anchored rapid maxillary expansion using the Dresden Distractor. J
Orofac Orthop. 2007 Mar;68(2):148-58. English, German. PubMed [citation] PMID:
17372711

763. LaClair SM. Reconstruction of the varus ankle from soft-tissue procedures with
osteotomy through arthrodesis. Foot Ankle Clin. 2007 Mar;12(1):153-76, x. Review.
PubMed [citation] PMID: 17350516

764. Schussler JM, Smith ER. Sixty-four-slice computed tomographic coronary
angiography: will the "triple rule out" change chest pain evaluation in the ED?
Am J Emerg Med. 2007 Mar;25(3):367-75. Review. PubMed [citation] PMID:
17349915

765. Lu AW, Zheng SS, Wu J, Liang TB, Wang WL, Shen Y, Zhang M, Shi SH, Wu YS. [Dual,
triple, and quadruple oral tacrolimus-based immunosuppression regimens after
orthotopic liver transplantation: a randomised comparative study of regimens].
Zhonghua Yi Xue Za Zhi. 2006 Dec 26;86(48):3389-92. Chinese. PubMed [citation]
PMID: 17313848

766. Mehldau K. [Klinefelter syndrome]. Kinderkrankenschwester. 2006 Dec;25(12):519-25. Review. German. No abstract available. PubMed [citation] PMID:
17236686

767. Gooren LJ, de Ronde W. [Some new aspects of the Klinefelter syndrome]. Ned
Tijdschr Geneeskd. 2006 Dec 9;150(49):2693-6. Review. Dutch. PubMed [citation]
PMID: 17194004

768. Mitraki A, Papanikolopoulou K, Van Raaij MJ. Natural triple beta-stranded fibrous
folds. Adv Protein Chem. 2006;73:97-124. Review. PubMed [citation]
PMID: 17190612

769. Dufourg MN, Landman-Parker J, Auclerc MF, Schmitt C, Perel Y, Michel G, Levy P,
Couillault G, Gandemer V, Tabone MD, Demeocq F, Vannier JP, Leblanc T, Leverger
G, Baruchel A. Age and high-dose methotrexate are associated to clinical acute
encephalopathy in FRALLE 93 trial for acute lymphoblastic leukemia in children.
Leukemia. 2007 Feb;21(2):238-47. Epub 2006 Dec 14. PubMed [citation] PMID:
17170721

770. Blaney M, Shen V, Kerner JA, Jacobs BR, Gray S, Armfield J, Semba CP; CAPS
Investigators.. Alteplase for the treatment of central venous catheter occlusion
in children: results of a prospective, open-label, single-arm study (The Cathflo
Activase Pediatric Study). J Vasc Interv Radiol. 2006 Nov;17(11 Pt 1):1745-51.
PubMed [citation] PMID: 17142704

771. Leitinger B, Hohenester E. Mammalian collagen receptors. Matrix

Biol. 2007
Apr;26(3):146-55. Epub 2006 Nov 10. Review. PubMed [citation] PMID:
17141492

772. Georgiou I, Syrrou M, Pardalidis N, Karakitsios K, Mantzavinos T, Giotitsas N,
Loutradis D, Dimitriadis F, Saito M, Miyagawa I, Tzoumis P, Sylakos A, Kanakas N,
Moustakareas T, Baltogiannis D, Touloupides S, Giannakis D, Fatouros M, Sofikitis
N. Genetic and epigenetic risks of intracytoplasmic sperm injection method. Asian
J Androl. 2006 Nov;8(6):643-73. Review. PubMed [citation] PMID:
17111067

773. Rovenský J. Rheumatic diseases and Klinefelter's syndrome. Autoimmun Rev. 2006
Nov;6(1):33-6. Epub 2006 Apr 19. Review. PubMed [citation] PMID:
17110314

774. Liu F, Xu Y, Rui L, Gao S, Dong H, Guo Q. Liquid chromatography/ tandem mass
spectrometry assay for the quantification of troxerutin in human plasma. Rapid
Commun Mass Spectrom. 2006;20(23):3522-6. PubMed [citation] PMID:
17072901

775. Escribá MJ, Martín J, Rubio C, Valbuena D, Remohí J, Pellicer A, Simón C.
Heteroparental blastocyst production from microsurgically corrected
tripronucleated human embryos. Fertil Steril. 2006 Dec;86(6):1601-7. Epub 2006
Oct 24. PubMed [citation] PMID: 17067583

776. Triplitt C, Glass L, Miyazaki Y, Wajcberg E, Gastaldelli A, De Filippis E,
Cersosimo E, DeFronzo RA. Comparison of glargine insulin versus rosiglitazone
addition in poorly controlled type 2 diabetic patients on metformin plus
sulfonylurea. Diabetes Care. 2006 Nov;29(11):2371-7. PubMed [citation] PMID:
17065670

777. Visootsak J, Graham JM Jr. Klinefelter syndrome and other sex chromosomal
aneuploidies. Orphanet J Rare Dis. 2006 Oct 24;1:42. Review. PubMed [citation]
PMID: 17062147, PMCID: PMC1634840

778. Agrawal A, Ayantunde AA, Rampaul R, Robertson JF. Male breast cancer: a review of
clinical management. Breast Cancer Res Treat. 2007 May;103(1):11-21. Epub 2006
Oct 11. Review. PubMed [citation] PMID: 17033919

779. Gusbin N, Verloes A, Daly A, Beckers A. [Tall stature: some classical syndromes].
Rev Med Liege. 2006 Jul-Aug;61(7-8):572-80. Review. French. PubMed [citation]
PMID: 17020230

780. Murphey MD, Gibson MS, Jennings BT, Crespo-Rodríguez AM, Fanburg-Smith J,
Gajewski DA. From the archives of the AFIP: Imaging of synovial sarcoma with
radiologic-pathologic correlation. Radiographics. 2006 Sep-Oct;26(5):1543-65.
Review. PubMed [citation] PMID: 16973781

781. Quallich S. Examining male infertility. Urol Nurs. 2006 Aug;26(4):277-88; quiz
289. Review. PubMed [citation] PMID: 16939045

782. Wikström AM, Bay K, Hero M, Andersson AM, Dunkel L. Serum insulin-like factor 3
levels during puberty in healthy boys and boys with Klinefelter syndrome. J Clin
Endocrinol Metab. 2006 Nov;91(11):4705-8. Epub 2006 Aug 22. PubMed [citation]
PMID: 16926256

783. Serebrovska ZA, Serebrovskaya TV, Pyle RL, Di Pietro ML. Transmission of male
infertility and intracytoplasmic sperm injection (mini-review). Fiziol Zh (1994).
2006;52(3):110-8. Review. PubMed [citation] PMID: 16909765

784. Fujieda K. [Klinefelter syndrome]. Nihon Rinsho. 2006 Jun 28;Suppl 2:487-90.
Review. Japanese. No abstract available. PubMed [citation] PMID: 16817447

785. Shima H, Yamamoto S. [Hermaphroditism]. Nihon Rinsho. 2006 Jun 28;Suppl 2:561-72.
Review. Japanese. No abstract available. PubMed [citation] PMID: 16817465

786. Itoh N. [XYY syndrome]. Nihon Rinsho. 2006 Jun 28;Suppl 2:525-7. Review.

Japanese. No abstract available. PubMed [citation] PMID: 16817457

787. Dambrauskas Z, Pundzius J, Barauskas G. Predicting development of infected
necrosis in acute necrotizing pancreatitis. Medicina (Kaunas). 2006;42(6):441-9.
Review. PubMed [citation] PMID: 16816537

788. Fujita N, Kaito M, Kai M, Sugimoto R, Tanaka H, Horiike S, Konishi M, Iwasa M,
Watanabe S, Adachi Y. Effects of bezafibrate in patients with chronic hepatitis C
virus infection: combination with interferon and ribavirin. J Viral Hepat. 2006
Jul;13(7):441-8. PubMed [citation] PMID: 16792537

789. Giedd JN, Clasen LS, Lenroot R, Greenstein D, Wallace GL, Ordaz S, Molloy EA,
Blumenthal JD, Tossell JW, Stayer C, Samango-Sprouse CA, Shen D, Davatzikos C,
Merke D, Chrousos GP. Puberty-related influences on brain development. Mol Cell
Endocrinol. 2006 Jul 25;254-255:154-62. Epub 2006 Jun 9. Review. PubMed
[citation] PMID: 16765510

790. Hammoud AO, Gibson M, Peterson CM, Hamilton BD, Carrell DT. Obesity and male
reproductive potential. J Androl. 2006 Sep-Oct;27(5):619-26. Epub 2006 Jun 2.
Review. No abstract available. PubMed [citation] PMID: 16751621

791. Gundermann KJ, Godehardt E, Ulbrich M. [The efficacy of a combination herbal
medicine in the treatment of functional dyspepsia. Meta-analysis of randomized
double-blind studies on the basis of a valid gastrointestinal symptom profile].
MMW Fortschr Med. 2004 Aug 5;146 Suppl 2:71-6. German. PubMed [citation] PMID:
16739362

792. Krausz C, Forti G. Sperm cryopreservation in male infertility due to genetic
disorders. Cell Tissue Bank. 2006;7(2):105-12. Review. PubMed [citation] PMID:
16732413

793. Kiewe P, Hasmüller S, Kahlert S, Heinrigs M, Rack B, Marmé A, Korfel A, Jäger M,

Lindhofer H, Sommer H, Thiel E, Untch M. Phase I trial of the
trifunctional
anti-HER2 x anti-CD3 antibody ertumaxomab in metastatic breast cancer.
Clin
Cancer Res. 2006 May 15;12(10):3085-91. PubMed [citation] PMID:
16707606

794. Hall H, Hunt P, Hassold T. Meiosis and sex chromosome aneuploidy:
how meiotic
errors cause aneuploidy; how aneuploidy causes meiotic errors. Curr
Opin Genet
Dev. 2006 Jun;16(3):323-9. Epub 2006 May 2. Review. PubMed [citation]
PMID:
16647844

795. Borah B, Dufresne TE, Ritman EL, Jorgensen SM, Liu S, Chmielewski
PA, Phipps RJ,
Zhou X, Sibonga JD, Turner RT. Long-term risedronate treatment
normalizes
mineralization and continues to preserve trabecular architecture:
sequential
triple biopsy studies with micro-computed tomography. Bone. 2006
Aug;39(2):345-52. Epub 2006 Mar 29. PubMed [citation] PMID: 16571382

796. Hassan-Alin M, Andersson T, Niazi M, Liljeblad M, Persson BA,
Röhss K. Studies on
drug interactions between esomeprazole, amoxicillin and clarithromycin
in healthy
subjects. Int J Clin Pharmacol Ther. 2006 Mar;44(3):119-27. PubMed
[citation]
PMID: 16550734

797. Rovenský J. Rheumatic diseases and Klinefelter's syndrome. Isr
Med Assoc J. 2006
Feb;8(2):119-21. Review. No abstract available. PubMed [citation]
PMID: 16544736

798. Lawton TW, Cronin JB, Lindsell RP. Effect of interrepetition rest
intervals on
weight training repetition power output. J Strength Cond Res. 2006
Feb;20(1):172-6. PubMed [citation] PMID: 16503678

799. Willis MJ, Bird LM, Dell'aquilla M, Jones MC. Natural history of
prenatally
diagnosed 46,X,isodicentric Y. Prenat Diagn. 2006 Feb;26(2):134-7.
Review. PubMed
[citation] PMID: 16463293

800. Antos D, Schneider-Brachert W, Bästlein E, Hänel C, Haferland C,
Buchner M, Meier

E, Trump F, Stolte M, Lehn N, Bayerdörffer E. 7-day triple therapy of
Helicobacter pylori infection with levofloxacin, amoxicillin, and
high-dose
esomeprazole in patients with known antimicrobial sensitivity.
Helicobacter. 2006
Feb;11(1):39-45. PubMed [citation] PMID: 16423088

801. Zhao L, Gray L, Leonardi-Bee J, Weaver CS, Heptinstall S, Bath
PM. Effect of
aspirin, clopidogrel and dipyridamole on soluble markers of vascular
function in
normal volunteers and patients with prior ischaemic stroke. Platelets.
2006
Mar;17(2):100-4. PubMed [citation] PMID: 16421011

802. Wang RX, Liu RZ. [Advances in research on sperm chromosomes in
male infertility].
Zhonghua Nan Ke Xue. 2005 Dec;11(12):941-3. Review. Chinese. PubMed
[citation]
PMID: 16398371

803. Braun JP, Schroeder T, Buehner S, Jain U, Döpfmer U, Schuster J,
Bas S, Schimke
I, Dohmen PM, Lochs H, Konertz W, Spies C. Small-dose epoprostenol
decreases
systemic oxygen consumption and splanchnic oxygen extraction during
normothermic
cardiopulmonary bypass. Anesth Analg. 2006 Jan;102(1):17-24. PubMed
[citation]
PMID: 16368799

804. Lee CH, Kuo SW, Hung YJ, Hsieh CH, He CT, Yang TC, Lian WC, Chyi-
Fan S, Pei D.
The effect of testosterone supplement on insulin sensitivity, glucose
effectiveness, and acute insulin response after glucose load in male
type 2
diabetics. Endocr Res. 2005;31(2):139-48. PubMed [citation] PMID:
16353672

805. Wattendorf DJ, Muenke M. Klinefelter syndrome. Am Fam Physician.
2005 Dec
1;72(11):2259-62. Review. PubMed [citation] PMID: 16342850

806. Glander HJ. [Infertility in the Klinefelter syndrome]. MMW
Fortschr Med. 2005 Nov
10;147(45):39-41. Review. German. PubMed [citation] PMID: 16320651

807. Douet-Guilbert N, Bris MJ, Amice V, Marchetti C, Delobel B, Amice
J, Braekeleer
MD, Morel F. Interchromosomal effect in sperm of males with

translocations:
report of 6 cases and review of the literature. Int J Androl. 2005 Dec;28(6):372-9. Review. PubMed [citation] PMID: 16300670

808. Ponzielli R, Katz S, Barsyte-Lovejoy D, Penn LZ. Cancer therapeutics: targeting
the dark side of Myc. Eur J Cancer. 2005 Nov;41(16):2485-501. Epub 2005 Oct 20.
Review. PubMed [citation] PMID: 16243519

809. Shah PM, Leong B, Babu SC, Goyal AM, Mateo RB. Cerebrovascular events associated
with infusion through arterially malpositioned triple-lumen catheter: report of
three cases and review of literature. Cardiol Rev. 2005 Nov-Dec;13(6):304-8.
Review. PubMed [citation] PMID: 16230888

810. Percy AK, Lane JB. Rett syndrome: model of neurodevelopmental disorders. J Child
Neurol. 2005 Sep;20(9):718-21. Review. PubMed [citation] PMID: 16225824

811. Boll M, Schink B, Messerschmidt A, Kroneck PM. Novel bacterial molybdenum and
tungsten enzymes: three-dimensional structure, spectroscopy, and reaction
mechanism. Biol Chem. 2005 Oct;386(10):999-1006. Review. PubMed [citation] PMID:
16218872

812. Kishore U, Greenhough TJ, Waters P, Shrive AK, Ghai R, Kamran MF, Bernal AL, Reid
KB, Madan T, Chakraborty T. Surfactant proteins SP-A and SP-D: structure,
function and receptors. Mol Immunol. 2006 Mar;43(9):1293-315. Epub 2005 Oct 5.
Review. PubMed [citation] PMID: 16213021

813. Kaal EC, Taphoorn MJ, Vecht CJ. Symptomatic management and imaging of brain
metastases. J Neurooncol. 2005 Oct;75(1):15-20. Review. PubMed [citation] PMID:
16215812

814. Mau-Holzmann UA. Somatic chromosomal abnormalities in infertile men and women.
Cytogenet Genome Res. 2005;111(3-4):317-36. Review. PubMed [citation] PMID:
16192711

815. Ferlin A, Garolla A, Foresta C. Chromosome abnormalities in sperm of individuals
with constitutional sex chromosomal abnormalities. Cytogenet Genome Res.
2005;111(3-4):310-6. Review. PubMed [citation] PMID: 16192710

816. Aksglaede L, Wikström AM, Rajpert-De Meyts E, Dunkel L, Skakkebaek NE, Juul A.
Natural history of seminiferous tubule degeneration in Klinefelter syndrome. Hum
Reprod Update. 2006 Jan-Feb;12(1):39-48. Epub 2005 Sep 19. Review. PubMed
[citation] PMID: 16172111

817. Kamalipour H, Bagheri M, Kamali K, Taleie A, Yarmohammadi H. Lateral neck
radiography for prediction of difficult orotracheal intubation. Eur J
Anaesthesiol. 2005 Sep;22(9):689-93. PubMed [citation] PMID: 16163916

818. Medraś M, Trzmiel A, Grabowski M, Bohdanowicz-Pawlak A, Zagodzka E. [Inhibin B--a
marker of the function of male gonad]. Ginekol Pol. 2005 Jun;76(6):484-90.
Review. Polish. PubMed [citation] PMID: 16149268

819. Idigbe EO, Adewole TA, Eisen G, Kanki P, Odunukwe NN, Onwujekwe DI, Audu RA,
Araoyinbo ID, Onyewuche JI, Salu OB, Adedoyin JA, Musa AZ. Management of HIV-1
infection with a combination of nevirapine, stavudine, and lamivudine: a
preliminary report on the Nigerian antiretroviral program. J Acquir Immune Defic
Syndr. 2005 Sep 1;40(1):65-9. PubMed [citation] PMID: 16123684

820. Tubiana JM, Biour M, Bavoux F, Kalifa G, Dion E. [Imaging features of iatrogenic
drug disorders]. J Radiol. 2005 May;86(5 Pt 2):558-66. Review. French. PubMed
[citation] PMID: 16106794

821. Ricard-Blum S, Ruggiero F. The collagen superfamily: from the extracellular
matrix to the cell membrane. Pathol Biol (Paris). 2005 Sep;53(7):430-42. Epub
2005 Jan 20. Review. PubMed [citation] PMID: 16085121

822. Yesilova Z, Oktenli C, Sanisoglu SY, Musabak U, Cakir E, Ozata M, Dagalp K.

Evaluation of insulin sensitivity in patients with Klinefelter's syndrome: a
hyperinsulinemic euglycemic clamp study. Endocrine. 2005 Jun;27(1):11-5. PubMed
[citation] PMID: 16077165

823. Kurzawski M, Dziewanowski K, Gawrońska-Szklarz B, Domański L, Droździk M. The
impact of thiopurine s-methyltransferase polymorphism on azathioprine-induced
myelotoxicity in renal transplant recipients. Ther Drug Monit. 2005
Aug;27(4):435-41. PubMed [citation] PMID: 16044099

824. Krusinskiene V, Alvesalo L, Sidlauskas A. The craniofacial complex in 47, XXX
females. Eur J Orthod. 2005 Aug;27(4):396-401. Erratum in: Eur J Orthod. 2005
Oct;27(5):532. Krusinskie, Viktorija [corrected to Krusinskiene, Viktorija].
PubMed [citation] PMID: 16043476

825. Kurbacher CM, Cree IA. Chemosensitivity testing using microplate adenosine
triphosphate-based luminescence measurements. Methods Mol Med. 2005;110:101-20.
Review. PubMed [citation] PMID: 15901931

826. Brandes BM, Mesrobian HG. Evaluation and management of genital anomalies in two
patients with Klinefelter syndrome and review of literature. Urology. 2005
May;65(5):976-9. Review. PubMed [citation] PMID: 15882735

827. Labek G, Auersperg V, Ziernhöld M, Poulios N, Böhler N. [Influence of local
anesthesia and energy level on the clinical outcome of extracorporeal shock
wave-treatment of chronic plantar fasciitis]. Z Orthop Ihre Grenzgeb. 2005
Mar-Apr;143(2):240-6. German. PubMed [citation] PMID: 15849646

828. Brodsky B, Persikov AV. Molecular structure of the collagen triple helix. Adv
Protein Chem. 2005;70:301-39. Review. PubMed [citation] PMID: 15837519

829. van Rijn S, Aleman A, Swaab H, Kahn RS. Neurobiology of emotion and high risk for
schizophrenia: role of the amygdala and the X-chromosome. Neurosci Biobehav Rev.
2005 May;29(3):385-97. Epub 2004 Dec 22. Review. PubMed [citation]

PMID: 15820545

830. Jockenhövel F. Testosterone therapy--what, when and to whom? Aging Male. 2004
Dec;7(4):319-24. Review. PubMed [citation] PMID: 15799128

831. Zhou Q, Cui YX. [Intracytoplasmic sperm injection for Klinefelter patients and
the risk of chromosome anomaly in the patients' offspring]. Zhonghua Nan Ke Xue.
2005 Feb;11(2):149-51. Review. Chinese. PubMed [citation] PMID: 15755040

832. Beyer T, Antoch G, Bockisch A, Stattaus J. Optimized intravenous contrast
administration for diagnostic whole-body 18F-FDG PET/CT. J Nucl Med. 2005
Mar;46(3):429-35. PubMed [citation] PMID: 15750155

833. Yin OQ, Lam SS, Chow MS. Simultaneous determination of paracetamol and
dextropropoxyphene in human plasma by liquid chromatography/tandem mass
spectrometry: application to clinical bioequivalence studies. Rapid Commun Mass
Spectrom. 2005;19(6):767-74. PubMed [citation] PMID: 15714600

834. Rose AB, Merke DP, Clasen LS, Rosenthal MA, Wallace GL, Vaituzis AC, Fields JD,
Giedd JN. Effects of hormones and sex chromosomes on stress-influenced regions of
the developing pediatric brain. Ann N Y Acad Sci. 2004 Dec;1032:231-3. PubMed
[citation] PMID: 15677417

835. Weiss JR, Moysich KB, Swede H. Epidemiology of male breast cancer. Cancer
Epidemiol Biomarkers Prev. 2005 Jan;14(1):20-6. Review. PubMed [citation] PMID:
15668471

836. Wielgos M, Bablok L, Fracki S, Czaplicki M, Marianowski L. The naloxone test in
Klinefelter syndrome. Neuro Endocrinol Lett. 2004 Dec;25(6):438-42. PubMed
[citation] PMID: 15665807

837. Ho CF, Wu MH, Wu HM, Chang CY, Chen MC, Chou TY. Comparison of auto-moving table
contrast-enhanced 3-D MRA and iodinated contrast-enhanced DSA for

evaluating the
lower-extremity arteries. J Chin Med Assoc. 2004 Oct;67(10):511-20.
PubMed
[citation] PMID: 15648286

838. Duong M, Dinoulos JG, Gupta A, Bryk T, Saps M, Di Lorenzo C,
Sveen A, Waseem M,
Kin LL. Index of suspicion. Pediatr Rev. 2005 Jan;26(1):23-33. Review.
No
abstract available. PubMed [citation] PMID: 15629904

839. Machatschek JN, Schrauder A, Helm F, Schrappe M, Claviez A. Acute
lymphoblastic
leukemia and Klinefelter syndrome in children: two cases and review of
the
literature. Pediatr Hematol Oncol. 2004 Oct-Nov;21(7):621-6. Review.
PubMed
[citation] PMID: 15626018

840. Basak M, Joseph S, Joshi S, Sawant S. Comparative bioavailability
of a novel
timed release and powder-filled glucosamine sulfate formulation--a
multi-dose,
randomized, crossover study. Int J Clin Pharmacol Ther. 2004
Nov;42(11):597-601.
PubMed [citation] PMID: 15598026

841. Trautner MC, Aladangady N, Maalouf E, Misra D. Jejunal atresia in
an infant with
triple-X syndrome. J Matern Fetal Neonatal Med. 2004
Sep;16(3):198-200. Review.
PubMed [citation] PMID: 15590447

842. Lopez R, Payoux P, Gantet P, Esquerré JP, Boutault F, Paoli JR.
Multimodal image
registration for localization of sentinel nodes in head and neck
squamous cell
carcinoma. J Oral Maxillofac Surg. 2004 Dec;62(12):1497-504. PubMed
[citation]
PMID: 15573349

843. Nista EC, Candelli M, Cremonini F, Cazzato IA, Zocco MA,
Franceschi F, Cammarota
G, Gasbarrini G, Gasbarrini A. Bacillus clausii therapy to reduce
side-effects of
anti-Helicobacter pylori treatment: randomized, double-blind, placebo
controlled
trial. Aliment Pharmacol Ther. 2004 Nov 15;20(10):1181-8. PubMed
[citation] PMID:
15569121

844. Eldar-Geva T, Milatiner D, Halle D. [Androgen receptor and male infertility].
Harefuah. 2004 Jun;143(6):432-9, 461. Review. Hebrew. PubMed [citation] PMID:
15524101

845. Le Gall JY, Jouanolle AM, Fergelot P, Mosser J, David V. [Genetics of hereditary
iron overload]. Bull Acad Natl Med. 2004;188(2):247-62; discussion 262-3. Review.
French. PubMed [citation] PMID: 15506716

846. Shen D, Liu D, Liu H, Clasen L, Giedd J, Davatzikos C. Automated morphometric
study of brain variation in XXY males. Neuroimage. 2004 Oct;23(2):648-53. PubMed
[citation] PMID: 15488414

847. Denschlag D, Tempfer C, Kunze M, Wolff G, Keck C. Assisted reproductive
techniques in patients with Klinefelter syndrome: a critical review. Fertil
Steril. 2004 Oct;82(4):775-9. Review. PubMed [citation] PMID: 15482743

848. Siffroi JP; Commission de Génétique de la Fédération Française des CECOS..
[Gamete donor karyotyping: between real usefulness and safety rules]. Gynecol
Obstet Fertil. 2004 Sep;32(9):803-12. Review. French. PubMed [citation] PMID:
15380766

849. De Morentin HM, Dodiuk-Gad RP, Brenner S. Klinefelter's syndrome presenting with
leg ulcers. Skinmed. 2004 Sep-Oct;3(5):274-8. Review. PubMed [citation] PMID:
15365265

850. Tyler C, Edman JC. Down syndrome, Turner syndrome, and Klinefelter syndrome:
primary care throughout the life span. Prim Care. 2004 Sep;31(3):627-48, x-xi.
Review. PubMed [citation] PMID: 15331252

851. Lette J, Cerino M, Eybalin MC, Levasseur A. Prevalence and clinical significance
of solitary pulmonary sub-segmental microembolism. Nucl Med Rev Cent East Eur.
2004;7(1):39-42. PubMed [citation] PMID: 15318309

852. Hyun G, Kolon TF. A practical approach to intersex in the newborn period. Urol
Clin North Am. 2004 Aug;31(3):435-43, viii. Review. PubMed [citation] PMID:
15313053

853. Lemke AJ, Niehues SM, Hosten N, Amthauer H, Boehmig M, Stroszczynski C, Rohlfing
T, Rosewicz S, Felix R. Retrospective digital image fusion of multidetector CT
and 18F-FDG PET: clinical value in pancreatic lesions--a prospective study with
104 patients. J Nucl Med. 2004 Aug;45(8):1279-86. PubMed [citation] PMID:
15299049

854. Fukunaga M. Immunohistochemical characterization of p57Kip2 expression in
tetraploid hydropic placentas. Arch Pathol Lab Med. 2004 Aug;128(8):897-900.
Review. PubMed [citation] PMID: 15270611

855. Lanfranco F, Kamischke A, Zitzmann M, Nieschlag E. Klinefelter's syndrome.
Lancet. 2004 Jul 17-23;364(9430):273-83. Review. PubMed [citation] PMID: 15262106

856. Weber LT, Armstrong VW, Shipkova M, Feneberg R, Wiesel M, Mehls O, Zimmerhackl
LB, Oellerich M, Tönshoff B; Members of the German Study Group on Pediatric Renal
Transplantion.. Cyclosporin A absorption profiles in pediatric renal transplant
recipients predict the risk of acute rejection. Ther Drug Monit. 2004
Aug;26(4):415-24. PubMed [citation] PMID: 15257072

857. Potter JM, McWhinney BC, Sampson L, Hickman PE. Area-under-the-curve monitoring
of prednisolone for dose optimization in a stable renal transplant population.
Ther Drug Monit. 2004 Aug;26(4):408-14. PubMed [citation] PMID: 15257071

858. Suga K, Kawakami Y, Zaki M, Yamashita T, Shimizu K, Matsunaga N. Clinical utility
of co-registered respiratory-gated(99m)Tc-Technegas/MAA SPECT-CT images in the
assessment of regional lung functional impairment in patients with lung cancer.

Eur J Nucl Med Mol Imaging. 2004 Sep;31(9):1280-90. Epub 2004 Jun 10. PubMed
[citation] PMID: 15197501

859. Diamond M, Watson LA. Androgen insensitivity syndrome and Klinefelter's syndrome:
sex and gender considerations. Child Adolesc Psychiatr Clin N Am. 2004
Jul;13(3):623-40, viii. Review. PubMed [citation] PMID: 15183377

860. Suga K, Kawakami Y, Zaki M, Yamashita T, Matsumoto T, Matsunaga N. Pulmonary
perfusion assessment with respiratory gated 99mTc macroaggregated albumin SPECT:
preliminary results. Nucl Med Commun. 2004 Feb;25(2):183-93. PubMed [citation]
PMID: 15154710

861. Spénard J, Aumais C, Massicotte J, Tremblay C, Lefebvre M. Influence of
omeprazole on bioavailability of bismuth following administration of a triple
capsule of bismuth biskalcitrate, metronidazole, and tetracycline. J Clin
Pharmacol. 2004 Jun;44(6):640-5. PubMed [citation] PMID: 15145972

862. Seitz B, Langenbucher A, Nguyen NX, Kus MM, Küchle M, Naumann GO. [Results of the
first 1,000 consecutive elective nonmechanical keratoplasties using the excimer
laser. A prospective study over more than 12 years]. Ophthalmologe. 2004
May;101(5):478-88. German. PubMed [citation] PMID: 15138797

863. Gribajcević M, Vanis N, Mesihović R. [Clinical effectiveness of omeprazole,
azithromycin and amoxicillin in ulcer healing and eradication of Helicobacter
pylori infection]. Med Arh. 2003;57(1 Suppl 2):107-10. Bosnian. PubMed [citation]
PMID: 15137249

864. Suga K, Yasuhiko K, Zaki M, Yamashita T, Seto A, Matsumoto T, Matsunaga N.
Assessment of regional lung functional impairment with co-registered
respiratory-gated ventilation/perfusion SPET-CT images: initial experiences. Eur
J Nucl Med Mol Imaging. 2004 Feb;31(2):240-9. PubMed [citation] PMID: 15129707

865. Blanckenberg DH, Wood R, Horban A, Beniowski M, Boron-Kaczmarska

A, Trocha H,
Halota W, Schmidt RE, Fatkenheuer G, Jessen H, Lange JM; CHARM Study Group..
Evaluation of nevirapine and/or hydroxyurea with nucleoside reverse transcriptase
inhibitors in treatment-naive HIV-1-infected subjects. AIDS. 2004 Mar 5;18(4):631-40. PubMed [citation] PMID: 15090768

866. Iliopoulos D, Poultsides G, Peristeri V, Kouri G, Andreou A, Voyiatzis N. Double
trisomy (48,XXY,+21) in monozygotic twins: case report and review of the
literature. Ann Genet. 2004 Jan-Mar;47(1):95-8. Review. PubMed [citation] PMID:
15050879

867. Jun SY, Cho KJ, Kim CS, Ayala AG, Ro JY. Triple synchronous neoplasms in one
kidney: report of a case and review of the literature. Ann Diagn Pathol. 2003
Dec;7(6):374-80. Review. PubMed [citation] PMID: 15018122

868. Kikuchi I, Takeuchi H, Kinoshita K. [XY type gonadal dysgenesis, trisomy X and
variants]. Nihon Rinsho. 2004 Feb;62(2):309-12. Review. Japanese. PubMed
[citation] PMID: 14968537

869. Itoh N, Tsukamoto T. [XX 'pure' gonadal dysgenesis and XYY syndrome]. Nihon
Rinsho. 2004 Feb;62(2):305-8. Review. Japanese. PubMed [citation] PMID: 14968536

870. Ogawa Y, Yoshida H. [Klinefelter syndrome]. Nihon Rinsho. 2004 Feb;62(2):327-32.
Review. Japanese. PubMed [citation] PMID: 14968540

871. Nowaczyk MJ, Zeesman S, Kam A, Taylor SA, Carter RF, Whelan DT. Boy with
47,XXY,del(15)(q11.2q13) karyotype and Prader-Willi syndrome: a new case and
review of the literature. Am J Med Genet A. 2004 Feb 15;125A(1):73-6. Review.
PubMed [citation] PMID: 14755470

872. Zhang Y, Wang XK, Yang CM, Liu GY. [Use of unfractionated heparin and a
low-molecular-weight heparin following thrombolytic therapy for acute ST-segment
elevation myocardial infarction]. Di Yi Jun Yi Da Xue Xue Bao. 2004

Jan;24(1):81-4. Chinese. PubMed [citation] PMID: 14724106

873. Hoffman B, Bradshaw KD. Delayed puberty and amenorrhea. Semin Reprod Med. 2003
Nov;21(4):353-62. Review. PubMed [citation] PMID: 14724768

874. Sankatsing SU, Weverling GJ, Peeters M, van't Klooster G, Gruzdev B, Rakhmanova
A, Danner SA, Jurriaans S, Prins JM, Lange JM. TMC125 exerts similar initial
antiviral potency as a five-drug, triple class antiretroviral regimen. AIDS. 2003
Dec 5;17(18):2623-7. PubMed [citation] PMID: 14685056

875. Kaido T, Sasaoka Y, Hashimoto H, Taira K. De novo germinoma in the brain in
association with Klinefelter's syndrome: case report and review of the
literature. Surg Neurol. 2003 Dec;60(6):553-8; discussion 559. Review. PubMed
[citation] PMID: 14670679

876. Bottio T, Rizzoli G, Vida V, Casarotto D, Gerosa G. Double crisscross sternal
wiring and chest wound infections: a prospective randomized study. J Thorac
Cardiovasc Surg. 2003 Nov;126(5):1352-6. PubMed [citation] PMID: 14666006

877. Alapont Alacreu JM, Domínguez Hinarejos C, Serrano Durbá A, Estornell F, Martínez
Verduch M, Vera Sempere F, Moreno F, García Ibarra F. [Bilateral testicular
epidermoid cyst in a pediatric patient with Klinefelter syndrome]. Actas Urol
Esp. 2003 Oct;27(9):742-4. Review. Spanish. PubMed [citation] PMID: 14626688

878. Satgé D, Moore SW, Stiller CA, Niggli FK, Pritchard-Jones K, Bown N, Bénard J,
Plantaz D. Abnormal constitutional karyotypes in patients with neuroblastoma: a
report of four new cases and review of 47 others in the literature. Cancer Genet
Cytogenet. 2003 Dec;147(2):89-98. Review. PubMed [citation] PMID: 14623457

879. Vogt T, Hafner C, Bross K, Bataille F, Jauch KW, Berand A, Landthaler M,
Andreesen R, Reichle A. Antiangiogenetic therapy with pioglitazone, rofecoxib,

and metronomic trofosfamide in patients with advanced malignant
vascular tumors.
Cancer. 2003 Nov 15;98(10):2251-6. PubMed [citation] PMID: 14601096

880. Kearns GL, Jungbluth GL, Abdel-Rahman SM, Hopkins NK, Welshman IR, Grzebyk RP,
Bruss JB, Van Den Anker JN; Pediatric Pharmacology Research Unit Network.. Impact
of ontogeny on linezolid disposition in neonates and infants. Clin Pharmacol
Ther. 2003 Nov;74(5):413-22. PubMed [citation] PMID: 14586382

881. Gonzalo IT, Itti E, Mlikotic A, Pham le H, Cesar RB, Meignan M, Mishkin FS.
[18F]fluorodeoxyglucose triple-head coincidence imaging as an adjunct to 131I
scanning for follow-up of papillary thyroid carcinoma. Endocr Pract. 2003
Jul-Aug;9(4):273-9. PubMed [citation] PMID: 14561570

882. Kadakia AR, Haddad SL. Hindfoot arthrodesis for the adult acquired flat foot.
Foot Ankle Clin. 2003 Sep;8(3):569-94, x. Review. PubMed [citation] PMID:
14560906

883. Stepan JJ, Burckhardt P, Hána V. The effects of three-month intravenous
ibandronate on bone mineral density and bone remodeling in Klinefelter's
syndrome: the influence of vitamin D deficiency and hormonal status. Bone. 2003
Oct;33(4):589-96. PubMed [citation] PMID: 14555263

884. Miehlke S, Schneider-Brachert W, Bästlein E, Ebert S, Kirsch C, Haferland C,
Buchner M, Neumeyer M, Vieth M, Stolte M, Lehn N, Bayerdörffer E.
Esomeprazole-based one-week triple therapy with clarithromycin and metronidazole
is effective in eradicating Helicobacter pylori in the absence of antimicrobial
resistance. Aliment Pharmacol Ther. 2003 Oct 15;18(8):799-804. PubMed [citation]
PMID: 14535873

885. Schummer W, Herrmann S, Schummer C, Funke F, Steenbeck J, Fuchs J, Uhlig T,
Reinhart K. Intra-atrial ECG is not a reliable method for positioning left
internal jugular vein catheters. Br J Anaesth. 2003 Oct;91(4):481-6.

PubMed
[citation] PMID: 14504146

886. Gerstoft J, Kirk O, Obel N, Pedersen C, Mathiesen L, Nielsen H, Katzenstein TL,
Lundgren JD. Low efficacy and high frequency of adverse events in a randomized
trial of the triple nucleoside regimen abacavir, stavudine and didanosine. AIDS.
2003 Sep 26;17(14):2045-52. PubMed [citation] PMID: 14502007

887. Pagotto U, Gambineri A, Pelusi C, Genghini S, Cacciari M, Otto B, Castañeda T,
Tschöp M, Pasquali R. Testosterone replacement therapy restores normal ghrelin in
hypogonadal men. J Clin Endocrinol Metab. 2003 Sep;88(9):4139-43. PubMed
[citation] PMID: 12970277

888. O'Brien S, Giles F, Talpaz M, Cortes J, Rios MB, Shan J, Thomas D, Andreeff M,
Kornblau S, Faderl S, Garcia-Manero G, White K, Mallard S, Freireich E,
Kantarjian HM. Results of triple therapy with interferon-alpha, cytarabine, and
homoharringtonine, and the impact of adding imatinib to the treatment sequence in
patients with Philadelphia chromosome-positive chronic myelogenous leukemia in
early chronic phase. Cancer. 2003 Sep 1;98(5):888-93. PubMed [citation] PMID:
12942553

889. Charles YP, Axt M, Döderlein L. [Surgical treatment of cavovarus foot deformity
considering dynamic pedobarography]. Z Orthop Ihre Grenzgeb. 2003
Jul-Aug;141(4):433-9. German. PubMed [citation] PMID: 12929001

890. Thomas NS, Hassold TJ. Aberrant recombination and the origin of Klinefelter
syndrome. Hum Reprod Update. 2003 Jul-Aug;9(4):309-17. Review. PubMed [citation]
PMID: 12926525

891. Staessen C, Tournaye H, Van Assche E, Michiels A, Van Landuyt L, Devroey P,
Liebaers I, Van Steirteghem A. PGD in 47,XXY Klinefelter's syndrome patients. Hum
Reprod Update. 2003 Jul-Aug;9(4):319-30. Review. PubMed [citation]
PMID: 12926526

892. Guzmán Martínez-Valls PL, Hita Villaplana G, Fernández Aparicio T, Miñana López
B, Martínez Díaz F, Sánchez Gascon F. [Significance and management of testicular
microlithiasis]. Arch Esp Urol. 2003 Jun;56(5):472-7. Review. Spanish. PubMed
[citation] PMID: 12918303

893. Brugger H, Sumann G, Meister R, Adler-Kastner L, Mair P, Gunga HC, Schobersberger
W, Falk M. Hypoxia and hypercapnia during respiration into an artificial air
pocket in snow: implications for avalanche survival. Resuscitation. 2003
Jul;58(1):81-8. PubMed [citation] PMID: 12867313

894. Temple CM, Sanfilippo PM. Executive skills in Klinefelter's syndrome.
Neuropsychologia. 2003;41(11):1547-59. PubMed [citation] PMID: 12849773

895. Rives N, Siméon N, Milazzo JP, Barthélémy C, Macé B. Meiotic segregation of sex
chromosomes in mosaic and non-mosaic XYY males: case reports and review of the
literature. Int J Androl. 2003 Aug;26(4):242-9. Review. PubMed [citation] PMID:
12846800

896. Hameed A, Hanna-Moussa S, David S. Use of multiple stents to seal off an
epicardial pseudoaneurysm. J Invasive Cardiol. 2003 Jul;15(7):405-7. Review.
PubMed [citation] PMID: 12840240

897. Shah K, Sivapalan G, Gibbons N, Tempest H, Griffin DK. The genetic basis of
infertility. Reproduction. 2003 Jul;126(1):13-25. Review. PubMed [citation] PMID:
12814343

898. Roghani HS, Massarrat S, Shirekhoda M, Butorab Z. Effect of different doses of
furazolidone with amoxicillin and omeprazole on eradication of Helicobacter
pylori. J Gastroenterol Hepatol. 2003 Jul;18(7):778-82. PubMed [citation] PMID:
12795748

899. Toretsky JA, Jenson J, Sun CC, Eskenazi AE, Campbell A, Hunger SP, Caires A,
Frantz C, Hill JL, Stamberg J. Translocation (11;15;19): a highly specific
chromosome rearrangement associated with poorly differentiated thymic carcinoma
in young patients. Am J Clin Oncol. 2003 Jun;26(3):300-6. Review. PubMed
[citation] PMID: 12796605

900. van Leeuwen R, Katlama C, Murphy RL, Squires K, Gatell J, Horban A, Clotet B,
Staszewski S, van Eeden A, Clumeck N, Moroni M, Pavia AT, Schmidt RE,
Gonzalez-Lahoz J, Montaner J, Antunes F, Gulick R, Bánhegyi D, van der Valk M,
Reiss P, van Weert L, van Leth F, et al. A randomized trial to study first-line
combination therapy with or without a protease inhibitor in HIV-1-infected
patients. AIDS. 2003 May 2;17(7):987-99. PubMed [citation] PMID: 12700448

901. Hawkey CJ, Atherton JC, Treichel HC, Thjodleifsson B, Ravic M. Safety and
efficacy of 7-day rabeprazole- and omeprazole-based triple therapy regimens for
the eradication of Helicobacter pylori in patients with documented peptic ulcer
disease. Aliment Pharmacol Ther. 2003 Apr;17(8):1065-74. PubMed [citation] PMID:
12694089

902. Beresford L, Fernandez CV, Cummings E, Sanderson S, Ming-Yu W, Giacomantonio M.
Mediastinal polyembryoma associated with Klinefelter syndrome. J Pediatr Hematol
Oncol. 2003 Apr;25(4):321-3. Review. PubMed [citation] PMID: 12679648

903. Hadziselimovic F, Huff D. Gonadal differentiation--normal and abnormal testicular
development. Adv Exp Med Biol. 2002;511:15-21; discussion 21-3. Review. No
abstract available. PubMed [citation] PMID: 12575753

904. Tachdjian G, Frydman N, Morichon-Delvallez N, Dû AL, Fanchin R, Vekemans M,
Frydman R. Reproductive genetic counselling in non-mosaic 47,XXY patients:
implications for preimplantation or prenatal diagnosis: Case report and review.

Hum Reprod. 2003 Feb;18(2):271-5. Review. PubMed [citation] PMID: 12571161

905. Kneeshaw PJ, Turnbull LW, Drew PJ. Current applications and future direction of
MR mammography. Br J Cancer. 2003 Jan 13;88(1):4-10. Review. PubMed [citation]
PMID: 12556951, PMCID: PMC2376788

906. Keung YK, Buss D, Chauvenet A, Pettenati M. Hematologic malignancies and
Klinefelter syndrome. a chance association? Cancer Genet Cytogenet. 2002
Nov;139(1):9-13. Review. PubMed [citation] PMID: 12547150

907. Matl I, Bachleda P, Lao M, Michalský R, Navrátil P, Treska V, Prestele H,
Matthisson M, Korn A. Safety and efficacy of an alternative basiliximab
(Simulect) regimen after renal transplantation: administration of a single 40-mg
dose on the first postoperative day in patients receiving triple therapy with
azathioprine. Transpl Int. 2003 Jan;16(1):45-52. Epub 2002 Nov 22. PubMed
[citation] PMID: 12545341

908. Sax L. How common is intersex? a response to Anne Fausto-Sterling. J Sex Res.
2002 Aug;39(3):174-8. Review. PubMed [citation] PMID: 12476264

909. Corti R, Binggeli C, Sudano I, Spieker L, Hänseler E, Ruschitzka F, Chaplin WF,
Lüscher TF, Noll G. Coffee acutely increases sympathetic nerve activity and blood
pressure independently of caffeine content: role of habitual versus nonhabitual
drinking. Circulation. 2002 Dec 3;106(23):2935-40. PubMed [citation] PMID:
12460875

910. Hara Y, Harada Y, Fujita Y, Taoda T, Nezu Y, Yamaguchi S, Orima H, Tagawa M.
Changes of hip joint congruity after triple pelvic osteotomy in the dog with hip
dysplasia. J Vet Med Sci. 2002 Oct;64(10):933-6. PubMed [citation]
PMID: 12419871

911. Ota K, Suehiro T, Ikeda Y, Arii K, Kumon Y, Hashimoto K. Diabetes mellitus

associated with Klinefelter's syndrome: a case report and review in Japan. Intern
Med. 2002 Oct;41(10):842-7. Review. PubMed [citation] PMID: 12413007

912. Gokel GW, Barbour LJ, Ferdani R, Hu J. Lariat ether receptor systems show
experimental evidence for alkali metal cation-pi interactions. Acc Chem Res. 2002
Oct;35(10):878-86. Review. PubMed [citation] PMID: 12379140

913. Giordano SH, Buzdar AU, Hortobagyi GN. Breast cancer in men. Ann Intern Med. 2002
Oct 15;137(8):678-87. Review. PubMed [citation] PMID: 12379069

914. McDonald BC. Recent developments in the application of the nonverbal learning
disabilities model. Curr Psychiatry Rep. 2002 Oct;4(5):323-30. Review. PubMed
[citation] PMID: 12230960

915. Light J, Salomon DR, Diethelm AG, Alexander JW, Hunsicker L, Thistlethwaite R,
Reinsmoen N, Stablein DM. Bone marrow transfusions in cadaver renal allografts:
pilot trials with concurrent controls. Clin Transplant. 2002 Oct;16(5):317-24.
Review. PubMed [citation] PMID: 12225426

916. Zamora L, Espinet B, Salido M, Solé F, Ligorria C, Florensa L. Report of
46,XX/46,XY/47,XXY/48,XXYY mosaicism in an adult phenotypic male. Am J Med Genet.
2002 Aug 1;111(2):215-7. Review. No abstract available. PubMed [citation] PMID:
12210355

917. Simoons M, Krzemiñska-Pakula M, Alonso A, Goodman S, Kali A, Loos U, Gosset F,
Louer V, Bigonzi F; AMI-SK Investigator.. Improved reperfusion and clinical
outcome with enoxaparin as an adjunct to streptokinase thrombolysis in acute
myocardial infarction. The AMI-SK study. Eur Heart J. 2002 Aug;23(16):1282-90.
PubMed [citation] PMID: 12175665

918. Meguerditchian AN, Falardeau M, Martin G. Male breast carcinoma. Can J Surg. 2002
Aug;45(4):296-302. Review. PubMed [citation] PMID: 12174988, PMCID: PMC3684685

919. Tsujimura A, Matsumiya K, Okuyama A. [Testicular dysfunction]. Nihon Rinsho. 2002
Jun;60 Suppl 6:344-7. Review. Japanese. No abstract available. PubMed [citation]
PMID: 12166173

920. Delgado H, Lehmann T, Bobbioni-Harsch E, Ybarra J, Golay A. Acarbose improves
indirectly both insulin resistance and secretion in obese type 2 diabetic
patients. Diabetes Metab. 2002 Jun;28(3):195-200. PubMed [citation] PMID:
12149599

921. Berisio R, Vitagliano L, Mazzarella L, Zagari A. Recent progress on collagen
triple helix structure, stability and assembly. Protein Pept Lett. 2002
Apr;9(2):107-16. Review. PubMed [citation] PMID: 12141907

922. Paul SB, Gulati MS. Spectrum of hepatocellular carcinoma on triple phase helical
CT: a pictorial essay. Clin Imaging. 2002 Jul-Aug;26(4):270-9. Review. PubMed
[citation] PMID: 12140159

923. Lim JH, Choi D, Kim SH, Lee SJ, Lee WJ, Lim HK, Kim S. Detection of
hepatocellular carcinoma: value of adding delayed phase imaging to dual-phase
helical CT. AJR Am J Roentgenol. 2002 Jul;179(1):67-73. PubMed [citation] PMID:
12076907

924. Murakami C, Nunomura J, Baba M. [MELAS syndrome associated with Klinefelter
syndrome]. Nihon Rinsho. 2002 Apr;60 Suppl 4:625-8. Review. Japanese. No abstract
available. PubMed [citation] PMID: 12013958

925. Manning MA, Hoyme HE. Diagnosis and management of the adolescent boy with
Klinefelter syndrome. Adolesc Med. 2002 Jun;13(2):367-74, viii. Review. PubMed
[citation] PMID: 11986043

926. Kumar S, Menke DM, Dewald GW, Colon-Otero G. Agnogenic myeloid metaplasia
associated with Klinefelter syndrome: a case report. Ann Hematol. 2002

Apr;81(4):215-8. Epub 2002 Mar 7. Review. PubMed [citation] PMID: 11976824

927. Kopp AF, Heuschmid M, Claussen CD. Multidetector helical CT of the liver for
tumor detection and characterization. Eur Radiol. 2002 Apr;12(4):745-52. Epub
2001 Nov 29. Review. PubMed [citation] PMID: 11960221

928. Jenkins CL, Raines RT. Insights on the conformational stability of collagen. Nat
Prod Rep. 2002 Feb;19(1):49-59. Review. PubMed [citation] PMID: 11902439

929. Wan TS, Yip SF, Yeung YM, Chan LC, Ma SK. Fatal diffuse alveolar damage
complicating acute myeloid leukemia with abnormal eosinophils and trisomy X. Ann
Hematol. 2002 Mar;81(3):167-9. Epub 2002 Feb 16. Review. PubMed [citation] PMID:
11904745

930. Noda Y, Oka D, Tei N, Takada S, Koide T, Miyajima S, Okada N. [48XXYY
Klinefelter's syndrome with recurrent foot ulcers: a case report]. Hinyokika
Kiyo. 2002 Jan;48(1):17-9. Review. Japanese. PubMed [citation] PMID: 11868379

931. Yamaguchi H, Inokuchi K, Yokomizo E, Miyata J, Watanabe A, Inami M, Tajika K, Dan
K. Philadelphia chromosome-positive acute myeloid leukemia with tetraploidy. Int
J Hematol. 2002 Jan;75(1):63-6. Review. PubMed [citation] PMID: 11843293

932. Baba K, Iwamoto T. [Klinefelter syndrome]. Nihon Rinsho. 2002 Jan;60 Suppl
1:614-7. Review. Japanese. No abstract available. PubMed [citation] PMID:
11838174

933. Sheu BS, Yang HB, Wang YL, Chuang CH, Huang AH, Wu JJ. Pretreatment gastric
histology is helpful to predict the symptomatic response after H. pylori
eradication in patients with nonulcer dyspepsia. Dig Dis Sci. 2001
Dec;46(12):2700-7. PubMed [citation] PMID: 11768263

934. Visootsak J, Aylstock M, Graham JM Jr. Klinefelter syndrome and

its variants: an
update and review for the primary pediatrician. Clin Pediatr (Phila). 2001
Dec;40(12):639-51. Review. PubMed [citation] PMID: 11771918

935. Triolo A, Altamura M, Cardinali F, Sisto A, Maggi CA. Mass spectrometry and
combinatorial chemistry: a short outline. J Mass Spectrom. 2001
Dec;36(12):1249-59. Review. PubMed [citation] PMID: 11754116

936. Schanen C. Rethinking the fate of males with mutations in the gene that causes
Rett syndrome. Brain Dev. 2001 Dec;23 Suppl 1:S144-6. Review. PubMed [citation]
PMID: 11738861

937. Seitz B, Langenbucher A, Diamantis A, Cursiefen C, Küchle M, Naumann GO.
[Immunological graft reactions after penetrating keratoplasty - A prospective
randomized trial comparing corneal excimer laser and motor trephination]. Klin
Monbl Augenheilkd. 2001 Nov;218(11):710-9. German. PubMed [citation] PMID:
11731898

938. Simpson JL, Lamb DJ. Genetic effects of intracytoplasmic sperm injection. Semin
Reprod Med. 2001 Sep;19(3):239-49. Review. PubMed [citation] PMID: 11679905

939. Ji H, McTavish JD, Mortele KJ, Wiesner W, Ros PR. Hepatic imaging with
multidetector CT. Radiographics. 2001 Oct;21 Spec No:S71-80. Review. PubMed
[citation] PMID: 11598249

940. Cirigliano V, Ejarque M, Cañadas MP, Lloveras E, Plaja A, Perez MM, Fuster C,
Egozcue J. Clinical application of multiplex quantitative fluorescent polymerase
chain reaction (QF-PCR) for the rapid prenatal detection of common chromosome
aneuploidies. Mol Hum Reprod. 2001 Oct;7(10):1001-6. PubMed [citation] PMID:
11574670

941. Hirohara D, Nomura K, Okamoto T, Ujihara M, Takano K. Performance of the basal
aldosterone to renin ratio and of the renin stimulation test by

furosemide and
upright posture in screening for aldosterone-producing adenoma in low renin
hypertensives. J Clin Endocrinol Metab. 2001 Sep;86(9):4292-8. PubMed [citation]
PMID: 11549664

942. Bartlett JA, DeMasi R, Quinn J, Moxham C, Rousseau F. Overview of the
effectiveness of triple combination therapy in antiretroviral-naive HIV-1
infected adults. AIDS. 2001 Jul 27;15(11):1369-77. PubMed [citation] PMID:
11504958

943. Samango-Sprouse C. Mental development in polysomy X Klinefelter syndrome (47,XXY;
48,XXXY): effects of incomplete X inactivation. Semin Reprod Med. 2001
Jun;19(2):193-202. Review. PubMed [citation] PMID: 11480917

944. Satgé D, Sasco AJ, Plantaz D, Bénard J, Vekemans MJ. Abnormal number of X
chromosomes and neuroblastic tumors. J Pediatr Hematol Oncol. 2001
Jun-Jul;23(5):331-2. Review. No abstract available. PubMed [citation] PMID:
11464996

945. Ruiz L, Negredo E, Domingo P, Paredes R, Francia E, Balagué M, Gel S, Bonjoch A,
Fumaz CR, Johnston S, Romeu J, Lange J, Clotet B; Spanish Lipodystrophy Group..
Antiretroviral treatment simplification with nevirapine in protease
inhibitor-experienced patients with hiv-associated lipodystrophy: 1-year
prospective follow-up of a multicenter, randomized, controlled study. J Acquir
Immune Defic Syndr. 2001 Jul 1;27(3):229-36. PubMed [citation] PMID:
11464141

946. Ohashi H. [Chromosome XYY, XXY]. Ryoikibetsu Shokogun Shirizu. 2001;(33):450-1.
Review. Japanese. No abstract available. PubMed [citation] PMID:
11462511

947. Shi Q, Martin RH. Aneuploidy in human spermatozoa: FISH analysis in men with
constitutional chromosomal abnormalities, and in infertile men. Reproduction.
2001 May;121(5):655-66. Review. PubMed [citation] PMID: 11427153

948. Montalban C, Santon A, Boixeda D, Redondo C, Alvarez I, Calleja JL, de Argila CM,
Bellas C. Treatment of low grade gastric mucosa-associated lymphoid tissue
lymphoma in stage I with Helicobacter pylori eradication. Long-term results after
sequential histologic and molecular follow-up. Haematologica. 2001 Jun;86(6):609-17. PubMed [citation] PMID: 11418369

949. Dubé MP, Edmondson-Melançon H, Qian D, Aqeel R, Johnson D, Buchanan TA.
Prospective evaluation of the effect of initiating indinavir-based therapy on
insulin sensitivity and B-cell function in HIV-infected patients. J Acquir Immune
Defic Syndr. 2001 Jun 1;27(2):130-4. PubMed [citation] PMID: 11404534

950. Kirk O, Mocroft A, Pradier C, Bruun JN, Hemmer R, Clotet B, Miller V, Viard JP,
Phillips AN, Lundgren JD; EuroSIDA Study Group.. Clinical outcome among
HIV-infected patients starting saquinavir hard gel compared to ritonavir or
indinavir. AIDS. 2001 May 25;15(8):999-1008. PubMed [citation] PMID: 11399982

951. Merviel P, Aractingi S, Uzan S. [Detection of fetal cells in maternal blood: myth
or reality?]. Gynecol Obstet Fertil. 2001 May;29(5):371-6. Review. French. PubMed
[citation] PMID: 11406933

952. De Rosa M, Paesano L, Nuzzo V, Zarrilli S, Del Puente A, Oriente P, Lupoli G.
Bone mineral density and bone markers in hypogonadotropic and hypergonadotropic
hypogonadal men after prolonged testosterone treatment. J Endocrinol Invest. 2001
Apr;24(4):246-52. PubMed [citation] PMID: 11383911

953. Odent S, Taque S, Lucas J, Le Mee F, Le Marec B. Prader-Willi syndrome and
polygonosomal abnormalities in males:about a Prader-Willi/47,XYY patient. Ann
Genet. 2001 Jan-Mar;44(1):1-3. Review. PubMed [citation] PMID: 11334609

954. Crémieux AC, Katlama C, Gillotin C, Demarles D, Yuen GJ, Raffi F; AZl10002 Study
Group.. A comparison of the steady-state pharmacokinetics and safety

of abacavir,
lamivudine, and zidovudine taken as a triple combination tablet and as abacavir
plus a lamivudine-zidovudine double combination tablet by HIV-1-infected adults.
Pharmacotherapy. 2001 Apr;21(4):424-30. PubMed [citation] PMID: 11310515

955. Buevich A, Baum J. Nuclear magnetic resonance characterization of peptide models
of collagen-folding diseases. Philos Trans R Soc Lond B Biol Sci. 2001 Feb
28;356(1406):159-68. Review. PubMed [citation] PMID: 11260796, PMCID: PMC1088421

956. Staszewski S, Keiser P, Montaner J, Raffi F, Gathe J, Brotas V, Hicks C, Hammer
SM, Cooper D, Johnson M, Tortell S, Cutrell A, Thorborn D, Isaacs R, Hetherington
S, Steel H, Spreen W; CNAAB3005 International Study Team..
Abacavir-lamivudine-zidovudine vs indinavir-lamivudine-zidovudine in
antiretroviral-naive HIV-infected adults: A randomized equivalence trial. JAMA.
2001 Mar 7;285(9):1155-63. Erratum in: JAMA 2001 Jun 13;285(22):2858. PubMed
[citation] PMID: 11231744

957. Breuil V, Euller-Ziegler L. Gonadal dysgenesis and bone metabolism. Joint Bone
Spine. 2001 Feb;68(1):26-33. Review. PubMed [citation] PMID: 11235777

958. Gu YQ, Ge ZY, Zhang GY, Bremner WJ. Quantitative and qualitative changes in serum
luteinizing hormone after injectable testosterone undecanoate treatment in
hypogonadal men. Asian J Androl. 2000 Mar;2(1):65-71. PubMed [citation] PMID:
11228940

959. Hainsworth JD, Greco FA. Germ cell neoplasms and other malignancies of the
mediastinum. Cancer Treat Res. 2001;105:303-25. Review. No abstract available.
PubMed [citation] PMID: 11224992

960. Kamps WA, Veerman AJ, van Wering ER, van Weerden JF, Slater R, van der Does-van
den Berg A. Long-term follow-up of Dutch Childhood Leukemia Study Group (DCLSG)
protocols for children with acute lymphoblastic leukemia, 1984-1991.

Leukemia.
2000 Dec;14(12):2240-6. PubMed [citation] PMID: 11187915

961. O'Brien G. Behavioural phenotypes. J R Soc Med. 2000
Dec;93(12):618-20. Review.
No abstract available. PubMed [citation] PMID: 11193058, PMCID:
PMC1298165

962. McKinlay J, Wildgoose A, Wood W, Gould IM, Anderton A. The effect
of system
design on bacterial contamination of enteral tube feeds. J Hosp
Infect. 2001
Feb;47(2):138-42. PubMed [citation] PMID: 11170778

963. Niemi G, Breivik H. Epidural fentanyl markedly improves thoracic
epidural
analgesia in a low-dose infusion of bupivacaine, adrenaline and
fentanyl. A
randomized, double-blind crossover study with and without fentanyl.
Acta
Anaesthesiol Scand. 2001 Feb;45(2):221-32. PubMed [citation] PMID:
11167169

964. Kleiman SE, Lagziel A, Yogev L, Botchan A, Paz G, Yavetz H.
Expression of CDY1
may identify complete spermatogenesis. Fertil Steril. 2001
Jan;75(1):166-73.
PubMed [citation] PMID: 11163833

965. Scheidbach H, Dworak O, Schmucker B, Hohenberger W. Lobular
carcinoma of the
breast in an 85-year-old man. Eur J Surg Oncol. 2000 Apr;26(3):319-21.
Review.
PubMed [citation] PMID: 10753542

966. Yasui T, Kishi H, Komiyama M, Iwai Y, Yamanaka K, Nishikawa M,
Nakajima H,
Morikawa T. [The limitations of three-dimensional CT angiography (3D-
CTA) in the
diagnosis of cerebral aneurysms]. No Shinkei Geka. 2000
Nov;28(11):975-81.
Japanese. PubMed [citation] PMID: 11127593

967. Soyka LA, Fairfield WP, Klibanski A. Clinical review 117:
Hormonal determinants
and disorders of peak bone mass in children. J Clin Endocrinol Metab.
2000
Nov;85(11):3951-63. Review. No abstract available. PubMed [citation]
PMID:
11095413

968. Lahita RG. Sex hormones and systemic lupus erythematosus. Rheum Dis Clin North
Am. 2000 Nov;26(4):951-68. Review. PubMed [citation] PMID: 11084953

969. García F, Alonso MM, Romeu J, Knobel H, Arrizabalaga J, Ferrer E, Dalmau D, Ruiz
I, Vidal F, Frances A, Segura F, Gomez-Sirvent JL, Cruceta A, Clotet B, Pumarola
T, Gallart T, O'Brien WA, Miró JM, Gatell JM. Comparison of immunologic
restoration and virologic response in plasma, tonsillar tissue, and cerebrospinal
fluid in HIV-1-infected patients treated with double versus triple antiretroviral
therapy in very early stages: The Spanish EARTH-2 Study. Early Anti-Retroviral
Therapy Study. J Acquir Immune Defic Syndr. 2000 Sep 1;25(1):26-35. PubMed
[citation] PMID: 11064501

970. Okuyama T, Oho Y, Kosuga M. [Sex chromosome abnormality]. Ryoikibetsu Shokogun
Shirizu. 2000;(30 Pt 5):353-4. Review. Japanese. No abstract available. PubMed
[citation] PMID: 11057258

971. Okuyama T, Kosuga M. [Klinefelter syndrome]. Ryoikibetsu Shokogun Shirizu.
2000;(30 Pt 5):355-7. Review. Japanese. No abstract available. PubMed [citation]
PMID: 11057259

972. Brunori M. Structural dynamics of myoglobin. Biophys Chem. 2000 Aug
30;86(2-3):221-30. Review. PubMed [citation] PMID: 11026686

973. Shin E. [Male breast cancer]. Nihon Rinsho. 2000 Apr;58 Suppl:571-4. Review.
Japanese. No abstract available. PubMed [citation] PMID: 11026052

974. Plana M, García F, Gallart T, Tortajada C, Soriano A, Palou E, Maleno MJ, Barceló
JJ, Vidal C, Cruceta A, Miró JM, Gatell JM. Immunological benefits of
antiretroviral therapy in very early stages of asymptomatic chronic HIV-1
infection. AIDS. 2000 Sep 8;14(13):1921-33. PubMed [citation] PMID: 10997396

975. Durak H, Söylev M, Durak I, Değirmenci B, Capa Kaya G, Uysal B.

Tc-99m polyclonal
human immunoglobulin G imaging in Graves' ophthalmopathy. Clin Nucl Med. 2000
Sep;25(9):704-7. PubMed [citation] PMID: 10983759

976. Reiss AL, Eliez S, Schmitt JE, Patwardhan A, Haberecht M. Brain imaging in
neurogenetic conditions: realizing the potential of behavioral neurogenetics
research. Ment Retard Dev Disabil Res Rev. 2000;6(3):186-97. Review. PubMed
[citation] PMID: 10982496

977. Buzás György M, Székely E, Illyés G, Széles I. [Lansoprazole versus ranitidine
bismuth citrate containing triple regimens for Helicobacter pylori eradication in
patients with duodenal ulcer]. Orv Hetil. 2000 Jul 30;141(31):1711-4. Hungarian.
PubMed [citation] PMID: 10976194

978. Devereux RB, Roman MJ, Palmieri V, Okin PM, Boman K, Gerdts E, Nieminen MS,
Papademetriou V, Wachtell K, Dahlöf B. Left ventricular wall stresses and wall
stress-mass-heart rate products in hypertensive patients with
electrocardiographic left ventricular hypertrophy: the LIFE study. Losartan
Intervention For Endpoint reduction in hypertension. J Hypertens. 2000
Aug;18(8):1129-38. PubMed [citation] PMID: 10954006

979. Kroon ED, Ungsedhapand C, Ruxrungtham K, Chuenyam M, Ubolyam S, Newell ME, van
Leeuwen R, Kunanusont C, Buranapraditkul S, Sirivichayakul S, Lange JM, Cooper
DA, Phanuphak P. A randomized, double-blind trial of half versus standard dose of
zidovudine plus zalcitabine in Thai HIV-1-infected patients (study HIV-NAT 001).
HIV Netherlands Australia Thailand Research Collaboration. AIDS. 2000 Jul
7;14(10):1349-56. PubMed [citation] PMID: 10930149

980. Geschwind DH, Boone KB, Miller BL, Swerdloff RS. Neurobehavioral phenotype of
Klinefelter syndrome. Ment Retard Dev Disabil Res Rev. 2000;6(2):107-16. Review.
PubMed [citation] PMID: 10899803

981. Baxter JD, Mayers DL, Wentworth DN, Neaton JD, Hoover ML, Winters

MA, Mannheimer
SB, Thompson MA, Abrams DI, Brizz BJ, Ioannidis JP, Merigan TC. A randomized
study of antiretroviral management based on plasma genotypic antiretroviral
resistance testing in patients failing therapy. CPCRA 046 Study Team for the
Terry Beirn Community Programs for Clinical Research on AIDS. AIDS. 2000 Jun
16;14(9):F83-93. PubMed [citation] PMID: 10894268

982. Utriainen T, Lovisatti S, Mäkimattila S, Bertoldo A, Weintraub S, DeFronzo R,
Cobelli C, Yki-Järvinen H. Direct measurement of the lumped constant for
2-deoxy-[1-(14)C]glucose in vivo in human skeletal muscle. Am J Physiol
Endocrinol Metab. 2000 Jul;279(1):E228-33. PubMed [citation] PMID: 10893344

983. Gupta D, Sharma BS, Gupta SK, Bapuraj R, Khosla VK. Postoperative
hypertensive-hypervolaemic-haemodilution (Triple H) therapy in the treatment of
vasospasm following aneurysmal subarachnoid haemorrhage. Neurol India. 2000
Jun;48(2):126-31. PubMed [citation] PMID: 10878775

984. Boluyt N, Hack WW, Schrander-Stumpel CT. [Klinefelter syndrome in young children:
possibility of diagnosis]. Ned Tijdschr Geneeskd. 2000 Jun 10;144(24):1145-8.
Review. Dutch. PubMed [citation] PMID: 10876691

985. Patwardhan AJ, Eliez S, Bender B, Linden MG, Reiss AL. Brain morphology in
Klinefelter syndrome: extra X chromosome and testosterone supplementation.
Neurology. 2000 Jun 27;54(12):2218-23. PubMed [citation] PMID: 10881243

986. Hargreave TB. Genetics and male infertility. Curr Opin Obstet Gynecol. 2000
Jun;12(3):207-19. Review. PubMed [citation] PMID: 10873122

987. Shi Q, Martin RH. Multicolor fluorescence in situ hybridization analysis of
meiotic chromosome segregation in a 47,XYY male and a review of the literature.
Am J Med Genet. 2000 Jul 3;93(1):40-6. Review. Erratum in: Am J Med Genet. 2001

Feb 15;99(1):76. PubMed [citation] PMID: 10861680

988. Barreiro P, Soriano V, Blanco F, Casimiro C, de la Cruz JJ, González-Lahoz J.
Risks and benefits of replacing protease inhibitors by nevirapine in HIV-infected
subjects under long-term successful triple combination therapy. AIDS. 2000 May
5;14(7):807-12. PubMed [citation] PMID: 10839588

989. [No authors listed] AVANTI 2. Randomized, double-blind trial to evaluate the
efficacy and safety of zidovudine plus lamivudine versus zidovudine plus
lamivudine plus indinavir in HIV-infected antiretroviral-naive patients. AIDS.
2000 Mar 10;14(4):367-74. PubMed [citation] PMID: 10770538

990. Kitamura M, Matsumiya K, Koga M, Nishimura K, Miura H, Tsuji T, Matsumoto M,
Okamoto Y, Okuyama A. Ejaculated spermatozoa in patients with non-mosaic
Klinefelter's syndrome. Int J Urol. 2000 Mar;7(3):88-92; discussion 93-4. Review.
PubMed [citation] PMID: 10750887

991. Kovac AL. Prevention and treatment of postoperative nausea and vomiting. Drugs.
2000 Feb;59(2):213-43. Review. PubMed [citation] PMID: 10730546

992. Gilliland WR, Stashower ME. Klinefelter's syndrome and systemic lupus
erythematosus. Clin Exp Rheumatol. 2000 Jan-Feb;18(1):107-9. Review. No abstract
available. PubMed [citation] PMID: 10728455

993. Frick SL, Kim SS, Wenger DR. Pre- and postoperative three-dimensional computed
tomography analysis of triple innominate osteotomy for hip dysplasia. J Pediatr
Orthop. 2000 Jan-Feb;20(1):116-23. PubMed [citation] PMID: 10641700

994. Stark B, Sharon R, Rechavi G, Attias D, Ballin A, Cividalli G, Burstein Y,
Sthoeger D, Abramov A, Zaizov R. Effective preventive central nervous system
therapy with extended triple intrathecal therapy and the modified ALL-BFM 86
chemotherapy program in an enlarged non-high risk group of children and

adolescents with non-B-cell acute lymphoblastic leukemia: the Israel National
Study report. Cancer. 2000 Jan 1;88(1):205-16. PubMed [citation] PMID: 10618625
