# Supplementary material for: Evidence-based recommendations for delivering the diagnosis of X & Y chromosome multisomies in children, adolescents, and young adults: an integrative review
Source: BMC Pediatr. 2024 Apr 22;24:263. doi: 10.1186/s12887-024-04723-0 (PMC11034074; doi:10.1186/s12887-024-04723-0)
Supplement: Supplementary file 1 — Supplementary Material 1. [file 12887_2024_4723_MOESM1_ESM.zip › Data+note Evidence-Based recommendations for delivering the diagnosis.docx]

­­­------------------------------------------------------------------------------------------------------------------------------------------

**Data Notes for**

**Evidence-Based Recommendations for Delivering the Diagnosis of X & Y Chromosome Multisomies in Children, Adolescents, and Young Adults: An Integrative Review**

-------------------------------------------------------------------------------------------------------------------------------

Kirsten A. Riggan, MA, MS,^1^ Kelly E. Ormond, MS, CGC,^2,3^ Megan A. Allyse, PhD, and ^1,4,^ Sharron Close, PhD, MS,CPNP-PC, FAAN^5,6^

^1^Biomedical Ethics Research Program, Mayo Clinic, Rochester, MN

^2^Department of Health Sciences and Technology, Health Ethics and Policy Lab, ETH-Zurich, Zurich, Switzerland

^3^Department of Genetics, Stanford University School of Medicine, Stanford, CA

^4^Department of Obstetrics & Gynecology, Mayo Clinic, Rochester, MN

^5^Nell Hodgson Woodruff School of Nursing, Emory University, Atlanta GA

^6^Department of Human Genetics Emory School of Medicine Atlanta, GA

Corresponding Author:

Sharron Close

Emory University

1520 Clifton Road NE

Atlanta, GA 30342

Tel: (404) 727-7906

[SClose@emory.edu](mailto:SClose@emory.edu)

ORCIDs:

Kirsten A. Riggan: <https://orcid.org/0000-0002-0127-9555>

Kelly E. Ormond: <https://orcid.org/0000-0002-1033-0818>

Megan A. Allyse: <https://orcid.org/0000-0001-6136-9256>

Sharron Close: <https://orcid.org/0000-0002-2117-2165>

Kirsten Riggan: Riggan.kirsten@mayo.edu

Kelly Ormond: kelly.ormond@hest.ethz.ch

Megan Allyse: Allyse.megan@mayo.edu

**Abstract**

**Background**

Empirical evidence about sex chromosome aneuploidy suggests that the delivery of the diagnosis has a lasting impact on how affected individuals and caregivers perceive and adapt to the diagnosis.

**Objective**

The purpose of this review is to synthesize the literature to obtain useful recommendations for delivering a pediatric diagnosis of a sex chromosome multisomy (SCM) based upon a growing body of quantitative and qualitative literature on patient experiences.

**Methods/Data**

We conducted an integrative literature review using PubMed and CINAHL and Web of Science employing keywords “genetic diagnosis delivery,” “genetic diagnosis disclosure,” “sex chromosome aneuploidy,” “Klinefelter syndrome or 47, XXY,” “Jacob syndrome or 47, XYY,” and “Trisomy X, Triple X or 47, XXX, and 48 XXYY” from January 1, 2000, to October 31, 2023.

**Results**

Literature supports that patients and parents value the provision of up-to-date information and connection with supportive resources. Discussion includes relevant referrals, with tips about preventing perceptions of provider abandonment and commitment to ongoing support. Proactively addressing special concerns such as disclosing the diagnosis to their child, family, and community is also beneficial.

**Conclusion**

The synthesis of this data provides information resources that may be helpful to support patients, and addresses common misconceptions that interfere with accurate information about the diagnosis.

**Keywords**

X and Y chromosome variations; sex chromosome variations; sex chromosome aneuploidies; sex chromosome multisomies; genetics; diagnosis delivery

**Objective**

The purpose of this review is to synthesize the literature to obtain useful recommendations for delivering a pediatric diagnosis of a sex chromosome multisomy (SCM) based upon a growing body of quantitative and qualitative literature on patient experiences.

**Data description**

The data consists of the list of references that were full-text read by two reviewers for extracting information about disclosing the diagnosis of sex chromosome multisomy. Data show references used for background and relevance with 12 selected references that addressed diagnosis disclosure in sex chromosome multisomies.

We conducted an integrative review of the literature focused upon addressing the research question of what evidence exists to inform pediatric clinicians about disclosing and delivering the news that a child has an SCM. An initial search was done to extract key search terms. The search strategies used the following formula: genetic diagnosis disclosure OR genetic diagnosis delivery AND sex chromosome aneuploidy OR Klinefelter syndrome OR 47 XXY OR Jacob’s syndrome OR 47 XYY OR Trisomy X , OR Triple X, OR 47 XXX, OR XXYY. During October of 2023, the databases of PubMed, CINAHL and Web of Science searching the literature from January 1, 2000, to October 31, 2023. Selection of studies for this review were based on the inclusion criteria of relevance to disclosing a diagnosis in a child, adolescent, or young adult and family support post-diagnosis. Additionally, references within discovered articles were reviewed for comprehensiveness. Articles discussing a diagnosis of monosomy conditions (e.g., 45, XO) only were excluded from this review. For specificity in this article, we use the term “sex chromosome multisomies” rather than “sex chromosome aneuploidy,” which includes monosomy conditions beyond the scope of this review. This review was conducted according to PRISMA-Scoping Review criteria. (21, 22) Two reviewers (KR, SC) independently read and scored abstracts of articles according to inclusion criteria by labeling as either relevant or irrelevant to the research question. Full text articles were reviewed by KR and SC and consensus for inclusion were achieved through review with MA and KO. Data from the selected articles were entered into a chart to demonstrate relevant variables to extract. We employed narrative synthesis to summarize evidence that we organized into six main categories. Pediatric clinicians are increasingly responsible for delivering a diagnosis of SCM, we present useful recommendations, informed by this review, to be used for delivering a diagnosis of SCM to facilitate parent and patient adaptation to the diagnosis.

Data include references culled to categorize areas of information needs for providers to use in preparation furing diagnosis disclosure of sex chromosome multisomies.

**Table 1**: Overview of data files/data sets.

| **Label** | **Name of data file/data set** | **File types**  **(file extension)** | **Data repository and identifier (DOI or accession number)** |
| --- | --- | --- | --- |
| *Data file 1* | *Full-text reviewed references used in synthesis of information used to establish evidence-based recommendations for providers who deliver the diagnosis of sex chromosome aneuplodies to individuals and caregivvers* | *(.xlxs)* | *Figshare 10.6084/m9.figshare.24914073* |

**Limitations**

Limitations of integrative reviews include potential problems with accuracy, bias, or rigor as different methodologies are used across included studies. Additionally, given the relatively small number of studies about diagnosis disclosure in SCMs, our synthesis yields broad conclusions. The major strength of this review, however, is that it addresses a gap in the literature about evidence-based recommendations for genetic diagnosis disclosure in SCM and offers useful recommendations that can be used in practice by pediatric health care providers.

**Abbreviations**

SCM Sex chromosome aneuploidies

U.S. United States

IEP Individualized Education Plan

IDEA Individuals with Disabilities Act

AYA Adolescents and Young Adults

Declarations

**Ethics approval and consent to participate**

Not applicable

**Consent for publication**

Not Applicable

**Availability of data and materials**
The data described in this Data note can be freely and openly accessed on Figshare

10.6084/m9.figshare.24914073

Please see table 1 and references for details and links to the data.

**Competing interests**

The authors (KR, KO, MA and SC) have no competing interests or financial disclosures to make.

**Funding**

**This project was not funded**

**Authors’ contributions**

Kirsten Riggan, Kelley Ormond, Megan Allyse and Sharron Close gave equal contribution to the literature review process, synthesis of review, derivation of evidence-based findings, writing and editing the manuscript. All authors read and approved the final manuscript.

**Acknowledgements**

**None**

**References**

1. Milunsky A, Huang X, Amos JA, Herskowitz J, Farrer LA, Wyandt HE. 46,XY/47,XYY male with the fragile X syndrome: cytogenetic and molecular studies. Am J Med Genet. 1993;45(5):589-93.

2. Berglund A, Stochholm K, Gravholt CH. The epidemiology of sex chromosome abnormalities. Am J Med Genet C Semin Med Genet. 2020;184(2):202-15.

3. Ricciardi G, Cammisa L, Bove R, Picchiotti G, Spaziani M, Isidori AM, et al. Clinical, Cognitive and Neurodevelopmental Profile in Tetrasomies and Pentasomies: A Systematic Review. Children (Basel). 2022;9(11).

4. Tartaglia N, Ayari N, Howell S, D'Epagnier C, Zeitler P. 48,XXYY, 48,XXXY and 49,XXXXY syndromes: not just variants of Klinefelter syndrome. Acta Paediatr. 2011;100(6):851-60.

5. Otter M, Crins PML, Campforts BCM, Stumpel C, van Amelsvoort T, Vingerhoets C. Social functioning and emotion recognition in adults with triple X syndrome. BJPsych Open. 2021;7(2):e51.

6. Tartaglia N, Howell S, Davis S, Kowal K, Tanda T, Brown M, et al. Early neurodevelopmental and medical profile in children with sex chromosome trisomies: Background for the prospective eXtraordinarY babies study to identify early risk factors and targets for intervention. Am J Med Genet C Semin Med Genet. 2020;184(2):428-43.

7. Wigby K, D'Epagnier C, Howell S, Reicks A, Wilson R, Cordeiro L, Tartaglia N. Expanding the phenotype of Triple X syndrome: A comparison of prenatal versus postnatal diagnosis. Am J Med Genet A. 2016;170(11):2870-81.

8. Hong DS, Reiss AL. Cognitive and neurological aspects of sex chromosome aneuploidies. Lancet Neurol. 2014;13(3):306-18.

9. Bardsley MZ, Kowal K, Levy C, Gosek A, Ayari N, Tartaglia N, et al. 47,XYY syndrome: clinical phenotype and timing of ascertainment. J Pediatr. 2013;163(4):1085-94.

10. Cordeiro L, Tartaglia N, Roeltgen D, Ross J. Social deficits in male children and adolescents with sex chromosome aneuploidy: a comparison of XXY, XYY, and XXYY syndromes. Res Dev Disabil. 2012;33(4):1254-63.

11. Tartaglia NR, Howell S, Sutherland A, Wilson R, Wilson L. A review of trisomy X (47,XXX). Orphanet J Rare Dis. 2010;5:8.

12. Leggett V, Jacobs P, Nation K, Scerif G, Bishop DV. Neurocognitive outcomes of individuals with a sex chromosome trisomy: XXX, XYY, or XXY: a systematic review. Dev Med Child Neurol. 2010;52(2):119-29.

13. Bearelly P, Oates R. Recent advances in managing and understanding Klinefelter syndrome. F1000Res. 2019;8.

14. Close S, Talboy A, Fennoy I. Complexities of Care in Klinefelter Syndrome: An APRN Perspective. Pediatr Endocrinol Rev. 2017;14(Suppl 2):462-71.

15. Davis S, Howell S, Wilson R, Tanda T, Ross J, Zeitler P, Tartaglia N. Advances in the Interdisciplinary Care of Children with Klinefelter Syndrome. Adv Pediatr. 2016;63(1):15-46.

16. Urbanus E, van Rijn S, Swaab H. A review of neurocognitive functioning of children with sex chromosome trisomies: Identifying targets for early intervention. Clin Genet. 2020;97(1):156-67.

17. May C, Dein A, Ford J. New insights into the formation and duration of flashbulb memories: Evidence from medical disgnosis memories. Applied Cognitive Psychology. 2020;34:1154-65.

18. Riggan KA, Close S, Allyse MA. Family experiences and attitudes about receiving the diagnosis of sex chromosome aneuploidy in a child. Am J Med Genet C Semin Med Genet. 2020;184(2):404-13.

19. Jaramillo C, Nyquist C, Riggan KA, Egginton J, Phelan S, Allyse M. Delivering the Diagnosis of Sex Chromosome Aneuploidy: Experiences and Preferences of Parents and Individuals. Clin Pediatr (Phila). 2019;58(3):336-42.

20. Riggan KA, Gross B, Close S, Steinberg A, Allyse MA. "Knowledge is Power": Parent Views on the Benefits of Early Diagnosis and Awareness of Sex Chromosome Multisomy Among Pediatric Professionals. J Dev Behav Pediatr. 2023;44(2):e119-e25.

21. McGowan J, Straus S, Moher D, Langlois EV, O'Brien KK, Horsley T, et al. Reporting scoping reviews-PRISMA ScR extension. J Clin Epidemiol. 2020;123:177-9.

22. Tricco AC, Lillie E, Zarin W, O'Brien KK, Colquhoun H, Levac D, et al. PRISMA Extension for Scoping Reviews (PRISMA-ScR): Checklist and Explanation. Ann Intern Med. 2018;169(7):467-73.

23. Saul RA, Trotter T, Sease K, Tarini B. Survey of family history taking and genetic testing in pediatric practice. J Community Genet. 2017;8(2):109-15.

24. Rinke ML, Mikat-Stevens N, Saul R, Driscoll A, Healy J, Tarini BA. Genetic services and attitudes in primary care pediatrics. Am J Med Genet A. 2014;164A(2):449-55.

25. Berglund A, Viuff MH, Skakkebaek A, Chang S, Stochholm K, Gravholt CH. Changes in the cohort composition of turner syndrome and severe non-diagnosis of Klinefelter, 47,XXX and 47,XYY syndrome: a nationwide cohort study. Orphanet J Rare Dis. 2019;14(1):16.

26. Tremblay I, Grondin S, Laberge AM, Cousineau D, Carmant L, Rowan A, Janvier A. Diagnostic and Therapeutic Misconception: Parental Expectations and Perspectives Regarding Genetic Testing for Developmental Disorders. J Autism Dev Disord. 2019;49(1):363-75.

27. Botkin JR. Ethical issues in pediatric genetic testing and screening. Curr Opin Pediatr. 2016;28(6):700-4.

28. Loughry L, Pynaker C, White M, Halliday J, Hui L. State-wide increase in prenatal diagnosis of klinefelter syndrome on amniocentesis and chorionic villus sampling: Impact of non-invasive prenatal testing for sex chromosome conditions. Prenat Diagn. 2023;43(2):156-61.

29. Howard-Bath A, Poulton A, Halliday J, Hui L. Population-based trends in the prenatal diagnosis of sex chromosome aneuploidy before and after non-invasive prenatal testing. Prenat Diagn. 2018;38(13):1062-8.

30. Visootsak J, Ayari N, Howell S, Lazarus J, Tartaglia N. Timing of diagnosis of 47,XXY and 48,XXYY: a survey of parent experiences. Am J Med Genet A. 2013;161A(2):268-72.

31. Close S, Sadler L, Grey M. In the Dark: Challenges of Caring for Sons with Klinefelter Syndrome. J Pediatr Nurs. 2016;31(1):11-20.

32. Linden MG, Bender BG, Robinson A. Genetic counseling for sex chromosome abnormalities. Am J Med Genet. 2002;110(1):3-10.

33. Tartaglia N, Howell S, Wilson R, Janusz J, Boada R, Martin S, et al. The eXtraordinarY Kids Clinic: an interdisciplinary model of care for children and adolescents with sex chromosome aneuploidy. J Multidiscip Healthc. 2015;8:323-34.

34. Richardson JP, Ahlawat N, Riggan KA, Close S, Allyse MA. Experiences of individuals receiving a sex chromosome multisomy diagnosis. J Community Genet. 2022;13(6):619-28.

35. Richardson JP, Riggan KA, Allyse M. The Expert in the Room: Parental Advocacy for Children with Sex Chromosome Aneuploidies. J Dev Behav Pediatr. 2021;42(3):213-9.

36. Ashtiani S, Makela N, Carrion P, Austin J. Parents' experiences of receiving their child's genetic diagnosis: a qualitative study to inform clinical genetics practice. Am J Med Genet A. 2014;164A(6):1496-502.

37. Harrison ME, Walling A. What do we know about giving bad news? A review. Clin Pediatr (Phila). 2010;49(7):619-26.

38. Rosenzweig MQ. Breaking bad news: a guide for effective and empathetic communication. Nurse Pract. 2012;37(2):1-4.

39. VandeKieft GK. Breaking bad news. Am Fam Physician. 2001;64(12):1975-8.

40. Riggan KA, Gross B, Close S, Weinberg A, Allyse MA. Prenatal Genetic Diagnosis of a Sex Chromosome Aneuploidy: Parent Experiences. J Genet Couns. 2021;30(5):1407-17.

41. Bourke E, Snow P, Herlihy A, Amor D, Metcalfe S. A qualitative exploration of mothers' and fathers' experiences of having a child with Klinefelter syndrome and the process of reaching this diagnosis. Eur J Hum Genet. 2014;22(1):18-24.

42. Bourke E, Herlihy A, Snow P, Metcalfe S, Amor D. Klinefelter syndrome - a general practice perspective. Aust Fam Physician. 2014;43(1):38-41.

43. Davis SM, Soares K, Howell S, Cree-Green M, Buyers E, Johnson J, Tartaglia NR. Diminished Ovarian Reserve in Girls and Adolescents with Trisomy X Syndrome. Reprod Sci. 2020;27(11):1985-91.

44. Davis SM, Bloy L, Roberts TPL, Kowal K, Alston A, Tahsin A, et al. Testicular function in boys with 47,XYY and relationship to phenotype. Am J Med Genet C Semin Med Genet. 2020;184(2):371-85.

45. Davis SM, Rogol AD, Ross JL. Testis Development and Fertility Potential in Boys with Klinefelter Syndrome. Endocrinol Metab Clin North Am. 2015;44(4):843-65.

46. Stochholm K, Bojesen A, Jensen AS, Juul S, Gravholt CH. Criminality in men with Klinefelter's syndrome and XYY syndrome: a cohort study. BMJ Open. 2012;2(1):e000650.

47. Ridder LO, Berglund A, Stochholm K, Chang S, Gravholt CH. Morbidity, mortality, and socioeconomics in Klinefelter syndrome and 47,XYY syndrome: a comparative review. Endocr Connect. 2023;12(5).

48. Szigety KM, Crowley TB, Gaiser KB, Chen EY, Priestley JRC, Williams LS, et al. Clinical Effectiveness of Telemedicine-Based Pediatric Genetics Care. Pediatrics. 2022;150(1).

49. Variations AfXYC. Association for X & Y Variations (AXYS) 2023 [Available from: https://genetic.org/variations/.

50. Meredith S. Understanding Klinefelter Syndrome 2023 [Available from: http://understandingklinefeltersyndrome.org.

51. Thompson T, Davis S, Takamatsu S, Howell S, Tartaglia N. Exploring academic and character strengths in students with sex chromosome aneuploidies. Journal of Positive School Psychology. 2022;6:12-24.

52. Thompson T, Davis S, Janusz J, Frith E, Pyle L, Howell S, et al. Supporting students with sex chromosome aneuploidies in educational settings: Results of a nationwide survey. J Sch Psychol. 2022;93:28-40.

53. AXYS Clinic and Research Consortium [Available from: https://genetic.org/im-adult-looking-answers/clinics/acrc-clinics-list/.

54. Thompson T, Stinnett N, Tartaglia N, Davis S, Janusz J. 'I Wish the School Had a Better Understanding of the Diagnosis': parent perspectives on educational needs of students with sex chromosome aneuploidies. J Res Spec Educ Needs. 2022;22(2):175-87.

55. Zganjar A, Nangia A, Sokol R, Ryabets A, Samplaski MK. Fertility in Adolescents With Klinefelter Syndrome: A Survey of Current Clinical Practice. J Clin Endocrinol Metab. 2020;105(4).

56. Dwyer AA, Heritier V, Llahana S, Edelman L, Papadakis GE, Vaucher L, et al. Navigating Disrupted Puberty: Development and Evaluation of a Mobile-Health Transition Passport for Klinefelter Syndrome. Front Endocrinol (Lausanne). 2022;13:909830.

57. Close S, Howell S, Talboy A, Cover V, R. M, Meerschaert C. Developing a model for the transition from pediatric to adult care 2018 [Available from: https://genetic.org/axys-awarded-grant-develop-adult-specialty-clinics/.

58. Turriff A, Macnamara E, Levy HP, Biesecker B. The Impact of Living with Klinefelter Syndrome: A Qualitative Exploration of Adolescents and Adults. J Genet Couns. 2017;26(4):728-37.

59. Klitzman R. "Am I my genes?": Questions of identity among individuals confronting genetic disease. Genet Med. 2009;11(12):880-9.

60. McConkie-Rosell A, Spiridigliozzi GA, Melvin E, Dawson DV, Lachiewicz AM. Living with genetic risk: effect on adolescent self-concept. Am J Med Genet C Semin Med Genet. 2008;148C(1):56-69.

61. Ahlawat N, Elliott K, Ormond KE, Allyse MA, Riggan KA. Healthcare and support experiences of adolescents and young adults diagnosed with 47,XXY, 47,XXX, and 48,XXYY. J Community Genet. 2023.

62. Committee on Bioethics and The American College of Medical Genetics and Genomics Social EaLIC. Ethical and ploicy issues in genetic testing and screening of chidren. Pediatrics. 2013;131:620-2.

63. Close S, Fennoy I, Smaldone A, Reame N. Phenotype and Adverse Quality of Life in Boys with Klinefelter Syndrome. J Pediatr. 2015;167(3):650-7.

64. Dennis A, Howell S, Cordeiro L, Tartaglia N. "How should I tell my child?" Disclosing the diagnosis of sex chromosome aneuploidies. J Genet Couns. 2015;24(1):88-103.

65. Turriff A, Levy HP, Biesecker B. Factors associated with adaptation to Klinefelter syndrome: the experience of adolescents and adults. Patient Educ Couns. 2015;98(1):90-5.

66. Aliberti L, Gagliardi I, Bigoni S, Lupo S, Caracciolo S, Ferlini A, et al. Communicating the diagnosis of Klinefelter syndrome to children and adolescents: when, how, and who? J Community Genet. 2022;13(3):271-80.

67. Gratton NC, Myring J, Middlemiss P, Shears D, Wellesley D, Wynn S, et al. Children with sex chromosome trisomies: parental disclosure of genetic status. Eur J Hum Genet. 2016;24(5):638-44.

68. Tremblay I, Van Vliet G, Gonthier M, Janvier A. Partnering with parents to disclose Klinefelter syndrome to their child. Acta Paediatr. 2016;105(5):456-61.

69. Bester J, Sabatello M, van Karnebeek CDM, Lantos JD. Please Test My Child for a Cancer Gene, but Don't Tell Her. Pediatrics. 2018;141(4).

70. Cole CM, Kodish E. Minors' right to know and therapeutic privilege. Virtual Mentor. 2013;15(8):638-44.

71. Hudson N, Spriggs M, Gillam L. Telling the truth to young children: Ethical reasons for information disclosure in paediatrics. J Paediatr Child Health. 2019;55(1):13-7.

72. Metcalfe A, Coad J, Plumridge GM, Gill P, Farndon P. Family communication between children and their parents about inherited genetic conditions: a meta-synthesis of the research. Eur J Hum Genet. 2008;16(10):1193-200.

73. Metcalfe A, Plumridge G, Coad J, Shanks A, Gill P. Parents' and children's communication about genetic risk: a qualitative study, learning from families' experiences. Eur J Hum Genet. 2011;19(6):640-6.
