# Supplementary material for: Evidence-based recommendations for delivering the diagnosis of X & Y chromosome multisomies in children, adolescents, and young adults: an integrative review
Source: BMC Pediatr. 2024 Apr 22;24:263. doi: 10.1186/s12887-024-04723-0 (PMC11034074; doi:10.1186/s12887-024-04723-0)
Supplement: Supplementary file 1 — Supplementary Material 1. [file 12887_2024_4723_MOESM1_ESM.zip › R-1 DIagnosis Disclosure in SCM 02.19.24 PRISMA-ScR Checklist_10Sept2019.docx]

**Preferred Reporting Items for Systematic reviews and Meta-Analyses extension for Scoping Reviews (PRISMA-ScR) Checklist for “Evidence-Based Recommendations for Delivering the Diagnosis of X & Y Chromosome Multisomies in Children, Adolescents, and Young Adults: An Integrative Review”**

| **SECTION** | **ITEM** | **PRISMA-ScR CHECKLIST ITEM** | **REPORTED ON PAGE #** |
| --- | --- | --- | --- |
| **TITLE** | | | |
| Title | 1 | Identify the report as a scoping review. This is an Integrative Review using PRISMA-ScR Guidelines  **We used on Integrative Review method as we were gathering evidence for application in practice and not to map the literature.** | Page 1 title, Page 3 abstract, Page 3 Methods |
| **ABSTRACT** | | | |
| Structured summary | 2 | Provide a structured summary that includes (as applicable): background, objectives, eligibility criteria, sources of evidence, charting methods, results, and conclusions that relate to the review questions and objectives. | Page 2,3 Abstract |
| **INTRODUCTION** | | | |
| Rationale | 3 | Describe the rationale for the review in the context of what is already known. Explain why the review questions/objectives lend themselves to a scoping review approach.  **While many excellent review articles exist on topics SCM, none as yet have focused upon the process of delivering the diagnosis. As well, no specific protocols or guidelines on diagnosis delivery can be found among national societies including the National Society of Genetic Counselors, the American College of Medical Genetics or the National Association of X & Y Chromosome Variations (AXYS). We endeavored to address this gap by gathering evidence that would provide guidance on how to disclose the diagnosis of SCM to individuals or caregivers.**  We chose to employ an **integrative literature** review method to provide a more comprehensive understanding of this particular healthcare problem and to provide methodological structure using the recommendations of Whittemore & Knafl. **While a scoping review aims to map the literature to classify results, this review focuses on synthesis of results that can be applied in practice** | Page 7 Background  Page 7  Methods |
| Objectives | 4 | Provide an explicit statement of the questions and objectives being addressed with reference to their key elements (e.g., population or participants, concepts, and context) or other relevant key elements used to conceptualize the review questions and/or objectives.  Objective was to synthesize current literature on diagnosis disclosure of SCM to parents and patients and to offer recommendations for pediatric providers to use when delivering this news.  RQ: What is known about disclosing the diagnosis of SCM to patients and caregivers?  RQ: What recommendations can be offered to clinicians about disclosing SCM to patients and caregivers? | Page 5 Introduction |
| **METHODS** | | | |
| Protocol and registration | 5 | Indicate whether a review protocol exists; state if and where it can be accessed (e.g., a Web address); and if available, provide registration information, including the registration number. | No protocol other than PRISMA for ScR |
| Eligibility criteria | 6 | Specify characteristics of the sources of evidence used as eligibility criteria (e.g., years considered, language, and publication status), and provide a rationale. | Page 7 & 8 Methods |
| Information sources* | 7 | Describe all information sources in the search (e.g., databases with dates of coverage and contact with authors to identify additional sources), as well as the date the most recent search was executed. | Page 7 & 8 Methods |
| Search | 8 | Present the full electronic search strategy for at least 1 database, including any limits used, such that it could be repeated. | Included in supplementary data |
| Selection of sources of evidence† | 9 | State the process for selecting sources of evidence (i.e., screening and eligibility) included in the scoping review. | Page 7 & 8 methods |
| Data charting process‡ | 10 | Describe the methods of charting data from the included sources of evidence (e.g., calibrated forms or forms that have been tested by the team before their use, and whether data charting was done independently or in duplicate) and any processes for obtaining and confirming data from investigators. | Page 8 & 9 Methods |
| Data items | 11 | List and define all variables for which data were sought and any assumptions and simplifications made. | Page 7 Methods |
| Critical appraisal of individual sources of evidence§ | 12 | If done, provide a rationale for conducting a critical appraisal of included sources of evidence; describe the methods used and how this information was used in any data synthesis (if appropriate). | No critical appraisal performed |
| Synthesis of results | 13 | Describe the methods of handling and summarizing the data that were charted. | Page 7 Methods |
| **RESULTS** | | | |
| Selection of sources of evidence | 14 | Give numbers of sources of evidence screened, assessed for eligibility, and included in the review, with reasons for exclusions at each stage, ideally using a flow diagram. | Page 9 Methods |
| Characteristics of sources of evidence | 15 | For each source of evidence, present characteristics for which data were charted and provide the citations. | Table 1 |
| Critical appraisal within sources of evidence | 16 | If done, present data on critical appraisal of included sources of evidence (see item 12). | No critical appraisal was performed |
| Results of individual sources of evidence | 17 | For each included source of evidence, present the relevant data that were charted that relate to the review questions and objectives. | Table 1 |
| Synthesis of results | 18 | Summarize and/or present the charting results as they relate to the review questions and objectives. | Pages 9-11 Results organized by categories |
| **DISCUSSION** | | | |
| Summary of evidence | 19 | Summarize the main results (including an overview of concepts, themes, and types of evidence available), link to the review questions and objectives, and consider the relevance to key groups. | Page 11 Discussion |
| Limitations | 20 | Discuss the limitations of the scoping review process. | Page 23 Limitations |
| Conclusions | 21 | Provide a general interpretation of the results with respect to the review questions and objectives, as well as potential implications and/or next steps. | Page 24 Conclusions |
| **FUNDING** | | | |
| Funding | 22 | Describe sources of funding for the included sources of evidence, as well as sources of funding for the scoping review. Describe the role of the funders of the scoping review. | No funding source. Statement appears in the Declarations statement |

JBI = Joanna Briggs Institute; PRISMA-ScR = Preferred Reporting Items for Systematic reviews and Meta-Analyses extension for Scoping Reviews.

* Where *sources of evidence* (see second footnote) are compiled from, such as bibliographic databases, social media platforms, and Web sites.

† A more inclusive/heterogeneous term used to account for the different types of evidence or data sources (e.g., quantitative and/or qualitative research, expert opinion, and policy documents) that may be eligible in a scoping review as opposed to only studies. This is not to be confused with *information sources* (see first footnote).

‡ The frameworks by Arksey and O’Malley (6) and Levac and colleagues (7) and the JBI guidance (4, 5) refer to the process of data extraction in a scoping review as data charting*.*

§ The process of systematically examining research evidence to assess its validity, results, and relevance before using it to inform a decision. This term is used for items 12 and 19 instead of "risk of bias" (which is more applicable to systematic reviews of interventions) to include and acknowledge the various sources of evidence that may be used in a scoping review (e.g., quantitative and/or qualitative research, expert opinion, and policy document).

*From:* Tricco AC, Lillie E, Zarin W, O'Brien KK, Colquhoun H, Levac D, et al. PRISMA Extension for Scoping Reviews (PRISMAScR): Checklist and Explanation. Ann Intern Med. 2018;169:467–473. [doi: 10.7326/M18-0850](http://annals.org/aim/fullarticle/2700389/prisma-extension-scoping-reviews-prisma-scr-checklist-explanation).
