# Supplementary figures and images for: Evidence-based recommendations for delivering the diagnosis of X & Y chromosome multisomies in children, adolescents, and young adults: an integrative review
Source: BMC Pediatr. 2024 Apr 22;24:263. doi: 10.1186/s12887-024-04723-0 (PMC11034074; doi:10.1186/s12887-024-04723-0)

PubMed Search

**
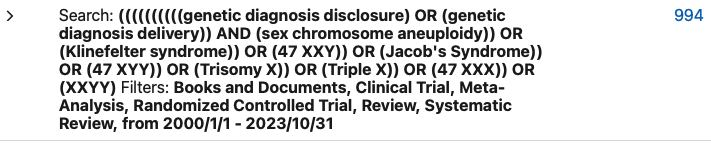
**

**CINAHL**

**
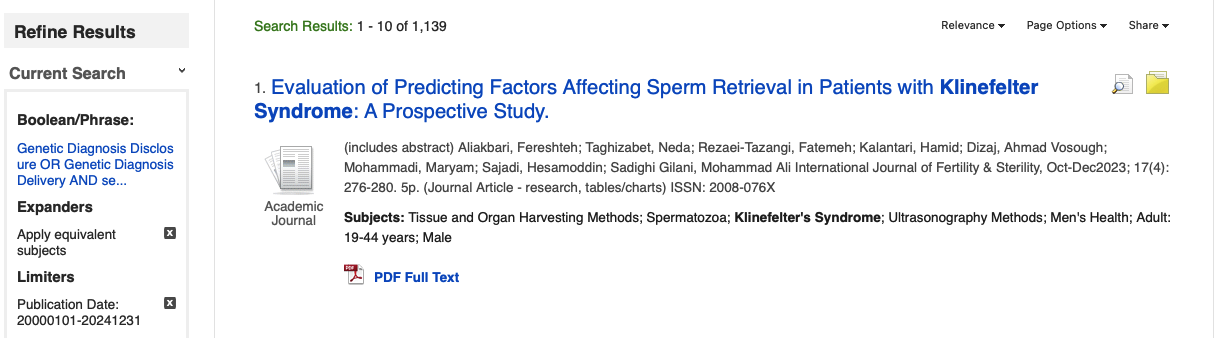
**

Supplement: Supplementary file 1 — Supplementary Material 1. [file 12887_2024_4723_MOESM1_ESM.zip › PubMed and CINAHL Strategy .docx]
